# Adaptive responses of carbon and nitrogen metabolisms to nitrogen-deficiency in *Citrus sinensis* seedlings

**DOI:** 10.1186/s12870-022-03759-7

**Published:** 2022-07-26

**Authors:** Wei-Tao Huang, Zhi-Chao Zheng, Dan Hua, Xu-Feng Chen, Jiang Zhang, Huan-Huan Chen, Xin Ye, Jiu-Xin Guo, Lin-Tong Yang, Li-Song Chen

**Affiliations:** Department of Resources and Environment, College of Resources and Environment, Fujian Agriculture and Forestry University, 15 Shangxiadian Road, Cangshan District, Fuzhou, 350002 China

**Keywords:** Amino acids, *Citrus sinensis*, Carbon metabolism, Nitrogen-deficiency, Nitrogen metabolism, Organic acids

## Abstract

**Background:**

In China, nitrogen (N)-deficiency often occurs in *Citrus* orchards, which is one of the main causes of yield loss and fruit quality decline. Little information is known about the adaptive responses of *Citrus* carbon (C) and N metabolisms to N-deficiency. Seedlings of ‘Xuegan’ (*Citrus sinensis* (L.) Osbeck) were supplied with nutrient solution at an N concentration of 0 (N-deficiency), 5, 10, 15 or 20 mM for 10 weeks. Thereafter, we examined the effects of N supply on the levels of C and N in roots, stems and leaves, and the levels of organic acids, nonstructural carbohydrates, NH_4_^+^-N, NO_3_^−^-N, total soluble proteins, free amino acids (FAAs) and derivatives (FAADs), and the activities of key enzymes related to N assimilation and organic acid metabolism in roots and leaves.

**Results:**

N-deficiency elevated sucrose export from leaves to roots, C and N distributions in roots and C/N ratio in roots, stems and leaves, thus enhancing root dry weight/shoot dry weight ratio and N use efficiency. N-deficient leaves displayed decreased accumulation of starch and total nonstructural carbohydrates (TNC) and increased sucrose/starch ratio as well as a partitioning trend of assimilated C toward to sucrose, but N-deficient roots displayed elevated accumulation of starch and TNC and reduced sucrose/starch ratio as well as a partitioning trend of assimilated C toward to starch. N-deficiency reduced the concentrations of most FAADs and the ratios of total FAADs (TFAADs)/N in leaves and roots. N-deficiency reduced the demand for C skeleton precursors for amino acid biosynthesis, thus lowering TFAADs/C ratio in leaves and roots. N-deficiency increased (decreased) the relative amounts of C-rich (N-rich) FAADs, thus increasing the molar ratio of C/N in TFAADs in leaves and roots.

**Conclusions:**

Our findings corroborated our hypothesis that C and N metabolisms displayed adaptive responses to N-deficiency in *C. sinensis* seedlings, and that some differences existed between roots and leaves in N-deficiency-induced alterations of and C and N metabolisms.

**Supplementary Information:**

The online version contains supplementary material available at 10.1186/s12870-022-03759-7.

## Background

Nitrogen (N) availability is one of the main factors limiting crop yield and quality including *Citrus* [1]. In order to meet the growing food demand of the global population, applying N fertilizers to crops has become a conventional agricultural practice to improve crop yield. Moreover, the application of N fertilizers is usually excessive [2]. The world N fertilizer application increased from 11.4 Tg year^− 1^ in 1961 to 107.7 Tg year^− 1^ in 2019 [3]. Reducing the application of N fertilizers without affecting crop yield is an urgent challenge for agriculture. Therefore, it is very important to enhance N use efficiency (NUE) of crops [4, 5]. Carbon (C) and N are the basic building blocks required for biomass accumulation and yield formation of crops [6]. C and N metabolisms are highly interlinked in plants. Inorganic N is necessary to allow carbohydrates to be used for growth, and photosynthesis or carbohydrate degradation provides reducing power, energy (ATP) and C skeletons to support inorganic N assimilation and N-containing compound biosynthesis [6–8]. C/N ratio of an organ is often considered to be a convenient indicator of growth and quality [9]. Increasing NUE in source leaves will increase the biomass produced per unit N, which is related to the higher ratio of C/N in plant materials [10]. A comprehensive understanding of the adaptive responses of C and N metabolisms to N-deficiency is a key for the improvement of crop yield and NUE as well as the reduction of N fertilizer application.

Carbon partitioning between roots and shoots is one of the major factors determining shoot growth [11]. Most experiments under controlled or field conditions showed that N-deficiency increased the partitioning of C to roots, thus increasing the ratio of root/shoot (R/S) [3, 12–18].

Nitrogen metabolism includes N uptake, reduction and assimilation, amino acid (AA) metabolism and transport, and N transport and remobilization [5, 19, 20]. Nitrate (NO_3_^−^) or ammonium (NH_4_^+^) are usually the main form of N uptake by plants from the soil. Within plant cells, NO_3_^−^ is reduced to NH_4_^+^ by nitrate reductase (NR) and nitrite reductase (NiR). NH_4_^+^ is assimilated to glutamine (Gln) and glutamate (Glu) by glutamine synthase (GS) and NADH-dependent glutamate 2-oxoglutarate aminotransferase (NADH-GOGAT), respectively [21]. Glutamate pyruvate aminotransferase (GPT) and glutamate oxaloacetate aminotransferase (GOT), two enzymes involved in the process of ammonia transfer, play a key role in the biosynthesis of amino acids (AAs) [22]. Evidence shows that N-deficiency has a great impact on N uptake and assimilation and AA biosynthesis, which in turn affects C assimilation and ultimately limits crop growth [3, 6, 23–25]. However, the results on N-deficiency-induced alterations of N metabolisms in plants are not consistent. Luo et al. [26] showed that poplar slowed up N acquisition and assimilation in the adaptation process to N limitation. Amiour et al. [27] reported that N-deficiency decreased the concentrations of most marker traits representative of the plant N status by 2–4 fold, including total N, NO_3_^−^-N, proteins, chlorophylls (Chls) and free AAs (FAAs) in maize. Further metabolite profile analysis indicated that most FAAs (18 out of 22) and many N-containing compounds biosynthesized from Gln and Glu, such as γ-aminobutyric acid (GABA), was decreased by 4–37 fold in N-deficient leaves. Ganie et al. [28] observed that most of FAAs and N-containing compounds were significantly decreased in N-starved maize roots and leaves. Low-N-induced an decrease in the concentration of FAAs was the greatest in young leaves, followed by mature leaves of oilseed rape; the concentration of FAAs did not differ significantly between low- and high-N treated old leaves [29]. N-deficiency-induced a decrease in the biosynthesis of AAs has also been observed in N-deficient roots, leaves and/or shoots of foxtail millet [16], *Arabidopsis* [30], maize [8], tomato [31], apple [32] and poplar [4, 21]. However, early responses to low-N in barley leaves led to an increased accumulation of FAAs [12]. Ganie et al. [33] reported that N-deficiency-induced alterations of FAAs profiles in the roots and leaves of low-N-sensitive and low-N-tolerant maize genotypes depended on the genotype and the duration of N-deficiency. Liu et al. [34] reported that the abundances of FAAs such as aspartic acid (Asp), alanine (Ala), serine (Ser), isoleucine (Ile) and threonine (Thr) increased in response to low-N in common wild soybean (W1) roots, but they displayed a decreased trend in low-N-tolerant wild soybean (W2) roots, concluding that W2 could tolerate low-N by reducing AA biosynthesis and consequently lowering energy consumption. N-deficiency reduced the accumulation of most FAAs in leaves, but it increased the accumulation of FAAs in roots of tea [25].

Nitrogen availability is the main factor influencing C assimilation and accumulation, and sufficient C level can improve N utilization of crops [21]. Ganie et al. [28] reported that low-N largely reduced the concentrations of major organic acids (OAs) involved in tricarboxylic acid (TCA) cycle, particularly isocitric acid, malic acid, succinate, and ketoglutaric acid, but increased soluble sugars in maize roots and leaves. N-starvation led to a general decrease in organic acid (OA) pools of maize resource leaves [8] and tomato roots and leaves [31]. Scheible et al. [35] showed that low-N reduced the concentrations of α-oxoglutarate, isocitrate, malate and citrate, and increased the concentration of starch in tobacco leaves. However, a 7-day N-deficient treatment led to increased concentrations of sugars and TCA cycle intermediates in barley leaves [12]. N-deficiency increased the abundances of OAs in leaves and most sugars and OAs in roots of tea [25]. Low-N downregulated and upregulated TCA cycle in apple leaves and roots, respectively [32]. Liu et al. [34] detected 25 differentially abundant OAs in wild soybean roots, 17 of which increased in W2, and 20 reduced in W1; and 12 sugar alcohols, 2 of which increased in the two soybean genotypes, and 10 displayed a decreased trend, with a greater decrease in W1 than in W2. In tobacco leaves, N-deficiency-induced accumulation of sugars was associated with reduced utilization for N assimilation (AA biosynthesis) [36]. Meng et al. [37] observed that low-N increased the concentrations of C, sucrose and glucose in roots and decreased the concentrations of C and sucrose in leaves of *Populus simonii* (Ps), but had no influence on their concentrations in roots and leaves of *Populus euramericana* (Pe) with the only exception that low-N reduced foliar C concentration. Low-N increased the concentration of total soluble sugars in foxtail millet roots and shoots [16]. N-deficiency increased the concentrations of starch and sugars in leaves, stems and roots of *Melaleuca leucadendra*, *Melaleuca cajuputi*, *Eucalyptus camaldulensis* and *Eucalyptus tereticornis* except for decreased sugar concentration in *M. cajuputi* stems [38]. In tomato, N-deficiency reduced carbohydrates by 25–50% in leaves, but increased them by several-fold in roots [31]. In apple, Zhao et al. [39] observed that low-N increased sucrose, sorbitol, glucose-6-phosphate and fructose-6-phosphate concentrations, reduced fructose, glucose-1-phosphate and starch concentrations, and did not affect glucose concentration in fine roots, while it increased glucose concentration, reduced fructose-6-phosphate, glucose-1-phosphate and glucose-6-phosphate concentrations, and had no impact on sucrose, sorbitol, fructose and starch concentrations in mature leaves, concluding that low-N improved sugar metabolism capability and sink strength in roots.

Although the adaptive responses of C and N metabolisms to N-deficiency have been investigated in some detail, the results in different plants are not consistent. Further studies on diverse plants are needed to answer the questions. So far, little information is available on the adaptive responses of *Citrus* C and N metabolisms to N-deficiency [3]. *Citrus* is one of the most important economic fruit trees in China. N-deficiency is one of the major factors affecting yield and quality of *Citrus* fruits [1, 40]. Based on the previous study [3], we examined the effects of N-deficiency on the concentrations of C and N in roots, stems and leaves, and the concentrations of OAs, nonstructural carbohydrates, NH_4_^+^-N, NO_3_^−^-N, total soluble proteins (TSPs), FAAs and derivatives (FAADs), and the activities of key enzymes related to N assimilation and OA metabolism in roots and leaves of *Citrus sinensis* seedlings. The objective of this study was to test the hypothesis that C and N metabolisms displayed adaptive responses to N-deficiency in *C. sinensis* seedlings, and that some differences existed between roots and leaves in N-deficiency-induced alterations of C and N metabolisms.

## Results

### Effects of N supply on C and N in leaves, stems and roots

**Fig. 1:**
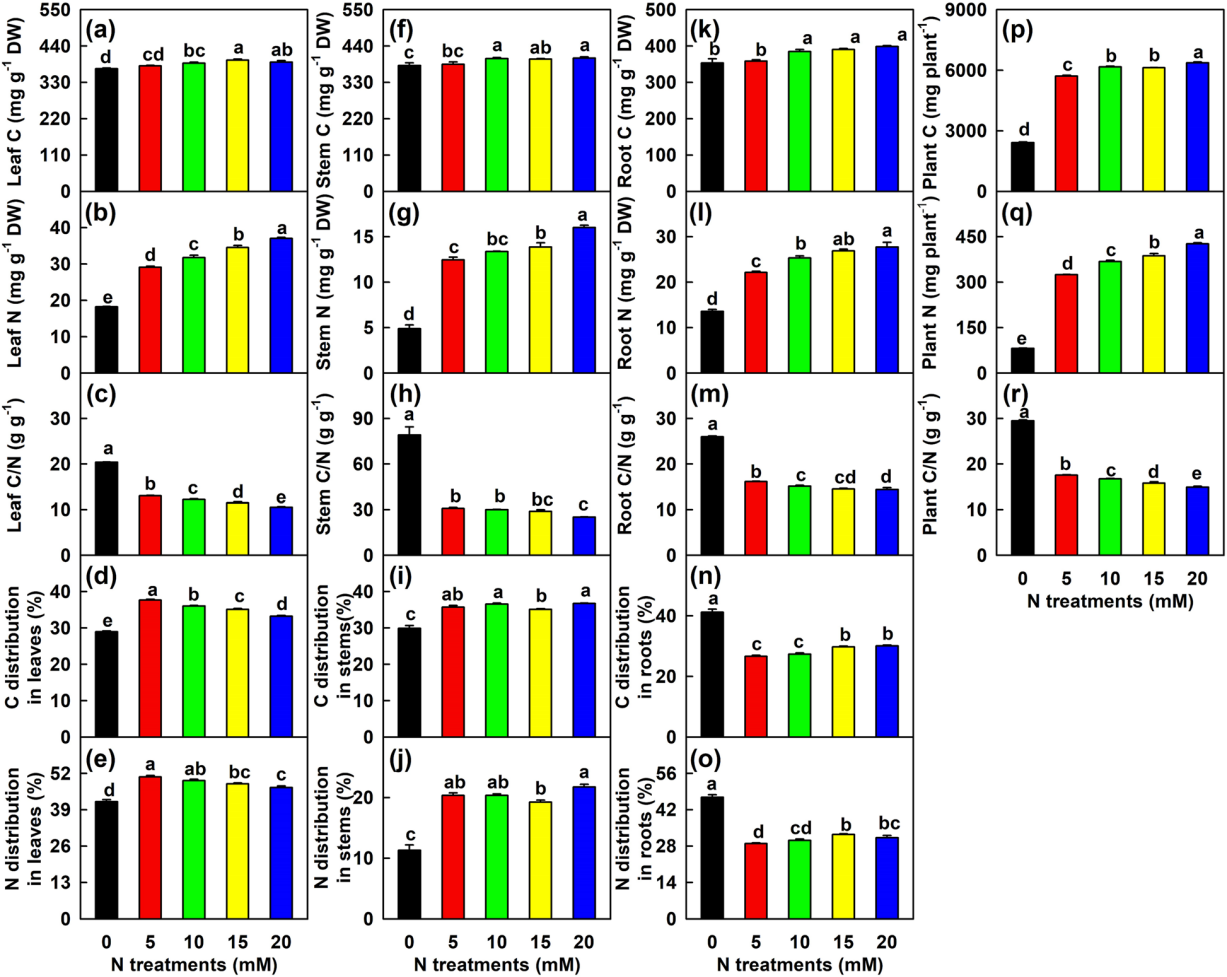
Effects of N supply on mean (± SE, *n* = 4) concentrations of C and N, ratio of C/N, and distributions of N in *C. sinensis* leaves, stems and roots, contents of C and N, and ratio of C/N in plants. **a**, **f** and **k** Leaf, stem and root C concentrations. **b**, **g** and **l** Leaf, stem and root N concentrations. **c**, **h** and **m** C/N ratios in leaves, stems and roots. **d**, **i** and **n** C distributions in leaves, stems and roots. **e**, **j** and **o** N distributions in leaves, stems and roots. **p**, **q** and **r** Plant C content, N content and C/N ratio. Different letters above the bars indicate a significant difference at *P* < 0.05. The same notation will be used in Figs. 2-6

With the increase of N supply, C and N concentrations in leaves, stems and roots increased, while C/N ratio decreased. With N-deficiency, C concentration reduced by 5.0, 5.6 and 11.3% in leaves, stems and roots, respectively, and N concentration by 50.8, 69.5 and 51.0% in leaves, stems and roots, respectively, and C/N ratio increased by 93.2, 214.8 and 80.5% in leaves, stems and roots, respectively, relative to 20 mM N. N-deficiency led to decreased C and N distributions by 12.9 and 10.8% in leaves and 18.6 and 48.0% in stems, respectively relative to 20 mM N; while it led to increased C and N distributions in roots by 36.9 and 49.7%, respectively. N-deficiency reduced C and N content per plant, but increased the ratio of C content per plant/N content per plant (hereinafter referred to as plant C/N; Fig. 1).

### Effects of N supply on NH_4_^+^-N, NO_3_^−^-N, TSPs, FAADs, and N-metabolism-related enzymes in leaves and roots

**Fig. 2:**
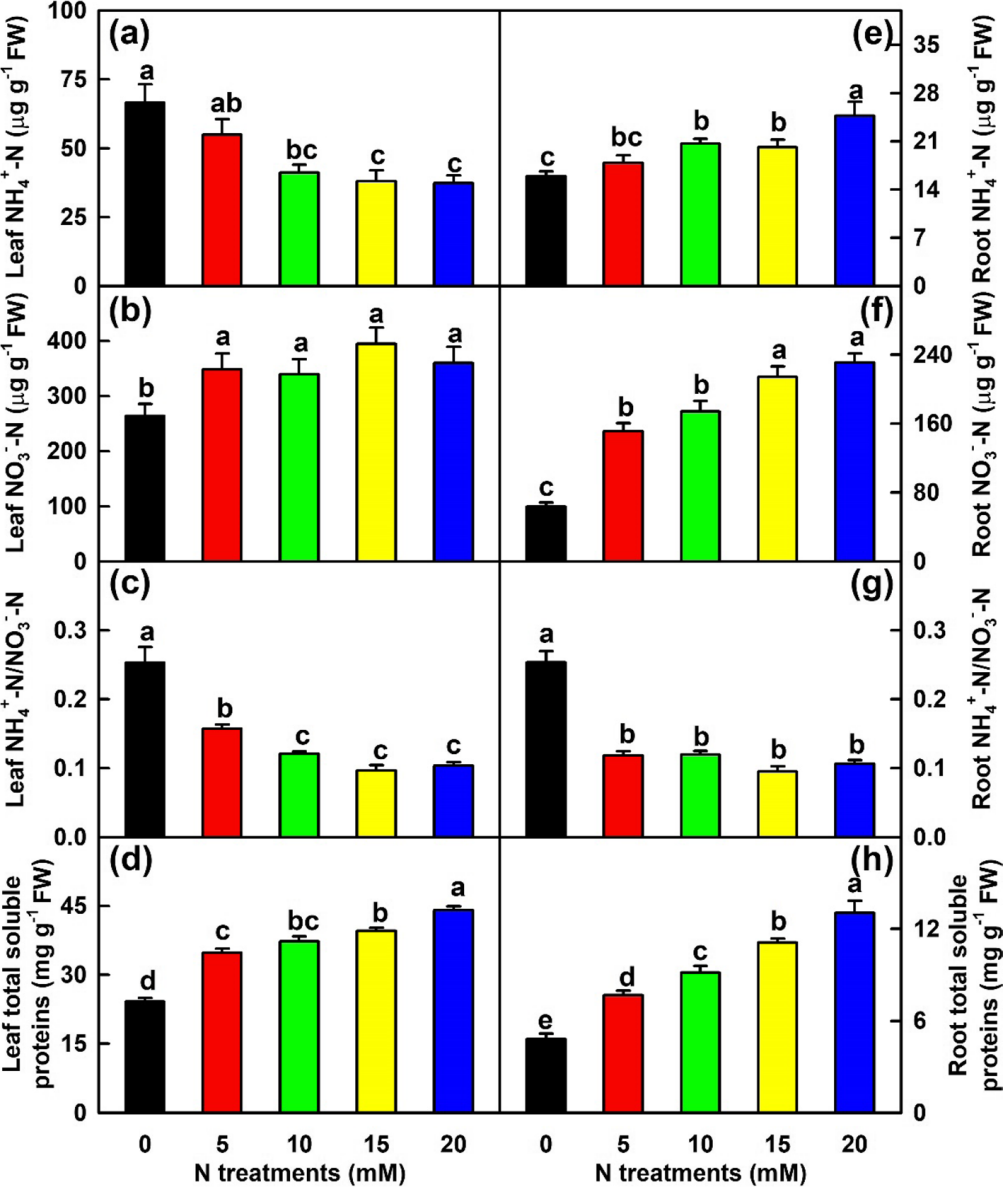
Effects of N supply on mean (± SE, *n* = 4) concentrations of NH_4_^+^-N, NO_3_^−^-N, ratios of NH_4_^+^-N/NO_3_^−^-N, and concentrations of TSPs in leaves and roots. **a** and **e** Leaf and root NH_4_^+^-N concentrations. **b** and **f** Leaf and root NO_3_^−^-N concentrations. **c** and **g** Leaf and root ratios of NH_4_^+^-N/NO_3_^−^-N. **d** and **h** Leaf and root concentrations of total soluble proteins (TSPs)

With the increase of N supply, NH_4_^+^-N concentration in leaves decreased, while NH_4_^+^-N and NO_3_^−^-N concentrations in roots increased. N-deficiency caused a reduction of NO_3_^−^-N concentration in leaves by 26.7% relative to 20 mM N. Interestingly, N-deficiency led to increased ratio of NH_4_^+^-N/NO_3_^−^-N ratio by 143.7% in leaves and 138.0% in roots relative to 20 mM N. The concentrations of TSPs in roots and leaves increased with the increase of N supply (Fig. 2).

In leaves, we detected 63 FAADs, 46 of which decreased, nine (Ile, Trp, 5-HTP, trimethylamine N-oxide, N8-acetylspermidine, succinic acid, creatine phosphate, kynurenic acid and Cys) increased, and eight (Val, Phe, N-acetylaspartate, N-glycyl-L-Leucine, N-acetyl-L-tyrosine, L-carnosine, α-aminoadipic acid and creatine) did not significantly alter in response to N-deficiency relative to 20 mM N (Table 1 and Fig. S1). In roots, we detected 66 FAADs, 50 of which decreased, four (L-cystathionine, trimethylamine N-oxide, succinic acid and α-aminoadipic acid) increased, and 12 (Val, Trp, 5-HTP, 3-N-methyl-L-histidine, L-tyrosine methyl ester, Nα-acetyl-L-arginine, D-Ala-D-Ala, 2-aminobutyric acid, 4-acetamidobutyric acid, kynurenic acid, Cys and creatine) did not significantly change in response to N-deficiency relative to 20 mM N (Table 2 and Fig. S1). N-deficiency led to decreased concentration of total FAADs (TFAADs) and ratio of TFAADs/N by 79.3 and 58.3% in leaves, respectively and 73.2 and 45.3% in roots, respectively relative to 20 mM N, but increased molar ratio of C/N in TFAADS in leaves and roots by 28.2 and 10.6%, respectively (Tables 1-2 and Fig. S2).

**Table 1:** Effects of N supply on mean (±SE, *n* = 3) concentrations (μg g^− 1^ FW) of FAADs as well as their summation in *Citrus sinensis* leaves

FAADs	Molecular formula	N treatments (mM)				
		0	5	10	15	20
N-Acetyl-L-tyrosine (NAT)	C_11_H_13_NO_4_	0.143 ± 0.007b	0.167 ± 0.014ab	0.172 ± 0.001ab	0.177 ± 0.008a	0.175 ± 0.015ab
L-tyrosine methyl ester (H-Tyr-OMe)	C_10_H_14_NO_3_	0.068 ± 0.007c	0.104 ± 0.008b	0.128 ± 0.009a	0.140 ± 0.006a	0.141 ± 0.002a
Kynurenic acid (KYNA)	C_10_H_7_NO_3_	0.527 ± 0.038a	0.224 ± 0.018c	0.283 ± 0.005bc	0.288 ± 0.004b	0.290 ± 0.017b
L-Phenylalanine (Phe)	C_9_H_11_NO_2_	1.351 ± 0.089a	0.701 ± 0.048c	0.782 ± 0.014c	0.912 ± 0.054bc	1.128 ± 0.138ab
L-Tyrosine (Tyr)	C_9_H_11_NO_3_	10.171 ± 0.342d	15.483 ± 0.393c	16.624 ± 0.637c	18.773 ± 0.101b	20.906 ± 0.809a
L-α-Aspartyl-L-phenylalanine (α-AP)	C_13_H_16_N_2_O_5_	1.429 ± 0.027d	2.383 ± 0.029c	2.719 ± 0.149b	2.789 ± 0.029b	3.073 ± 0.109a
L-Leucine (Leu)	C_6_H_13_NO_2_	0.825 ± 0.111c	0.905 ± 0.042bc	1.055 ± 0.041b	1.418 ± 0.048a	1.585 ± 0.085a
L-Isoleucine (Ile)	C_6_H_13_NO_2_	4.994 ± 0.140a	2.567 ± 0.140d	2.830 ± 0.076d	3.741 ± 0.051c	4.087 ± 0.042b
L-Pipecolic acid	C_6_H_11_NO_2_	10.000 ± 0.424c	10.020 ± 0.066c	34.541 ± 6.155b	28.793 ± 3.203b	48.705 ± 3.427a
N-Acetylaspartate (NAA)	C_6_H_9_NO_5_	2.355 ± 0.141a	2.854 ± 0.662a	2.361 ± 0.619a	3.286 ± 0.263a	2.740 ± 0.081a
α-Aminoadipic acid	C_6_H_11_NO_4_	2.700 ± 0.513a	1.804 ± 0.101b	2.061 ± 0.154ab	2.725 ± 0.117a	2.476 ± 0.204ab
4-Acetamidobutyric acid	C_6_H_11_NO_3_	0.515 ± 0.010b	0.644 ± 0.028a	0.647 ± 0.013a	0.650 ± 0.003a	0.621 ± 0.002a
6-Aminocaproic acid (ACA)	C_6_H_13_NO_2_	ND	1.142 ± 0.015a	1.169 ± 0.018a	1.186 ± 0.021a	1.186 ± 0.018a
L-Tryptophan (Trp)	C_11_H_12_N_2_O_2_	80.878 ± 6.563a	7.412 ± 0.182b	8.142 ± 0.167b	9.741 ± 0.512b	11.346 ± 2.123b
Glycylphenylalanine	C_11_H_14_N_2_O_3_	ND	ND	ND	ND	ND
N′-Formylkynurenine	C_11_H_12_N_2_O_4_	15.418 ± 1.215d	24.929 ± 1.011c	26.456 ± 0.825bc	28.228 ± 0.787b	33.954 ± 0.631a
L-Valine (Val)	C_5_H_11_NO_2_	12.968 ± 0.884a	10.232 ± 0.526b	9.962 ± 0.350b	11.927 ± 0.231a	12.776 ± 0.183a
L-Methionine (Met)	C_5_H_11_NO_2_S	2.542 ± 0.102c	7.612 ± 0.635b	8.192 ± 0.358b	9.776 ± 0.452a	9.888 ± 0.206a
L-Proline (Pro)	C_5_H_9_NO_2_	645.889 ± 44.914c	2151.429 ± 78.846b	2267.012 ± 83.253ab	2425.094 ± 25.926a	2420.926 ± 50.214a
L-Glutamic acid (Glu)	C_5_H_9_NO_4_	2700.119 ± 91.072b	6639.120 ± 317.405a	6478.371 ± 174.902a	6279.665 ± 161.942a	6820.514 ± 96.150a
5-Hydroxy-tryptamine (5-HTP)	C_10_H_12_N_2_O	11.198 ± 0.910a	8.362 ± 0.462bc	10.089 ± 1.628ab	5.906 ± 0.266c	6.528 ± 0.381c
Trans-4-hydroxy-L-proline	C_5_H_9_NO_3_	2.865 ± 0.039d	8.615 ± 0.421c	14.151 ± 1.189b	12.566 ± 0.507b	17.954 ± 0.745a
Methionine sulfoxide (MetSO)	C_5_H_11_NO_3_S	1.079 ± 0.020d	1.953 ± 0.125c	2.477 ± 0.087b	2.416 ± 0.014b	2.927 ± 0.088a
5-Aminovaleric acid	NH_2_(CH_2_)_4_CO_2_H	0.529 ± 0.529b	1.799 ± 0.015a	1.865 ± 0.012a	1.810 ± 0.034a	1.825 ± 0.002a
L-Aspartate (Asp)	C_4_H_7_NO_4_	56.124 ± 1.650c	713.182 ± 56.401b	1043.476 ± 37.160a	1041.208 ± 55.672a	1161.492 ± 8.634a
L-Threonine (Thr)	C_4_H_9_NO_3_	66.646 ± 2.734d	111.219 ± 4.981c	112.264 ± 7.045c	156.143 ± 3.358b	189.988 ± 5.612a
Homoserine	C_4_H_9_NO_3_	2.073 ± 0.202b	4.841 ± 0.705a	5.168 ± 1.067a	5.788 ± 0.381a	4.102 ± 0.352a
N6-Acetyl-L-lysine	C_8_H_16_N_2_O_3_	0.522 ± 0.025c	0.653 ± 0.018b	0.831 ± 0.042a	0.879 ± 0.008a	0.869 ± 0.032a
(5-L-Glutamyl)-L-amino acid	C_8_H_14_N_2_O_5_S	ND	16.118 ± 0.631b	18.517 ± 0.816ab	20.437 ± 0.767a	19.994 ± 1.624a
N-Glycyl-L-leucine	C_8_H_16_N_2_O_3_	0.388 ± 0.052a	0.370 ± 0.006a	0.372 ± 0.009a	0.432 ± 0.012a	0.392 ± 0.024a
γ-Glutamate-cysteine	C_8_H_14_N_2_O_5_S	51.107 ± 1.171b	74.516 ± 2.935a	69.447 ± 7.705a	66.172 ± 4.770a	67.760 ± 1.597a
2-Aminobutyric acid	C_4_H_9_NO	0.418 ± 0.036c	0.657 ± 0.030b	0.740 ± 0.029ab	0.813 ± 0.042ab	0.918 ± 0.137a
(S)-β-Aminoisobutyric acid	C_4_H_9_NO	ND	ND	ND	ND	ND
γ-Aminobutyric acid (GABA)	C_4_H_9_NO	2695.942 ± 88.538b	4725.078 ± 206.671a	4886.078 ± 250.541a	5135.903 ± 64.712a	5100.838 ± 244.809a
N,N-Dimethylglycine (DMG)	C_4_H_9_NO_2_	ND	ND	ND	ND	ND
L-Cystathionine (LCYH)	C_7_H_14_N_2_O_4_S	0.608 ± 0.080c	1.235 ± 0.127b	1.637 ± 0.162a	1.662 ± 0.028a	1.684 ± 0.040a
Glycyl-L-proline (GLY-PRO)	C_7_H_12_N_2_O_3_	0.756 ± 0.014d	1.099 ± 0.030c	1.011 ± 0.027b	1.069 ± 0.025ab	0.980 ± 0.0170a
Nα-Acetyl-L-glutamine (NAG)	C_7_H_12_N_2_O_4_	0.132 ± 0.007e	0.997 ± 0.100d	1.963 ± 0.311c	2.966 ± 0.192b	4.193 ± 0.153a
Glutathione oxidized (GSSG)	C_20_H_32_N_6_O_12_S_2_	1854.060 ± 53.315d	7915.502 ± 682.289c	14,138.470 ± 703.316b	15,289.299 ± 1396.802b	18,558.053 ± 1028.213a
L-Alanine (Ala)	C_3_H_7_NO_2_	111.211 ± 4.295c	308.191 ± 13.137b	339.559 ± 8.060ab	366.460 ± 9.366a	368.487 ± 15.608a
L-Serine (Ser)	C_3_H_7_NO_3_	272.336 ± 20.443d	975.161 ± 39.892c	1159.738 ± 30.190b	1163.465 ± 10.628b	1339.013 ± 22.170a
L-Lysine (Lys)	C_6_H_14_N_2_O_2_	24.672 ± 1.606e	53.614 ± 1.669d	74.418 ± 6.176c	93.404 ± 1.124b	136.712 ± 7.862a
Beta-Alanine (β-Ala)	C_3_H_7_NO_2_	ND	12.461 ± 1.018b	17.243 ± 1.392a	16.776 ± 0.469a	18.423 ± 0.481a
Trimethylamine N-oxide (TMAO)	C_3_H_13_NO_3_	0.00612 ± 0.0006a	0.00348 ± 0.0003b	0.00265 ± 0.0001bc	0.00235 ± 0.0001c	0.00213 ± 0.00006c
N8-Acetylspermidine (N8AS)	C_9_H_21_N_3_O	2.476 ± 0.057a	2.340 ± 0.087ab	2.292 ± 0.017ab	2.216 ± 0.092bc	2.072 ± 0.066c
D-Alanyl-D-alanine (D-Ala-D-Ala)	C_6_H_12_N_2_O_3_	1.678 ± 0.076d	10.542 ± 0.310a	9.204 ± 0.099b	9.441 ± 0.157b	8.308 ± 0.110c
L-Cysteine (Cys)	C_3_H_7_NO_2_S	5.224 ± 0.854a	ND	ND	ND	ND
5-Hydroxylysine (Hyl)	C_6_H_14_N_2_O_3_	ND	ND	ND	ND	ND
L-Glutamine (Gln)	C_5_H_10_N_2_O_3_	353.734 ± 22.747e	1815.399 ± 89.840d	3624.851 ± 266.384c	4727.936 ± 162.809b	6030.362 ± 238.848a
L-Ornithine (Orn)	C_5_H_12_N_2_O_2_	8.172 ± 0.341d	14.600 ± 1.112d	36.474 ± 3.917c	59.289 ± 3.386b	100.013 ± 6.793a
Argininosuccinic acid (ASA)	C_10_H_20_N_4_O_6_	ND	ND	5.642 ± 2.824b	8.339 ± 0.421ab	9.846 ± 0.330a
L-Homocitrulline (HC)	C_7_H_15_N_3_O_3_	ND	ND	0.759 ± 0.003b	0.767 ± 0.007b	0.782 ± 0.007a
3-N-Methyl-L-histidine (3-N-Methyl-L-His)	C_7_H_11_N_3_O_2_	2.473 ± 0.039b	2.542 ± 0.089b	2.629 ± 0.107b	2.678 ± 0.038ab	2.853 ± 0.026a
S-(5-Adenosy)-L-homocysteine	C_14_H_20_N_6_O_5_S	1.717 ± 0.052b	4.225 ± 0.297a	4.153 ± 0.051a	4.498 ± 0.146a	4.459 ± 0.089a
L-Carnosine	C_9_H_14_N_4_O_3_	1.894 ± 0.025ab	1.882 ± 0.022ab	1.841 ± 0.051ab	1.926 ± 0.031a	1.789 ± 0.034b
L-Asparagine anhydrous (Asn)	C_4_H_8_N_2_O_3_	13.734 ± 0.973d	1003.304 ± 128.749c	3083.540 ± 397.933b	3376.453 ± 160.504b	4580.323 ± 136.984a
L-Citrulline (Cit)	C_6_H_13_N_3_O_3_	3.149 ± 0.163e	43.396 ± 3.843d	163.437 ± 20.155c	216.965 ± 14.841b	319.212 ± 13.408a
Glycine (Gly)	C_2_H_5_NO_2_	6.946 ± 1.082d	44.294 ± 3.368c	123.118 ± 5.959b	125.389 ± 5.278b	168.703 ± 3.520a
Nα-Acetyl-L-arginine	C_8_H_16_N_4_O_3_	10.478 ± 0.18b	19.97 ± 1.10a	22.48 ± 0.98a	21.84 ± 0.68a	23.36 ± 2.90a
2-Aminoethanesulfonic acid	C_2_H_7_NO_3_S	1.673 ± 0.007 cd	1.654 ± 0.011d	1.709 ± 0.008ab	1.685 ± 0.002bc	1.735 ± 0.011a
Ethanolamine (EtA)	C_2_H_7_NO	20.179 ± 3.581c	71.230 ± 0.489a	60.926 ± 1.657b	65.651 ± 2.804ab	61.441 ± 2.380b
Homo-L-arginine (homo-Arg)	C_7_H_16_N_4_O_2_	0.993 ± 0.126d	2.530 ± 0.170c	3.010 ± 0.176bc	3.415 ± 0.325b	4.152 ± 0.031a
3,7-Dimethyluric acid	C_7_H_8_N_4_O_3_	0.187 ± 0.001c	0.197 ± 0.002c	0.225 ± 0.021bc	0.303 ± 0.014a	0.262 ± 0.034ab
L-Arginine (Arg)	C_6_H_14_N_4_O_2_	8.876 ± 0.888e	231.915 ± 16.001d	500.928 ± 55.152c	773.747 ± 19.609b	912.786 ± 37.763a
Creatine-phosphate (CP)	C_4_H_8_N_3_Na_2_O_5_P	380.690 ± 12.393a	188.519 ± 17.603bc	247.029 ± 26.919b	218.475 ± 13.702bc	156.963 ± 42.067c
Creatine	C_4_H_9_N_3_O_2_	0.329 ± 0.006a	0.136 ± 0.136ab	ND	ND	0.122 ± 0.122ab
Succinic acid	C_4_H_6_O_4_	715.448 ± 23.935a	706.139 ± 78.683a	574.498 ± 24.891b	532.770 ± 14.402b	454.466 ± 7.096b
Summation of FAADs (TFAADs)		10,185.614 ± 352.688d	27,980.176 ± 1172.927c	39,231.767 ± 1400.485b	42,368.673 ± 1244.381b	49,243.660 ± 1676.003a

*ND* Not detected. Different letters within a row indicate a significant difference at *P* < 0.05. FAADs in the table were arranged from high to low according to their C/N ratio. The same notation will be used in Table 2

**Table 2:** Effects of N supply on mean (±SE, *n* = 3) concentrations (μg g^−1^ DW) of FAADs as well as their summation in *Citrus sinensis* roots

FAADs	Molecular formula	N treatments (mM)				
		0	5	10	15	20
NAT	C_11_H_13_NO_4_	0.165±0.001b	0.340±0.022a	0.323±0.017a	0.408±0.035a	0.403±0.051a
H-Tyr-OMe	C_10_H_14_NO_3_	0.199±0.016a	0.080±0.005c	0.122±0.026bc	0.148±0.025ab	0.147±0.017ab
KYNA	C_10_H_7_NO_3_	0.233±0.010a	0.291±0.030a	0.269±0.016a	0.273±0.024a	0.231±0.015a
Phe	C_9_H_11_NO_2_	2.154±0.065b	2.291±0.189b	3.145±0.323a	3.633±0.318a	3.782±0.268a
Tyr	C_9_H_11_NO_3_	31.199±1.428b	73.209±4.578a	83.615±3.571a	78.660±6.685a	70.230±6.440a
α-AP	C_13_H_16_N_2_O_5_	1.566±0.040b	2.192±0.084a	2.443±0.094a	2.540±0.155a	2.221±0.192a
Leu	C_6_H_13_NO_2_	5.354±0.425b	6.832±0.324a	7.747±0.531a	7.650±0.540a	6.968±0.405a
Ile	C_6_H_13_NO_2_	8.767±0.782c	17.868±0.948ab	17.952±1.152a	16.048±1.230ab	14.522±1.160b
L-Pipecolic acid	C_6_H_11_NO_2_	29.837±5.389c	95.538±12.080b	154.734±9.567a	189.444±8.235a	206.392±34.005a
NAA	C_6_H_9_NO_5_	0.284±0.048c	1.352±0.090bc	1.762±0.420ab	2.675±0.327a	2.347±0.651ab
α-Aminoadipic acid	C_6_H_11_NO_4_	73.208±4.056a	25.016±3.301b	18.591±1.362bc	14.702±0.925c	14.432±1.486c
4-Acetamidobutyric acid	C_6_H_11_NO_3_	0.688±0.044a	0.701±0.009a	0.653±0.046a	0.645±0.038a	0.597±0.048a
ACA	C_6_H_13_NO_2_	1.236±0.018c	1.923±0.033a	1.626±0.060b	1.600±0.068b	1.501±0.056b
Trp	C_11_H_12_N_2_O_2_	71.085±5.983a	71.149±3.780a	66.691±3.888a	71.457±3.302a	67.582±5.361a
Glycylphenylalanine	C_11_H_14_N_2_O_3_	0.409±0.024c	0.531±0.009a	0.547±0.008a	0.547±0.018a	0.463±0.014b
N'-Formylkynurenine	C_11_H_12_N_2_O_4_	4.974±0.554c	7.231±0.074b	8.085±0.116ab	9.054±0.503a	9.036±0.643a
Val	C_5_H_11_NO_2_	18.698±1.565c	27.613±1.391a	24.994±0.985ab	23.315±1.307b	22.661±1.051bc
Met	C_5_H_11_NO_2_S	3.743±0.085c	7.003±0.195a	7.059±0.337a	5.983±0.452b	6.210±0.184ab
Pro	C_5_H_9_NO_2_	374.028±22.621c	1492.480±27.103b	1928.221±122.469a	2048.379±127.600a	1980.211±6.484a
Glu	C_5_H_9_NO_4_	4882.527±168.286b	6279.027±253.890a	6550.942±263.117a	6795.105±94.542a	6721.240±73.946a
5-HTP	C_10_H_12_N_2_O	36.506±1.836a	26.402±2.582c	27.071±0.638bc	31.948±2.034abc	33.912±3.297ab
Trans-4-Hydroxy-L-proline	C_5_H_9_NO_3_	3.819±0.585c	37.349±0.798b	49.452±3.473a	51.573±4.419a	48.613±5.078a
MetSO	C_5_H_11_NO_3_S	2.174±0.054c	4.281±0.304a	4.090±0.078a	3.867±0.198ab	3.474±0.174b
5-Aminovaleric acid	NH_2_(CH_2_)_4_CO_2_H	1.944±0.079c	2.197±0.018bc	2.483±0.106ab	2.651±0.142a	2.596±0.249ab
Asp	C_4_H_7_NO_4_	421.848±45.155c	825.214±30.502a	716.624±44.105b	876.584±14.487a	897.974±23.794a
Thr	C_4_H_9_NO_3_	59.546±1.176c	117.639±8.039b	142.318±7.715a	139.047±8.561ab	132.957±9.415ab
Homoserine	C_4_H_9_NO_3_	1.742±0.043b	6.916±1.022a	7.793±1.188a	8.486±0.119a	6.889±2.227a
N6-Acetyl-L-lysine	C_8_H_16_N_2_O_3_	1.641±0.038b	3.397±0.230a	3.171±0.365a	3.038±0.316a	2.777±0.434a
(5-L-Glutamyl)-L-amino acid	C_8_H_14_N_2_O_5_S	7.225±0.319c	24.528±1.782ab	29.969±2.840a	25.353±1.698ab	22.180±2.472b
N-Glycyl-L-leucine	C_8_H_16_N_2_O_3_	1.056±0.082c	1.389±0.105b	1.697±0.032a	1.770±0.113a	1.339±0.065b
γ-Glutamate-cysteine	C_8_H_14_N_2_O_5_S	ND	72.421±6.540a	65.085±9.579a	46.665±19.215a	62.097±13.921a
2-Aminobutyric acid	C_4_H_9_NO	0.605±0.035b	1.107±0.133a	1.014±0.124a	1.042±0.168a	0.937±0.133ab
(S)-β-Aminoisobutyric acid	C_4_H_9_NO	1.799±0.134b	3.215±0.202a	3.536±0.477a	3.578±0.426a	3.343±0.496a
GABA	C_4_H_9_NO	885.850±23.997b	3313.895±27.368a	3594.377±395.769a	3791.500±258.905a	3257.049±294.992a
DMG	C_4_H_9_NO_2_	0.152±0.076b	0.430±0.036a	0.426±0.069a	0.431±0.035a	0.382±0.036a
LCYH	C_7_H_14_N_2_O_4_S	19.039±2.350a	5.215±0.392b	7.133±0.294b	8.231±1.228b	8.069±0.584b
GLY-PRO	C_7_H_12_N_2_O_3_	0.745±0.005d	1.280±0.050c	1.762±0.114ab	1.973±0.110a	1.596±0.070b
NAG	C_7_H_12_N_2_O_4_	1.657±0.104b	2.077±0.186b	4.511±0.529a	4.525±0.808a	5.491±0.589a
GSSG	C_20_H_32_N_6_O_12_S_2_	8291.753±901.041c	28363.315±2223.965b	39540.366±2348.517a	44467.728±7029.281a	41518.728±1469.166a
Ala	C_3_H_7_NO_2_	169.976±7.362c	414.387±32.296b	589.784±32.025a	508.838±40.846ab	437.666±59.094b
Ser	C_3_H_7_NO_3_	92.352±1.495c	306.843±13.234b	382.944±32.603a	375.183±20.996ab	365.122±27.631ab
Lys	C_6_H_14_N_2_O_2_	54.745±3.990d	272.506±14.334c	443.637±45.060b	439.929±47.889ab	447.527±26.121a
β-Ala	C_3_H_7_NO_2_	44.684±3.817b	321.239±13.730a	429.385±53.519a	419.892±45.015a	383.111±59.216a
TMAO	C_3_H_13_NO_3_	0.00919±0.00018a	0.00590±0.00038b	0.00276±0.00017c	0.00273±0.00006c	0.00256±0.00002c
N8AS	C_9_H_21_N_3_O	2.745±0.162b	4.203±0.250a	4.331±0.010a	4.671±0.203a	4.472±0.176a
D-Ala-D-Ala	C_6_H_12_N_2_O_3_	11.662±0.236c	26.255±0.886a	22.260±2.114a	17.498±1.604b	13.289±0.954c
Cys	C_3_H_7_NO_2_S	ND	2.185±1.148a	3.799±0.230a	ND	ND
Hyl	C_6_H_14_N_2_O_3_	ND	3.752±0.021b	4.376±0.088a	4.708±0.216a	4.688±0.222a
Gln	C_5_H_10_N_2_O_3_	1660.744±97.980c	2470.030±94.279c	3775.858±77.545b	4729.450±445.431a	5471.375±327.451a
Orn	C_5_H_12_N_2_O_2_	20.165±1.102d	62.994±4.976c	162.107±19.792b	177.968±30.235ab	210.096±16.232a
ASA	C_10_H_20_N_4_O_6_	38.332±0.776a	9.740±0.267c	13.256±1.147b	14.199±0.706b	13.821±0.493b
HC	C_7_H_15_N_3_O_3_	0.734±0.011c	0.822±0.015b	0.926±0.044a	0.869±0.012ab	0.880±0.030ab
3-N-Methyl-L-His	C_7_H_11_N_3_O_2_	4.067±0.184a	3.805±0.259a	3.697±0.237a	3.717±0.130a	3.641±0.196a
S-(5-Adenosy)-L-homocysteine	C_14_H_20_N_6_O_5_S	6.063±0.054b	16.725±0.436a	15.887±0.955a	15.472±0.872a	15.785±1.463a
L-Carnosine	C_9_H_14_N_4_O_3_	2.018±0.028c	2.787±0.170b	3.324±0.101a	3.245±0.104a	2.798±0.041b
Asn	C_4_H_8_N_2_O_3_	793.707±45.995d	3076.171±270.464c	5167.251±144.278b	5887.175±388.061ab	6316.118±145.607a
Cit	C_6_H_13_N_3_O_3_	18.431±2.632d	107.488±3.100c	210.235±17.070b	234.994±31.482ab	275.774±6.540a
Gly	C_2_H_5_NO_2_	13.987±1.395c	59.992±1.186b	84.196±8.524a	88.209±5.087a	88.209±5.087a
Nα-Acetyl-L-arginine	C_8_H_16_N_4_O_3_	5.953±0.588a	5.235±0.228a	5.813±0.325a	5.364±0.261a	4.900±0.776a
2-Aminoethanesulfonic acid	C_2_H_7_NO_3_S	1.804±0.031b	1.804±0.031b	3.559±0.434a	3.647±0.361a	3.616±0.240a
EtA	C_2_H_7_NO	34.798±2.553c	85.061±5.648a	81.762±2.640a	69.332±12.292ab	61.569±2.705b
Homo-Arg	C_7_H_16_N_4_O_2_	9.273±0.102d	11.952±0.515c	15.939±1.399b	16.224±0.356ab	17.929±1.918a
3,7-Dimethyluric acid	C_7_H_8_N_4_O_3_	ND	ND	ND	ND	ND
Arg	C_6_H_14_N_4_O_2_	291.375±14.853c	474.445±8.089b	906.772±30.635a	967.224±88.045a	1054.664±63.417a
CP	C_4_H_8_N_3_Na_2_O_5_P	73.734±5.552b	146.347±31.718a	123.320±18.966ab	164.386±10.906a	155.567±8.084a
Creatine	C_4_H_9_N_3_O_2_	0.104±0.104a	0.163±0.163a	0.154±0.154a	0.370±0.013a	0.375±0.020a
Succinic acid	C_4_H_6_O_4_	691.983±50.826c	1770.347±76.789a	1542.250±34.615ab	1570.586±90.656ab	1394.339±134.070b
TFAADs		19292.898±1188.472c	50582.832±3001.737b	67070.472±3239.715a	74465.409±7887.679a	71882.835±1558.005a

Also, we calculated the relative amount of individual FAA or derivative (as a percentage of the TFAADs content). In leaves, N-deficiency decreased the relative amount of 15 FAADs [Gly, Asp, Asn, Gln, Arg, Cit, HC, β-Ala, trans-4-hydroxy-L-proline, Orn, (5-L-glutamyl)-L-amino acid, GSSG, NAG, ASA and ACA] and increased the relative amount of 42 FAADs relative to 20 mM N, but had no significant impact on the relative amount of 6 FAADs (Ser, Lys, L-pipecolic acid, D-Ala-D-Ala, homo-L-Arg and 5-aminovaleric acid) (Table S1 and Fig. S3a). In roots, N-deficiency reduced the relative amount of 12 FAADs (Gly, Pro, Asn, Lys, Cit, β-Ala, L-pipecolic acid, trans-4-hydroxy-L-proline, Orn, NAA, γ-glutamate-cysteine and Hyl) and improved the relative amount of 43 FAADs relative to 20 mM N, but had no significant influence on the relative amount of 11 FAADs [Ser, Gln, Arg, homoserine, (5-L-glutamyl)-L-amino acid, GSSG, NAG, GABA, Cys, creatine and DMG] (Table S2 and Fig. S3b).

**Fig. 3:**
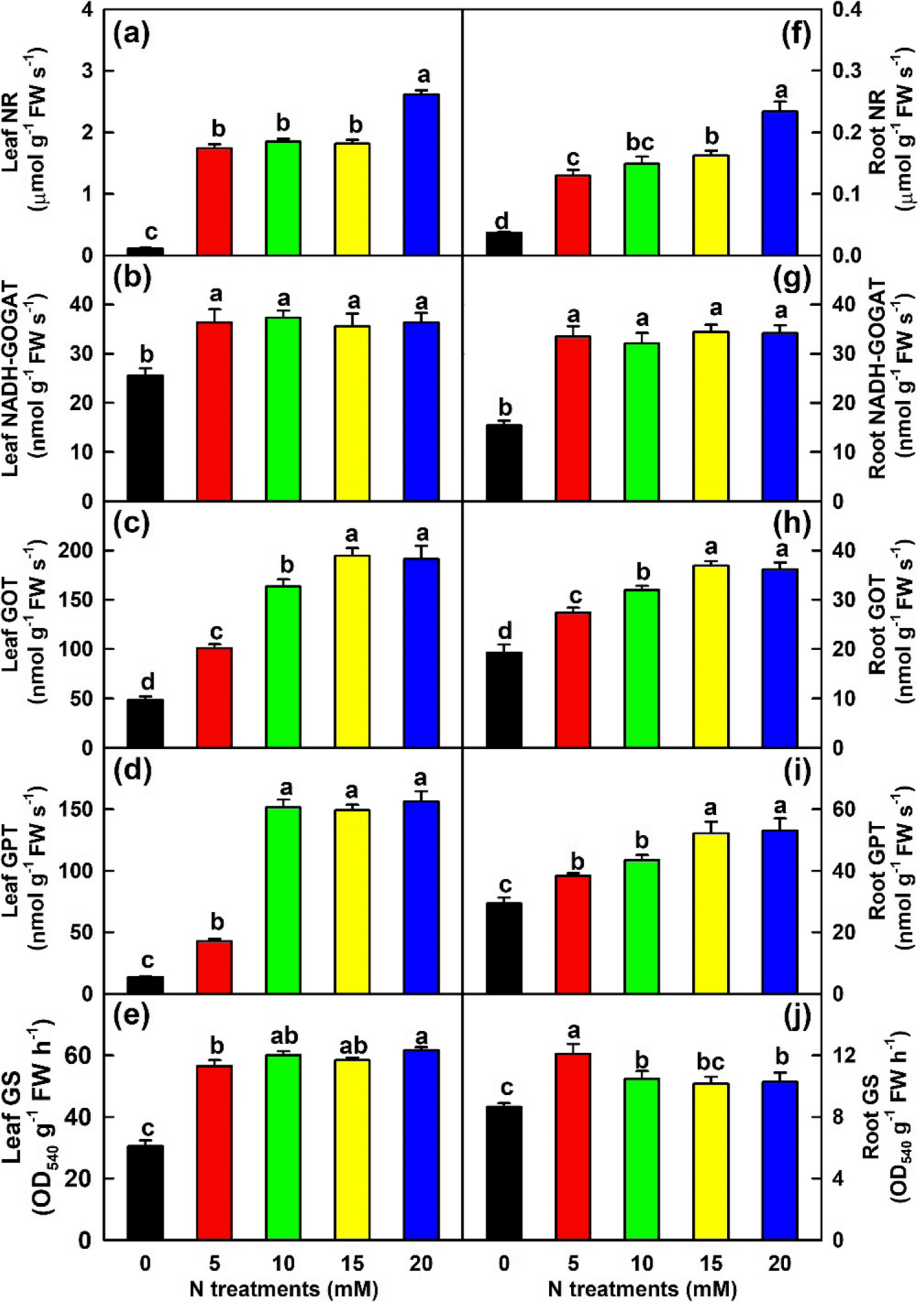
Effects of N supply on mean (± SE, *n* = 4) activities of N assimilation-related enzymes in leaves and roots. **a** and **f** Leaf and root nitrate reductase (NR). **b** and **g** Leaf and root NADH-dependent glutamate 2-oxoglutarate aminotransferase (NADH-GOGAT). **c** and **h** Leaf and root glutamate oxaloacetate aminotransferase (GOT). **d** and **i** Leaf and root glutamate pyruvate aminotransferase (GPT). **e** and **j** Leaf and root glutamine synthase (GS)

As shown in Fig. 3, the activities of NR, GOT and GPT in leaves and roots increased with the increase of N supply, while the activities of NADH-GOGAT and GS in leaves and roots increased as N supply increased from 0 to 5 mM, then kept relative stable with the further increase of N supply. Compared with 20 mM N, N-deficiency led to reduced activities of NR, NADH-GOGAT, GOT, GPT and GS by 95.7, 29.6, 74.6, 91.3 and 50.5%, respectively in leaves, and 84.2, 54.9, 46.7, 44.7 and 15.7%, respectively in roots.

### Effects of N supply on nonstructural carbohydrates, OAs and acid-metabolism-related in leaves and roots

**Fig. 4:**
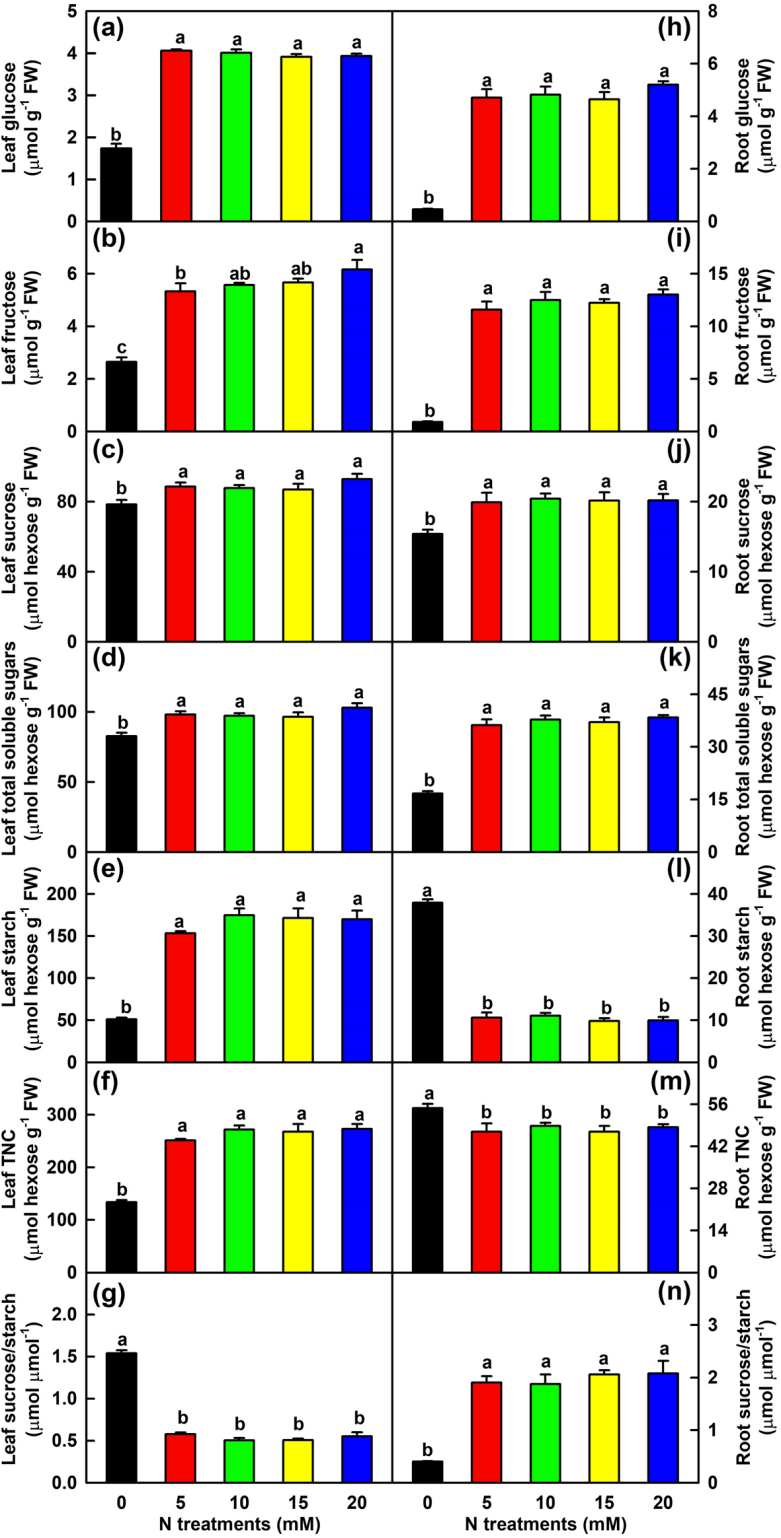
Effects of N supply on mean (± SE, *n* = 4) concentrations of nonstructural carbohydrates and ratios of sucrose/starch in leaves and roots. **a** and **h** Leaf and root glucose. **b** and **i** Leaf and root fructose. **c** and **j** Leaf and root sucrose. **d** and **k** Leaf and root total soluble sugars (summation of glucose + fructose + sucrose). **e** and **l** Leaf and root starch. **f** and **m** Leaf and root total nonstructural carbohydrate (TNC, summation of starch + total soluble sugars. **g** and **n** Leaf and root ratios of sucrose/starch

In leaves, the concentrations of glucose, fructose, sucrose, total soluble sugars, starch and TNC were lower at 0 mM N than at 5–20 mM N, but the reverse was the case for sucrose/starch ratio. In roots, the concentrations of glucose, fructose, sucrose and total soluble sugars as well as sucrose/starch ratio were lower at 0 mM N than at 5–20 mM N, but the opposite was true for the concentrations of starch and TNC (Fig. 4). TNC/C ratio in leaves decreased in response to N-deficiency because N-deficiency affected TNC concentration more than C concentration, while the ratio in roots increased in response to N-deficiency because N-deficiency increased and decreased TNC and C concentrations, respectively (Figs. 1 and 4).

**Fig. 5:**
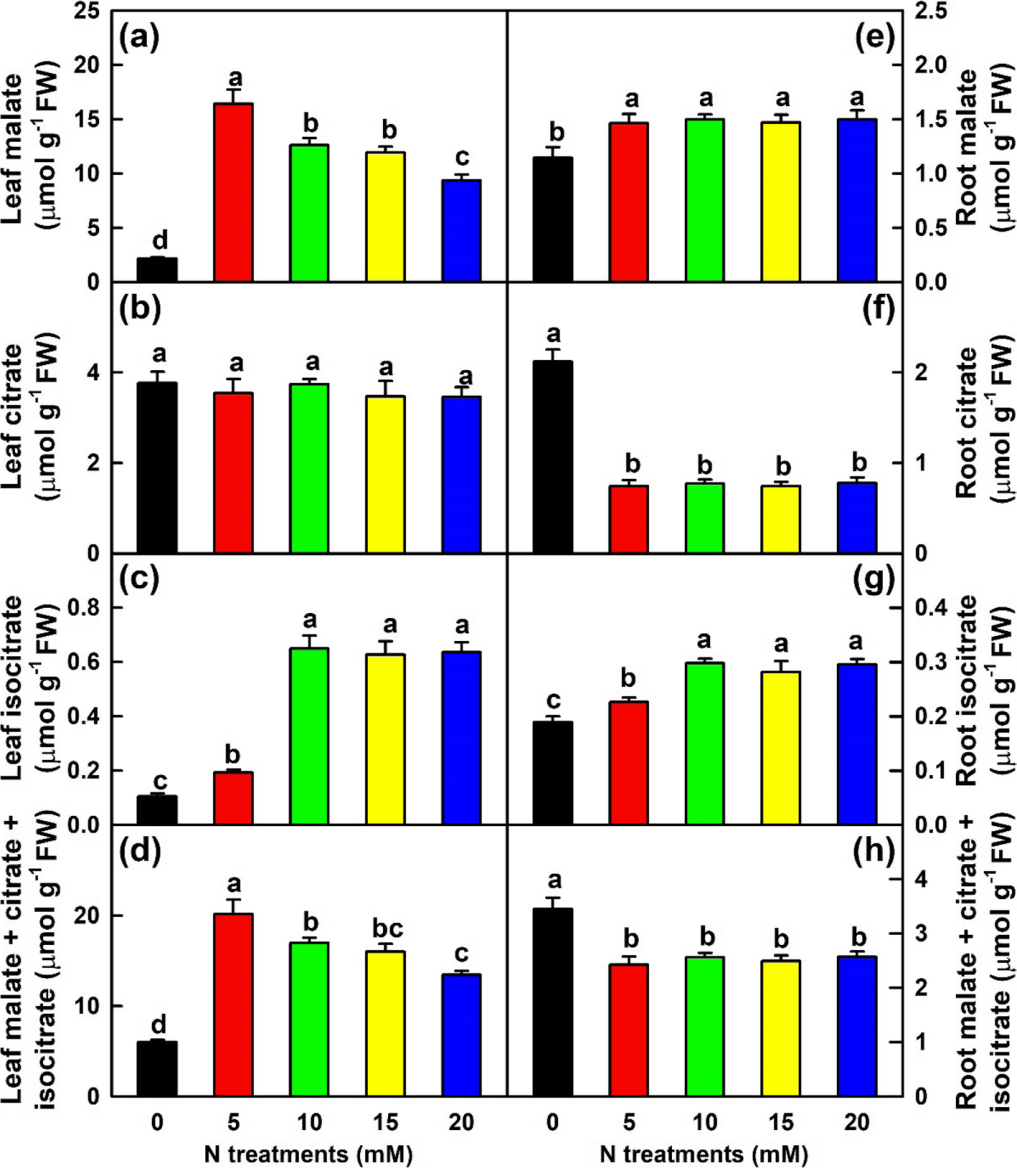
Effects of N supply on mean (± SE, *n* = 4) concentrations of organic acids in leaves and roots. **a** and **e** Leaf and root malate. **b** and **f** Leaf and root citrate. **c** and **g** Leaf and root isocitrate. **d** and **h** Leaf and root malate + citrate + isocitrate

In leaves, the concentrations of malate and malate + citrate + isocitrate increased as N supply increased from 0 to 5 mM, then decreased with further increase of N supply. The concentration of isocitrate increased as N supply increased from 0 to 10 mM, then kept unchanged with the further increase of N supply. N supply had no significant impact on citrate concentration. In roots, the concentration of isocitrate increased with the increase of N supply from 0 to 10 mM, then remained unchanged with the increase of N supply. The concentration of malate was lower at 0 mM N than at 5–20 mM N, while the opposite was true for the concentrations of citrate and malate + citrate + isocitrate (Fig. 5).

**Fig. 6:**
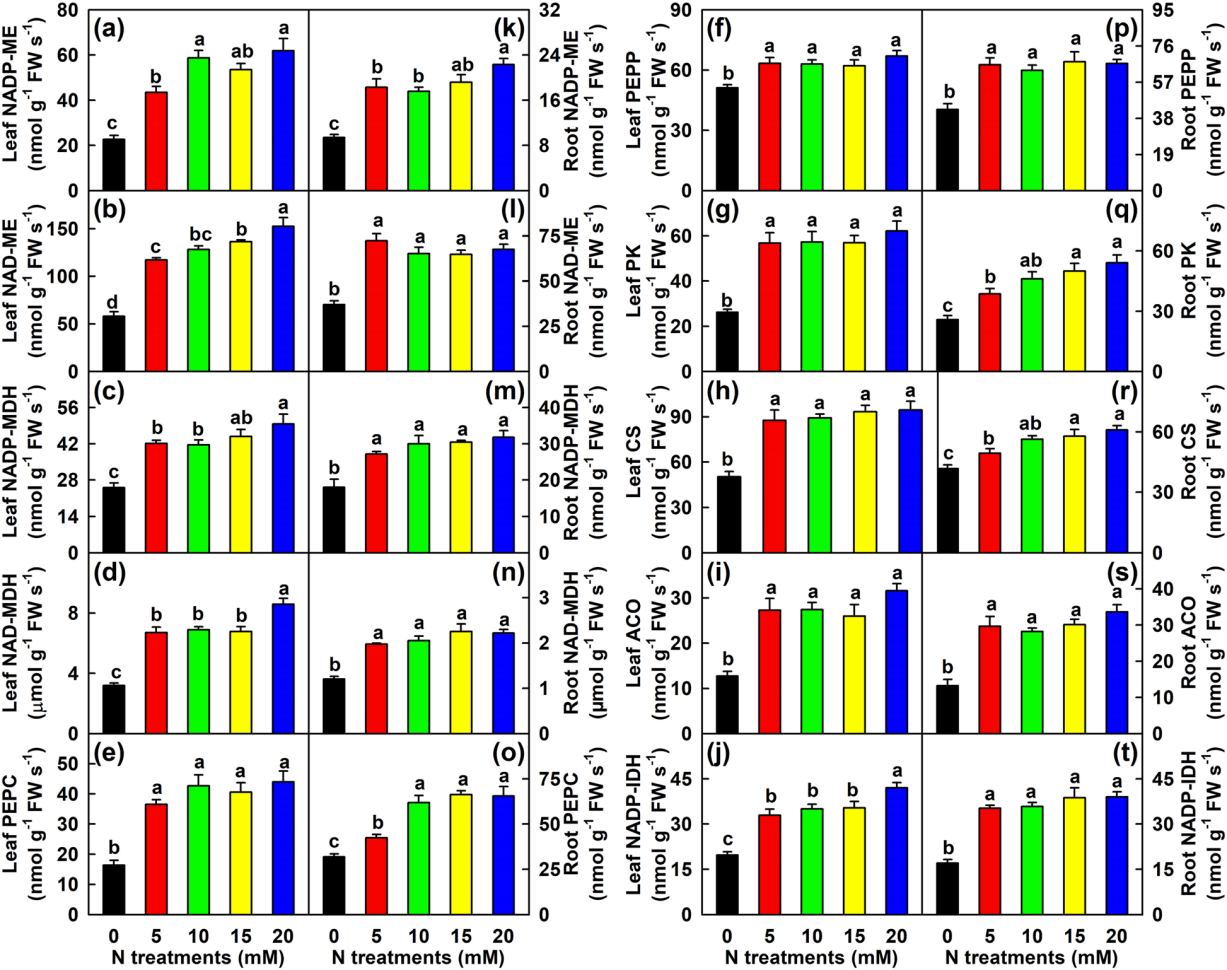
Effects of N supply on mean (± SE, *n* = 4) activities of acid-metabolizing enzymes in leaves and roots. **a** and **k** Leaf and root NADP-malic enzyme (NADP-ME). **b** and **l** Leaf and root NAD-ME. **c** and **m** Leaf and root NADP-malate dehydrogenase (NADP-MDH). **d** and **n** Leaf and root NAD-MDH. **e** and **o** Leaf and root phosphoenolpyruvate carboxylase (PEPC). **f** and **p** Leaf and root phosphoenolpyruvate phosphatase (PEPP). **g** and **q** Leaf and root pyruvate kinase (PK). **h** and **r** Leaf and root citrate synthase (CS). **i** and **s** Leaf and root aconitase (ACO). **j** and **t** Leaf and root NADP-isocitrate dehydrogenase (NADP-IDH)

Compared with 20 mM N, N-deficiency led to reduced activities of NADP-ME, NAD-ME, NADP-MDH, NAD-MDH, PEPC, PEPP, PK, CS, ACO and NADP-IDH by 63.4, 61.9, 49.5, 62.7, 62.8, 23.7, 57.9, 46.9, 59.7 and 53.0%, respectively in leaves, and 57.8, 45.1, 43.4, 45.6, 51.4, 36.3, 52.3, 31.5, 60.6 and 56.4%, respectively in roots (Fig. 6).

### Principal component analysis (PCA) loading plots

Using PCA, we investigated the response patterns of 105 and 102 physiological parameters in roots and leaves, responsively to N-deficiency (Fig. S4). PC1 and PC2 contributed 67.81 and 11.22%, and 69.22 and 11.64% of the total variation for roots and leaves, respectively. For roots, trans-4-hydroxy-L-proline (0.989), Ser (0.987), C/N (− 0.984), Hyl (0.983), Pro (0.982), Thr (0.973), Gly (0.972), TFAADs/C (0.972), N (0.964) and α-aminoadipic acid (− 0.963) were the main contributors of PC1 (Table S3). For leaves, PCI greatly depended on Ser (0.994), C/N (− 0.991), Asp (0.986), N (0.984), Asp-Phe (0.982), Ala (0.981), TFAADs/C (0.978), Met (0.977), β-Ala (0.976) and Pro (0.976) (Table S4).

## Discussion

### Nitrogen-deficiency increased the partitioning of C and N to roots and C/N ratios in leaves, stems and roots

An increase in R/S is an adaptive strategy to N-deficiency, since relatively more roots feed relatively less shoots with N, so plants can accumulate more N in shoots [41, 42]. We found that N-deficiency increased the partitioning of C and N to roots (Fig. 1), as obtained on tomato [43], maize (*Zea mays* cv. Green) [44], *Populus cathayana* [4], tall fescue [11], *Eucalyptus camaldulensis* and *Eucalyptus tereticornis* [38]. These results suggested that both N and C was preferentially allocated to the roots of N-deficient plants to maintain their growth, thus increasing R/S.

Our findings that N-deficiency reduced C and N concentrations and increased C/N ratio in leaves, stems and roots, with a greater increase of C/N ratio in leaves (93.2%) and stems (214.8%) than in roots (80.5%) (Fig. 1) agreed with the report that N-deficiency reduced N concentration and increased C/N ratio in maize axile roots, lateral roots and shoots, with a stronger increment of C/N ratio in shoots than in axile and lateral roots [41]. An increase of C/N ratio indicates that the acquisition of N and C is unbalanced, resulting in an apparent N-deficiency, which leads to sink limitation [45]. Alternatively, an increment of C/N ratio is regarded as an apparent increase in plant NUE [46]. The increased ratios of R/S [3] and C/N (Fig. 1) in N-deficient *C. sinensis* roots suggested that higher NUE contributed to N optimization [17, 46]. Roots provide shoots with the mineral nutrients they require and shoots provide roots with carbohydrates. Deficiencies of mineral nutrients and carbohydrates may be the limiting factors for shoot and root growth, respectively. A decreasing supply of mineral nutrients usually leads to an increased R/S due to increased root growth relative to shoot growth [3, 14, 41, 47]. The distribution of biomass is related to C/N ratio in plants [48]. Saarinen [47] indicated that a high ratio of TNC/FAA (a better indicator of the internal C to N balance) might increase the allocation of biomass to roots. Liu et al. [34] reported that low-N led to increased C/N ratio in soybean roots, and the increment was stronger in low-N-tolerant wild soybean than in common wild soybean. Therefore, N-deficiency-induced an increment in C/N ratio might be an adaptive response to N-deficiency by increasing R/S and NUE of *C. sinensis* seedlings.

### Nitrogen-deficiency decreased the accumulation of TNC and malate + citrate + isocitrate and increased sucrose/starch ratio in leaves, but the reverse was the case in roots

Because the partitioning of C and N to roots was elevated by N-deficiency, the N-deficiency-induced alterations of C and N metabolisms should be some different between roots and leaves. PCA indicated that the difference in the cumulative contribution of PC1 and PC2 to total variation between leaves and roots was very small, and most of these parameters was highly clustered in the right; but the 10 acid metabolizing enzymes in leaves were more highly clustered than in roots (Fig. S4). In addition, the principal components of leaves and roots were different. For example, the first five main contributors of PC1 to total variation for roots and leaves were trans-4-hydroxy-L-proline, Ser, C/N, Hyl and Pro, and Ser, C/N, Asp, N and Asp-Phe, respectively (Tables S3-S4). Regressive analysis indicated that 54 and 8 (C distribution in leaves, N distribution in leaves, lle, creatine-phosphate, starch, TNC, sucrose/starch and malate + citrate + isocitrate) leaf physiological parameters displayed positive and negative relation with the corresponding root parameters, respectively (Table S5 and Fig. S5). Obviously, some differences existed between roots and leaves in N-deficiency-induced alterations of C and N metabolisms.

Further analysis indicated that in leaves, N-deficiency lowered the concentrations of glucose, fructose, sucrose, total soluble sugars, starch, TNC, malate, isocitrate and malate + citrate + isocitrate, increased the ratios of sucrose/starch and sucrose/TNC, and had no significant impact on citrate concentration. In addition, N-deficiency decreased the distribution of C in leaves and stems (Figs. 1 and 4-5). Because sucrose is the major translocation form of assimilated C in *Citrus*, N-deficiency-induced an increase of sucrose/starch ratio in leaves and a decrease of C distributions in leaves and stems implied that the partitioning trend of assimilated C had shifted to sucrose, thus increasing sucrose export to roots and improving R/S [3]. N-deficiency-induced decreases of TNC and starch concentrations and increase of sucrose/starch ratio have also been obtained in sunflower leaves [49]. Schlüter et al. [8] observed that the levels of many TCA cycle intermediates and sugars were reduced in 30-day low-N (0.15 mM) treated maize leaves, and that low-N did not lead to a large accumulation of starch, especially under long-term N-deficiency. Instead, parts of the assimilated C were shifted to the biosynthesis of raffinose and related sugars, into the cell wall and some secondary products. However, a 7-day N-deficient treatment led to increased accumulation of sugars and TCA cycle intermediates in barley leaves [12]. Boussadia et al. [23] showed that a 58-day N-deficient treatment significantly increased the levels of starch, mannitol, glucose and fructose in ‘Koroneiki’ olive leaves and starch in ‘Meski’ olive leaves, but had no significant impact on the levels of sucrose in ‘Koroneiki’ leaves and mannitol, glucose, fructose and sucrose in ‘Meski’ leaves. In apple leaves, a 35-day low-N (0.3 mM) treatment reduced the levels of sucrose and sorbitol, increased the level of glucose, but had no significant influence on the levels of starch and fructose [39]. Obviously, N-deficient effects on nonstructural carbohydrates in leaves depend on the degree of N-deficiency, duration of exposure to N-deficiency, and plant species or cultivar. In addition to reduced accumulation of OAs, N-deficiency reduced the activities of 10 acid-metabolizing enzymes in leaves (Fig. 6), implying that N-deficiency might downregulate OA metabolism in leaves, as obtained in N-deficient tobacco leaves [35]. OAs are the preferred source of plant C under nutrient-limited conditions and can act as C precursors for AA biosynthesis [8, 28]. N-deficiency-induced a reduction in the OA pool agrees with the decreased demand for these C skeleton precursors due to reduced biosynthesis of AAs (Table 1) [8].

Unlike to leaves, N-deficiency reduced the concentrations of glucose, fructose, sucrose, total soluble sugars, malate and isocitrate and the ratio of sucrose/starch, and increased the concentrations of starch, TNC, citrate and malate + citrate + isocitrate in roots (Figs. 4-5). This implied that the partitioning trend of assimilated C had shifted to starch in N-deficient *C. sinensis* roots, as obtained in N-deficient tobacco roots [35], because N-deficiency increased starch/TNC ratio by 236.7% and decreased sucrose/starch ratio by 80.5% in roots relative to 20 mM N (Fig. 4). N-deficiency-induced increases of TNC and malate + citrate + isocitrate agrees with the increased distribution of C in N-deficient roots (Fig. 1). Under N-starved conditions, the decreased demand for C skeletons in N assimilation improve C storage forms as starch [4, 30]. Thus, N-deficiency-induced accumulation of TNC and starch in roots could be explained in this way, as indicated by the reduced TFAADs/C ratio (Fig. S2). N-deficiency-induced accumulation of TNC and starch have also been obtained in roots of *P. cathayana* [4] and tobacco [18, 35]. However, N-deficiency enhanced the transport of sorbitol and sucrose to roots as well as the accumulation of sorbitol and sucrose in roots, but largely reduced the accumulation of starch in roots of apple [39]. This might be related to the fact that in apple, sorbitol is the primary photosynthetic and phloem-translocated carbohydrate in source leaves and the level of sorbitol in roots is much higher than that of starch. Further studies are needed to answer the question. Our results indicated that the activities of all the 10 acid-metabolizing enzymes in roots reduced in response to N-deficiency (Fig. 6), implying that OA metabolism might be downregulated in N-deficient *Citrus* roots, as obtained in N-starved tobacco roots [35]. However, N-deficiency caused an increase in the concentrations of citrate and malate + citrate + isocitrate in roots (Fig. 5). The increment might be caused by decreased utilization due to reduced biosynthesis of AAs, as indicated by reduced concentrations of most FAADs and TFAADs (Table 2) and ratio of TFAADs/C (Fig. S2) in roots. Our results showed that the concentration of citrate was significantly and negatively related to ACO (*r* = − 0.9658) or NADP-IDH (*r* = − 0.9817) activity, and displayed a decreased trend with the increase of PEPC (*r* = 0.7736) or CS (*r* = 0.8256) activity in roots (Table S6). Thus, N-deficiency-induced an accumulation of citrate in roots was caused by decreased utilization (catabolism), rather than by increased biosynthesis.

### Nitrogen-deficiency decreased N uptake and N concentrations in leaves, stems and roots, as well as N assimilation and AA biosynthesis in leaves and roots, but increased NH_4_^+^-N/NO_3_^−^-N ratio in leaves and roots

Our findings that N-deficiency reduced N content per plant (N uptake), the levels of N in leaves, stems and roots, as well as the levels of TSPs, TFAADs, NH_4_^+^-N and NO_3_^−^-N and the activities of N-assimilation related enzymes in leaves and roots (Figs. 1-3 and Tables 1-2) indicated that N-deficiency suppressed N uptake and assimilation during acclimation to N-starvation. N-deficiency-induced an increment in leaf NH_4_^+^-N concentration was not caused by increased reduction of NO_3_^−^, because NR activity was decreased in these leaves (Fig. 1) and NH_4_^+^-N concentration decreased with the increase of NR activity in leaves (*r* = − 0.882; Table S7). The increment might be caused by decreased N assimilation, because the concentrations of TSPs and TFAADs and the activities of N-assimilatory enzymes decreased in N-deficient leaves and NH_4_^+^-N concentration increased with decreased levels of TSPs and TFAADs and activities of NADH-GOGAT, GOT, GPT and GS (*r* ≤ − 0.889 except for *r* = − 0.817 for NADH-GOGAT; Figs. 2-3 and Table S7). Interestingly, NH_4_^+^-N/NO_3_^−^-N ratios in leaves and roots were higher at 0 mM N than at 5–20 mM N (Fig. 2). Excessive accumulation of NH_4_^+^ is detrimental to plants since it promotes the formation of amides [50]. *Citrus* trees are very sensitive to NH_4_^+^-toxicity [51, 52]. Thus, N-deficiency-induced decreases of N assimilation and TSPs and increases of NH_4_^+^-N and NH_4_^+^-N/NO_3_^−^-N ratio might be responsible for N-deficiency-induced leaf senescence [53]. N-deficiency-induced decreases of N uptake and assimilation have also been found in N-deficient poplar [4, 24, 37], apple [32] and tea [25].

In this study, the concentrations of most FAADs and the activities of five N assimilation-related enzymes in leaves and roots decreased in response to N-deficiency (Tables 1-2). Regressive analysis showed that the concentrations of 33 leaf and 35 root FAADs as well as TFAADs significantly increased with the increase of N concentration, and that the concentration of N significantly increased with increasing activities of NR, NADH-GOGAT, GOT, GPT and GS in leaves and roots except for root GS activity (Tables S6–7). Generally viewed, AA biosynthesis was repressed in N-deficient leaves and roots. In addition, our results demonstrated that N-deficiency decreased TFAADs/N ratio in leaves and roots and that the concentration of TFAADs decreased significantly with the decrease of TFAADs/N ratio in leaves and roots (*r* ≥ 0.990) (Tables S6–7), implying that N-deficiency reduced the relative amount of N used for AA biosynthesis. N-deficiency-induced repression of AA biosynthesis has also been observed in N-deficient tea roots and leaves [25], foxtail millet roots and shoots [16], and poplar leaves, stems and roots [4, 21, 24].

Evidence shows that various stresses lead to a rapid increase of GABA in plants [54]. Unexpectedly, N-deficiency reduced GABA concentrations in leaves and roots (Tables 1-2). Similar results have been obtained in N-deficient poplar leaves and stems [21]. It’s worth noting that the relative amount of GABA was increased or unaltered in N-deficient leaves and roots, respectively (Tables S1–2). Under high-N conditions, plants preferentially accumulated Gln, Asp, Pro and Arg [30]. N-starvation typically causes a large reduction in the level of Gln, the first AA produced in plant NH_4_^+^ assimilation and a decrease in Gln/Glu ratio (a marker for the N-deficiency) [30, 55]. As expected, N-deficiency decreased Gln levels by 94.1% in leaves and 69.6% in roots; Glu levels by 60.4% in leaves and 27.4% in roots; and Gln/Glu ratios by 69.6% in leaves and 58.2% in roots relative to 20 mM N (Tables 1-2). N-deficiency reduced (did not significantly affect) the relative amount of Gln in leaves (roots) relative to 20 mM N, but increased the relative amount of Glu in leaves and roots (Tables S1–2). Arg, which contains 4 N and 6 C, can serve as an N reservoir. Pro can serve as a ready source of N and energy in plants [56]. In addition, Asp and Asn can act as temporary N storage compounds when N assimilation is high [30]. As expected, N-deficiency reduced the concentrations of Arg, Pro, Asp and Asn in leaves and roots and the relative amount of Asp, Asn and Arg in leaves and Pro and Asn in roots. However, N-deficiency increased the relative amount of Pro in leaves and Asp in roots, and had no significant impact on the relative amount of Arg in roots (Tables 1-2 and S1–2). We observed that N-deficiency reduced the concentrations of GSSG in leaves and roots and the relative amount of GSSG in leaves relative to 20 mM N, but did not alter its relative amount in roots (Tables 1-2 and S1–2). N-deficiency-induced reduction of GSSG has also been observed in N-deficient apple leaves [57], tobacco leaves [58] and *Hypericum perforatum* roots [59]. This implied that GSSG could serve as an N storage compound when N was high, because it contains 6 N.

To conclude, N-deficiency increased the relative amount of C-rich (high C/N ratio) FAADs (Glu, Tyr, Trp, Phe, H-TP, H-Tyr-OME, succinic acid and GABA) and decreased the relative amount of N-rich (low C/N ratio) FAADs (Gly, Asn, Cit and Orn) in leaves and roots, thus increasing C/N ratio in TFAADs.

## Conclusions

**Fig. 7:**
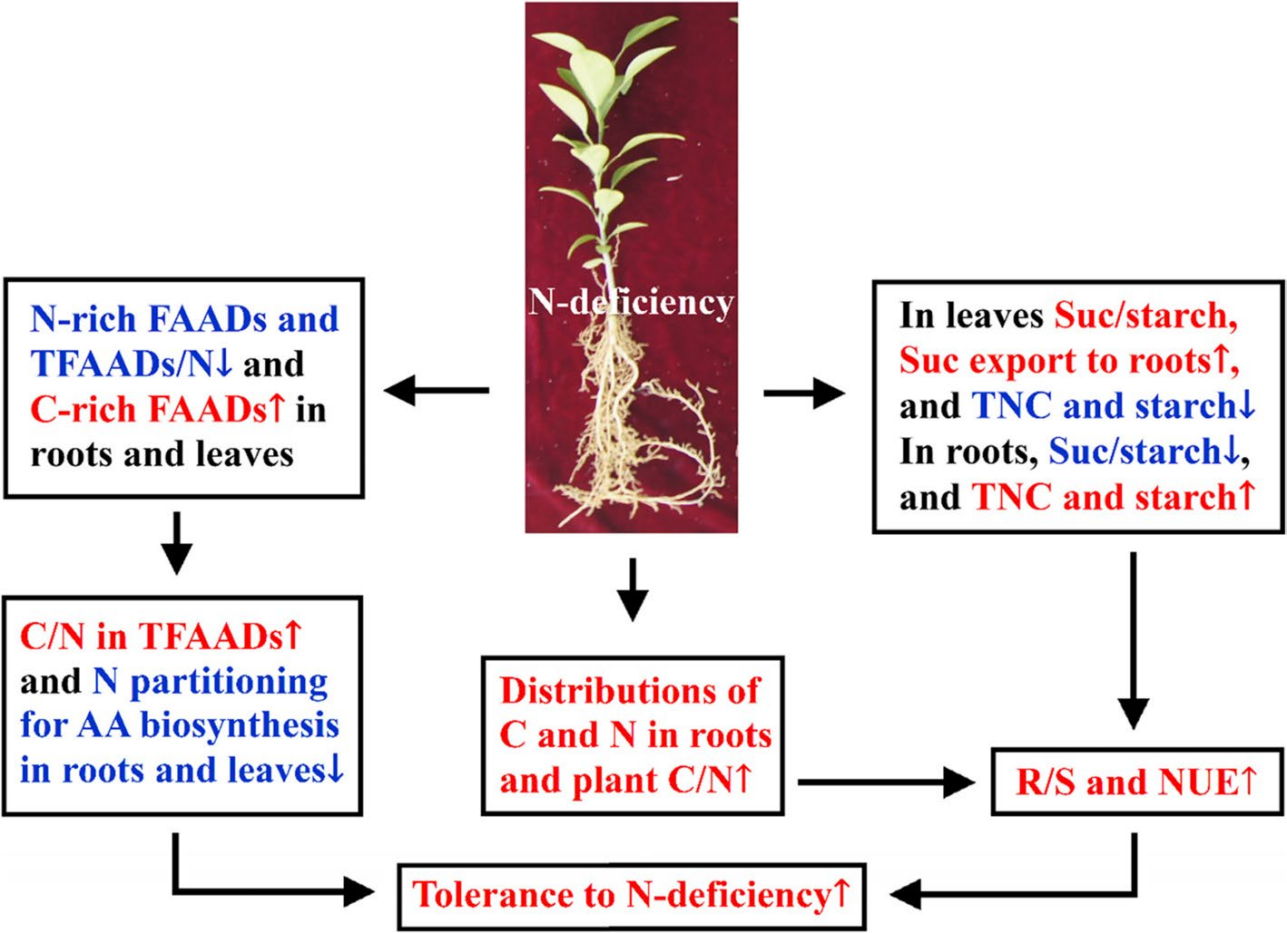
The adaptive responses of C and N metabolisms to N-deficiency in *C. sinensis* leaves and roots\

Nitrogen-deficiency increased sucrose export from leaves to roots, C and N distributions in roots and C/N ratios in roots, stems and leaves, thus improving R/S and NUE. Under N-deficiency, *Citrus* leaves displayed reduced accumulation of starch and TNC and increased ratio of sucrose/starch as well as a partitioning trend of assimilated C toward to sucrose, but roots displayed increased accumulation of starch and TNC and decreased ratio of sucrose/starch as well as a partitioning trend of assimilated C toward to starch. N-deficiency decreased the concentrations of most FAADs and the ratios of TFAADs/N in leaves and roots. N-deficiency reduced the demand for C skeleton precursors for AA biosynthesis, thus reducing TFAADs/C ratios in leaves and roots. N-deficiency increased the relative amounts of C-rich FAADs and decreased the relative amounts of N-rich FAADs, thus increasing the molar ratios of C/N in TFAADs in leaves and roots. To conclude, our results confirmed the hypothesis that C and N metabolisms displayed adaptive responses to N-deficiency in *C. sinensis* seedlings, and that some differences existed between roots and leaves in N-deficiency-induced alterations of and C and N metabolisms (Fig. 7). Based on the present and the previous [3] research, 5 mM N may be suitable for *C. sinensis* growth under sand culture, because seedling growth and most physiological parameters reach normal levels at 5 mM N and did not change with the further increment of N supply. Next step, we will combine physiology, transcriptome and metabolome to further study the adaptive responses of C and N metabolisms to N-deficiency, and screen the key metabolic pathways, genes and/or metabolites that may lead to high NUE, so as to finally improve *C. sinensis* NUE.

## Methods

### Seedling culture and N treatments

Our study did not include any wild plants. We have obtained the permissions to collect cultivated ‘Xuegan’ [*Citrus sinensis* (L.) Osbeck] fruits, which are public and available for non-commercial purpose, from a commercial orchard in Minan village, Tingjiang town, Mawei district, Fuzhou city, Fujian province, China and identified by professor Li-Song Chen. Seeds of *C. sinensis* were germinated in plastic trays filled with river sand washed thoroughly with 0.1‰ HCl followed by tap water. 6 weeks after germination, seedlings were transplanted to 6 L pots (2 plants per pot) filled with river sand and cultivated in a greenhouse under a natural photoperiod at Fujian Agriculture and Forestry University, Fuzhou. For each treatment, 24 seedlings (12 pots) were randomly arranged. 7 weeks after transplantation, seedlings were fertilized thrice weekly with nutrient solution at an N concentration of 0 (N-deficiency), 5 10, 15 or 20 mM until part of the nutrient solution leaking out of the hole at the bottom of the pot (~ 500 mL per pot) according to Huang et al. [3]. 10 weeks after N treatments, ~ 0.5 cm in length white root tips and recent fully expanded mature (~ 7-week-old) leaves (petioles and midribs removed) were collected on a sunny noon and immediately frozen in liquid N_2_, then stored at − 80 °C until extract of enzymes and metabolites. These unsampled seedlings were used to measure C and N concentrations in roots, stems and leaves.

### Carbon and N in leaves, stems and roots, and NH_4_^+^-N and NO_3_^−^-N in leaves and roots

Carbon and N concentrations in leaves, stems and roots were determined with a C/N analyzer (TruMac CN, LECO Corp. MI, USA. C (N) distributions in roots, stems or leaves (%) was calculated as C (N) content in roots, stems or leaves/total C (N) content in plants × 100 [60].

NH_4_^+^-N and NO_3_^−^-N in roots and leaves were determined according to Huang et al. [61].

### Metabolites in leaves and roots

Extraction and measurements of sucrose, fructose, glucose and starch were performed according to Yang et al. [62].

Malate, citrate and isocitrate were extracted and determined according to Chen et al. [63].

Total soluble proteins were determined according to Bradford [64] after being extracted with 50 mM phosphate-buffer solution (pH 7.0).

Free amino acids and derivatives were assayed by Wuhan MetWare Biotechnology Co., Ltd. (Wu, China). Briefly, 50 mg of frozen sample was extracted with 500 μL of 70% (v/v) methanol/water (precooled at − 20 °C). The sample extracts were analyzed using an LC-ESI-MS/MS system (UPLC, ExionLC AD, https://sciex.com.cn /; MS, QTRAP® 6500+ System, https://sciex.com/).

### Enzymes in leaves and roots

Glutamate oxaloacetate transaminase, NADH-GOGAT and GPT were determined according to Lu et al. [65]. NR and GS were extracted and assayed according to Huang et al. [61].

NADP-malic enzyme, NAD-ME, NADP-MDH, NAD-MDH, PK, PEPP, NADP-IDH, ACO, CS and PEPC were extracted and assayed according to Lu et al. [65].

### Data analysis

Results were the means ± SE (*n* = 3 or 4). Data were analyzed by one-way ANOVA followed by LSD at *P* < 0.05 level. Calculation of Pearson correlation coefficients and PCA were performed with the SPSS statistical software (version 17.0, IBM, NY, USA).

## Supplementary Information

**Additional file 1: Figure S1.** Heatmap for the mean concentrations of 63 and 66 FAADs detected in leaves and roots, respectively.

**Additional file 2: Figure S2.** Effects of N supply on mean (±SE, *n* = 3) ratios of TFAADs/N, TFAADs/C and C/N in TFAADs of leaves and roots.

**Additional file 3: Figure S3.** Heatmap for the proportions (as a percentage of TFAADs) of 63 and 66 FAADs detected in leaves and roots, respectively.

**Additional file 4: Figure S4.** Principal component analysis (PCA) loading plots for 105 and 102 physiological parameters in roots and leaves, respectively. **a** Roots. **b** Leaves.

**Additional file 5: Figure S5.** Pearson correlation coefficient matrix between *Citrus sinensis* roots (ordinate) and leaves (abscissa) for the mean values of 54 positively and 8 negatively related physiological parameters.

**Additional file 6: Table S1.** Effects of N supply on mean (±SE, *n* =3) proportions (as a percentage of TFAADs) of FAADs in *Citrus sinensis* leaves.

**Additional file 7: Table S2.** Effects of N supply on mean (±SE, *n* =3) proportions (as a percentage of TFAADs) of FAADs in *Citrus sinensis* roots.

**Additional file 8: Table S3.** PCA for 105 physiological parameters measured here in *Citrus sinensis* roots.

**Additional file 9: Table S4.** PCA for 102 physiological parameters measured here in *Citrus sinensis* leaves.

**Additional file 10: Table S5.** Pearson correlation coefficient matrix between *Citrus sinensis* roots (first column) and leaves (first low) for the mean values of 101 physiological parameters.

**Additional file 11: Table S6.** Pearson correlation coefficient matrix for the mean values of 105 physiological parameters in *Citrus sinensis* roots.

**Additional file 12: Table S7.** Pearson correlation coefficient matrix for the mean values of 102 physiological parameters in *Citrus sinensis* leaves.

## Data Availability

All data analyzed in this study are included in this published article and its additional files.
