# Supplementary material for: Adaptive responses of carbon and nitrogen metabolisms to nitrogen-deficiency in Citrus sinensis seedlings
Source: BMC Plant Biol. 2022 Jul 26;22:370. doi: 10.1186/s12870-022-03759-7 (PMC9316421; doi:10.1186/s12870-022-03759-7)

**Additional file 2: Figure S2.** Effects of N supply on mean (±SE, *n* = 3) ratios of TFAADs/N, TFAADs/C and C/N in TFAADs of leaves and roots. **a** and **d** Leaf and root TFAADs/N. **b** and **e** Leaf and root TFAADs/C. **c** and **f** Leaf and root C/N in TFAADs. Different letters above the bars indicate a significant difference at *P* < 0.05.


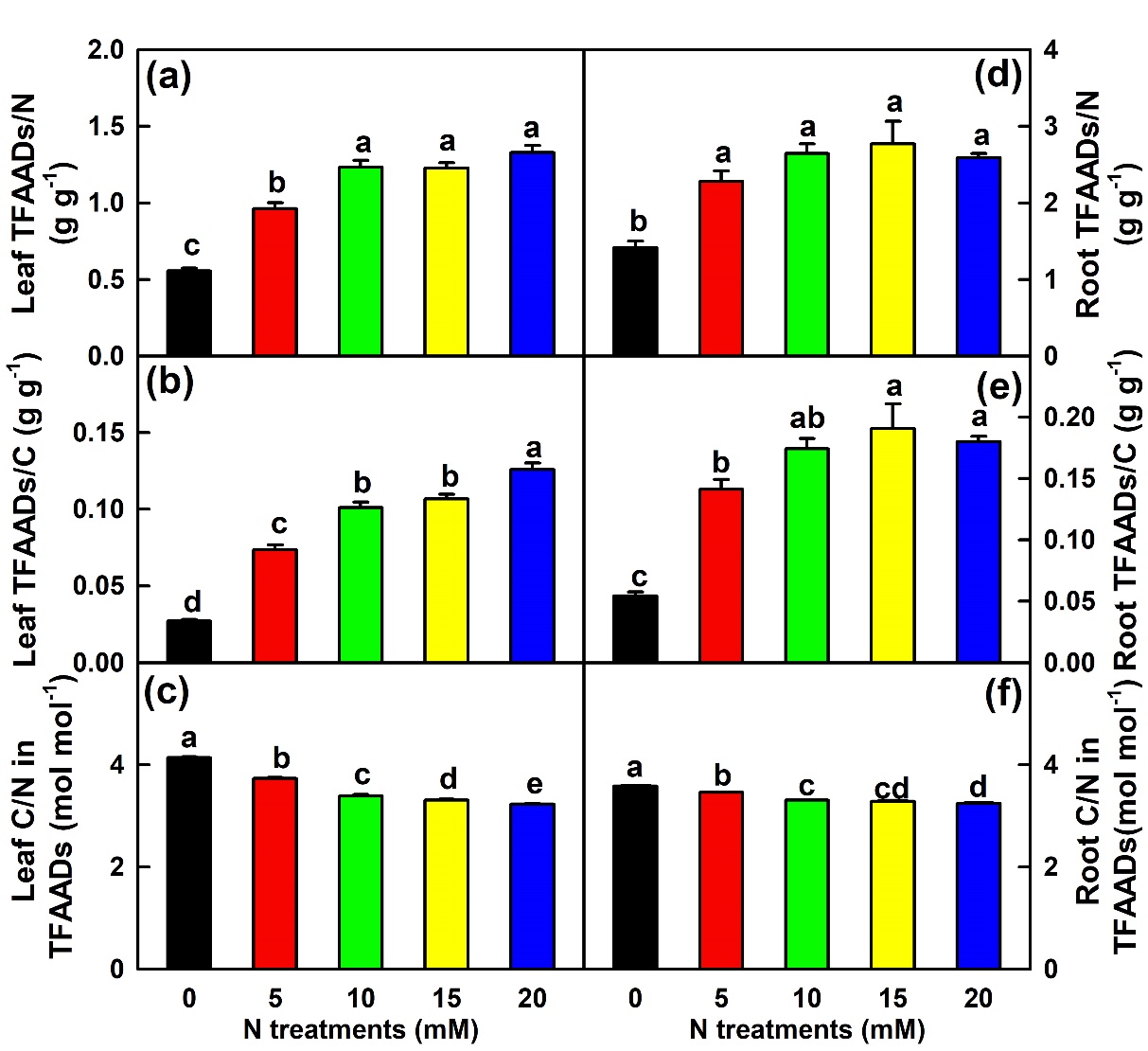

Supplement: Supplementary file 2 — Additional file 2: Figure S2. Effects of N supply on mean (±SE, n = 3) ratios of TFAADs/N, TFAADs/C and C/N in TFAADs of leaves and roots. [file 12870_2022_3759_MOESM2_ESM.docx]
