# Supplementary material for: Adaptive responses of carbon and nitrogen metabolisms to nitrogen-deficiency in Citrus sinensis seedlings
Source: BMC Plant Biol. 2022 Jul 26;22:370. doi: 10.1186/s12870-022-03759-7 (PMC9316421; doi:10.1186/s12870-022-03759-7)

**Additional file 3: Figure S3.** Heatmap for the proportions (as a percentage of TFAADs) of 63 and 66 FAADs detected in leaves and roots, respectively. L0, L5, L10, L15 and L20 indicate 0, 5, 10, 15 and 20 mM N-treated leaves, respectively; R0, R5, R10, R15 and R20 indicate 0, 5, 10, 15 and 20 mM N-treated roots, respectively. The data were converted to log10 values before the Heatmaps were made. FAADs in the figure were arranged from high to low according to their C/N ratio. **a** and **b** Leaves and roots.


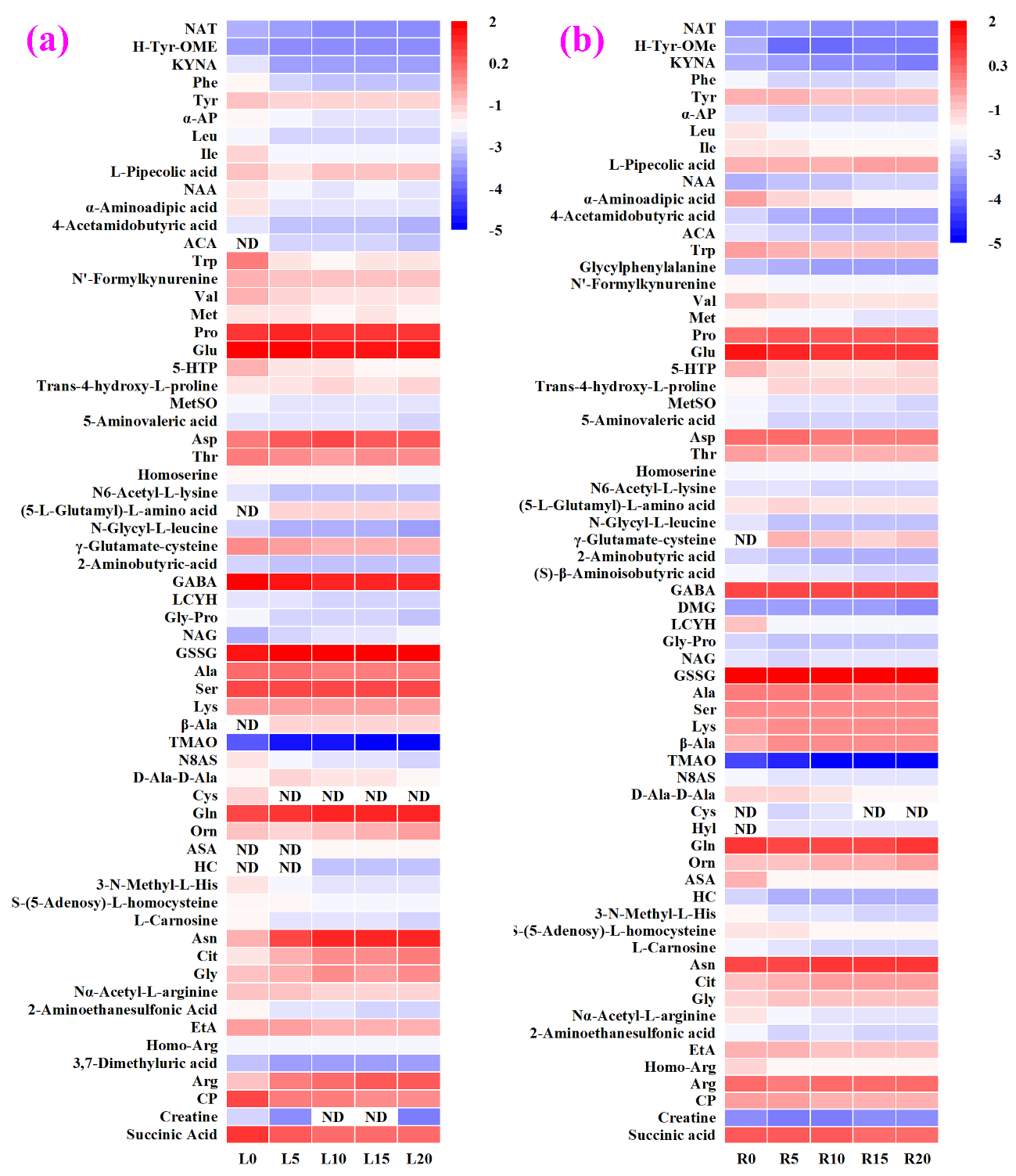

Supplement: Supplementary file 3 — Additional file 3: Figure S3. Heatmap for the proportions (as a percentage of TFAADs) of 63 and 66 FAADs detected in leaves and roots, respectively. [file 12870_2022_3759_MOESM3_ESM.docx]
