# Supplementary material for: Adaptive responses of carbon and nitrogen metabolisms to nitrogen-deficiency in Citrus sinensis seedlings
Source: BMC Plant Biol. 2022 Jul 26;22:370. doi: 10.1186/s12870-022-03759-7 (PMC9316421; doi:10.1186/s12870-022-03759-7)

**Additional file 4: Figure S4.** Principal component analysis (PCA) loading plots for 105 and 102 physiological parameters in roots and leaves, respectively. **a** Roots. **b** Leaves.


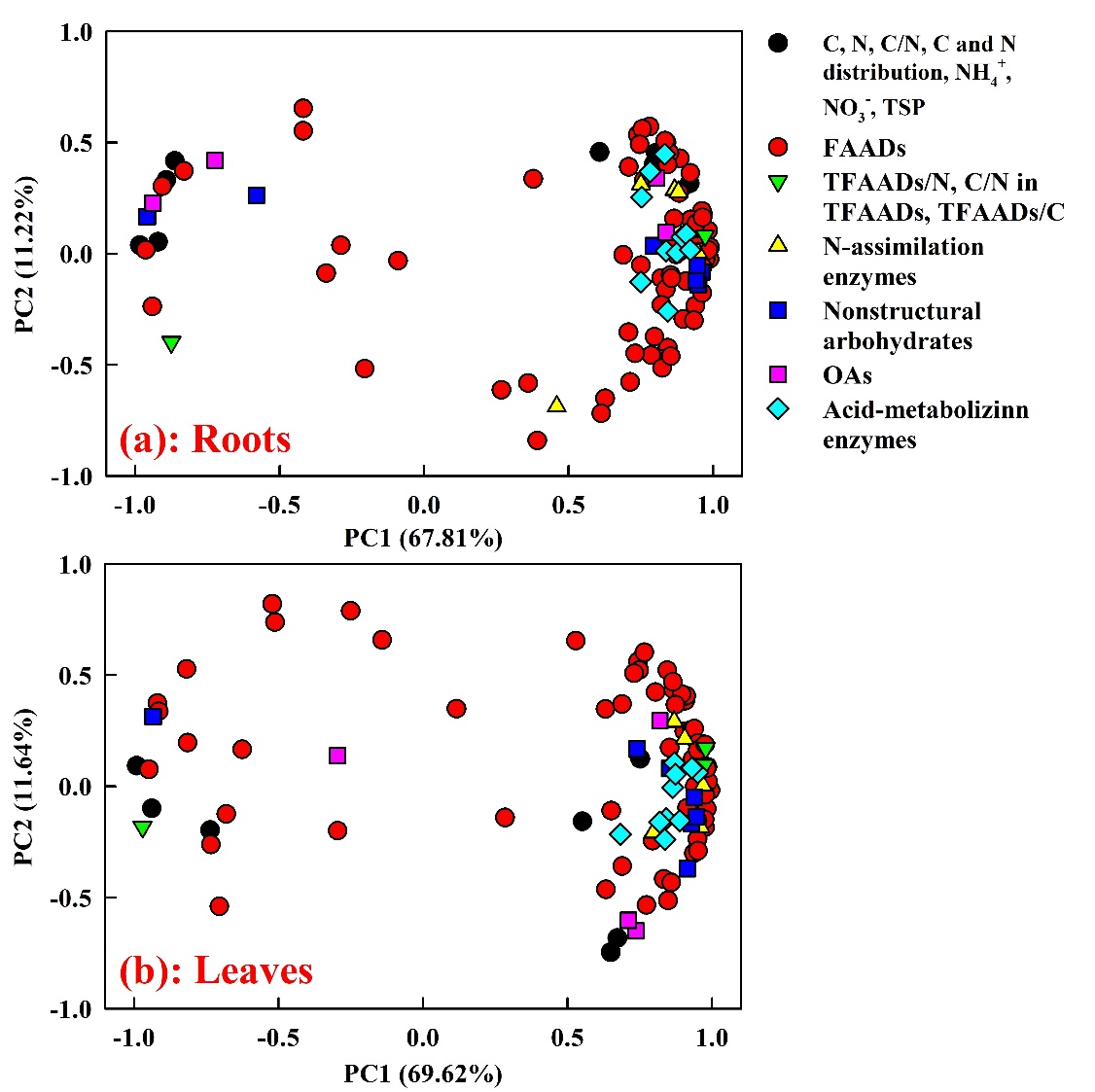

Supplement: Supplementary file 4 — Additional file 4: Figure S4. Principal component analysis (PCA) loading plots for 105 and 102 physiological parameters in roots and leaves, respectively. a Roots. b Leaves. [file 12870_2022_3759_MOESM4_ESM.docx]
