# Supplementary material for: Adaptive responses of carbon and nitrogen metabolisms to nitrogen-deficiency in Citrus sinensis seedlings
Source: BMC Plant Biol. 2022 Jul 26;22:370. doi: 10.1186/s12870-022-03759-7 (PMC9316421; doi:10.1186/s12870-022-03759-7)

**Additional file 5: Figure S5.** Pearson correlation coefficient matrix between *Citrus sinensis* roots (first column) and leaves (abscissa) for the mean values of 54 positively and 8 negatively related physiological parameters. TOAs: malate + citrate + isocitrate.


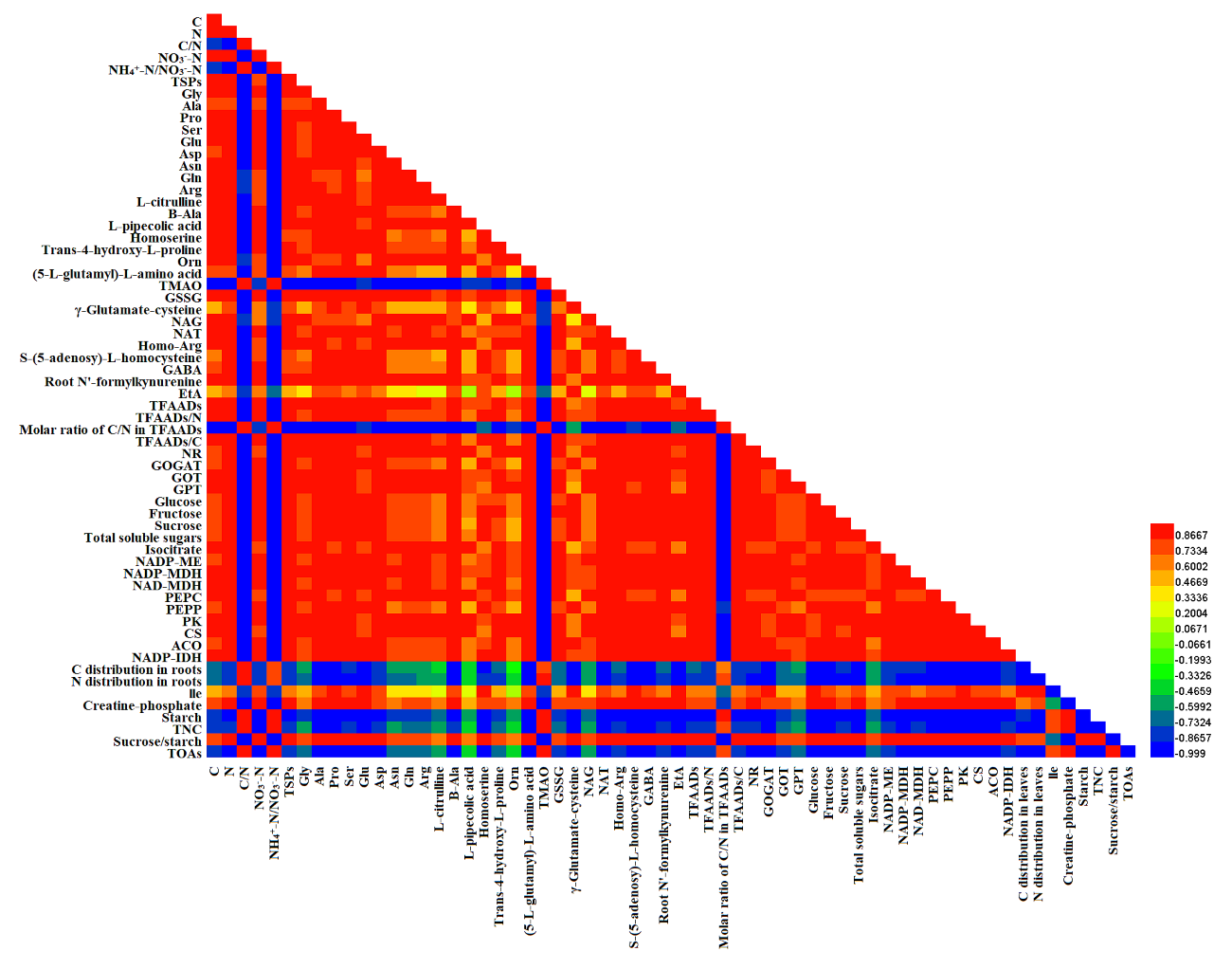

Supplement: Supplementary file 5 — Additional file 5: Figure S5. Pearson correlation coefficient matrix between Citrus sinensis roots (ordinate) and leaves (abscissa) for the mean values of 54 positively and 8 negatively related physiological parameters. [file 12870_2022_3759_MOESM5_ESM.docx]
