# Supplementary material for: Adaptive responses of carbon and nitrogen metabolisms to nitrogen-deficiency in Citrus sinensis seedlings
Source: BMC Plant Biol. 2022 Jul 26;22:370. doi: 10.1186/s12870-022-03759-7 (PMC9316421; doi:10.1186/s12870-022-03759-7)
Supplement: Supplementary file 6 — Additional file 6: Table S1. Effects of N supply on mean (±SE, n =3) proportions (as a percentage of TFAADs) of FAADs in Citrus sinensis leaves. [file 12870_2022_3759_MOESM6_ESM.docx]

**Additional file 6: Table S1.** Effects of N supply on mean (±SE, *n* =3) proportions (as a percentage of TFAADs) of FAADs in *Citrus sinensis* leaves

| **FAADs** | **Molecular formula** | **N treatments (mM)** |  |  |  |  |
| --- | --- | --- | --- | --- | --- | --- |
|  |  | **0** | **5** | **10** | **15** | **20** |
| NAT | C_11_H_13_NO_4_ | 0.00140±0.00007a | 0.00060±0.00005b | 0.00044±0.00001c | 0.00042±0.00002c | 0.00035±0.00003c |
| H-Tyr-OME | C_10_H_14_NO_3_ | 0.00066±0.00007a | 0.00037±0.00003b | 0.00033±0.00002b | 0.00033±0.00001b | 0.00029±0.00000b |
| KYNA | C_10_H_7_NO_3_ | 0.00517±0.00037a | 0.00080±0.00006b | 0.00072±0.00001b | 0.00068±0.00001b | 0.00059±0.00003b |
| Phe | C_9_H_11_NO_2_ | 0.01326±0.0009a | 0.00251±0.0002b | 0.00199±0.0000b | 0.00215±0.0001b | 0.00229±0.0003b |
| Tyr | C_9_H_11_NO_3_ | 0.09985±0.0034a | 0.05534±0.0014b | 0.04237±0.0016c | 0.04431±0.0002c | 0.04245±0.0016c |
| α-AP | C_13_H_16_N_2_O_5_ | 0.01403±0.0003a | 0.00852±0.0001b | 0.00693±0.0004c | 0.00658±0.0001c | 0.00624±0.0002c |
| Leu | C_6_H_13_NO_2_ | 0.00810±0.0011a | 0.00324±0.0002b | 0.00269±0.0001b | 0.00335±0.0001b | 0.00322±0.0002b |
| Ile | C_6_H_13_NO_2_ | 0.04903±0.0014a | 0.00917±0.0005b | 0.00721±0.0002b | 0.00883±0.0001b | 0.00830±0.0001b |
| L-Pipecolic acid | C_6_H_11_NO_2_ | 0.09817±0.0042a | 0.03581±0.0002c | 0.08804±0.0157ab | 0.06796±0.0076b | 0.09891±0.0070a |
| NAA | C_6_H_9_NO_5_ | 0.02312±0.0014a | 0.01020±0.0024b | 0.00602±0.0016bc | 0.00775±0.0006bc | 0.00556±0.0002c |
| α-Aminoadipic acid | C_6_H_11_NO_4_ | 0.02651±0.0050a | 0.00645±0.0004b | 0.00525±0.0004b | 0.00643±0.0003b | 0.00503±0.0004b |
| 4-Acetamidobutyric acid | C_6_H_11_NO_3_ | 0.00505±0.00010a | 0.00230±0.00010b | 0.00165±0.00003c | 0.00153±0.00001c | 0.00126±0.00001d |
| ACA | C_6_H_13_NO_2_ | 0 | 0.00408±0.0001a | 0.00298±0.0001b | 0.00280±0.0001c | 0.00241±0.0001d |
| Trp | C_11_H_12_N_2_O_2_ | 0.79404±0.0644a | 0.02649±0.0006b | 0.02075±0.0004b | 0.02299±0.0012b | 0.02304±0.0043b |
| Glycylphenylalanine | C_11_H_14_N_2_O_3_ | ND | ND | ND | ND | ND |
| N'-Formylkynurenine | C_11_H_12_N_2_O_4_ | 0.15137±0.0119a | 0.08910±0.0036b | 0.06743±0.0021c | 0.06663±0.0019c | 0.06895±0.0013c |
| Val | C_5_H_11_NO_2_ | 0.12732±0.0087a | 0.03657±0.0019b | 0.02539±0.0009b | 0.02815±0.0005b | 0.02595±0.0004b |
| Met | C_5_H_11_NO_2_S | 0.02496±0.0010ab | 0.02721±0.0023a | 0.02088±0.0009c | 0.02307±0.0011bc | 0.02008±0.0004c |
| Pro | C_5_H_9_NO_2_ | 6.34119±0.4410b | 7.68912±0.2818a | 5.77851±0.2122b | 5.72379±0.0612bc | 4.91622±0.1020c |
| Glu | C_5_H_9_NO_4_ | 26.50914±0.8941a | 23.72794±1.1344b | 16.51308±0.4458c | 14.82148±0.3822cd | 13.85054±0.1953d |
| 5-HTP | C_10_H_12_N_2_O | 0.10994±0.0089a | 0.02988±0.0017b | 0.02572±0.0041bc | 0.01394±0.0006c | 0.01326±0.0008c |
| Trans-4-hydroxy-L-proline | C_5_H_9_NO_3_ | 0.02813±0.0004c | 0.03079±0.0015bc | 0.03607±0.0030ab | 0.02966±0.0012c | 0.03646±0.0015a |
| MetSO | C_5_H_11_NO_3_S | 0.01059±0.0002a | 0.00698±0.0004b | 0.00631±0.0002bc | 0.00570±0.0001c | 0.00594±0.0002c |
| 5-Aminovaleric acid | NH_2_(CH_2_)_4_CO_2_H | 0.00520±0.0052a | 0.00643±0.0001a | 0.00475±0.0001a | 0.00427±0.0001a | 0.00371±0.0001a |
| Asp | C_4_H_7_NO_4_ | 0.55101±0.0162b | 2.54888±0.2016a | 2.65977±0.0947a | 2.45750±0.1314a | 2.35866±0.0175a |
| Thr | C_4_H_9_NO_3_ | 0.65431±0.0268a | 0.39749±0.0178b | 0.28615±0.0180c | 0.36854±0.0079b | 0.38581±0.0114b |
| Homoserine | C_4_H_9_NO_3_ | 0.02035±0.0020a | 0.01730±0.0025ab | 0.01317±0.0027bc | 0.01366±0.0009bc | 0.00833±0.0007c |
| N6-Acetyl-L-lysine | C_8_H_16_N_2_O_3_ | 0.00513±0.0002a | 0.00233±0.0001b | 0.00212±0.0001bc | 0.00208±0.0001bc | 0.00176±0.0001c |
| (5-L-Glutamyl)-L-amino acid | C_8_H_14_N_2_O_5_S | ND | 0.05761±0.0023a | 0.04720±0.0021bc | 0.04824±0.0018b | 0.04060±0.0033c |
| N-Glycyl-L-leucine | C_8_H_16_N_2_O_3_ | 0.00381±0.00051a | 0.00132±0.00002b | 0.00095±0.00002b | 0.00102±0.00003b | 0.00080±0.00005b |
| γ-Glutamate-cysteine | C_8_H_14_N_2_O_5_S | 0.50175±0.0115a | 0.26632±0.0105b | 0.17702±0.0196c | 0.15618±0.0113cd | 0.13760±0.0032d |
| 2-Aminobutyric-acid | C_4_H_9_NO | 0.00410±0.0004a | 0.00235±0.0001b | 0.00189±0.0001b | 0.00192±0.0001b | 0.00186±0.0003b |
| (S)-β-Aminoisobutyric-acid | C_4_H_9_NO | ND | ND | ND | ND | ND |
| GABA | C_4_H_9_NO | 26.46813±0.8692a | 16.88724±0.7386b | 12.45439±0.6386c | 12.12193±0.1527cd | 10.35836±0.4971d |
| DMG | C_4_H_9_NO_2_ | ND | ND | ND | ND | ND |
| LCYH | C_7_H_14_N_2_O_4_S | 0.00597±0.0008a | 0.00441±0.0005b | 0.00417±0.0004b | 0.00392±0.0001b | 0.00342±0.0001b |
| Gly-Pro | C_7_H_12_N_2_O_3_ | 0.00742±0.00014a | 0.00393±0.00011b | 0.00258±0.00007c | 0.00252±0.00006c | 0.00199±0.00004d |
| NAG | C_7_H_12_N_2_O_4_ | 0.00130±0.00007d | 0.00356±0.00036c | 0.00500±0.00079c | 0.00700±0.00045b | 0.00851±0.00031a |
| GSSG | C₂₀H₃₂N₆O₁₂S₂ | 18.20273±0.5234c | 28.28968±2.4385b | 36.03832±1.7927a | 36.08633±3.2968a | 37.68618±2.0880a |
| Ala | C_3_H_7_NO_2_ | 1.09184±0.0421a | 1.10146±0.0470a | 0.86552±0.0205b | 0.86493±0.0221b | 0.74829±0.0317c |
| Ser | C_3_H_7_NO_3_ | 2.67373±0.2007b | 3.48519±0.1426a | 2.95612±0.0770b | 2.74604±0.0251b | 2.71916±0.0450b |
| Lys | C_6_H_14_N_2_O_2_ | 0.24223±0.0158ab | 0.19161±0.0060c | 0.18969±0.0157c | 0.22045±0.0027bc | 0.27762±0.0160a |
| β-Ala | C_3_H_7_NO_2_ | ND | 0.04454±0.0036a | 0.04395±0.0035a | 0.03960±0.0011a | 0.03741±0.0010a |
| TMAO | C_3_H_13_NO_3_ | 0.000060±0.000006a | 0.000010±0.000001b | 0.000010±0.000000bc | 0.000006±0.0000002c | 0.000004±0.0000001c |
| N8AS | C_9_H_21_N_3_O | 0.02431±0.00056a | 0.00836±0.00031b | 0.00584±0.00004c | 0.00523±0.00022c | 0.00421±0.00013d |
| D-Ala-D-Ala | C_6_H_12_N_2_O_3_ | 0.01647±0.0007c | 0.03768±0.0011a | 0.02346±0.0003b | 0.02228±0.0004b | 0.01687±0.0002c |
| Cys | C_3_H_7_NO_2_S | 0.05129±0.0084a | ND | ND | ND | ND |
| Hyl | C_6_H_14_N_2_O_3_ | ND | ND | ND | ND | ND |
| Gln | C_5_H_10_N_2_O_3_ | 3.47291±0.2233d | 6.48816±0.3211c | 9.23958±0.6790b | 11.15904±0.3843a | 12.24597±0.4850a |
| Orn | C_5_H_12_N_2_O_2_ | 0.08024±0.0034c | 0.05218±0.0040d | 0.09297±0.0100c | 0.13994±0.0080b | 0.20310±0.0138a |
| ASA | C_10_H_20_N_4_O_6_ | ND | ND | 0.01438±0.0072a | 0.01968±0.0010a | 0.01999±0.0007a |
| HC | C_7_H_15_N_3_O_3_ | ND | ND | 0.00193±0.00001a | 0.00181±0.00002b | 0.00159±0.00001c |
| 3-N-Methyl-L-His | C_7_H_11_N_3_O_2_ | 0.02428±0.0004a | 0.00908±0.0003b | 0.00670±0.0003c | 0.00632±0.0001cd | 0.00579±0.0001d |
| S-(5-Adenosy)-L-homocysteine | C_14_H_20_N_6_O_5_S | 0.01686±0.0005a | 0.01510±0.0011b | 0.01058±0.0001c | 0.01062±0.0003c | 0.00905±0.0002c |
| L-Carnosine | C_9_H_14_N_4_O_3_ | 0.01859±0.0002a | 0.00672±0.0001b | 0.00469±0.0001c | 0.00455±0.0001c | 0.00363±0.0001d |
| Asn | C_4_H_8_N_2_O_3_ | 0.13484±0.0096c | 3.58577±0.4601b | 7.85980±1.0143a | 7.96922±0.3788a | 9.30135±0.2782a |
| Cit | C_6_H_13_N_3_O_3_ | 0.03092±0.0016d | 0.15510±0.0137c | 0.41659±0.0514b | 0.51209±0.0350b | 0.64823±0.0272a |
| Gly | C_2_H_5_NO_2_ | 0.06820±0.0106d | 0.15830±0.0120c | 0.31382±0.0152ab | 0.29595±0.0125b | 0.34259±0.0071a |
| Nα-Acetyl-L-arginine | C_8_H_16_N_4_O_3_ | 0.10287±0.0018a | 0.07138±0.0039b | 0.05731±0.0025bc | 0.05156±0.0016c | 0.04744±0.0059c |
| 2-Aminoethanesulfonic Acid | C_2_H_7_NO_3_S | 0.01643±0.00006a | 0.00591±0.00004b | 0.00436±0.00002c | 0.00398±0.00001d | 0.00352±0.00002e |
| EtA | C_2_H_7_NO | 0.19811± 0.0352b | 0.25457±0.0017a | 0.15530±0.0042bc | 0.15495±0.0066bc | 0.12477±0.0048c |
| Homo-Arg | C_7_H_16_N_4_O_2_ | 0.00975±0.0012a | 0.00904±0.0006a | 0.00767±0.0004a | 0.00806±0.0008a | 0.00843±0.0001a |
| 3,7-Dimethyluric acid | C_7_H_8_N_4_O_3_ | 0.00184±0.00001a | 0.00070±0.00001bc | 0.00057±0.00005cd | 0.00072±0.00003b | 0.00053±0.00007d |
| Arg | C_6_H_14_N_4_O_2_ | 0.08714±0.0087d | 0.82886±0.0572c | 1.27684±0.1406b | 1.82622±0.0463a | 1.85361±0.0767a |
| CP | C_4_H_8_N_3_Na_2_O_5_P | 3.73753±0.1217a | 0.67376±0.0629b | 0.62967±0.0686b | 0.51565±0.0323bc | 0.31875±0.0854c |
| Creatine | C_4_H_9_N_3_O_2_ | 0.00323±0.0001a | 0.00049±0.0005b | ND | ND | 0.00025±0.0002b |
| Succinic Acid | C_4_H_6_O_4_ | 7.02411±0.2350a | 2.52371±0.2812b | 1.46437±0.0634c | 1.25746±0.0340cd | 0.92289±0.0144d |

Note: ND, not detected; Different letters within a row indicate a significant difference at *P* < 0.05. FAADs in the table were arranged from high to low according to their C/N ratio. The same notation will be used in Table S2.
