# Supplementary material for: Adaptive responses of carbon and nitrogen metabolisms to nitrogen-deficiency in Citrus sinensis seedlings
Source: BMC Plant Biol. 2022 Jul 26;22:370. doi: 10.1186/s12870-022-03759-7 (PMC9316421; doi:10.1186/s12870-022-03759-7)
Supplement: Supplementary file 7 — Additional file 7: Table S2. Effects of N supply on mean (±SE, n =3) proportions (as a percentage of TFAADs) of FAADs in Citrus sinensis roots. [file 12870_2022_3759_MOESM7_ESM.docx]

**Additional file 7: Table S2.** Effects of N supply on mean (±SE, *n* =3) proportions (as a percentage of TFAADs) of FAADs in *Citrus sinensis* roots

| **FAADs** | **Molecular formula** | **N treatments (mM)** |  |  |  |  |
| --- | --- | --- | --- | --- | --- | --- |
|  |  | **0** | **5** | **10** | **15** | **20** |
| NAT | C11H13NO4 | 0.00086±0.00001a | 0.00067±0.00004b | 0.00048±0.00003c | 0.00055±0.00005bc | 0.00056±0.00007bc |
| H-Tyr-OMe | C10H14NO3 | 0.00103±0.00008a | 0.00016±0.00001b | 0.00018±0.00004b | 0.00020±0.00003b | 0.00020±0.00002b |
| KYNA | C10H7NO3 | 0.00121±0.00005a | 0.00058±0.00006b | 0.00040±0.00002c | 0.00037±0.00003c | 0.00032±0.00002c |
| Phe | C9H11NO2 | 0.01116±0.0003a | 0.00453±0.0004b | 0.00469±0.0005b | 0.00488±0.0004b | 0.00526±0.0004b |
| Tyr | C9H11NO3 | 0.16171±0.0074a | 0.14473±0.0090ab | 0.12465±0.0053bc | 0.10563±0.0090cd | 0.09770±0.0090d |
| α-AP | C_13_H_16_N_2_O_5_ | 0.00811± 0.0002a | 0.00433±0.0002b | 0.00364±0.0001c | 0.00341±0.0002c | 0.00309±0.0003c |
| Leu | C_6_H_13_NO_2_ | 0.02775±0.0022a | 0.01351±0.0006b | 0.01155± 0.0008bc | 0.01027±0.0007bc | 0.00969±0.0006c |
| Ile | C_6_H_13_NO_2_ | 0.04544±0.0041a | 0.03533±0.0019b | 0.02676±0.0017c | 0.02155±0.0017c | 0.02020±0.0016c |
| L-Pipecolic acid | C_6_H_11_NO_2_ | 0.15466±0.0279c | 0.18887±0.0239bc | 0.23067±0.0143abc | 0.25441±0.0111ab | 0.28712±0.0473a |
| NAA | C_6_H_9_NO_5_ | 0.00147±0.0003b | 0.00267±0.0002ab | 0.00263±0.0006ab | 0.00359±0.0004a | 0.00326±0.0009a |
| α-Aminoadipic acid | C_6_H_11_NO_4_ | 0.37946±0.0210a | 0.04946±0.0065b | 0.02771±0.0020b | 0.01974±0.0012b | 0.02008±0.0021b |
| 4-Acetamidobutyric acid | C_6_H_11_NO_3_ | 0.00357±0.00023a | 0.00139±0.00002b | 0.00097±0.00007c | 0.00087±0.00005c | 0.00083±0.00007c |
| ACA | C_6_H_13_NO_2_ | 0.00641±0.0001a | 0.00380±0.0001b | 0.00242±0.0001c | 0.00215±0.0001d | 0.00209±0.0001d |
| Trp | C_11_H_12_N_2_O_2_ | 0.36845±0.0310a | 0.14066±0.0075b | 0.09942±0.0058c | 0.09596±0.0044c | 0.09402±0.0075c |
| Glycylphenylalanine | C_11_H_14_N_2_O_3_ | 0.00212±0.00012a | 0.00105±0.00002b | 0.00082±0.00001c | 0.00073±0.00002c | 0.00064±0.00002c |
| N'-Formylkynurenine | C_11_H_12_N_2_O_4_ | 0.02578±0.0029a | 0.01430±0.0001b | 0.01205±0.0002b | 0.01216±0.0007b | 0.01257±0.0009b |
| Val | C_5_H_11_NO_2_ | 0.09692±0.0081a | 0.05459±0.0027b | 0.03726±0.0015c | 0.03131±0.0018c | 0.03153±0.0015c |
| Met | C_5_H_11_NO_2_S | 0.01940±0.0004a | 0.01384±0.0004b | 0.01052±0.0005c | 0.00803±0.0006d | 0.00864±0.0003d |
| Pro | C_5_H_9_NO_2_ | 1.93868±0.1173b | 2.95057±0.0536a | 2.87454±0.1826a | 2.75078±0.1714a | 2.75478±0.0090a |
| Glu | C_5_H_9_NO_4_ | 25.30738±0.8723a | 12.41336±0.5019b | 9.76598±0.3922c | 9.12518±0.1270c | 9.35027±0.1029c |
| 5-HTP | C_10_H_12_N_2_O | 0.18922±0.0095a | 0.05219±0.0051b | 0.04036±0.0010b | 0.04290±0.0027b | 0.04718±0.0046b |
| Trans-4-hydroxy-L-proline | C_5_H_9_NO_3_ | 0.01980±0.0030b | 0.07384±0.0016a | 0.07372±0.0052a | 0.06926±0.0059a | 0.06763±0.0071a |
| MetSO | C_5_H_11_NO_3_S | 0.01127±0.0003a | 0.00846±0.0006b | 0.00610±0.0001c | 0.00519±0.0003cd | 0.00483±0.0002d |
| 5-Aminovaleric acid | NH_2_(CH_2_)_4_CO_2_H | 0.01008±0.0004a | 0.00434±0.0001b | 0.00370±0.0002b | 0.00356±0.0002b | 0.00361±0.0003b |
| Asp | C_4_H_7_NO_4_ | 2.18654±0.2340a | 1.63141±0.0603b | 1.06833±0.0658c | 1.17717±0.0195c | 1.24922±0.0331c |
| Thr | C_4_H_9_NO_3_ | 0.30864±0.0061a | 0.23257±0.0159b | 0.21216±0.0115bc | 0.18673±0.0115c | 0.18496±0.0131c |
| Homoserine | C_4_H_9_NO_3_ | 0.00903±0.0002a | 0.01367±0.0020a | 0.01162± 0.0018a | 0.01140± 0.0002a | 0.00958±0.0031a |
| N6-Acetyl-L-lysine | C_8_H_16_N_2_O_3_ | 0.00851±0.0002a | 0.00672±0.0005b | 0.00473±0.0005c | 0.00408±0.0004c | 0.00386±0.0006c |
| (5-L-Glutamyl)-L-amino acid | C_8_H_14_N_2_O_5_S | 0.03745±0.0017bc | 0.04849±0.0035a | 0.04468±0.0042ab | 0.03405±0.0023c | 0.03086±0.0034c |
| N-Glycyl-L-leucine | C_8_H_16_N_2_O_3_ | 0.00547±0.00042a | 0.00275±0.00021b | 0.00253±0.00005bc | 0.00238±0.00015bc | 0.00186±0.00009c |
| γ-Glutamate-cysteine | C_8_H_14_N_2_O_5_S | ND | 0.14317±0.0129a | 0.09703±0.0143ab | 0.06267±0.0258b | 0.08639±0.0194b |
| 2-Aminobutyric acid | C_4_H_9_NO | 0.00314±0.0002a | 0.00219±0.0003b | 0.00151±0.0002c | 0.00140±0.0002c | 0.00130±0.0002c |
| (S)-β-Aminoisobutyric acid | C_4_H_9_NO | 0.00933±0.0007a | 0.00636±0.0004b | 0.00527±0.0007b | 0.00480±0.0006b | 0.00465±0.0007b |
| GABA | C_4_H_9_NO | 4.59159±0.1244b | 6.55142±0.0541a | 5.35841±0.5900b | 5.09163±0.3477b | 4.53105±0.4104b |
| DMG | C_4_H_9_NO_2_ | 0.00079±0.00039a | 0.00085±0.00007a | 0.00063±0.00010a | 0.00058±0.00005a | 0.00053±0.00005a |
| LCYH | C_7_H_14_N_2_O_4_S | 0.09868±0.0122a | 0.01031±0.0008b | 0.01063±0.0004bb | 0.01105± 0.0016b | 0.01123± 0.0008b |
| Gly-Pro | C_7_H_12_N_2_O_3_ | 0.00386±0.00003a | 0.00253±0.00010bc | 0.00263±0.00017b | 0.00265±0.00015b | 0.00222±0.00010c |
| NAG | C_7_H_12_N_2_O_4_ | 0.00859±0.0005a | 0.00411± 0.0004c | 0.00673±0.0008ab | 0.00608±0.0011bc | 0.00764±0.0008ab |
| GSSG | C₂₀H₃₂N₆O₁₂S₂ | 42.97827±4.6703a | 56.07301±4.3967a | 58.94577±3.5011a | 59.71595±9.4397a | 57.75889±2.0438a |
| Ala | C_3_H_7_NO_2_ | 0.88103±0.0382a | 0.81922±0.0638ab | 0.87923±0.0477a | 0.68332±0.0549bc | 0.60886±0.0822c |
| Ser | C_3_H_7_NO_3_ | 0.47868±0.0077b | 0.60661±0.0262a | 0.57088±0.0486ab | 0.50383±0.0282ab | 0.50794±0.0384ab |
| Lys | C_6_H_14_N_2_O_2_ | 0.28376±0.0207b | 0.53873±0.0283a | 0.66136±0.0672a | 0.59078±0.0643a | 0.62258±0.0363a |
| β-Ala | C_3_H_7_NO_2_ | 0.23161±0.0198b | 0.63507±0.0271a | 0.64012±0.0798a | 0.56388±0.0605a | 0.53297±0.0824a |
| TMAO | C_3_H_13_NO_3_ | 0.000048±0.0000009a | 0.000012±0.0000007b | 0.000004±0.0000002c | 0.000004±0.0000001c | 0.000004±0.0000001c |
| N8AS | C_9_H_21_N_3_O | 0.01423±0.00084a | 0.00831±0.00049b | 0.00646±0.00002c | 0.00627±0.00027c | 0.00622±0.00025c |
| D-Ala-D-Ala | C_6_H_12_N_2_O_3_ | 0.06045±0.0012a | 0.05190±0.0018b | 0.03318±0.0032c | 0.02350±0.0022d | 0.01849±0.0013d |
| Cys | C_3_H_7_NO_2_S | ND | 0.00432±0.0023 | 0.00566±0.0003a | ND | ND |
| Hyl | C_6_H_14_N_2_O_3_ | ND | 0.00742±0.00004a | 0.00652±0.00013b | 0.00632±0.00029b | 0.00652±0.00031b |
| Gln | C_5_H_10_N_2_O_3_ | 8.60806±0.5079a | 4.88314±0.1864d | 5.62895±0.1156cd | 6.35120±0.5982bc | 7.61152±0.4555ab |
| Orn | C_5_H_12_N_2_O_2_ | 0.10452±0.0057b | 0.12454±0.0098b | 0.24167±0.0295a | 0.23899±0.0406a | 0.29228±0.0226a |
| ASA | C_10_H_20_N_4_O_6_ | 0.19869±0.0040a | 0.01926±0.0005b | 0.01976±0.0017b | 0.01907±0.0009b | 0.01923±0.0007b |
| HC | C_7_H_15_N_3_O_3_ | 0.00380±0.00006a | 0.00163±0.00003b | 0.00138±0.00007c | 0.00117± 0.00002d | 0.00122±0.00004d |
| 3-N-Methyl-L-His | C_7_H_11_N_3_O_2_ | 0.02108±0.0010a | 0.00752±0.0005b | 0.00551±0.0004c | 0.00499±0.0002c | 0.00507±0.0003c |
| S-(5-Adenosy)-L-homocysteine | C_14_H_20_N_6_O_5_S | 0.03142±0.0003a | 0.03306±0.0009a | 0.02368±0.0014b | 0.02078±0.0012b | 0.02196±0.0020b |
| L-Carnosine | C_9_H_14_N_4_O_3_ | 0.01046±0.0001a | 0.00551±0.0003b | 0.00496±0.0002b | 0.00436±0.0001c | 0.00389±0.0001c |
| Asn | C_4_H_8_N_2_O_3_ | 4.11398± 0.2384c | 6.08145±0.5347b | 7.70321±0.2151a | 7.90592±0.5211a | 8.78668±0.2026a |
| Cit | C_6_H_13_N_3_O_3_ | 0.09553±0.0136c | 0.21250±0.0061b | 0.31341±0.0254a | 0.31558±0.0423a | 0.38364±0.0091a |
| Gly | C_2_H_5_NO_2_ | 0.07250±0.0072b | 0.11860±0.0023a | 0.12552±0.0127a | 0.11846± 0.0068a | 0.11426± 0.0135a |
| Nα-Acetyl-L-arginine | C_8_H_16_N_4_O_3_ | 0.03086±0.0030a | 0.01035±0.0005b | 0.00867±0.0005b | 0.00720±0.0003b | 0.00682±0.0011b |
| 2-Aminoethanesulfonic acid | C_2_H_7_NO_3_S | 0.00935±0.0002a | 0.00478±0.0002b | 0.00531±0.0006b | 0.00490±0.0005b | 0.00503±0.0003b |
| EtA | C_2_H_7_NO | 0.18037±0.0132a | 0.16816±0.0112a | 0.12189±0.0039b | 0.09311± 0.0165bc | 0.08565±0.0038c |
| Homo-Arg | C_7_H_16_N_4_O_2_ | 0.04807±0.0005a | 0.02363±0.0010b | 0.02376±0.0021b | 0.02179±0.0005b | 0.02494±0.0027b |
| 3,7-Dimethyluric-acid | C_7_H_8_N_4_O_3_ | ND | ND | ND | ND | ND |
| Arg | C_6_H_14_N_4_O_2_ | 1.51027±0.0770a | 0.93796±0.0160b | 1.35179±0.0457a | 1.29889±0.1182a | 1.46720±0.0882a |
| CP | C_4_H_8_N_3_Na_2_O_5_P | 0.38218±0.0288a | 0.28932±0.0627ab | 0.18384±0.0283b | 0.22075±0.0146b | 0.21642±0.0112b |
| Creatine | C_4_H_9_N_3_O_2_ | 0.00054±0.00054a | 0.00032±0.00032a | 0.00023±0.00023a | 0.00050±0.00002a | 0.00052±0.00003a |
| Succinic acid | C_4_H_6_O_4_ | 3.58672±0.2634a | 3.49990±0.1518a | 2.29915±0.0516b | 2.10915±0.1217b | 1.93974±0.1865b |
