# Supplementary material for: Adaptive responses of carbon and nitrogen metabolisms to nitrogen-deficiency in Citrus sinensis seedlings
Source: BMC Plant Biol. 2022 Jul 26;22:370. doi: 10.1186/s12870-022-03759-7 (PMC9316421; doi:10.1186/s12870-022-03759-7)
Supplement: Supplementary file 8 — Additional file 8: Table S3. PCA for 105 physiological parameters measured here in Citrus sinensis roots. [file 12870_2022_3759_MOESM8_ESM.docx]

| **Additional file 8: Table S3.** PCA for 105 physiological parameters measured here in *Citrus sinensis* roots | | | | | | | |  |  |  |
| --- | --- | --- | --- | --- | --- | --- | --- | --- | --- | --- |
|  | Pricipal component | | | | | | | | | |
|  | 1 | 2 | 3 | 4 | 5 | 6 | 7 | 8 | 9 | 10 |
| C concentration | 0.801 | 0.453 | 0.062 | -0.196 | -0.033 | -0.146 | -0.047 | 0.066 | 0.035 | 0.216 |
| N concentration | 0.964 | 0.180 | -0.074 | -0.099 | -0.016 | -0.020 | 0.045 | 0.012 | 0.057 | 0.122 |
| C/N | -0.984 | 0.038 | 0.149 | 0.057 | 0.025 | -0.030 | -0.029 | -0.003 | -0.013 | -0.043 |
| C distribution | -0.862 | 0.417 | 0.199 | -0.007 | 0.081 | -0.081 | 0.010 | 0.018 | 0.000 | 0.159 |
| N distribution | -0.892 | 0.331 | 0.217 | 0.028 | -0.008 | -0.099 | 0.050 | -0.051 | 0.024 | 0.170 |
| NH_4_^+^-N concentration | 0.607 | 0.456 | 0.045 | -0.217 | 0.067 | -0.005 | 0.176 | 0.134 | 0.520 | 0.036 |
| NO_3_^-^-N concentration | 0.918 | 0.315 | -0.061 | -0.028 | 0.078 | -0.108 | 0.036 | -0.135 | 0.072 | 0.022 |
| NH_4_^+^-N/NO_3_^-^-N | -0.921 | 0.053 | 0.216 | 0.074 | -0.023 | 0.024 | 0.029 | 0.045 | 0.184 | -0.064 |
| TSP concentration | 0.794 | 0.402 | -0.178 | -0.304 | 0.151 | -0.202 | -0.066 | 0.040 | -0.021 | 0.008 |
| Gly concentration | 0.972 | 0.069 | 0.158 | -0.011 | -0.018 | -0.124 | -0.003 | -0.054 | -0.005 | 0.020 |
| Ala concentration | 0.906 | -0.125 | 0.272 | 0.107 | -0.253 | -0.028 | -0.031 | -0.033 | 0.057 | 0.042 |
| Val concentration | 0.626 | -0.651 | 0.181 | 0.036 | 0.197 | 0.239 | -0.129 | -0.007 | 0.067 | -0.007 |
| Leu concentration | 0.820 | -0.110 | 0.409 | 0.141 | 0.076 | -0.012 | -0.146 | 0.040 | -0.056 | 0.007 |
| Met concentration | 0.843 | -0.424 | 0.033 | 0.000 | -0.121 | 0.060 | -0.174 | 0.004 | 0.101 | -0.110 |
| lle concentration | 0.829 | -0.474 | 0.192 | 0.131 | 0.050 | 0.063 | -0.084 | -0.040 | 0.054 | -0.042 |
| Pro concentration | 0.982 | 0.104 | -0.042 | 0.056 | -0.071 | -0.035 | -0.071 | -0.046 | -0.047 | -0.020 |
| Ser concentration | 0.987 | -0.024 | 0.125 | -0.007 | -0.022 | -0.045 | -0.050 | -0.048 | 0.023 | -0.004 |
| Trp concentration | -0.090 | -0.031 | 0.057 | 0.745 | 0.514 | 0.282 | -0.060 | -0.004 | 0.061 | -0.037 |
| Phe concentration | 0.739 | 0.534 | 0.281 | -0.006 | 0.116 | -0.112 | -0.084 | -0.185 | -0.027 | -0.069 |
| Tyr concentration | 0.938 | -0.234 | 0.194 | 0.072 | -0.038 | -0.027 | -0.083 | -0.006 | -0.014 | -0.024 |
| Glu concentration | 0.957 | 0.045 | 0.101 | 0.014 | 0.072 | 0.094 | 0.037 | -0.068 | -0.048 | -0.051 |
| Asp concentration | 0.884 | 0.008 | -0.222 | -0.046 | 0.309 | 0.059 | 0.085 | -0.012 | -0.093 | -0.061 |
| Asn concentration | 0.921 | 0.362 | -0.039 | 0.046 | -0.020 | -0.018 | -0.087 | 0.037 | -0.014 | -0.042 |
| Gln concentration | 0.780 | 0.571 | -0.146 | 0.044 | 0.051 | -0.022 | -0.107 | 0.060 | -0.040 | -0.110 |
| Lys concentration | 0.961 | 0.191 | 0.148 | 0.074 | -0.038 | -0.002 | -0.050 | 0.019 | 0.034 | -0.041 |
| Arg concentration | 0.838 | 0.499 | -0.046 | 0.126 | -0.110 | -0.014 | -0.090 | 0.005 | 0.012 | -0.097 |
| Thr concentration | 0.973 | -0.025 | 0.180 | 0.080 | -0.041 | 0.002 | -0.065 | -0.040 | 0.045 | 0.017 |
| L-Citrulline concentration | 0.885 | 0.428 | -0.046 | 0.059 | -0.040 | -0.037 | -0.106 | -0.037 | 0.042 | -0.075 |
| 5-Hydroxy-tryptamine concentration | -0.418 | 0.653 | -0.009 | 0.437 | 0.314 | 0.218 | -0.128 | 0.099 | 0.095 | 0.031 |
| L-Homocitrulline concentration | 0.857 | 0.042 | 0.376 | -0.047 | -0.190 | -0.048 | -0.087 | -0.151 | 0.183 | -0.002 |
| Beta-alanine concentration | 0.963 | -0.060 | 0.241 | -0.028 | -0.004 | -0.082 | -0.014 | -0.027 | 0.001 | 0.009 |
| L-Pipecolic acid concentration | 0.849 | 0.452 | -0.099 | 0.098 | -0.021 | 0.175 | 0.069 | -0.006 | -0.015 | 0.006 |
| 3-N-Methyl-L-histidine concentration | -0.339 | -0.088 | 0.746 | -0.249 | 0.224 | 0.274 | -0.009 | -0.174 | -0.051 | 0.190 |
| Homoserine concentration | 0.836 | -0.161 | 0.252 | -0.209 | 0.044 | -0.148 | 0.116 | -0.286 | -0.097 | 0.048 |
| L-Cystathionine concentration | -0.831 | 0.371 | 0.220 | 0.148 | 0.026 | 0.060 | -0.191 | -0.004 | -0.018 | 0.099 |
| N6-Acetyl-L-lysine concentration | 0.786 | -0.457 | 0.358 | -0.037 | 0.167 | -0.030 | -0.029 | -0.040 | 0.058 | 0.072 |
| Trans-4-Hydroxy-L-proline concentration | 0.989 | 0.028 | 0.078 | 0.040 | -0.001 | -0.081 | 0.009 | -0.007 | 0.016 | 0.020 |
| L-Ornithine concentration | 0.833 | 0.508 | 0.094 | 0.112 | -0.025 | 0.026 | -0.090 | 0.003 | 0.051 | -0.088 |
| L-tyrosine methyl ester concentration | -0.418 | 0.552 | 0.647 | 0.010 | 0.142 | -0.202 | 0.111 | 0.035 | -0.013 | -0.028 |
| N-Acetylaspartate concentration | 0.760 | 0.331 | -0.169 | 0.110 | -0.100 | 0.214 | 0.225 | -0.183 | -0.031 | 0.310 |
| (5-L-Glutamyl)-L-amino acid concentration | 0.896 | -0.294 | 0.197 | -0.069 | -0.180 | -0.008 | -0.041 | -0.075 | 0.014 | 0.036 |
| Glycyl-L-proline concentration | 0.922 | 0.153 | 0.177 | 0.179 | -0.110 | -0.114 | -0.013 | -0.015 | -0.153 | 0.101 |
| Trimethylamine N-oxide concentration | -0.940 | -0.238 | 0.057 | -0.007 | 0.135 | 0.111 | 0.015 | -0.096 | -0.028 | 0.002 |
| N8-Acetylspermidine concentration | 0.945 | 0.033 | -0.001 | 0.037 | 0.008 | 0.208 | 0.008 | -0.034 | -0.143 | 0.033 |
| Glutathione oxidized concentration | 0.942 | 0.135 | 0.172 | 0.014 | 0.025 | -0.018 | -0.005 | 0.211 | -0.091 | 0.002 |
| Methionine sulfoxide concentration | 0.824 | -0.515 | -0.016 | 0.085 | -0.043 | -0.072 | -0.016 | 0.116 | 0.049 | 0.114 |
| Asp-Phe concentration | 0.870 | -0.004 | 0.041 | 0.379 | -0.152 | -0.004 | 0.054 | -0.038 | -0.029 | 0.157 |
| Nα-Acetyl-L-arginine concentration | -0.287 | 0.036 | 0.127 | 0.632 | -0.367 | 0.287 | 0.432 | -0.138 | 0.148 | -0.157 |
| N-Glycyl-L-leucine concentration | 0.750 | -0.052 | 0.094 | 0.427 | -0.297 | -0.286 | 0.137 | 0.026 | -0.172 | 0.092 |
| γ-Glutamate-cysteine concentration | 0.797 | -0.373 | 0.206 | -0.065 | 0.172 | 0.077 | 0.046 | 0.245 | 0.257 | -0.008 |
| Nα-Acetyl-L-glutamine concentration | 0.754 | 0.561 | 0.148 | 0.161 | -0.019 | 0.030 | -0.003 | -0.019 | 0.128 | -0.165 |
| N-Acetyl-L-tyrosine concentration | 0.865 | 0.158 | -0.092 | 0.183 | 0.236 | 0.283 | 0.039 | 0.130 | -0.124 | -0.023 |
| D-Alanyl-D-alanine concentration | 0.391 | -0.841 | -0.178 | -0.045 | -0.242 | 0.087 | 0.145 | 0.027 | -0.052 | 0.022 |
| Homo-Arg concentration | 0.842 | 0.401 | 0.148 | -0.019 | -0.043 | 0.005 | 0.122 | -0.185 | 0.208 | 0.063 |
| L-Carnosine concentration | 0.853 | -0.096 | -0.007 | 0.054 | -0.367 | -0.062 | 0.176 | 0.083 | -0.205 | -0.018 |
| Glycylphenylalanine concentration | 0.730 | -0.449 | 0.055 | 0.306 | -0.181 | -0.180 | 0.024 | 0.017 | -0.231 | -0.077 |
| S-(5-Adenosy)-L-homocysteine concentration | 0.933 | -0.301 | 0.038 | -0.126 | 0.096 | -0.015 | 0.014 | 0.001 | 0.077 | 0.048 |
| Argininosuccinic-acid concentration | -0.906 | 0.303 | 0.273 | 0.020 | 0.002 | -0.071 | -0.041 | 0.001 | -0.011 | 0.045 |
| Succinic-acid concentration | 0.853 | -0.462 | 0.053 | -0.057 | 0.063 | 0.089 | 0.126 | -0.051 | -0.076 | 0.020 |
| 5-Aminovaleric-acid concentration | 0.708 | 0.390 | -0.262 | 0.409 | -0.131 | 0.181 | 0.075 | -0.113 | 0.096 | 0.147 |
| α-Aminoadipic-acid concentration | -0.963 | 0.017 | 0.215 | 0.045 | 0.035 | 0.082 | -0.065 | -0.047 | 0.039 | 0.052 |
| 2-Aminoethanesulfonic-acid concentration | 0.746 | 0.491 | 0.323 | 0.081 | -0.099 | -0.232 | -0.096 | -0.072 | 0.014 | -0.063 |
| (S)-β-Aminoisobutyric-acid concentration | 0.856 | -0.111 | 0.425 | -0.007 | 0.142 | -0.084 | 0.081 | -0.137 | -0.008 | -0.100 |
| γ-Aminobutyric-acid concentration | 0.962 | -0.178 | 0.138 | -0.025 | 0.036 | -0.076 | 0.018 | -0.029 | -0.089 | 0.036 |
| 4-Acetamidobutyric-acid concentration | -0.206 | -0.517 | 0.633 | 0.017 | 0.286 | -0.204 | 0.372 | 0.045 | 0.040 | -0.082 |
| 6-Aminocaproic-acid concentration | 0.613 | -0.719 | -0.084 | -0.002 | 0.213 | -0.042 | -0.018 | 0.016 | -0.075 | -0.055 |
| Creatine-phosphate concentration | 0.688 | -0.007 | -0.436 | 0.237 | 0.182 | -0.162 | -0.089 | 0.191 | 0.079 | 0.355 |
| Kynurenic-acid concentration | 0.360 | -0.582 | 0.368 | 0.346 | 0.227 | -0.259 | 0.070 | 0.290 | 0.045 | 0.190 |
| N'-Formylkynurenine concentration | 0.882 | 0.272 | -0.134 | 0.187 | 0.083 | 0.148 | -0.061 | 0.130 | -0.104 | -0.039 |
| 2-Aminobutyric-acid concentration | 0.706 | -0.354 | 0.428 | 0.024 | 0.195 | 0.078 | -0.187 | -0.164 | -0.106 | -0.073 |
| Ethanolamine concentration | 0.713 | -0.577 | 0.084 | -0.073 | -0.143 | 0.180 | 0.056 | 0.160 | 0.008 | 0.067 |
| L-Cysteine concentration | 0.268 | -0.613 | 0.058 | 0.194 | -0.404 | -0.223 | -0.164 | 0.156 | 0.384 | -0.085 |
| Creatine concentration | 0.377 | 0.336 | -0.243 | 0.032 | 0.317 | -0.346 | 0.554 | 0.227 | -0.079 | -0.196 |
| N,N-Dimethylglycine concentration | 0.821 | -0.231 | 0.205 | -0.050 | 0.073 | 0.247 | -0.157 | -0.087 | -0.058 | 0.234 |
| 5-Hydroxylysine concentration | 0.983 | 0.016 | -0.139 | 0.029 | -0.012 | 0.040 | -0.023 | 0.040 | -0.059 | -0.051 |
| Sum of amino acids concentration | 0.963 | 0.163 | 0.115 | 0.021 | 0.021 | -0.016 | -0.019 | 0.142 | -0.073 | -0.012 |
| TFAADs/N | 0.936 | 0.023 | 0.238 | 0.084 | 0.043 | -0.004 | -0.010 | 0.185 | -0.087 | -0.045 |
| Molar ratio of C/N in TFAADs | -0.874 | -0.399 | 0.156 | -0.079 | 0.074 | 0.034 | 0.103 | -0.047 | -0.036 | 0.074 |
| TFAADs/C | 0.972 | 0.082 | 0.119 | 0.033 | 0.030 | -0.004 | -0.018 | 0.141 | -0.081 | -0.013 |
| NR activity | 0.866 | 0.287 | -0.091 | -0.148 | 0.202 | 0.157 | -0.104 | 0.049 | 0.040 | -0.216 |
| GOGAT activity | 0.948 | 0.003 | -0.084 | -0.047 | -0.037 | 0.178 | -0.037 | -0.127 | -0.021 | -0.116 |
| GOT activity | 0.880 | 0.278 | -0.143 | -0.109 | -0.048 | -0.021 | -0.172 | 0.118 | -0.166 | 0.116 |
| GPT activity | 0.750 | 0.311 | -0.164 | 0.010 | 0.137 | -0.245 | 0.173 | -0.217 | 0.097 | 0.136 |
| Gsactivity | 0.458 | -0.688 | -0.353 | -0.148 | 0.078 | -0.076 | -0.156 | -0.170 | 0.207 | -0.065 |
| Starch concentration | -0.958 | 0.166 | 0.216 | -0.011 | -0.051 | -0.008 | -0.004 | 0.016 | -0.031 | 0.032 |
| TNC concentration | -0.580 | 0.262 | 0.462 | -0.272 | -0.323 | 0.268 | 0.113 | 0.233 | -0.077 | -0.020 |
| Total soluble sugars concentration | 0.960 | -0.084 | -0.048 | -0.124 | -0.098 | 0.148 | 0.063 | 0.098 | 0.002 | -0.052 |
| Glucose concentration | 0.949 | -0.138 | -0.176 | -0.062 | -0.010 | -0.075 | 0.000 | 0.131 | 0.019 | -0.099 |
| Fructose concentration | 0.940 | -0.119 | -0.193 | -0.053 | -0.114 | 0.162 | -0.034 | 0.028 | -0.016 | -0.065 |
| Sucrose concentration | 0.793 | 0.036 | 0.312 | -0.267 | -0.109 | 0.247 | 0.266 | 0.178 | 0.022 | 0.015 |
| Sucrose/starch | 0.946 | -0.054 | -0.118 | -0.025 | 0.111 | 0.026 | 0.145 | 0.022 | 0.152 | -0.015 |
| Malate concentration | 0.836 | 0.095 | 0.021 | -0.106 | -0.003 | 0.203 | 0.447 | -0.037 | -0.042 | -0.140 |
| Citrate concentration | -0.939 | 0.227 | 0.221 | -0.039 | -0.024 | 0.065 | -0.012 | 0.006 | -0.037 | -0.024 |
| Isocitrate concentration | 0.804 | 0.341 | 0.295 | -0.069 | -0.220 | 0.037 | -0.160 | 0.085 | 0.029 | -0.029 |
| Malate+citrate+isocitrate concentration | -0.723 | 0.420 | 0.362 | -0.126 | -0.065 | 0.215 | 0.224 | -0.003 | -0.073 | -0.119 |
| NADP-ME activity | 0.837 | 0.013 | -0.181 | -0.301 | 0.208 | 0.021 | 0.191 | 0.041 | 0.143 | 0.127 |
| NAD-ME activity | 0.843 | -0.260 | -0.222 | -0.027 | -0.051 | 0.335 | -0.004 | -0.188 | 0.020 | 0.009 |
| NADP-MDH activity | 0.875 | 0.002 | 0.117 | 0.015 | 0.015 | -0.256 | 0.123 | -0.135 | 0.052 | -0.116 |
| NAD-MDH activity | 0.892 | 0.074 | -0.042 | 0.087 | 0.226 | -0.192 | -0.176 | -0.093 | -0.137 | -0.139 |
| PEPC activity | 0.833 | 0.447 | 0.092 | 0.039 | -0.090 | 0.121 | 0.049 | 0.058 | 0.048 | 0.240 |
| PEPP activity | 0.749 | -0.126 | -0.353 | 0.033 | 0.129 | -0.127 | 0.290 | -0.363 | -0.010 | 0.057 |
| PK activity | 0.752 | 0.254 | -0.085 | -0.479 | -0.090 | 0.003 | 0.094 | 0.057 | -0.097 | 0.122 |
| CS activity | 0.781 | 0.369 | 0.058 | -0.343 | -0.050 | -0.023 | -0.279 | -0.084 | 0.054 | -0.136 |
| ACO activity | 0.908 | 0.088 | -0.048 | -0.062 | 0.099 | 0.331 | 0.040 | 0.032 | 0.064 | -0.030 |
| NADP-IDH activity | 0.921 | 0.016 | 0.044 | -0.219 | 0.140 | 0.151 | -0.017 | 0.186 | -0.064 | 0.074 |
|  |  |  |  |  |  |  |  |  |  |  |
| ***Eigen value*** | 71.196 | 11.783 | 5.357 | 3.372 | 2.501 | 2.267 | 1.980 | 1.375 | 1.210 | 1.104 |
| ***Variation percent (%)*** | 67.806 | 11.222 | 5.102 | 3.212 | 2.382 | 2.159 | 1.885 | 1.309 | 1.153 | 1.051 |
|  |  |  |  |  |  |  |  |  |  |  |
