# Supplementary material for: Adaptive responses of carbon and nitrogen metabolisms to nitrogen-deficiency in Citrus sinensis seedlings
Source: BMC Plant Biol. 2022 Jul 26;22:370. doi: 10.1186/s12870-022-03759-7 (PMC9316421; doi:10.1186/s12870-022-03759-7)
Supplement: Supplementary file 9 — Additional file 9: Table S4. PCA for 102 physiological parameters measured here in Citrus sinensis leaves. [file 12870_2022_3759_MOESM9_ESM.docx]

| **Additional file 9: Table S4.** PCA for 102 physiological parameters measured here in *Citrus sinensis* leaves | | | | | | | |  |  |
| --- | --- | --- | --- | --- | --- | --- | --- | --- | --- |
|  | Principal component | | | | | | | | |
|  | 1 | 2 | 3 | 4 | 5 | 6 | 7 | 8 | 9 |
| C concentration | 0.751 | 0.123 | 0.106 | -0.359 | -0.298 | -0.099 | 0.148 | 0.199 | 0.180 |
| N concentration | 0.984 | 0.086 | 0.004 | 0.007 | -0.092 | 0.009 | 0.005 | -0.010 | 0.054 |
| C/N | -0.991 | 0.092 | 0.016 | -0.016 | 0.035 | -0.038 | 0.015 | 0.022 | -0.025 |
| C distribution | 0.649 | -0.747 | -0.027 | -0.062 | -0.016 | 0.062 | -0.020 | 0.017 | -0.032 |
| N distribution | 0.672 | -0.683 | -0.073 | -0.125 | -0.148 | 0.037 | -0.043 | 0.045 | -0.089 |
| NH_4_^+^-N concentration | -0.737 | -0.198 | -0.051 | 0.281 | 0.048 | -0.245 | 0.400 | 0.061 | 0.205 |
| NO_3_^-^-N concentration | 0.551 | -0.159 | 0.384 | -0.347 | -0.417 | -0.339 | 0.258 | -0.001 | 0.122 |
| NH_4_^+^-N/NO_3_^-^-N | -0.939 | -0.099 | -0.106 | 0.208 | -0.014 | -0.086 | 0.115 | -0.063 | 0.104 |
| TSP concentration | 0.960 | 0.086 | -0.053 | 0.070 | -0.072 | -0.019 | 0.057 | -0.083 | 0.081 |
| Gly concentration | 0.907 | 0.385 | -0.114 | -0.042 | 0.057 | -0.029 | -0.033 | 0.080 | -0.001 |
| Ala concentration | 0.981 | -0.103 | 0.055 | -0.025 | 0.056 | 0.071 | 0.007 | 0.049 | 0.007 |
| Val concentration | -0.251 | 0.788 | 0.372 | 0.076 | -0.219 | 0.109 | -0.084 | -0.124 | -0.020 |
| Leu concentration | 0.744 | 0.560 | 0.153 | -0.065 | -0.192 | 0.086 | -0.087 | -0.172 | 0.067 |
| Met concentration | 0.977 | -0.023 | 0.160 | 0.055 | -0.006 | -0.014 | -0.003 | 0.020 | 0.066 |
| lle concentration | -0.522 | 0.819 | 0.185 | 0.050 | -0.079 | -0.012 | -0.006 | -0.007 | 0.059 |
| Pro concentration | 0.976 | -0.185 | 0.092 | 0.019 | 0.028 | 0.038 | -0.043 | -0.005 | 0.005 |
| Ser concentration | 0.994 | -0.019 | -0.034 | -0.009 | 0.007 | 0.014 | -0.041 | -0.020 | -0.041 |
| Trp concentration | -0.919 | 0.373 | 0.064 | -0.022 | -0.021 | -0.004 | -0.039 | -0.010 | -0.065 |
| Phe concentration | -0.513 | 0.738 | -0.052 | -0.054 | -0.321 | -0.089 | -0.026 | -0.146 | 0.179 |
| Tyr concentration | 0.952 | 0.194 | 0.060 | 0.014 | -0.185 | 0.042 | -0.063 | -0.010 | 0.036 |
| Glu concentration | 0.937 | -0.302 | 0.020 | 0.100 | -0.025 | -0.008 | -0.024 | -0.053 | -0.055 |
| Asp concentration | 0.986 | 0.020 | -0.023 | -0.076 | 0.039 | -0.038 | 0.047 | 0.059 | -0.058 |
| Asn concentration | 0.893 | 0.415 | -0.113 | -0.040 | 0.063 | -0.035 | -0.041 | 0.029 | 0.007 |
| Gln concentration | 0.910 | 0.406 | 0.028 | 0.052 | 0.023 | -0.018 | -0.006 | 0.021 | 0.016 |
| Lys concentration | 0.865 | 0.435 | -0.024 | 0.053 | -0.194 | -0.044 | -0.033 | -0.112 | -0.055 |
| Arg concentration | 0.894 | 0.414 | 0.093 | -0.038 | -0.061 | -0.025 | 0.001 | -0.015 | 0.041 |
| Thr concentration | 0.873 | 0.367 | 0.133 | 0.157 | -0.151 | 0.064 | 0.035 | -0.039 | -0.015 |
| L-Citrulline concentration | 0.844 | 0.522 | -0.062 | 0.031 | 0.006 | -0.052 | -0.025 | -0.030 | -0.002 |
| 5-Hydroxy-tryptamine concentration | -0.681 | -0.126 | -0.538 | -0.286 | 0.176 | -0.053 | 0.025 | -0.091 | 0.030 |
| L-Homocitrulline concentration | 0.804 | 0.424 | -0.018 | -0.252 | 0.218 | -0.097 | -0.016 | 0.154 | 0.015 |
| Beta-Alanine concentration | 0.976 | -0.038 | -0.105 | -0.085 | 0.054 | 0.032 | -0.087 | 0.040 | 0.045 |
| L-Pipecolic acid concentration | 0.748 | 0.521 | -0.037 | 0.118 | 0.073 | -0.059 | -0.071 | 0.074 | -0.140 |
| 3-N-Methyl-L-histidine concentration | 0.688 | 0.369 | 0.377 | 0.180 | -0.176 | -0.205 | -0.071 | 0.002 | -0.250 |
| Homoserine concentration | 0.689 | -0.359 | 0.494 | -0.106 | 0.167 | -0.047 | 0.020 | 0.073 | 0.107 |
| L-Cystathionine concentration | 0.940 | -0.001 | 0.182 | -0.021 | 0.116 | -0.156 | -0.039 | 0.058 | -0.088 |
| N6-Acetyl-L-Lysine concentration | 0.903 | 0.246 | -0.052 | -0.245 | 0.180 | 0.052 | -0.024 | -0.053 | 0.050 |
| Trans-4-hydroxy-L-proline | 0.929 | 0.254 | -0.237 | 0.029 | 0.076 | -0.010 | -0.047 | -0.008 | -0.057 |
| L-Ornithine concentration | 0.765 | 0.601 | -0.017 | 0.187 | -0.052 | -0.022 | 0.024 | -0.031 | -0.024 |
| L-tyrosine methyl ester concentration | 0.932 | 0.122 | 0.256 | -0.079 | 0.004 | -0.036 | -0.029 | 0.068 | -0.124 |
| N-Acetylaspartate concentration | 0.283 | -0.141 | 0.814 | 0.136 | -0.088 | -0.340 | 0.269 | 0.033 | -0.027 |
| (5-L-Glutamyl)-L-amino acid concentration | 0.974 | -0.150 | 0.086 | 0.008 | 0.023 | 0.073 | 0.027 | 0.109 | 0.022 |
| Glycyl-L-proline concentration | 0.773 | -0.535 | 0.229 | 0.002 | 0.081 | 0.071 | -0.040 | -0.152 | 0.047 |
| Trimethylamine N-oxide concentration | -0.949 | 0.076 | 0.019 | 0.006 | 0.012 | 0.209 | -0.100 | -0.127 | -0.004 |
| N8-Acetylspermidine concentration | -0.735 | -0.262 | -0.121 | -0.190 | 0.152 | 0.311 | 0.329 | 0.110 | 0.075 |
| Glutathione oxidized concentration | 0.938 | 0.258 | -0.032 | -0.047 | 0.062 | -0.007 | 0.135 | 0.116 | -0.098 |
| Methionine sulfoxide concentration | 0.965 | 0.174 | -0.099 | 0.013 | 0.037 | 0.017 | -0.047 | 0.017 | -0.028 |
| Asp-Phe concentration | 0.982 | 0.080 | -0.005 | 0.077 | 0.071 | 0.063 | -0.040 | -0.021 | -0.033 |
| Nα-Acetyl-L-arginine concentration | 0.913 | -0.097 | 0.050 | -0.059 | 0.147 | -0.054 | 0.094 | -0.218 | -0.125 |
| N-Glycyl-L-leucine concentration | 0.115 | 0.348 | 0.537 | -0.030 | 0.315 | 0.588 | -0.155 | -0.120 | -0.023 |
| γ-Glutamate-cysteine concentration | 0.632 | -0.464 | 0.216 | 0.294 | -0.041 | -0.028 | -0.310 | -0.125 | -0.022 |
| Nα-Acetyl-L-glutamine concentration | 0.864 | 0.469 | 0.061 | 0.113 | -0.068 | -0.016 | -0.008 | -0.034 | 0.028 |
| N-Acetyl-L-tyrosine concentration | 0.651 | -0.110 | 0.112 | 0.198 | 0.362 | -0.110 | 0.574 | 0.032 | -0.173 |
| D-Alanyl-D-alanine concentration | 0.847 | -0.514 | 0.043 | -0.013 | 0.016 | 0.078 | -0.007 | -0.046 | 0.012 |
| Homo-Arg concentration | 0.950 | 0.193 | 0.016 | 0.031 | -0.106 | 0.024 | 0.008 | 0.013 | 0.032 |
| L-Carnosine concentration | -0.296 | -0.201 | 0.628 | -0.254 | -0.187 | 0.432 | -0.116 | 0.289 | 0.053 |
| S-(5-Adenosy)-L-homocysteine concentration | 0.949 | -0.237 | 0.130 | -0.030 | -0.010 | -0.010 | 0.057 | -0.066 | -0.003 |
| Argininosuccinic-acid concentration | 0.730 | 0.508 | -0.155 | -0.219 | 0.109 | -0.059 | 0.121 | 0.105 | 0.021 |
| Succinic-acid concentration | -0.705 | -0.540 | 0.229 | 0.195 | 0.012 | -0.181 | 0.043 | 0.049 | -0.178 |
| 5-Aminovaleric-acid concentration | 0.794 | -0.245 | -0.129 | 0.008 | 0.024 | 0.096 | 0.259 | -0.199 | 0.327 |
| α-Aminoadipic-acid concentration | -0.142 | 0.658 | 0.308 | -0.023 | 0.259 | 0.255 | 0.217 | -0.355 | 0.337 |
| 2-Aminoethanesulfonic-acid concentration | 0.528 | 0.654 | -0.434 | -0.041 | 0.152 | 0.032 | 0.086 | -0.033 | -0.093 |
| γ-Aminobutyric-acid concentration | 0.949 | -0.156 | 0.144 | 0.060 | 0.044 | 0.112 | -0.063 | 0.086 | 0.031 |
| 4-Acetamidobutyric-acid concentration | 0.832 | -0.417 | 0.247 | -0.015 | 0.117 | -0.063 | 0.015 | -0.054 | 0.031 |
| 6-Aminocaproic-acid concentration | 0.951 | -0.291 | -0.028 | -0.053 | -0.009 | 0.054 | -0.020 | -0.044 | 0.012 |
| Creatine-phosphate concentration | -0.815 | 0.196 | 0.035 | -0.059 | 0.505 | 0.010 | -0.024 | 0.109 | -0.040 |
| Kynurenic-acid concentration | -0.818 | 0.527 | 0.057 | -0.055 | 0.126 | 0.084 | 0.016 | 0.014 | 0.038 |
| N'-Formylkynurenine concentration | 0.950 | 0.162 | 0.069 | 0.118 | -0.097 | 0.016 | 0.020 | -0.065 | -0.147 |
| 2-Aminobutyric-acid concentration | 0.853 | 0.173 | 0.094 | -0.064 | 0.063 | -0.036 | 0.140 | -0.339 | -0.258 |
| 3,7-Dimethyluric-acid concentration | 0.631 | 0.347 | 0.441 | 0.073 | 0.228 | 0.192 | 0.245 | 0.245 | 0.108 |
| Ethanolamine concentration | 0.859 | -0.433 | 0.096 | 0.010 | -0.029 | 0.204 | 0.025 | -0.076 | -0.073 |
| L-Cysteine concentration | -0.915 | 0.337 | 0.089 | -0.020 | 0.031 | 0.054 | -0.088 | -0.021 | -0.120 |
| Creatine concentration | -0.626 | 0.166 | -0.199 | 0.474 | -0.127 | 0.343 | 0.138 | 0.265 | -0.109 |
| Sum of amino acids concentration | 0.976 | 0.187 | -0.004 | -0.002 | 0.043 | -0.004 | 0.045 | 0.059 | -0.048 |
| TFAADs/N | 0.974 | 0.095 | -0.011 | -0.030 | 0.118 | -0.003 | 0.029 | 0.093 | -0.083 |
| Molar ratio of C/N in TFAADs | -0.971 | -0.185 | 0.037 | 0.113 | -0.066 | -0.021 | 0.034 | -0.027 | -0.033 |
| TFAADs/C | 0.978 | 0.171 | -0.017 | 0.009 | 0.039 | -0.005 | 0.041 | 0.054 | -0.058 |
| NR activity | 0.969 | 0.002 | -0.140 | 0.155 | -0.068 | 0.047 | 0.008 | -0.058 | -0.047 |
| GOGAT activity | 0.797 | -0.209 | -0.181 | -0.159 | -0.178 | 0.095 | -0.098 | 0.116 | 0.236 |
| GOT activity | 0.905 | 0.214 | 0.075 | -0.260 | 0.053 | -0.097 | 0.055 | -0.077 | 0.037 |
| GPT activity | 0.868 | 0.290 | -0.078 | -0.292 | 0.143 | -0.133 | -0.036 | 0.059 | 0.018 |
| GS activity | 0.960 | -0.183 | -0.112 | 0.076 | 0.077 | -0.068 | 0.004 | 0.082 | -0.053 |
| Starch concentration | 0.929 | -0.167 | -0.142 | -0.113 | 0.051 | -0.028 | -0.149 | -0.127 | 0.088 |
| TNC concentration | 0.946 | -0.137 | -0.138 | -0.040 | 0.048 | -0.031 | -0.137 | -0.104 | 0.106 |
| Total soluble sugars concentration | 0.853 | 0.079 | -0.083 | 0.435 | 0.018 | -0.043 | -0.036 | 0.066 | 0.197 |
| Glucose concentration | 0.914 | -0.371 | -0.004 | -0.010 | 0.026 | 0.088 | -0.057 | -0.078 | 0.016 |
| Fructose concentration | 0.938 | -0.050 | -0.070 | 0.063 | -0.077 | 0.182 | 0.057 | 0.141 | -0.120 |
| Sucrose concentration | 0.739 | 0.167 | -0.089 | 0.537 | 0.035 | -0.107 | -0.050 | 0.065 | 0.272 |
| Sucrose/starch | -0.934 | 0.313 | 0.074 | 0.104 | 0.002 | -0.006 | 0.053 | 0.047 | -0.022 |
| Malate concentration | 0.736 | -0.651 | 0.046 | -0.019 | 0.065 | -0.014 | -0.059 | -0.091 | 0.045 |
| Citrate concentration | -0.296 | 0.137 | 0.219 | 0.244 | 0.534 | -0.297 | -0.498 | 0.255 | 0.178 |
| Isocitrate concentration | 0.819 | 0.294 | -0.109 | -0.293 | 0.310 | -0.034 | 0.095 | 0.101 | -0.077 |
| Malate+citrate+isocitrate concentration | 0.711 | -0.604 | 0.096 | 0.027 | 0.219 | -0.092 | -0.182 | -0.021 | 0.087 |
| NADP-ME activity | 0.870 | 0.106 | -0.208 | -0.186 | -0.121 | -0.076 | -0.030 | 0.309 | 0.026 |
| NAD-ME activity | 0.951 | 0.060 | -0.101 | 0.095 | -0.064 | 0.079 | -0.040 | 0.180 | -0.005 |
| NADP-MDH activity | 0.864 | -0.006 | -0.052 | 0.361 | 0.012 | 0.065 | 0.011 | 0.114 | 0.295 |
| NAD-MDH activity | 0.930 | 0.081 | -0.182 | 0.192 | -0.181 | -0.002 | -0.049 | 0.095 | -0.016 |
| PEPC activity | 0.842 | -0.147 | -0.022 | -0.196 | -0.175 | 0.017 | -0.127 | -0.017 | -0.002 |
| PEPP activity | 0.683 | -0.217 | -0.179 | 0.135 | -0.241 | 0.406 | 0.219 | 0.200 | -0.123 |
| PK activity | 0.889 | -0.155 | -0.077 | 0.172 | 0.124 | 0.077 | -0.011 | -0.159 | -0.165 |
| CS activity | 0.837 | -0.239 | -0.087 | 0.179 | 0.186 | 0.063 | 0.185 | -0.168 | 0.051 |
| ACO activity | 0.819 | -0.161 | -0.304 | -0.022 | -0.077 | 0.105 | 0.030 | -0.015 | 0.125 |
| NADP-IDH activity | 0.874 | 0.055 | -0.314 | 0.209 | -0.031 | 0.003 | 0.022 | -0.139 | -0.049 |
|  |  |  |  |  |  |  |  |  |  |
| ***Eigen value*** | 71.017 | 11.872 | 4.114 | 2.651 | 2.366 | 1.903 | 1.738 | 1.380 | 1.200 |
| ***Variation percent (%)*** | 69.625 | 11.639 | 4.033 | 2.599 | 2.320 | 1.865 | 1.704 | 1.353 | 1.176 |
