# Supplementary material for: Adaptive responses of carbon and nitrogen metabolisms to nitrogen-deficiency in Citrus sinensis seedlings
Source: BMC Plant Biol. 2022 Jul 26;22:370. doi: 10.1186/s12870-022-03759-7 (PMC9316421; doi:10.1186/s12870-022-03759-7)
Supplement: Supplementary file 10 — Additional file 10: Table S5. Pearson correlation coefficient matrix between Citrus sinensis roots (first column) and leaves (first low) for the mean values of 101 physiological parameters. [file 12870_2022_3759_MOESM10_ESM.docx]

| **Additional file 10: Table S5.** Pearson correlation coefficient matrix between Citrus sinensis roots (first column) and leaves (first low) for the mean values of 101 physiological parameters | | | | | | | | | | | | | | | | | | |  |  |  |  |  |  |  |  |  |  |  |  |  |  |  |  |  |  |  |  |  |  |  |  |  |  |  |  |  |  |  |  |  |  |  |  |  |  |  |  |  |  |  |  |
| --- | --- | --- | --- | --- | --- | --- | --- | --- | --- | --- | --- | --- | --- | --- | --- | --- | --- | --- | --- | --- | --- | --- | --- | --- | --- | --- | --- | --- | --- | --- | --- | --- | --- | --- | --- | --- | --- | --- | --- | --- | --- | --- | --- | --- | --- | --- | --- | --- | --- | --- | --- | --- | --- | --- | --- | --- | --- | --- | --- | --- | --- | --- |
|  | Leaf C | Leaf N | Leaf C/N | Leaf NO_3_^-^-N | Leaf NH_4_^+^-N/NO_3_^-^-N | Leaf TSPs | Leaf Gly | Leaf Ala | Leaf Pro | Leaf Ser | Leaf Glu | Leaf Asp | Leaf Asn | Leaf Gln | Leaf Arg | Leaf L-citrulline | Leaf beta-alanine | Leaf L-pipecolic acid | Leaf homoserine | Leaf trans-4-hydroxy-L-proline | Leaf L-ornithine | Leaf (5-L-glutamyl)-L-amino acid | Leaf trimethylamine-N-oxide | Leaf glutathione-oxidized | Leaf γ-glutamate-cysteine | Leaf Nα-acetyl-L-glutamine | Leaf N-acetyl-L-tyrosine | Leaf homo-Arg | Leaf S-(5-adenosy)-L-homocysteine | Leaf γ-aminobutyric acid | Leaf N'-formylkynurenine | Leaf ethanolamine | Leaf TFAADs | Leaf TFAADs/N | Leaf molar ratio of C/N in TFAADs | Leaf TFAADs/C | Leaf NR | Leaf GOGAT | Leaf GOT | Leaf GPT | Leaf glucose | Leaf fructose | Leaf sucrose | Leaf total soluble sugars | Leaf isocitrate | Leaf NADP-ME | Leaf NADP-MDH | Leaf NAD-MDH | Leaf PEPC | Leaf PEPP | Leaf PK | Leaf CS | Leaf ACO | Leaf NADP-IDH | C distribution in leaves | N distribution in leaves | Leaf Ile | Leaf creatine-phosphate | Leaf starch | Leaf TNC | Leaf sucrose/starch | Leaf malate + citrate + isocitrateLeaf NH_4_^+^-N Leaf Leu Leaf Val Leaf Met Leaf Trp Leaf Phe Leaf Tyr Leaf Lys Leaf Thr Leaf 5-hydroxy-tryptamine Leaf L-homocitrulline Leaf 3-N-methyl-L-histidine Leaf L-cystathionine Leaf N6-acetyl-L-lysine Leaf L-tyrosine-methyl-ester Leaf N-acetylaspartate Leaf glycyl-L-proline Leaf N8-acetylspermidine Leaf methionine-sulfoxide Leaf Asp-Phe Leaf Nα-acetyl-L-arginine Leaf N-glycyl-L-leucine Leaf D-alanyl-D-alanine Leaf L-carnosine Leaf argininosuccinic acid Leaf succinic acid Leaf 5-aminovaleric acid Leaf α-aminoadipic acid Leaf 2-aminobutyric acid Leaf 4-acetamidobutyric acid Leaf 6-aminocaproic acid Leaf kynurenic acid Leaf 2-aminoethanesulfonic-acid Leaf Cys Leaf creatine Leaf GS Leaf malate Leaf citrate Leaf NAD-ME |
| Root C | 0.911 | 0.889 | ##### | 0.727 | -0.874 | 0.894 | 0.988 | 0.807 | 0.757 | 0.848 | 0.654 | 0.888 | 0.991 | 0.979 | 0.974 | 0.979 | 0.847 | 0.951 | 0.562 | 0.935 | 0.918 | 0.786 | ##### | 0.970 | 0.367 | 0.957 | 0.815 | 0.914 | #### | 0.762 | 0.879 | 0.516 | 0.943 | 0.917 | -0.948 | 0.936 | 0.804 | 0.644 | 0.965 | 0.973 | 0.619 | 0.785 | 0.714 | 0.726 | 0.964 | 0.897 | 0.800 | 0.796 | 0.810 | 0.726 | 0.725 | 0.753 | 0.729 | 0.848 | 0.242 | 0.316 | ##### | -0.632 | 0.753 | 0.755 | -0.686 | 0.259 -0.960 0.925 0.094 0.841 ##### -0.130 0.907 0.933 0.885 -0.696 0.970 0.928 0.896 0.960 0.932 0.365 0.414 ##### 0.921 0.889 0.808 0.431 0.482 -0.473 0.990 -0.988 0.671 0.229 0.921 0.580 0.684 -0.469 0.838 -0.659 -0.712 0.750 0.227 -0.567 0.862 |
| Root N | 0.932 | 0.994 | ##### | 0.916 | -0.996 | 0.980 | 0.926 | 0.988 | 0.972 | 0.991 | 0.921 | 0.995 | 0.921 | 0.927 | 0.918 | 0.868 | 0.991 | 0.791 | 0.828 | 0.945 | 0.780 | 0.981 | ##### | 0.969 | 0.743 | 0.883 | 0.988 | 0.976 | #### | 0.974 | 0.957 | 0.851 | 0.986 | 0.991 | -0.987 | 0.987 | 0.953 | 0.911 | 0.964 | 0.909 | 0.907 | 0.977 | 0.895 | 0.927 | 0.889 | 0.976 | 0.968 | 0.946 | 0.979 | 0.930 | 0.954 | 0.971 | 0.935 | 0.966 | 0.648 | 0.705 | ##### | -0.876 | 0.965 | 0.967 | -0.940 | 0.667 -0.973 0.809 ##### 0.991 ##### -0.538 0.972 0.880 0.882 -0.787 0.840 0.837 0.992 0.963 0.992 0.534 0.779 ##### 0.975 0.995 0.982 0.277 0.829 -0.386 0.840 -0.843 0.932 ##### 0.978 0.878 0.941 -0.820 0.590 -0.929 -0.876 0.964 0.645 -0.702 0.991 |
| Root C/N | -0.859 | ##### | 0.994 | ##### | 0.973 | -0.937 | ##### | ##### | -1.000 | ##### | -0.982 | -0.969 | -0.808 | -0.817 | -0.804 | ##### | -0.984 | -0.648 | -0.896 | -0.873 | -0.634 | ##### | 0.984 | -0.886 | -0.876 | ##### | -0.991 | -0.920 | #### | ##### | -0.910 | ##### | -0.925 | -0.949 | 0.925 | -0.929 | -0.947 | -0.977 | ##### | -0.806 | -0.980 | -0.992 | ##### | -0.950 | -0.783 | -0.940 | -0.966 | -0.942 | -0.988 | -0.955 | ##### | -0.999 | -0.962 | -0.943 | -0.807 | ##### | 0.678 | 0.918 | -0.996 | -0.997 | 0.993 | -0.823 0.899 -0.668 0.454 ##### 0.983 0.711 -0.916 -0.770 ##### 0.742 -0.709 -0.712 -0.960 ##### -0.937 -0.547 ##### 0.808 -0.923 -0.964 -0.991 -0.151 ##### 0.321 -0.688 0.694 -0.990 0.358 -0.920 -0.959 -0.994 0.931 -0.423 0.990 0.895 -0.995 -0.806 0.684 -0.970 |
| Root NO_3_^-^-N | 0.935 | 0.995 | ##### | 0.916 | -0.976 | 0.990 | 0.935 | 0.959 | 0.938 | 0.967 | 0.876 | 0.968 | 0.939 | 0.962 | 0.961 | 0.915 | 0.954 | 0.812 | 0.756 | 0.939 | 0.858 | 0.947 | ##### | 0.973 | 0.670 | 0.938 | 0.960 | 0.991 | #### | 0.940 | 0.976 | 0.801 | 0.987 | 0.968 | -0.978 | 0.985 | 0.947 | 0.844 | 0.966 | 0.891 | 0.848 | 0.951 | 0.890 | 0.911 | 0.866 | 0.942 | 0.969 | 0.941 | 0.936 | 0.910 | 0.919 | 0.939 | 0.909 | 0.965 | 0.548 | 0.610 | ##### | -0.880 | 0.912 | 0.919 | -0.884 | 0.577 -0.962 0.891 ##### 0.984 ##### -0.417 0.995 0.933 0.949 -0.865 0.830 0.895 0.960 0.952 0.985 0.598 0.722 ##### 0.968 0.982 0.942 0.371 0.763 -0.389 0.882 -0.886 0.876 ##### 0.994 0.806 0.892 -0.760 0.616 -0.877 -0.796 0.919 0.558 -0.792 0.984 |
| Root NH_4_^+^-N/NO_3_^-^-N | -0.859 | ##### | 0.984 | ##### | 0.964 | -0.919 | ##### | ##### | -0.996 | ##### | -0.974 | -0.945 | -0.769 | -0.790 | -0.786 | ##### | -0.963 | -0.585 | -0.917 | -0.828 | -0.603 | ##### | 0.970 | -0.855 | -0.880 | ##### | -0.988 | -0.900 | #### | ##### | -0.889 | ##### | -0.899 | -0.920 | 0.900 | -0.903 | -0.924 | -0.964 | ##### | -0.766 | -0.978 | -0.980 | ##### | -0.931 | -0.742 | -0.902 | -0.958 | -0.918 | -0.966 | -0.937 | ##### | -0.996 | -0.942 | -0.919 | -0.824 | ##### | 0.681 | 0.921 | -0.984 | -0.985 | 0.989 | -0.843 0.874 -0.657 0.447 ##### 0.982 0.729 -0.902 -0.741 ##### 0.782 -0.667 -0.676 -0.938 ##### -0.921 -0.627 ##### 0.785 -0.888 -0.943 -0.971 -0.207 ##### 0.234 -0.658 0.656 -0.984 0.343 -0.901 -0.967 -0.991 0.940 -0.343 0.988 0.889 -0.981 -0.829 0.728 -0.955 |
| Root TSPs | 0.899 | 0.962 | ##### | 0.829 | -0.916 | 0.974 | 0.961 | 0.884 | 0.850 | 0.917 | 0.781 | 0.921 | 0.971 | 0.992 | 0.989 | 0.973 | 0.892 | 0.888 | 0.608 | 0.948 | 0.946 | 0.864 | ##### | 0.975 | 0.533 | 0.989 | 0.882 | 0.984 | #### | 0.853 | 0.973 | 0.672 | 0.973 | 0.934 | -0.955 | 0.971 | 0.915 | 0.735 | 0.950 | 0.891 | 0.734 | 0.884 | 0.860 | 0.862 | 0.865 | 0.908 | 0.925 | 0.911 | 0.867 | 0.858 | 0.840 | 0.855 | 0.852 | 0.942 | 0.372 | 0.445 | ##### | -0.824 | 0.818 | 0.830 | -0.776 | 0.407 -0.941 0.956 0.073 0.927 ##### -0.227 0.987 0.985 0.983 -0.849 0.850 0.965 0.908 0.930 0.951 0.525 0.569 ##### 0.957 0.947 0.872 0.395 0.621 -0.487 0.936 -0.950 0.769 0.099 0.986 0.669 0.788 -0.628 0.732 -0.769 -0.679 0.836 0.387 -0.781 0.945 |
| Root Gly | 0.937 | 0.959 | ##### | 0.915 | -0.992 | 0.930 | 0.888 | 0.981 | 0.969 | 0.969 | 0.911 | 0.986 | 0.878 | 0.871 | 0.864 | 0.800 | 0.987 | 0.735 | 0.893 | 0.902 | 0.681 | 0.981 | ##### | 0.935 | 0.752 | 0.806 | 0.987 | 0.923 | #### | 0.971 | 0.892 | 0.858 | 0.954 | 0.979 | -0.971 | 0.953 | 0.901 | 0.925 | 0.952 | 0.914 | 0.916 | 0.956 | 0.830 | 0.879 | 0.901 | 0.964 | 0.921 | 0.892 | 0.976 | 0.887 | 0.937 | 0.962 | 0.897 | 0.915 | 0.704 | 0.752 | ##### | -0.814 | 0.977 | 0.972 | -0.954 | 0.712 -0.966 0.726 ##### 0.969 ##### -0.621 0.916 0.793 0.792 -0.716 0.847 0.744 0.995 0.962 0.978 0.512 0.806 ##### 0.938 0.966 0.976 0.271 0.852 -0.292 0.797 -0.785 0.942 ##### 0.928 0.916 0.946 -0.828 0.527 -0.935 -0.947 0.960 0.686 -0.607 0.956 |
| Root Ala | 0.811 | 0.821 | ##### | 0.794 | -0.895 | 0.777 | 0.746 | 0.898 | 0.899 | 0.875 | 0.865 | 0.901 | 0.721 | 0.683 | 0.664 | 0.598 | 0.919 | 0.589 | 0.916 | 0.783 | 0.433 | 0.910 | ##### | 0.796 | 0.777 | 0.585 | 0.905 | 0.761 | #### | 0.899 | 0.723 | 0.829 | 0.819 | 0.887 | -0.856 | 0.822 | 0.778 | 0.921 | 0.822 | 0.829 | 0.896 | 0.868 | 0.708 | 0.775 | 0.828 | 0.893 | 0.783 | 0.768 | 0.920 | 0.790 | 0.866 | 0.886 | 0.810 | 0.781 | 0.794 | 0.826 | ##### | -0.680 | 0.942 | 0.928 | -0.926 | 0.781 -0.858 0.470 ##### 0.846 ##### -0.767 0.740 0.575 0.549 -0.469 0.757 0.522 0.920 0.856 0.859 0.328 0.803 ##### 0.817 0.852 0.913 0.076 0.856 -0.220 0.611 -0.594 0.915 ##### 0.766 0.927 0.905 -0.820 0.399 -0.901 -0.979 0.909 0.752 -0.345 0.831 |
| Root Pro | 0.928 | 0.972 | ##### | 0.925 | -0.996 | 0.947 | 0.886 | 0.992 | 0.982 | 0.981 | 0.933 | 0.990 | 0.878 | 0.877 | 0.869 | 0.805 | 0.993 | 0.732 | 0.888 | 0.909 | 0.695 | 0.991 | ##### | 0.937 | 0.779 | 0.817 | 0.995 | 0.938 | #### | 0.983 | 0.913 | 0.880 | 0.960 | 0.981 | -0.971 | 0.961 | 0.925 | 0.938 | 0.947 | 0.897 | 0.933 | 0.973 | 0.863 | 0.907 | 0.881 | 0.967 | 0.945 | 0.917 | 0.985 | 0.913 | 0.956 | 0.977 | 0.922 | 0.936 | 0.718 | 0.767 | ##### | -0.852 | 0.984 | 0.982 | -0.965 | 0.730 -0.961 0.736 ##### 0.982 ##### -0.627 0.933 0.810 0.816 -0.743 0.823 0.760 0.993 0.953 0.979 0.531 0.825 ##### 0.947 0.977 0.985 0.255 0.870 -0.313 0.789 -0.783 0.955 ##### 0.942 0.923 0.960 -0.852 0.519 -0.950 -0.929 0.974 0.707 -0.647 0.971 |
| Root Ser | 0.885 | 0.949 | ##### | 0.901 | -0.979 | 0.921 | 0.847 | 0.988 | 0.985 | 0.975 | 0.952 | 0.980 | 0.833 | 0.825 | 0.810 | 0.746 | 0.991 | 0.688 | 0.906 | 0.888 | 0.625 | 0.990 | ##### | 0.904 | 0.830 | 0.757 | 0.987 | 0.907 | #### | 0.985 | 0.886 | 0.906 | 0.932 | 0.966 | -0.945 | 0.935 | 0.919 | 0.967 | 0.907 | 0.865 | 0.958 | 0.974 | 0.864 | 0.911 | 0.850 | 0.961 | 0.931 | 0.911 | 0.990 | 0.920 | 0.966 | 0.980 | 0.930 | 0.922 | 0.779 | 0.824 | ##### | -0.851 | 0.996 | 0.993 | -0.982 | 0.787 -0.931 0.658 ##### 0.963 ##### -0.700 0.897 0.757 0.757 -0.680 0.782 0.703 0.982 0.921 0.951 0.479 0.855 ##### 0.928 0.962 0.990 0.160 0.904 -0.319 0.723 -0.722 0.976 ##### 0.910 0.951 0.976 -0.888 0.477 -0.970 -0.941 0.985 0.764 -0.590 0.956 |
| Root Glu | 0.913 | 0.976 | ##### | 0.947 | -0.993 | 0.952 | 0.858 | 0.999 | 0.994 | 0.983 | 0.953 | 0.981 | 0.852 | 0.863 | 0.857 | 0.785 | 0.988 | 0.691 | 0.895 | 0.891 | 0.685 | 0.998 | ##### | 0.920 | 0.815 | 0.809 | 1.000 | 0.940 | #### | 0.995 | 0.921 | 0.915 | 0.950 | 0.967 | -0.955 | 0.952 | 0.938 | 0.948 | 0.927 | 0.855 | 0.952 | 0.984 | 0.885 | 0.927 | 0.835 | 0.949 | 0.963 | 0.931 | 0.983 | 0.932 | 0.972 | 0.992 | 0.938 | 0.943 | 0.749 | 0.796 | ##### | -0.892 | 0.987 | 0.987 | -0.976 | 0.765 -0.938 0.731 ##### 0.992 ##### -0.650 0.939 0.806 0.828 -0.782 0.772 0.751 0.979 0.929 0.968 0.584 0.864 ##### 0.937 0.975 0.986 0.254 0.899 -0.293 0.757 -0.754 0.969 ##### 0.943 0.938 0.976 -0.886 0.466 -0.968 -0.909 0.981 0.746 -0.704 0.977 |
| Root Asp | 0.818 | 0.941 | ##### | 0.945 | -0.919 | 0.931 | 0.754 | 0.949 | 0.954 | 0.928 | 0.937 | 0.893 | 0.759 | 0.809 | 0.812 | 0.729 | 0.904 | 0.576 | 0.807 | 0.805 | 0.687 | 0.945 | ##### | 0.834 | 0.830 | 0.790 | 0.942 | 0.915 | #### | 0.955 | 0.920 | 0.928 | 0.881 | 0.869 | -0.860 | 0.884 | 0.933 | 0.888 | 0.825 | 0.687 | 0.919 | 0.953 | 0.915 | 0.938 | 0.652 | 0.840 | 0.974 | 0.930 | 0.905 | 0.937 | 0.951 | 0.961 | 0.932 | 0.927 | 0.725 | 0.768 | ##### | -0.969 | 0.912 | 0.922 | -0.922 | 0.761 -0.822 0.737 ##### 0.964 ##### -0.594 0.928 0.801 0.871 -0.890 0.586 0.741 0.872 0.805 0.885 0.716 0.879 ##### 0.867 0.919 0.916 0.269 0.889 -0.264 0.662 -0.672 0.921 ##### 0.913 0.878 0.938 -0.897 0.336 -0.933 -0.744 0.928 0.756 -0.878 0.946 |
| Root Asn | 0.958 | 0.975 | ##### | 0.870 | -0.975 | 0.966 | 0.974 | 0.939 | 0.909 | 0.955 | 0.831 | 0.977 | 0.973 | 0.971 | 0.966 | 0.934 | 0.957 | 0.873 | 0.751 | 0.960 | 0.852 | 0.928 | ##### | 0.994 | 0.601 | 0.933 | 0.945 | 0.972 | #### | 0.912 | 0.943 | 0.734 | 0.993 | 0.987 | -0.999 | 0.990 | 0.908 | 0.822 | 0.994 | 0.964 | 0.810 | 0.920 | 0.831 | 0.859 | 0.949 | 0.967 | 0.919 | 0.900 | 0.933 | 0.861 | 0.880 | 0.905 | 0.866 | 0.936 | 0.498 | 0.563 | ##### | -0.789 | 0.903 | 0.905 | -0.860 | 0.516 -0.997 0.878 ##### 0.955 ##### -0.387 0.967 0.918 0.902 -0.772 0.922 0.888 0.981 0.992 0.996 0.490 0.652 ##### 0.974 0.975 0.934 0.368 0.707 -0.409 0.927 -0.924 0.848 0.011 0.977 0.781 0.859 -0.694 0.700 -0.840 -0.842 0.899 0.489 -0.662 0.960 |
| Root Gln | 0.917 | 0.927 | ##### | 0.788 | -0.894 | 0.936 | 0.978 | 0.839 | 0.795 | 0.874 | 0.701 | 0.897 | 0.987 | 0.998 | 0.998 | 0.991 | 0.859 | 0.922 | 0.574 | 0.936 | 0.954 | 0.818 | ##### | 0.975 | 0.424 | 0.991 | 0.845 | 0.953 | #### | 0.800 | 0.929 | 0.580 | 0.959 | 0.919 | -0.952 | 0.953 | 0.853 | 0.668 | 0.964 | 0.931 | 0.658 | 0.826 | 0.778 | 0.784 | 0.913 | 0.891 | 0.862 | 0.847 | 0.825 | 0.780 | 0.770 | 0.796 | 0.778 | 0.891 | 0.278 | 0.352 | ##### | -0.725 | 0.771 | 0.778 | -0.716 | 0.307 -0.951 0.969 0.134 0.885 ##### -0.144 0.955 0.973 0.955 -0.805 0.913 0.961 0.895 0.947 0.944 0.481 0.479 ##### 0.934 0.914 0.828 0.463 0.533 -0.460 0.981 -0.984 0.703 0.214 0.959 0.606 0.722 -0.530 0.784 -0.699 -0.681 0.778 0.281 -0.706 0.901 |
| Root Arg | 0.940 | 0.926 | ##### | 0.786 | -0.926 | 0.921 | 0.990 | 0.870 | 0.829 | 0.899 | 0.735 | 0.938 | 0.988 | 0.973 | 0.966 | 0.955 | 0.907 | 0.924 | 0.664 | 0.953 | 0.873 | 0.855 | ##### | 0.986 | 0.474 | 0.936 | 0.879 | 0.935 | #### | 0.833 | 0.898 | 0.612 | 0.969 | 0.959 | -0.979 | 0.963 | 0.845 | 0.735 | 0.986 | 0.990 | 0.710 | 0.847 | 0.756 | 0.779 | 0.982 | 0.942 | 0.845 | 0.836 | 0.875 | 0.782 | 0.796 | 0.823 | 0.788 | 0.883 | 0.369 | 0.438 | ##### | -0.684 | 0.832 | 0.831 | -0.773 | 0.382 -0.988 0.887 ##### 0.890 ##### -0.266 0.926 0.914 0.872 -0.699 0.972 0.898 0.947 0.988 0.967 0.391 0.521 ##### 0.949 0.930 0.873 0.388 0.588 -0.443 0.965 -0.960 0.758 0.118 0.942 0.682 0.768 -0.570 0.787 -0.746 -0.799 0.823 0.350 -0.568 0.904 |
| Root L-citrulline | 0.937 | 0.966 | ##### | 0.829 | -0.953 | 0.965 | 0.990 | 0.914 | 0.880 | 0.942 | 0.803 | 0.965 | 0.989 | 0.985 | 0.976 | 0.959 | 0.940 | 0.911 | 0.691 | 0.974 | 0.887 | 0.899 | ##### | 0.999 | 0.559 | 0.954 | 0.917 | 0.974 | #### | 0.882 | 0.948 | 0.688 | 0.992 | 0.979 | -0.993 | 0.989 | 0.906 | 0.791 | 0.987 | 0.968 | 0.773 | 0.901 | 0.832 | 0.851 | 0.952 | 0.963 | 0.907 | 0.899 | 0.915 | 0.852 | 0.858 | 0.877 | 0.855 | 0.935 | 0.437 | 0.507 | ##### | -0.774 | 0.873 | 0.877 | -0.824 | 0.457 -0.991 0.898 ##### 0.934 ##### -0.321 0.967 0.942 0.914 -0.754 0.932 0.921 0.965 0.985 0.985 0.440 0.593 ##### 0.978 0.967 0.915 0.349 0.658 -0.472 0.946 -0.951 0.814 0.042 0.978 0.732 0.825 -0.650 0.757 -0.805 -0.798 0.875 0.429 -0.647 0.951 |
| Root β-Ala | 0.886 | 0.932 | ##### | 0.901 | -0.973 | 0.899 | 0.829 | 0.979 | 0.978 | 0.960 | 0.940 | 0.970 | 0.814 | 0.803 | 0.791 | 0.720 | 0.982 | 0.663 | 0.924 | 0.866 | 0.591 | 0.984 | ##### | 0.887 | 0.823 | 0.729 | 0.982 | 0.885 | #### | 0.978 | 0.858 | 0.902 | 0.916 | 0.955 | -0.934 | 0.918 | 0.893 | 0.962 | 0.901 | 0.862 | 0.953 | 0.959 | 0.833 | 0.886 | 0.849 | 0.948 | 0.910 | 0.885 | 0.981 | 0.896 | 0.952 | 0.970 | 0.909 | 0.897 | 0.790 | 0.831 | ##### | -0.824 | 0.992 | 0.987 | -0.979 | 0.794 -0.924 0.632 ##### 0.952 ##### -0.720 0.875 0.724 0.726 -0.661 0.780 0.669 0.976 0.917 0.942 0.482 0.860 ##### 0.908 0.946 0.979 0.173 0.905 -0.274 0.708 -0.700 0.971 ##### 0.888 0.957 0.970 -0.882 0.448 -0.964 -0.960 0.975 0.770 -0.563 0.938 |
| Root L-pipecolic acid | 0.957 | 0.972 | ##### | 0.866 | -0.962 | 0.967 | 0.977 | 0.922 | 0.889 | 0.940 | 0.805 | 0.961 | 0.979 | 0.986 | 0.984 | 0.954 | 0.936 | 0.881 | 0.713 | 0.954 | 0.887 | 0.908 | ##### | 0.993 | 0.562 | 0.957 | 0.928 | 0.976 | #### | 0.892 | 0.948 | 0.706 | 0.990 | 0.972 | -0.991 | 0.986 | 0.901 | 0.786 | 0.993 | 0.954 | 0.778 | 0.904 | 0.825 | 0.847 | 0.937 | 0.947 | 0.915 | 0.894 | 0.909 | 0.848 | 0.860 | 0.886 | 0.850 | 0.931 | 0.447 | 0.514 | ##### | -0.787 | 0.874 | 0.877 | -0.829 | 0.469 -0.989 0.915 ##### 0.949 ##### -0.326 0.975 0.941 0.931 -0.807 0.917 0.914 0.962 0.985 0.990 0.518 0.620 ##### 0.967 0.965 0.911 0.413 0.672 -0.409 0.946 -0.943 0.817 0.071 0.982 0.743 0.831 -0.661 0.711 -0.812 -0.801 0.873 0.443 -0.701 0.954 |
| Root homoserine | 0.871 | 0.897 | ##### | 0.939 | -0.952 | 0.853 | 0.741 | 0.966 | 0.973 | 0.922 | 0.935 | 0.925 | 0.728 | 0.734 | 0.733 | 0.633 | 0.946 | 0.536 | 0.973 | 0.778 | 0.503 | 0.975 | ##### | 0.818 | 0.850 | 0.658 | 0.974 | 0.834 | #### | 0.974 | 0.805 | 0.939 | 0.859 | 0.900 | -0.881 | 0.860 | 0.848 | 0.952 | 0.854 | 0.783 | 0.960 | 0.936 | 0.790 | 0.853 | 0.769 | 0.882 | 0.887 | 0.838 | 0.945 | 0.863 | 0.937 | 0.965 | 0.875 | 0.847 | 0.848 | 0.877 | ##### | -0.822 | 0.979 | 0.971 | -0.981 | 0.853 -0.870 0.576 ##### 0.940 ##### -0.784 0.833 0.647 0.682 -0.697 0.694 0.578 0.935 0.866 0.903 0.595 0.924 ##### 0.840 0.900 0.947 0.230 0.943 -0.122 0.629 -0.607 0.969 ##### 0.839 0.984 0.972 -0.912 0.292 -0.968 -0.961 0.955 0.835 -0.607 0.902 |
| Root trans-4-hydroxy-L-proline | 0.913 | 0.961 | ##### | 0.922 | -0.990 | 0.933 | 0.864 | 0.991 | 0.985 | 0.977 | 0.941 | 0.984 | 0.853 | 0.850 | 0.841 | 0.773 | 0.992 | 0.702 | 0.906 | 0.893 | 0.657 | 0.993 | ##### | 0.919 | 0.803 | 0.786 | 0.994 | 0.921 | #### | 0.986 | 0.897 | 0.896 | 0.945 | 0.972 | -0.958 | 0.946 | 0.918 | 0.951 | 0.930 | 0.881 | 0.946 | 0.972 | 0.857 | 0.905 | 0.865 | 0.960 | 0.937 | 0.910 | 0.986 | 0.912 | 0.960 | 0.980 | 0.922 | 0.925 | 0.751 | 0.798 | ##### | -0.850 | 0.991 | 0.988 | -0.975 | 0.761 -0.947 0.699 ##### 0.975 ##### -0.667 0.915 0.780 0.786 -0.720 0.803 0.727 0.988 0.939 0.967 0.522 0.846 ##### 0.934 0.968 0.986 0.225 0.890 -0.296 0.757 -0.750 0.966 ##### 0.925 0.941 0.969 -0.871 0.486 -0.961 -0.941 0.978 0.738 -0.626 0.963 |
| Root L-ornithine | 0.928 | 0.928 | ##### | 0.774 | -0.917 | 0.929 | 0.995 | 0.863 | 0.821 | 0.899 | 0.730 | 0.933 | 0.995 | 0.983 | 0.975 | 0.970 | 0.900 | 0.939 | 0.632 | 0.961 | 0.899 | 0.846 | ##### | 0.989 | 0.464 | 0.954 | 0.869 | 0.944 | #### | 0.824 | 0.912 | 0.601 | 0.971 | 0.955 | -0.976 | 0.966 | 0.855 | 0.723 | 0.981 | 0.982 | 0.699 | 0.845 | 0.771 | 0.788 | 0.971 | 0.939 | 0.851 | 0.847 | 0.869 | 0.789 | 0.793 | 0.817 | 0.793 | 0.892 | 0.344 | 0.416 | ##### | -0.696 | 0.819 | 0.821 | -0.759 | 0.360 -0.982 0.906 ##### 0.887 ##### -0.232 0.935 0.936 0.893 -0.710 0.963 0.923 0.938 0.979 0.963 0.385 0.502 ##### 0.954 0.931 0.867 0.380 0.571 -0.479 0.972 -0.973 0.747 0.129 0.950 0.660 0.757 -0.560 0.808 -0.735 -0.767 0.816 0.329 -0.588 0.907 |
| Root (5-L-glutamyl)-L-amino acid | 0.734 | 0.803 | ##### | 0.797 | -0.868 | 0.760 | 0.661 | 0.902 | 0.918 | 0.870 | 0.918 | 0.873 | 0.635 | 0.609 | 0.586 | 0.508 | 0.906 | 0.483 | 0.932 | 0.733 | 0.353 | 0.917 | ##### | 0.732 | 0.887 | 0.514 | 0.900 | 0.734 | #### | 0.916 | 0.713 | 0.908 | 0.774 | 0.846 | -0.800 | 0.781 | 0.795 | 0.964 | 0.744 | 0.722 | 0.950 | 0.887 | 0.753 | 0.817 | 0.714 | 0.857 | 0.802 | 0.788 | 0.921 | 0.831 | 0.902 | 0.909 | 0.849 | 0.783 | 0.900 | 0.924 | ##### | -0.750 | 0.954 | 0.944 | -0.958 | 0.891 -0.786 0.386 ##### 0.841 ##### -0.869 0.715 0.520 0.516 -0.469 0.624 0.457 0.880 0.776 0.808 0.356 0.889 ##### 0.783 0.833 0.916 -0.032 0.934 -0.210 0.491 -0.485 0.954 ##### 0.735 0.971 0.943 -0.910 0.276 -0.945 -0.945 0.931 0.870 -0.386 0.825 |
| Root trimethylamine-N-oxide | -0.946 | ##### | 0.943 | ##### | 0.981 | -0.941 | ##### | ##### | -0.930 | ##### | -0.861 | -0.987 | -0.938 | -0.923 | -0.912 | ##### | -0.977 | -0.831 | -0.815 | -0.945 | -0.763 | ##### | 0.977 | -0.973 | -0.661 | ##### | -0.960 | -0.942 | #### | ##### | -0.908 | ##### | -0.977 | -0.992 | 0.992 | -0.975 | -0.897 | -0.873 | ##### | -0.965 | -0.854 | -0.931 | ##### | -0.858 | -0.955 | -0.981 | -0.904 | -0.888 | -0.959 | -0.864 | ##### | -0.923 | -0.874 | -0.920 | -0.588 | ##### | 0.457 | 0.773 | -0.940 | -0.937 | 0.901 | -0.597 0.993 -0.789 0.275 ##### 0.861 0.499 -0.931 -0.851 ##### 0.696 -0.916 -0.815 -0.996 ##### -0.989 -0.439 ##### 0.861 -0.963 -0.970 -0.957 -0.289 ##### 0.381 -0.873 0.866 -0.889 0.097 -0.947 -0.841 -0.893 0.742 -0.653 0.878 0.909 -0.927 -0.567 0.575 -0.951 |
| Root glutathione-oxidized | 0.963 | 0.970 | ##### | 0.927 | -0.997 | 0.945 | 0.911 | 0.976 | 0.959 | 0.965 | 0.888 | 0.985 | 0.906 | 0.906 | 0.904 | 0.842 | 0.980 | 0.763 | 0.870 | 0.911 | 0.734 | 0.973 | ##### | 0.954 | 0.706 | 0.849 | 0.985 | 0.941 | #### | 0.961 | 0.908 | 0.831 | 0.968 | 0.982 | -0.984 | 0.966 | 0.901 | 0.893 | 0.975 | 0.929 | 0.888 | 0.948 | 0.824 | 0.869 | 0.916 | 0.960 | 0.926 | 0.891 | 0.962 | 0.875 | 0.921 | 0.952 | 0.884 | 0.919 | 0.647 | 0.699 | ##### | -0.811 | 0.959 | 0.955 | -0.931 | 0.659 -0.981 0.785 ##### 0.976 ##### -0.555 0.939 0.832 0.836 -0.765 0.870 0.785 0.994 0.978 0.992 0.554 0.776 ##### 0.945 0.971 0.963 0.346 0.817 -0.285 0.843 -0.828 0.917 ##### 0.948 0.885 0.925 -0.792 0.555 -0.911 -0.925 0.943 0.634 -0.653 0.961 |
| Root γ-glutamate-cysteine | 0.555 | 0.786 | ##### | 0.720 | -0.781 | 0.774 | 0.582 | 0.864 | 0.895 | 0.860 | 0.964 | 0.812 | 0.562 | 0.559 | 0.520 | 0.466 | 0.854 | 0.433 | 0.777 | 0.718 | 0.378 | 0.874 | ##### | 0.665 | 0.985 | 0.504 | 0.837 | 0.741 | #### | 0.889 | 0.766 | 0.944 | 0.726 | 0.775 | -0.708 | 0.741 | 0.870 | 0.964 | 0.609 | 0.542 | 0.961 | 0.901 | 0.894 | 0.919 | 0.515 | 0.802 | 0.855 | 0.872 | 0.891 | 0.926 | 0.936 | 0.901 | 0.932 | 0.835 | 0.908 | 0.940 | ##### | -0.898 | 0.904 | 0.913 | -0.930 | 0.921 -0.657 0.358 ##### 0.810 ##### -0.836 0.723 0.554 0.565 -0.493 0.409 0.498 0.779 0.626 0.712 0.315 0.893 ##### 0.768 0.812 0.892 -0.258 0.948 -0.414 0.363 -0.406 0.947 ##### 0.730 0.913 0.937 -0.973 0.243 -0.946 -0.733 0.926 0.913 -0.510 0.829 |
| Root Nα-acetyl-L-glutamine | 0.883 | 0.882 | ##### | 0.690 | -0.865 | 0.889 | 0.993 | 0.802 | 0.754 | 0.852 | 0.662 | 0.893 | 0.991 | 0.969 | 0.955 | 0.972 | 0.854 | 0.969 | 0.546 | 0.951 | 0.905 | 0.783 | ##### | 0.970 | 0.383 | 0.942 | 0.807 | 0.909 | #### | 0.757 | 0.877 | 0.511 | 0.941 | 0.923 | -0.946 | 0.936 | 0.812 | 0.660 | 0.951 | 0.978 | 0.625 | 0.788 | 0.727 | 0.737 | 0.970 | 0.913 | 0.795 | 0.805 | 0.821 | 0.737 | 0.731 | 0.749 | 0.742 | 0.853 | 0.252 | 0.328 | ##### | -0.628 | 0.758 | 0.761 | -0.689 | 0.266 -0.956 0.894 0.049 0.827 ##### -0.145 0.895 0.926 0.861 -0.637 0.972 0.924 0.898 0.953 0.923 0.284 0.401 ##### 0.930 0.891 0.817 0.346 0.482 -0.536 0.975 -0.982 0.678 0.172 0.914 0.579 0.686 -0.474 0.870 -0.662 -0.711 0.758 0.233 -0.511 0.860 |
| Root N-acetyl-L-tyrosine | 0.901 | 0.972 | ##### | 0.970 | -0.963 | 0.957 | 0.828 | 0.970 | 0.963 | 0.950 | 0.918 | 0.933 | 0.833 | 0.875 | 0.882 | 0.802 | 0.935 | 0.656 | 0.831 | 0.851 | 0.745 | 0.964 | ##### | 0.896 | 0.768 | 0.849 | 0.970 | 0.947 | #### | 0.966 | 0.937 | 0.894 | 0.930 | 0.918 | -0.921 | 0.930 | 0.931 | 0.879 | 0.903 | 0.785 | 0.903 | 0.959 | 0.889 | 0.919 | 0.755 | 0.884 | 0.975 | 0.926 | 0.925 | 0.919 | 0.944 | 0.967 | 0.916 | 0.937 | 0.674 | 0.722 | ##### | -0.928 | 0.928 | 0.934 | -0.923 | 0.706 -0.895 0.805 ##### 0.989 ##### -0.548 0.960 0.846 0.902 -0.905 0.702 0.789 0.923 0.885 0.943 0.719 0.844 ##### 0.906 0.951 0.935 0.366 0.858 -0.251 0.762 -0.758 0.916 ##### 0.950 0.875 0.934 -0.853 0.420 -0.924 -0.803 0.933 0.695 -0.857 0.967 |
| Root homo-Arg | 0.921 | 0.952 | ##### | 0.793 | -0.938 | 0.953 | 0.996 | 0.897 | 0.861 | 0.933 | 0.786 | 0.959 | 0.993 | 0.981 | 0.967 | 0.960 | 0.932 | 0.931 | 0.664 | 0.980 | 0.886 | 0.882 | ##### | 0.997 | 0.539 | 0.948 | 0.899 | 0.963 | #### | 0.863 | 0.938 | 0.659 | 0.987 | 0.976 | -0.988 | 0.984 | 0.896 | 0.779 | 0.980 | 0.976 | 0.755 | 0.887 | 0.821 | 0.838 | 0.962 | 0.966 | 0.888 | 0.889 | 0.908 | 0.840 | 0.842 | 0.858 | 0.844 | 0.927 | 0.414 | 0.486 | ##### | -0.749 | 0.860 | 0.864 | -0.807 | 0.431 -0.987 0.886 ##### 0.914 ##### -0.301 0.953 0.938 0.897 -0.711 0.944 0.921 0.959 0.981 0.974 0.381 0.558 ##### 0.977 0.958 0.906 0.315 0.631 -0.509 0.948 -0.956 0.798 0.039 0.967 0.710 0.806 -0.625 0.792 -0.786 -0.789 0.862 0.401 -0.601 0.938 |
| Root S-(5-adenosy)-L-homocysteine | 0.719 | 0.875 | ##### | 0.864 | -0.889 | 0.852 | 0.671 | 0.946 | 0.969 | 0.923 | 0.993 | 0.890 | 0.657 | 0.668 | 0.648 | 0.568 | 0.923 | 0.487 | 0.887 | 0.769 | 0.470 | 0.955 | ##### | 0.759 | 0.966 | 0.610 | 0.933 | 0.823 | #### | 0.966 | 0.829 | 0.989 | 0.816 | 0.855 | -0.810 | 0.826 | 0.905 | 0.990 | 0.741 | 0.653 | 0.998 | 0.957 | 0.897 | 0.937 | 0.627 | 0.858 | 0.919 | 0.902 | 0.944 | 0.944 | 0.978 | 0.970 | 0.950 | 0.883 | 0.910 | 0.942 | ##### | -0.925 | 0.967 | 0.971 | -0.987 | 0.925 -0.771 0.494 ##### 0.910 ##### -0.829 0.816 0.635 0.670 -0.650 0.531 0.569 0.872 0.750 0.826 0.501 0.952 ##### 0.830 0.886 0.948 -0.022 0.986 -0.291 0.498 -0.515 0.992 ##### 0.818 0.975 0.990 -0.990 0.258 -0.995 -0.839 0.974 0.915 -0.630 0.902 |
| Root γ-aminobutyric acid | 0.846 | 0.901 | ##### | 0.929 | -0.947 | 0.861 | 0.734 | 0.971 | 0.981 | 0.931 | 0.956 | 0.927 | 0.721 | 0.727 | 0.721 | 0.626 | 0.951 | 0.533 | 0.963 | 0.785 | 0.500 | 0.980 | ##### | 0.814 | 0.883 | 0.653 | 0.974 | 0.839 | #### | 0.981 | 0.818 | 0.958 | 0.858 | 0.900 | -0.875 | 0.862 | 0.868 | 0.970 | 0.838 | 0.764 | 0.977 | 0.950 | 0.821 | 0.879 | 0.748 | 0.887 | 0.902 | 0.860 | 0.955 | 0.889 | 0.955 | 0.975 | 0.900 | 0.863 | 0.869 | 0.899 | ##### | -0.851 | 0.986 | 0.980 | -0.992 | 0.877 -0.857 0.562 ##### 0.941 ##### -0.802 0.837 0.650 0.685 -0.689 0.667 0.582 0.931 0.850 0.895 0.574 0.937 ##### 0.847 0.906 0.957 0.173 0.961 -0.164 0.607 -0.593 0.984 ##### 0.842 0.991 0.985 -0.937 0.291 -0.983 -0.945 0.969 0.860 -0.613 0.911 |
| Root N'-formylkynurenine | 0.962 | 0.990 | ##### | 0.937 | -0.993 | 0.974 | 0.928 | 0.972 | 0.952 | 0.968 | 0.881 | 0.978 | 0.928 | 0.944 | 0.946 | 0.888 | 0.968 | 0.788 | 0.817 | 0.925 | 0.809 | 0.964 | ##### | 0.969 | 0.682 | 0.906 | 0.978 | 0.974 | #### | 0.955 | 0.948 | 0.818 | 0.983 | 0.977 | -0.986 | 0.981 | 0.926 | 0.864 | 0.979 | 0.913 | 0.867 | 0.952 | 0.857 | 0.890 | 0.893 | 0.949 | 0.954 | 0.918 | 0.948 | 0.893 | 0.921 | 0.949 | 0.895 | 0.945 | 0.591 | 0.648 | ##### | -0.852 | 0.935 | 0.937 | -0.907 | 0.613 -0.977 0.856 ##### 0.989 ##### -0.476 0.977 0.893 0.908 -0.841 0.854 0.849 0.979 0.972 0.996 0.606 0.753 ##### 0.958 0.980 0.952 0.391 0.790 -0.320 0.874 -0.866 0.895 ##### 0.979 0.845 0.909 -0.775 0.581 -0.894 -0.860 0.930 0.592 -0.748 0.978 |
| Root ethanolamine | 0.481 | 0.619 | ##### | 0.673 | -0.679 | 0.574 | 0.377 | 0.762 | 0.806 | 0.711 | 0.864 | 0.680 | 0.349 | 0.338 | 0.312 | 0.216 | 0.738 | 0.177 | 0.860 | 0.502 | 0.081 | 0.786 | ##### | 0.478 | 0.955 | 0.249 | 0.750 | 0.532 | #### | 0.801 | 0.535 | 0.912 | 0.547 | 0.632 | -0.561 | 0.560 | 0.667 | 0.903 | 0.478 | 0.420 | 0.906 | 0.766 | 0.672 | 0.732 | 0.407 | 0.652 | 0.677 | 0.664 | 0.777 | 0.746 | 0.814 | 0.802 | 0.762 | 0.628 | 0.991 | 0.992 | ##### | -0.715 | 0.835 | 0.829 | -0.876 | 0.984 -0.527 0.110 ##### 0.683 ##### -0.980 0.517 0.276 0.308 -0.339 0.292 0.202 0.673 0.510 0.581 0.324 0.923 ##### 0.572 0.648 0.775 -0.237 0.946 -0.100 0.165 -0.171 0.883 ##### 0.527 0.926 0.867 -0.937 -0.031 -0.882 -0.786 0.824 0.977 -0.324 0.661 |
| Root TFAADs | 0.959 | 0.978 | ##### | 0.925 | -0.998 | 0.955 | 0.919 | 0.979 | 0.961 | 0.972 | 0.894 | 0.989 | 0.914 | 0.916 | 0.913 | 0.854 | 0.983 | 0.775 | 0.858 | 0.922 | 0.751 | 0.975 | ##### | 0.961 | 0.710 | 0.862 | 0.986 | 0.952 | #### | 0.964 | 0.922 | 0.833 | 0.975 | 0.986 | -0.988 | 0.974 | 0.914 | 0.895 | 0.976 | 0.929 | 0.890 | 0.955 | 0.841 | 0.882 | 0.914 | 0.965 | 0.936 | 0.905 | 0.966 | 0.888 | 0.928 | 0.956 | 0.895 | 0.932 | 0.642 | 0.695 | ##### | -0.825 | 0.960 | 0.957 | -0.931 | 0.656 -0.983 0.797 ##### 0.981 ##### -0.545 0.949 0.848 0.851 -0.773 0.868 0.802 0.995 0.978 0.994 0.549 0.773 ##### 0.955 0.978 0.968 0.336 0.816 -0.310 0.848 -0.837 0.919 ##### 0.958 0.881 0.927 -0.795 0.570 -0.914 -0.914 0.947 0.631 -0.666 0.969 |
| Root TFAADs/N | 0.935 | 0.951 | ##### | 0.934 | -0.991 | 0.919 | 0.860 | 0.983 | 0.974 | 0.961 | 0.917 | 0.976 | 0.850 | 0.849 | 0.845 | 0.770 | 0.981 | 0.691 | 0.919 | 0.875 | 0.649 | 0.985 | ##### | 0.914 | 0.771 | 0.782 | 0.991 | 0.909 | #### | 0.976 | 0.877 | 0.879 | 0.938 | 0.965 | -0.957 | 0.937 | 0.891 | 0.930 | 0.939 | 0.890 | 0.927 | 0.955 | 0.820 | 0.873 | 0.877 | 0.947 | 0.918 | 0.881 | 0.970 | 0.881 | 0.938 | 0.967 | 0.891 | 0.902 | 0.734 | 0.778 | ##### | -0.819 | 0.980 | 0.974 | -0.962 | 0.742 -0.952 0.705 ##### 0.970 ##### -0.654 0.905 0.767 0.778 -0.730 0.819 0.712 0.986 0.948 0.969 0.553 0.838 ##### 0.918 0.956 0.971 0.288 0.874 -0.238 0.769 -0.753 0.949 ##### 0.915 0.934 0.954 -0.847 0.471 -0.944 -0.955 0.961 0.719 -0.623 0.948 |
| Root molar ratio of C/N in TFAADs | -0.943 | ##### | 0.916 | ##### | 0.958 | -0.958 | ##### | ##### | -0.885 | ##### | -0.807 | -0.970 | -0.984 | -0.976 | -0.967 | ##### | -0.946 | -0.904 | -0.712 | -0.970 | -0.866 | ##### | 0.954 | -0.997 | -0.568 | ##### | -0.923 | -0.966 | #### | ##### | -0.937 | ##### | -0.991 | -0.983 | 0.996 | -0.988 | -0.899 | -0.801 | ##### | -0.976 | -0.782 | -0.903 | ##### | -0.845 | -0.962 | -0.968 | -0.901 | -0.892 | -0.922 | -0.847 | ##### | -0.881 | -0.852 | -0.929 | -0.457 | ##### | 0.309 | 0.763 | -0.884 | -0.885 | 0.834 | -0.473 0.996 -0.880 0.106 ##### 0.789 0.347 -0.958 -0.925 ##### 0.737 -0.940 -0.902 -0.973 ##### -0.987 -0.432 ##### 0.926 -0.976 -0.967 -0.921 -0.344 ##### 0.455 -0.940 0.941 -0.823 ##### -0.971 -0.748 -0.833 0.658 -0.748 0.813 0.823 -0.881 -0.444 0.623 -0.948 |
| Root TFAADs/C | 0.943 | 0.972 | ##### | 0.937 | -0.998 | 0.946 | 0.888 | 0.989 | 0.978 | 0.975 | 0.920 | 0.986 | 0.881 | 0.884 | 0.880 | 0.812 | 0.988 | 0.730 | 0.891 | 0.903 | 0.704 | 0.988 | ##### | 0.939 | 0.759 | 0.825 | 0.995 | 0.938 | #### | 0.980 | 0.910 | 0.872 | 0.960 | 0.978 | -0.973 | 0.960 | 0.916 | 0.924 | 0.955 | 0.901 | 0.921 | 0.966 | 0.848 | 0.894 | 0.885 | 0.959 | 0.941 | 0.907 | 0.976 | 0.900 | 0.946 | 0.972 | 0.908 | 0.928 | 0.702 | 0.751 | ##### | -0.843 | 0.977 | 0.974 | -0.957 | 0.715 -0.965 0.753 ##### 0.984 ##### -0.610 0.935 0.814 0.824 -0.763 0.831 0.762 0.992 0.960 0.983 0.561 0.820 ##### 0.942 0.974 0.977 0.300 0.860 -0.284 0.802 -0.791 0.945 ##### 0.943 0.916 0.952 -0.838 0.513 -0.941 -0.930 0.964 0.692 -0.663 0.968 |
| Root NR | 0.815 | 0.967 | ##### | 0.793 | -0.905 | 0.987 | 0.933 | 0.905 | 0.884 | 0.950 | 0.860 | 0.934 | 0.936 | 0.951 | 0.930 | 0.926 | 0.918 | 0.866 | 0.602 | 0.967 | 0.901 | 0.888 | ##### | 0.955 | 0.661 | 0.947 | 0.889 | 0.989 | #### | 0.884 | 0.998 | 0.744 | 0.966 | 0.938 | -0.934 | 0.970 | 0.976 | 0.814 | 0.895 | 0.837 | 0.807 | 0.931 | 0.951 | 0.944 | 0.806 | 0.933 | 0.963 | 0.975 | 0.910 | 0.940 | 0.904 | 0.893 | 0.934 | 0.987 | 0.476 | 0.550 | ##### | -0.904 | 0.857 | 0.875 | -0.827 | 0.511 -0.903 0.876 ##### 0.929 ##### -0.328 0.983 0.969 0.956 -0.786 0.770 0.948 0.904 0.881 0.925 0.431 0.628 ##### 0.976 0.965 0.914 0.180 0.697 -0.612 0.847 -0.887 0.832 ##### 0.984 0.716 0.844 -0.725 0.717 -0.831 -0.660 0.891 0.494 -0.755 0.968 |
| Root GOGAT | 0.813 | 0.933 | ##### | 0.931 | -0.942 | 0.910 | 0.747 | 0.981 | 0.992 | 0.955 | 0.988 | 0.930 | 0.739 | 0.762 | 0.752 | 0.668 | 0.952 | 0.559 | 0.898 | 0.816 | 0.580 | 0.984 | ##### | 0.830 | 0.914 | 0.712 | 0.973 | 0.888 | #### | 0.992 | 0.886 | 0.979 | 0.879 | 0.902 | -0.874 | 0.885 | 0.933 | 0.973 | 0.824 | 0.723 | 0.988 | 0.981 | 0.909 | 0.948 | 0.696 | 0.889 | 0.960 | 0.928 | 0.961 | 0.953 | 0.988 | 0.994 | 0.957 | 0.921 | 0.848 | 0.885 | ##### | -0.943 | 0.979 | 0.983 | -0.990 | 0.868 -0.841 0.622 ##### 0.964 ##### -0.750 0.889 0.725 0.770 -0.763 0.614 0.660 0.916 0.824 0.893 0.602 0.938 ##### 0.877 0.932 0.967 0.136 0.964 -0.268 0.611 -0.619 0.989 ##### 0.886 0.967 0.995 -0.963 0.320 -0.994 -0.857 0.983 0.857 -0.728 0.948 |
| Root GOT | 0.980 | 0.975 | ##### | 0.927 | -0.983 | 0.959 | 0.937 | 0.948 | 0.921 | 0.944 | 0.834 | 0.963 | 0.940 | 0.955 | 0.962 | 0.906 | 0.946 | 0.804 | 0.796 | 0.916 | 0.827 | 0.938 | ##### | 0.971 | 0.614 | 0.919 | 0.958 | 0.962 | #### | 0.926 | 0.930 | 0.768 | 0.977 | 0.967 | -0.985 | 0.973 | 0.892 | 0.818 | 0.991 | 0.931 | 0.820 | 0.919 | 0.813 | 0.847 | 0.914 | 0.933 | 0.924 | 0.883 | 0.920 | 0.849 | 0.881 | 0.918 | 0.852 | 0.919 | 0.529 | 0.587 | ##### | -0.804 | 0.904 | 0.904 | -0.870 | 0.550 -0.983 0.882 ##### 0.971 ##### -0.416 0.967 0.896 0.909 -0.843 0.885 0.856 0.970 0.982 0.994 0.612 0.704 ##### 0.945 0.963 0.924 0.458 0.738 -0.292 0.907 -0.892 0.854 0.039 0.970 0.806 0.870 -0.717 0.604 -0.852 -0.852 0.895 0.527 -0.735 0.956 |
| Root GPT | 0.961 | 0.961 | ##### | 0.895 | -0.946 | 0.956 | 0.943 | 0.904 | 0.870 | 0.911 | 0.777 | 0.926 | 0.952 | 0.978 | 0.988 | 0.945 | 0.900 | 0.833 | 0.702 | 0.909 | 0.895 | 0.888 | ##### | 0.967 | 0.532 | 0.961 | 0.913 | 0.965 | #### | 0.875 | 0.938 | 0.699 | 0.967 | 0.936 | -0.965 | 0.961 | 0.878 | 0.743 | 0.978 | 0.909 | 0.749 | 0.881 | 0.802 | 0.823 | 0.888 | 0.897 | 0.910 | 0.870 | 0.868 | 0.821 | 0.835 | 0.870 | 0.819 | 0.909 | 0.417 | 0.481 | ##### | -0.792 | 0.840 | 0.844 | -0.801 | 0.446 -0.961 0.944 0.031 0.945 ##### -0.286 0.974 0.940 0.956 -0.883 0.873 0.908 0.928 0.959 0.973 0.626 0.624 ##### 0.932 0.940 0.875 0.512 0.655 -0.327 0.939 -0.928 0.786 0.143 0.972 0.718 0.807 -0.642 0.647 -0.786 -0.760 0.839 0.425 -0.787 0.937 |
| Root glucose | 0.787 | 0.938 | ##### | 0.880 | -0.938 | 0.922 | 0.780 | 0.979 | 0.989 | 0.972 | 0.998 | 0.948 | 0.768 | 0.775 | 0.753 | 0.691 | 0.969 | 0.621 | 0.860 | 0.861 | 0.600 | 0.982 | ##### | 0.852 | 0.920 | 0.724 | 0.966 | 0.901 | #### | 0.987 | 0.904 | 0.960 | 0.896 | 0.924 | -0.887 | 0.904 | 0.957 | 0.990 | 0.825 | 0.751 | 0.990 | 0.991 | 0.940 | 0.972 | 0.725 | 0.926 | 0.964 | 0.954 | 0.982 | 0.977 | 0.998 | 0.991 | 0.982 | 0.945 | 0.834 | 0.879 | ##### | -0.943 | 0.986 | 0.992 | -0.990 | 0.852 -0.852 0.613 ##### 0.954 ##### -0.738 0.892 0.747 0.764 -0.696 0.642 0.691 0.929 0.831 0.896 0.485 0.901 ##### 0.909 0.948 0.985 0.032 0.949 -0.387 0.623 -0.644 0.994 ##### 0.897 0.954 0.995 -0.956 0.406 -0.994 -0.849 0.997 0.838 -0.663 0.958 |
| Root fructose | 0.818 | 0.944 | ##### | 0.895 | -0.954 | 0.924 | 0.796 | 0.987 | 0.995 | 0.976 | 0.992 | 0.959 | 0.783 | 0.788 | 0.769 | 0.703 | 0.978 | 0.631 | 0.883 | 0.867 | 0.603 | 0.990 | ##### | 0.865 | 0.905 | 0.731 | 0.978 | 0.904 | #### | 0.993 | 0.901 | 0.956 | 0.907 | 0.937 | -0.904 | 0.914 | 0.950 | 0.990 | 0.849 | 0.779 | 0.990 | 0.991 | 0.922 | 0.959 | 0.756 | 0.936 | 0.960 | 0.945 | 0.988 | 0.965 | 0.995 | 0.995 | 0.972 | 0.941 | 0.831 | 0.875 | ##### | -0.925 | 0.995 | 0.998 | -0.995 | 0.846 -0.874 0.624 ##### 0.962 ##### -0.739 0.896 0.748 0.764 -0.700 0.676 0.691 0.946 0.857 0.913 0.497 0.901 ##### 0.914 0.954 0.990 0.072 0.947 -0.357 0.647 -0.661 0.996 ##### 0.902 0.962 0.997 -0.947 0.413 -0.995 -0.880 0.999 0.830 -0.652 0.961 |
| Root sucrose | 0.808 | 0.923 | ##### | 0.889 | -0.945 | 0.897 | 0.769 | 0.980 | 0.990 | 0.962 | 0.988 | 0.948 | 0.755 | 0.754 | 0.735 | 0.663 | 0.971 | 0.597 | 0.907 | 0.842 | 0.552 | 0.986 | ##### | 0.842 | 0.914 | 0.690 | 0.972 | 0.876 | #### | 0.988 | 0.868 | 0.960 | 0.885 | 0.924 | -0.888 | 0.892 | 0.925 | 0.995 | 0.833 | 0.769 | 0.993 | 0.979 | 0.895 | 0.938 | 0.748 | 0.924 | 0.937 | 0.920 | 0.982 | 0.946 | 0.986 | 0.988 | 0.955 | 0.915 | 0.859 | 0.898 | ##### | -0.900 | 0.996 | 0.996 | -0.999 | 0.870 -0.861 0.578 ##### 0.947 ##### -0.779 0.866 0.703 0.719 -0.665 0.665 0.644 0.939 0.845 0.899 0.485 0.914 ##### 0.892 0.937 0.983 0.057 0.959 -0.319 0.615 -0.624 0.999 ##### 0.874 0.977 0.997 -0.953 0.376 -0.996 -0.903 0.994 0.853 -0.610 0.941 |
| Root total soluble sugars | 0.810 | 0.939 | ##### | 0.892 | -0.949 | 0.918 | 0.787 | 0.985 | 0.993 | 0.973 | 0.993 | 0.955 | 0.774 | 0.778 | 0.759 | 0.692 | 0.976 | 0.622 | 0.884 | 0.860 | 0.591 | 0.988 | ##### | 0.858 | 0.911 | 0.721 | 0.975 | 0.898 | #### | 0.992 | 0.895 | 0.959 | 0.901 | 0.932 | -0.898 | 0.908 | 0.947 | 0.992 | 0.841 | 0.772 | 0.991 | 0.989 | 0.920 | 0.958 | 0.748 | 0.932 | 0.956 | 0.942 | 0.986 | 0.964 | 0.995 | 0.993 | 0.971 | 0.937 | 0.839 | 0.882 | ##### | -0.924 | 0.994 | 0.997 | -0.996 | 0.854 -0.867 0.612 ##### 0.958 ##### -0.748 0.889 0.738 0.754 -0.691 0.667 0.681 0.942 0.849 0.907 0.492 0.905 ##### 0.909 0.950 0.988 0.060 0.951 -0.355 0.635 -0.649 0.998 ##### 0.895 0.965 0.998 -0.951 0.403 -0.996 -0.880 0.998 0.838 -0.645 0.957 |
| Root isocitrate | 0.899 | 0.909 | ##### | 0.754 | -0.926 | 0.898 | 0.963 | 0.887 | 0.855 | 0.916 | 0.784 | 0.955 | 0.951 | 0.914 | 0.894 | 0.885 | 0.936 | 0.894 | 0.716 | 0.954 | 0.773 | 0.878 | ##### | 0.964 | 0.563 | 0.856 | 0.891 | 0.905 | #### | 0.856 | 0.870 | 0.661 | 0.953 | 0.970 | -0.970 | 0.951 | 0.851 | 0.808 | 0.956 | 0.987 | 0.770 | 0.868 | 0.768 | 0.800 | 0.983 | 0.971 | 0.835 | 0.843 | 0.916 | 0.807 | 0.830 | 0.846 | 0.819 | 0.880 | 0.480 | 0.545 | ##### | -0.681 | 0.879 | 0.876 | -0.825 | 0.483 -0.978 0.771 ##### 0.879 ##### -0.400 0.885 0.844 0.781 -0.581 0.955 0.824 0.965 0.974 0.952 0.276 0.572 ##### 0.949 0.932 0.909 0.225 0.654 -0.478 0.890 -0.892 0.814 ##### 0.910 0.748 0.813 -0.637 0.762 -0.796 -0.862 0.867 0.447 -0.446 0.900 |
| Root NADP-ME | 0.814 | 0.976 | ##### | 0.879 | -0.933 | 0.981 | 0.854 | 0.958 | 0.954 | 0.972 | 0.946 | 0.942 | 0.855 | 0.883 | 0.868 | 0.825 | 0.944 | 0.729 | 0.733 | 0.912 | 0.785 | 0.949 | ##### | 0.909 | 0.806 | 0.867 | 0.943 | 0.970 | #### | 0.953 | 0.981 | 0.880 | 0.942 | 0.929 | -0.914 | 0.948 | 0.991 | 0.901 | 0.866 | 0.770 | 0.910 | 0.979 | 0.976 | 0.984 | 0.736 | 0.919 | 0.998 | 0.990 | 0.946 | 0.982 | 0.969 | 0.962 | 0.978 | 0.988 | 0.657 | 0.716 | ##### | -0.974 | 0.924 | 0.938 | -0.916 | 0.692 -0.875 0.789 ##### 0.969 ##### -0.519 0.971 0.890 0.914 -0.825 0.677 0.848 0.913 0.852 0.920 0.549 0.794 ##### 0.948 0.969 0.953 0.160 0.841 -0.478 0.742 -0.773 0.921 ##### 0.966 0.840 0.933 -0.863 0.531 -0.926 -0.729 0.952 0.680 -0.809 0.985 |
| Root NADP-MDH | 0.898 | 0.988 | ##### | 0.902 | -0.988 | 0.975 | 0.905 | 0.993 | 0.983 | 0.998 | 0.950 | 0.996 | 0.897 | 0.899 | 0.883 | 0.835 | 0.998 | 0.770 | 0.835 | 0.942 | 0.744 | 0.989 | ##### | 0.952 | 0.794 | 0.851 | 0.989 | 0.967 | #### | 0.984 | 0.953 | 0.880 | 0.976 | 0.988 | -0.974 | 0.979 | 0.966 | 0.943 | 0.938 | 0.885 | 0.936 | 0.990 | 0.918 | 0.949 | 0.864 | 0.981 | 0.973 | 0.961 | 0.994 | 0.953 | 0.975 | 0.983 | 0.959 | 0.973 | 0.697 | 0.754 | ##### | -0.897 | 0.980 | 0.984 | -0.960 | 0.715 -0.954 0.762 ##### 0.986 ##### -0.591 0.959 0.856 0.855 -0.750 0.806 0.812 0.988 0.941 0.977 0.494 0.807 ##### 0.974 0.995 0.995 0.192 0.862 -0.416 0.795 -0.805 0.957 ##### 0.967 0.902 0.962 -0.859 0.570 -0.953 -0.878 0.983 0.694 -0.676 0.992 |
| Root NAD-MDH | 0.913 | 0.980 | ##### | 0.960 | -0.990 | 0.959 | 0.852 | 0.997 | 0.992 | 0.979 | 0.950 | 0.973 | 0.849 | 0.869 | 0.867 | 0.792 | 0.979 | 0.682 | 0.883 | 0.883 | 0.704 | 0.995 | ##### | 0.917 | 0.810 | 0.823 | 0.997 | 0.947 | #### | 0.994 | 0.931 | 0.917 | 0.949 | 0.957 | -0.950 | 0.950 | 0.942 | 0.935 | 0.923 | 0.837 | 0.945 | 0.984 | 0.893 | 0.931 | 0.813 | 0.935 | 0.973 | 0.935 | 0.972 | 0.935 | 0.971 | 0.991 | 0.939 | 0.947 | 0.735 | 0.782 | ##### | -0.910 | 0.977 | 0.978 | -0.968 | 0.757 -0.929 0.755 ##### 0.997 ##### -0.628 0.950 0.821 0.854 -0.823 0.752 0.764 0.968 0.919 0.966 0.629 0.866 ##### 0.933 0.974 0.977 0.286 0.896 -0.281 0.760 -0.756 0.961 ##### 0.950 0.927 0.971 -0.885 0.451 -0.963 -0.883 0.974 0.740 -0.754 0.980 |
| Root PEPC | 0.969 | 0.930 | ##### | 0.835 | -0.950 | 0.914 | 0.967 | 0.896 | 0.859 | 0.907 | 0.759 | 0.948 | 0.964 | 0.951 | 0.950 | 0.919 | 0.923 | 0.872 | 0.741 | 0.928 | 0.821 | 0.885 | ##### | 0.975 | 0.513 | 0.903 | 0.909 | 0.924 | #### | 0.863 | 0.880 | 0.660 | 0.964 | 0.963 | -0.983 | 0.958 | 0.836 | 0.767 | 0.995 | 0.989 | 0.747 | 0.861 | 0.739 | 0.775 | 0.982 | 0.941 | 0.848 | 0.825 | 0.891 | 0.780 | 0.814 | 0.850 | 0.788 | 0.872 | 0.439 | 0.501 | ##### | -0.687 | 0.863 | 0.859 | -0.811 | 0.449 -0.996 0.857 ##### 0.910 ##### -0.346 0.918 0.875 0.846 -0.715 0.966 0.848 0.964 0.998 0.979 0.453 0.587 ##### 0.936 0.932 0.889 0.424 0.642 -0.347 0.942 -0.925 0.792 0.093 0.933 0.739 0.802 -0.614 0.711 -0.781 -0.859 0.845 0.417 -0.574 0.908 |
| Root PEPP | 0.816 | 0.931 | ##### | 0.944 | -0.938 | 0.907 | 0.734 | 0.976 | 0.988 | 0.946 | 0.980 | 0.918 | 0.730 | 0.759 | 0.754 | 0.664 | 0.940 | 0.541 | 0.896 | 0.800 | 0.582 | 0.979 | ##### | 0.821 | 0.905 | 0.713 | 0.970 | 0.885 | #### | 0.987 | 0.883 | 0.979 | 0.872 | 0.889 | -0.865 | 0.877 | 0.925 | 0.960 | 0.820 | 0.707 | 0.980 | 0.974 | 0.902 | 0.941 | 0.679 | 0.872 | 0.960 | 0.921 | 0.948 | 0.945 | 0.981 | 0.990 | 0.947 | 0.914 | 0.840 | 0.876 | ##### | -0.947 | 0.968 | 0.972 | -0.981 | 0.863 -0.831 0.632 ##### 0.964 ##### -0.738 0.889 0.724 0.779 -0.791 0.599 0.658 0.905 0.816 0.888 0.643 0.942 ##### 0.865 0.924 0.955 0.172 0.961 -0.236 0.609 -0.613 0.980 ##### 0.884 0.960 0.988 -0.958 0.295 -0.987 -0.844 0.972 0.853 -0.760 0.943 |
| Root PK | 0.936 | 0.990 | ##### | 0.876 | -0.973 | 0.988 | 0.969 | 0.949 | 0.922 | 0.969 | 0.857 | 0.979 | 0.970 | 0.977 | 0.969 | 0.939 | 0.961 | 0.868 | 0.732 | 0.968 | 0.873 | 0.936 | ##### | 0.994 | 0.637 | 0.948 | 0.949 | 0.992 | #### | 0.924 | 0.973 | 0.761 | 0.999 | 0.985 | -0.994 | 0.997 | 0.942 | 0.836 | 0.980 | 0.934 | 0.828 | 0.941 | 0.879 | 0.899 | 0.914 | 0.966 | 0.950 | 0.936 | 0.941 | 0.899 | 0.905 | 0.922 | 0.900 | 0.964 | 0.512 | 0.579 | ##### | -0.843 | 0.908 | 0.913 | -0.870 | 0.536 -0.983 0.891 ##### 0.969 ##### -0.388 0.989 0.943 0.934 -0.805 0.882 0.913 0.973 0.973 0.992 0.508 0.671 ##### 0.985 0.987 0.944 0.335 0.727 -0.450 0.912 -0.919 0.862 ##### 0.995 0.784 0.874 -0.724 0.692 -0.858 -0.806 0.913 0.512 -0.718 0.979 |
| Root CS | 0.933 | 0.976 | ##### | 0.842 | -0.964 | 0.975 | 0.984 | 0.933 | 0.902 | 0.959 | 0.833 | 0.977 | 0.983 | 0.979 | 0.968 | 0.947 | 0.956 | 0.898 | 0.713 | 0.978 | 0.874 | 0.919 | ##### | 0.999 | 0.602 | 0.946 | 0.933 | 0.981 | #### | 0.904 | 0.958 | 0.723 | 0.997 | 0.988 | -0.996 | 0.996 | 0.925 | 0.821 | 0.984 | 0.960 | 0.805 | 0.923 | 0.856 | 0.876 | 0.943 | 0.974 | 0.926 | 0.918 | 0.935 | 0.877 | 0.884 | 0.900 | 0.881 | 0.951 | 0.480 | 0.549 | ##### | -0.802 | 0.896 | 0.900 | -0.851 | 0.500 -0.990 0.883 ##### 0.948 ##### -0.364 0.973 0.938 0.913 -0.757 0.916 0.913 0.975 0.982 0.989 0.445 0.629 ##### 0.986 0.979 0.935 0.317 0.694 -0.477 0.928 -0.935 0.843 ##### 0.984 0.762 0.852 -0.688 0.738 -0.834 -0.811 0.899 0.473 -0.657 0.965 |
| Root ACO | 0.802 | 0.963 | ##### | 0.897 | -0.939 | 0.958 | 0.808 | 0.975 | 0.979 | 0.975 | 0.981 | 0.943 | 0.804 | 0.829 | 0.813 | 0.755 | 0.956 | 0.658 | 0.803 | 0.879 | 0.695 | 0.971 | ##### | 0.876 | 0.876 | 0.798 | 0.960 | 0.941 | #### | 0.978 | 0.950 | 0.937 | 0.919 | 0.922 | -0.898 | 0.926 | 0.981 | 0.949 | 0.842 | 0.745 | 0.959 | 0.992 | 0.968 | 0.987 | 0.713 | 0.915 | 0.993 | 0.979 | 0.964 | 0.988 | 0.992 | 0.986 | 0.987 | 0.972 | 0.757 | 0.808 | ##### | -0.979 | 0.957 | 0.968 | -0.959 | 0.786 -0.858 0.708 ##### 0.971 ##### -0.635 0.940 0.822 0.852 -0.790 0.640 0.770 0.917 0.835 0.908 0.555 0.867 ##### 0.926 0.961 0.970 0.112 0.908 -0.415 0.676 -0.701 0.964 ##### 0.936 0.904 0.972 -0.924 0.444 -0.969 -0.782 0.978 0.775 -0.773 0.978 |
| Root NADP-IDH | 0.869 | 0.969 | ##### | 0.943 | -0.974 | 0.949 | 0.820 | 0.996 | 0.999 | 0.980 | 0.976 | 0.964 | 0.815 | 0.833 | 0.825 | 0.751 | 0.977 | 0.648 | 0.884 | 0.871 | 0.664 | 0.996 | ##### | 0.891 | 0.863 | 0.786 | 0.992 | 0.933 | #### | 0.999 | 0.925 | 0.947 | 0.930 | 0.944 | -0.926 | 0.934 | 0.953 | 0.961 | 0.886 | 0.797 | 0.971 | 0.993 | 0.917 | 0.953 | 0.772 | 0.929 | 0.978 | 0.948 | 0.979 | 0.957 | 0.989 | 0.999 | 0.961 | 0.950 | 0.784 | 0.830 | ##### | -0.935 | 0.985 | 0.989 | -0.984 | 0.806 -0.899 0.704 ##### 0.988 ##### -0.679 0.933 0.793 0.826 -0.791 0.701 0.735 0.954 0.885 0.941 0.597 0.895 ##### 0.923 0.967 0.984 0.198 0.928 -0.308 0.703 -0.709 0.981 ##### 0.933 0.946 0.988 -0.924 0.418 -0.983 -0.872 0.988 0.791 -0.739 0.977 |
| C distribution in roots | -0.626 | ##### | 0.885 | ##### | 0.817 | -0.754 | ##### | ##### | -0.918 | ##### | -0.961 | -0.823 | -0.546 | -0.544 | -0.518 | ##### | -0.867 | -0.380 | -0.884 | -0.682 | -0.318 | ##### | 0.846 | -0.660 | -0.983 | ##### | -0.872 | -0.719 | #### | ##### | -0.725 | ##### | -0.722 | -0.783 | 0.724 | -0.734 | -0.828 | -0.978 | ##### | -0.574 | -0.980 | -0.898 | ##### | -0.875 | -0.553 | -0.797 | -0.836 | -0.825 | -0.897 | -0.885 | ##### | -0.918 | -0.896 | -0.798 | -0.961 | ##### | 0.905 | 0.854 | -0.933 | -0.933 | 0.960 | -0.966 0.685 -0.338 0.736 ##### 0.978 0.908 -0.706 -0.500 ##### 0.514 -0.447 -0.432 -0.808 ##### -0.739 -0.407 ##### 0.556 -0.745 -0.807 -0.898 0.140 ##### 0.245 -0.369 0.385 -0.968 0.673 -0.714 -0.971 -0.959 0.986 -0.161 0.967 0.832 -0.933 -0.956 0.497 -0.820 |
| N distribution in roots | -0.666 | ##### | 0.926 | ##### | 0.855 | -0.819 | ##### | ##### | -0.947 | ##### | -0.987 | -0.865 | -0.618 | -0.622 | -0.595 | ##### | -0.902 | -0.458 | -0.865 | -0.746 | -0.421 | ##### | 0.886 | -0.723 | -0.980 | ##### | -0.904 | -0.788 | #### | ##### | -0.798 | ##### | -0.782 | -0.829 | 0.775 | -0.793 | -0.888 | -0.989 | ##### | -0.617 | -0.993 | -0.938 | ##### | -0.927 | -0.592 | -0.840 | -0.893 | -0.886 | -0.929 | -0.934 | ##### | -0.950 | -0.942 | -0.861 | -0.926 | ##### | 0.852 | 0.909 | -0.952 | -0.956 | 0.974 | -0.938 0.733 -0.433 0.653 ##### 0.989 0.853 -0.777 -0.593 ##### 0.586 -0.491 -0.529 -0.844 ##### -0.787 -0.436 ##### 0.646 -0.805 -0.859 -0.931 0.106 ##### 0.317 -0.444 0.467 -0.983 0.624 -0.782 -0.966 -0.978 0.991 -0.240 0.984 0.819 -0.961 -0.928 0.575 -0.875 |
| Root lle | 0.587 | 0.704 | ##### | 0.746 | -0.764 | 0.659 | 0.484 | 0.832 | 0.868 | 0.785 | 0.906 | 0.763 | 0.458 | 0.446 | 0.423 | 0.328 | 0.813 | 0.283 | 0.906 | 0.592 | 0.188 | 0.853 | ##### | 0.579 | 0.954 | 0.356 | 0.824 | 0.622 | #### | 0.864 | 0.617 | 0.938 | 0.642 | 0.720 | -0.658 | 0.652 | 0.732 | 0.943 | 0.585 | 0.529 | 0.943 | 0.829 | 0.722 | 0.783 | 0.516 | 0.734 | 0.745 | 0.727 | 0.843 | 0.797 | 0.867 | 0.862 | 0.813 | 0.701 | 0.981 | 0.990 | ##### | -0.756 | 0.895 | 0.888 | -0.926 | 0.975 -0.630 0.224 ##### 0.762 ##### -0.958 0.608 0.377 0.403 -0.418 0.408 0.303 0.760 0.614 0.677 0.373 0.940 ##### 0.659 0.730 0.842 -0.149 0.967 -0.129 0.287 -0.288 0.929 ##### 0.619 0.964 0.916 -0.953 0.065 -0.927 -0.853 0.881 0.964 -0.383 0.739 |
| Root creatine-phosphate | 0.840 | 0.916 | ##### | 0.972 | -0.909 | 0.896 | 0.717 | 0.930 | 0.934 | 0.891 | 0.898 | 0.862 | 0.726 | 0.785 | 0.800 | 0.699 | 0.872 | 0.518 | 0.832 | 0.749 | 0.654 | 0.927 | ##### | 0.803 | 0.789 | 0.764 | 0.930 | 0.881 | #### | 0.936 | 0.876 | 0.913 | 0.850 | 0.836 | -0.838 | 0.851 | 0.880 | 0.849 | 0.820 | 0.668 | 0.890 | 0.918 | 0.851 | 0.883 | 0.634 | 0.794 | 0.941 | 0.875 | 0.867 | 0.882 | 0.913 | 0.939 | 0.877 | 0.878 | 0.711 | 0.748 | ##### | -0.928 | 0.886 | 0.893 | -0.899 | 0.747 -0.806 0.733 ##### 0.951 ##### -0.587 0.902 0.762 0.852 -0.924 0.574 0.696 0.849 0.795 0.872 0.801 0.884 ##### 0.821 0.885 0.880 0.378 0.874 -0.137 0.654 -0.645 0.892 ##### 0.883 0.870 0.913 -0.869 0.263 -0.907 -0.753 0.892 0.743 -0.899 0.914 |
| Root starch | -0.802 | ##### | 0.978 | ##### | 0.940 | -0.900 | ##### | ##### | -0.992 | ##### | -0.992 | -0.934 | -0.735 | -0.748 | -0.734 | ##### | -0.958 | -0.562 | -0.905 | -0.820 | -0.555 | ##### | 0.956 | -0.826 | -0.925 | ##### | -0.972 | -0.877 | #### | ##### | -0.874 | ##### | -0.875 | -0.906 | 0.873 | -0.882 | -0.929 | -0.987 | ##### | -0.731 | -0.995 | -0.980 | ##### | -0.946 | -0.707 | -0.899 | -0.950 | -0.924 | -0.969 | -0.952 | ##### | -0.993 | -0.958 | -0.916 | -0.865 | ##### | 0.747 | 0.929 | -0.987 | -0.990 | 0.997 | -0.882 0.841 -0.591 0.523 ##### 0.997 0.776 -0.874 -0.706 ##### 0.717 -0.622 -0.642 -0.921 ##### -0.889 -0.553 ##### 0.753 -0.878 -0.930 -0.973 -0.090 ##### 0.285 -0.598 0.607 -0.997 0.447 -0.875 -0.976 -0.999 0.968 -0.329 0.999 0.875 -0.989 -0.869 0.677 -0.942 |
| Root TNC | -0.745 | ##### | 0.913 | ##### | 0.870 | -0.808 | ##### | ##### | -0.945 | ##### | -0.945 | -0.831 | -0.589 | -0.628 | -0.632 | ##### | -0.865 | -0.366 | -0.922 | -0.666 | -0.428 | ##### | 0.876 | -0.699 | -0.923 | ##### | -0.918 | -0.779 | #### | ##### | -0.776 | ##### | -0.763 | -0.790 | 0.762 | -0.768 | -0.836 | -0.928 | ##### | -0.584 | -0.962 | -0.911 | ##### | -0.869 | -0.556 | -0.767 | -0.889 | -0.831 | -0.877 | -0.874 | ##### | -0.946 | -0.877 | -0.816 | -0.900 | ##### | 0.761 | 0.902 | -0.923 | -0.923 | 0.954 | -0.921 0.727 -0.504 0.528 ##### 0.965 0.818 -0.789 -0.584 ##### 0.758 -0.466 -0.505 -0.821 ##### -0.798 -0.701 ##### 0.645 -0.750 -0.831 -0.887 -0.170 ##### 0.073 -0.467 0.459 -0.951 0.445 -0.778 -0.966 -0.959 0.970 -0.101 0.962 0.822 -0.919 -0.917 0.737 -0.859 |
| Root sucrose/starch | 0.837 | 0.952 | ##### | 0.933 | -0.958 | 0.931 | 0.784 | 0.990 | 0.997 | 0.970 | 0.987 | 0.949 | 0.777 | 0.796 | 0.786 | 0.708 | 0.968 | 0.606 | 0.890 | 0.847 | 0.619 | 0.992 | ##### | 0.861 | 0.895 | 0.747 | 0.983 | 0.911 | #### | 0.997 | 0.907 | 0.965 | 0.905 | 0.925 | -0.900 | 0.911 | 0.946 | 0.973 | 0.853 | 0.761 | 0.984 | 0.990 | 0.918 | 0.955 | 0.734 | 0.913 | 0.971 | 0.942 | 0.974 | 0.960 | 0.992 | 0.999 | 0.964 | 0.939 | 0.822 | 0.863 | ##### | -0.942 | 0.985 | 0.989 | -0.990 | 0.842 -0.869 0.657 ##### 0.976 ##### -0.721 0.910 0.758 0.795 -0.769 0.657 0.697 0.937 0.853 0.917 0.588 0.918 ##### 0.903 0.951 0.979 0.151 0.950 -0.299 0.654 -0.662 0.989 ##### 0.910 0.959 0.995 -0.948 0.373 -0.992 -0.866 0.989 0.829 -0.726 0.964 |
| Root malate + citrate + isocitrate | -0.740 | ##### | 0.942 | ##### | 0.891 | -0.838 | ##### | ##### | -0.968 | ##### | -0.981 | -0.875 | -0.635 | -0.656 | -0.645 | ##### | -0.909 | -0.441 | -0.915 | -0.733 | -0.451 | ##### | 0.907 | -0.742 | -0.956 | ##### | -0.937 | -0.809 | #### | ##### | -0.810 | ##### | -0.801 | -0.838 | 0.799 | -0.809 | -0.881 | -0.975 | ##### | -0.636 | -0.993 | -0.946 | ##### | -0.913 | -0.610 | -0.830 | -0.911 | -0.877 | -0.927 | -0.920 | ##### | -0.969 | -0.926 | -0.860 | -0.917 | ##### | 0.805 | 0.917 | -0.960 | -0.962 | 0.984 | -0.933 0.763 -0.498 0.587 ##### 0.993 0.838 -0.809 -0.614 ##### 0.692 -0.516 -0.542 -0.862 ##### -0.823 -0.582 ##### 0.670 -0.804 -0.871 -0.932 -0.058 ##### 0.198 -0.490 0.496 -0.985 0.517 -0.807 -0.984 -0.987 0.990 -0.196 0.991 0.849 -0.961 -0.925 0.669 -0.891 |
| Root NH_4_^+^-N | 0.748 | 0.880 | ##### | 0.620 | -0.804 | 0.915 | 0.957 | 0.778 | 0.739 | 0.857 | 0.703 | 0.860 | 0.959 | 0.951 | 0.921 | 0.964 | 0.825 | 0.967 | 0.412 | 0.966 | 0.951 | 0.753 | ##### | 0.938 | 0.454 | 0.956 | 0.761 | 0.930 | #### | 0.739 | 0.935 | 0.531 | 0.921 | 0.884 | -0.890 | 0.923 | 0.886 | 0.664 | 0.859 | 0.860 | 0.634 | 0.808 | 0.851 | 0.829 | 0.837 | 0.890 | 0.844 | 0.886 | 0.809 | 0.823 | 0.764 | 0.746 | 0.818 | 0.910 | 0.244 | 0.330 | ##### | -0.746 | 0.725 | 0.743 | -0.668 | 0.275 -0.871 0.888 0.099 0.808 ##### -0.103 0.915 0.976 0.913 -0.646 0.829 0.983 0.832 0.850 0.860 0.220 0.387 ##### 0.939 0.889 0.808 0.152 0.484 -0.742 0.897 -0.946 0.674 0.037 0.926 0.528 0.683 -0.516 0.890 -0.664 -0.546 0.762 0.250 -0.596 0.875 |
| Root Leu | 0.870 | 0.845 | ##### | 0.862 | -0.924 | 0.796 | 0.755 | 0.918 | 0.917 | 0.880 | 0.862 | 0.909 | 0.735 | 0.713 | 0.707 | 0.623 | 0.924 | 0.575 | 0.953 | 0.772 | 0.463 | 0.929 | ##### | 0.812 | 0.760 | 0.619 | 0.932 | 0.781 | #### | 0.918 | 0.737 | 0.846 | 0.836 | 0.894 | -0.876 | 0.836 | 0.776 | 0.910 | 0.858 | 0.840 | 0.897 | 0.876 | 0.697 | 0.768 | 0.838 | 0.884 | 0.802 | 0.764 | 0.918 | 0.782 | 0.869 | 0.903 | 0.801 | 0.784 | 0.786 | 0.815 | ##### | -0.693 | 0.948 | 0.932 | -0.933 | 0.778 -0.881 0.528 ##### 0.879 ##### -0.751 0.770 0.597 0.596 -0.564 0.772 0.537 0.933 0.884 0.888 0.455 0.831 ##### 0.817 0.863 0.914 0.211 0.866 -0.126 0.649 -0.618 0.917 ##### 0.789 0.939 0.913 -0.821 0.361 -0.907 -0.997 0.909 0.751 -0.434 0.847 |
| Root Val | 0.317 | 0.512 | ##### | 0.586 | -0.549 | 0.476 | 0.218 | 0.659 | 0.716 | 0.606 | 0.809 | 0.548 | 0.194 | 0.201 | 0.175 | 0.078 | 0.616 | 0.020 | 0.752 | 0.370 | -0.020 | 0.684 | ##### | 0.332 | 0.956 | 0.133 | 0.638 | 0.428 | #### | 0.709 | 0.453 | 0.882 | 0.415 | 0.490 | -0.411 | 0.431 | 0.599 | 0.826 | 0.314 | 0.223 | 0.843 | 0.682 | 0.640 | 0.686 | 0.201 | 0.512 | 0.611 | 0.600 | 0.665 | 0.696 | 0.746 | 0.718 | 0.706 | 0.546 | 0.978 | 0.970 | ##### | -0.710 | 0.729 | 0.729 | -0.790 | 0.979 -0.361 0.000 ##### 0.583 ##### -0.961 0.419 0.177 0.234 -0.305 0.079 0.102 0.526 0.336 0.436 0.324 0.900 ##### 0.449 0.534 0.668 -0.332 0.908 -0.076 -0.005 -0.015 0.806 ##### 0.419 0.846 0.791 -0.917 -0.181 -0.812 -0.622 0.734 0.982 -0.347 0.563 |
| Root Met | 0.554 | 0.738 | ##### | 0.702 | -0.767 | 0.711 | 0.541 | 0.843 | 0.877 | 0.825 | 0.937 | 0.793 | 0.514 | 0.496 | 0.458 | 0.394 | 0.839 | 0.377 | 0.831 | 0.669 | 0.273 | 0.859 | ##### | 0.624 | 0.974 | 0.419 | 0.824 | 0.675 | #### | 0.871 | 0.687 | 0.931 | 0.684 | 0.754 | -0.684 | 0.698 | 0.803 | 0.963 | 0.591 | 0.548 | 0.954 | 0.864 | 0.811 | 0.853 | 0.529 | 0.783 | 0.791 | 0.802 | 0.875 | 0.864 | 0.902 | 0.876 | 0.877 | 0.769 | 0.947 | 0.969 | ##### | -0.814 | 0.904 | 0.904 | -0.928 | 0.948 -0.644 0.267 ##### 0.774 ##### -0.906 0.654 0.458 0.461 -0.404 0.421 0.397 0.774 0.620 0.690 0.274 0.900 ##### 0.721 0.772 0.873 -0.264 0.952 -0.319 0.319 -0.346 0.941 ##### 0.668 0.936 0.925 -0.960 0.186 -0.936 -0.799 0.908 0.936 -0.392 0.780 |
| Root Trp | -0.299 | ##### | 0.433 | ##### | 0.416 | -0.485 | ##### | ##### | -0.381 | ##### | -0.409 | -0.546 | -0.610 | -0.510 | -0.435 | ##### | -0.528 | -0.755 | -0.141 | -0.691 | -0.477 | ##### | 0.469 | -0.576 | -0.278 | ##### | -0.380 | -0.494 | #### | ##### | -0.501 | ##### | -0.542 | -0.577 | 0.534 | -0.552 | -0.524 | -0.458 | ##### | -0.623 | -0.366 | -0.455 | ##### | -0.489 | -0.629 | -0.651 | -0.403 | -0.527 | -0.538 | -0.494 | ##### | -0.378 | -0.508 | -0.535 | -0.141 | ##### | 0.224 | 0.301 | -0.449 | -0.457 | 0.384 | -0.128 0.531 -0.323 0.228 ##### 0.357 0.112 -0.436 -0.513 ##### -0.081 -0.613 -0.553 -0.527 ##### -0.466 0.489 ##### 0.480 -0.616 -0.525 -0.519 0.420 ##### 0.845 -0.494 0.565 -0.402 0.260 -0.485 -0.276 -0.376 0.250 -0.823 0.368 0.373 -0.474 -0.092 -0.143 -0.474 |
| Root Phe | 0.930 | 0.878 | ##### | 0.756 | -0.865 | 0.879 | 0.965 | 0.790 | 0.739 | 0.819 | 0.620 | 0.861 | 0.974 | 0.976 | 0.984 | 0.975 | 0.817 | 0.914 | 0.561 | 0.895 | 0.924 | 0.769 | ##### | 0.952 | 0.324 | 0.961 | 0.804 | 0.901 | #### | 0.744 | 0.861 | 0.500 | 0.925 | 0.889 | -0.932 | 0.915 | 0.772 | 0.600 | 0.963 | 0.950 | 0.586 | 0.759 | 0.676 | 0.691 | 0.940 | 0.855 | 0.786 | 0.763 | 0.773 | 0.689 | 0.695 | 0.734 | 0.690 | 0.820 | 0.208 | 0.279 | ##### | -0.617 | 0.723 | 0.724 | -0.658 | 0.229 -0.946 0.954 0.166 0.837 ##### -0.091 0.903 0.928 0.902 -0.758 0.953 0.919 0.872 0.950 0.924 0.461 0.410 ##### 0.888 0.865 0.774 0.542 0.460 -0.382 0.997 -0.984 0.639 0.316 0.911 0.557 0.657 -0.441 0.788 -0.631 -0.691 0.716 0.200 -0.628 0.843 |
| Root Tyr | 0.796 | 0.856 | ##### | 0.863 | -0.914 | 0.814 | 0.707 | 0.942 | 0.954 | 0.907 | 0.942 | 0.909 | 0.687 | 0.673 | 0.657 | 0.572 | 0.937 | 0.519 | 0.953 | 0.769 | 0.428 | 0.954 | ##### | 0.781 | 0.888 | 0.586 | 0.943 | 0.791 | #### | 0.953 | 0.768 | 0.934 | 0.823 | 0.883 | -0.846 | 0.828 | 0.836 | 0.975 | 0.799 | 0.756 | 0.969 | 0.923 | 0.790 | 0.851 | 0.745 | 0.883 | 0.854 | 0.828 | 0.946 | 0.864 | 0.933 | 0.946 | 0.879 | 0.828 | 0.889 | 0.916 | ##### | -0.798 | 0.978 | 0.969 | -0.981 | 0.887 -0.831 0.474 ##### 0.895 ##### -0.841 0.779 0.588 0.599 -0.570 0.659 0.523 0.915 0.823 0.859 0.451 0.913 ##### 0.823 0.876 0.945 0.063 0.950 -0.196 0.556 -0.546 0.975 ##### 0.793 0.987 0.969 -0.926 0.296 -0.969 -0.957 0.957 0.866 -0.487 0.873 |
| Root Lys | 0.934 | 0.963 | ##### | 0.872 | -0.985 | 0.943 | 0.934 | 0.965 | 0.946 | 0.970 | 0.885 | 0.992 | 0.923 | 0.908 | 0.895 | 0.853 | 0.986 | 0.811 | 0.833 | 0.942 | 0.740 | 0.961 | ##### | 0.965 | 0.701 | 0.848 | 0.969 | 0.940 | #### | 0.947 | 0.910 | 0.803 | 0.974 | 0.993 | -0.988 | 0.973 | 0.909 | 0.899 | 0.968 | 0.951 | 0.880 | 0.945 | 0.836 | 0.876 | 0.939 | 0.985 | 0.915 | 0.901 | 0.972 | 0.883 | 0.919 | 0.939 | 0.893 | 0.927 | 0.630 | 0.687 | ##### | -0.794 | 0.957 | 0.954 | -0.922 | 0.638 -0.985 0.765 ##### 0.956 ##### -0.542 0.929 0.838 0.815 -0.690 0.894 0.799 0.999 0.979 0.986 0.438 0.734 ##### 0.964 0.975 0.970 0.253 0.794 -0.382 0.846 -0.842 0.912 ##### 0.945 0.866 0.915 -0.776 0.628 -0.902 -0.918 0.945 0.610 -0.576 0.958 |
| Root Thr | 0.888 | 0.935 | ##### | 0.894 | -0.974 | 0.904 | 0.840 | 0.979 | 0.976 | 0.964 | 0.939 | 0.974 | 0.825 | 0.813 | 0.799 | 0.733 | 0.985 | 0.680 | 0.915 | 0.877 | 0.604 | 0.983 | ##### | 0.895 | 0.817 | 0.740 | 0.982 | 0.891 | #### | 0.976 | 0.865 | 0.894 | 0.922 | 0.961 | -0.940 | 0.925 | 0.898 | 0.960 | 0.906 | 0.871 | 0.949 | 0.960 | 0.838 | 0.889 | 0.859 | 0.956 | 0.911 | 0.890 | 0.983 | 0.899 | 0.952 | 0.969 | 0.911 | 0.903 | 0.778 | 0.822 | ##### | -0.824 | 0.992 | 0.986 | -0.977 | 0.783 -0.930 0.641 ##### 0.952 ##### -0.707 0.880 0.735 0.732 -0.657 0.791 0.682 0.980 0.922 0.946 0.468 0.848 ##### 0.916 0.950 0.982 0.168 0.897 -0.295 0.719 -0.713 0.968 ##### 0.894 0.950 0.967 -0.875 0.470 -0.961 -0.957 0.976 0.758 -0.559 0.941 |
| Root 5-hydroxy-tryptamine | -0.199 | ##### | 0.503 | ##### | 0.417 | -0.291 | ##### | ##### | -0.573 | ##### | -0.665 | -0.430 | -0.066 | -0.035 | 0.000 | 0.083 | -0.501 | 0.070 | -0.696 | -0.246 | 0.223 | ##### | 0.452 | -0.197 | -0.842 | 0.062 | -0.501 | -0.244 | #### | ##### | -0.253 | ##### | -0.268 | -0.377 | 0.289 | -0.284 | -0.417 | -0.729 | ##### | -0.172 | -0.722 | -0.526 | ##### | -0.503 | -0.167 | -0.416 | -0.412 | -0.416 | -0.552 | -0.520 | ##### | -0.567 | -0.540 | -0.366 | -0.927 | ##### | 0.991 | 0.480 | -0.624 | -0.613 | 0.676 | -0.905 0.257 0.212 0.978 ##### 0.717 0.967 -0.220 0.026 0.011 0.028 -0.049 0.093 -0.425 ##### -0.303 -0.092 ##### 0.033 -0.309 -0.383 -0.547 0.450 ##### 0.013 0.130 -0.120 -0.689 0.887 -0.237 -0.758 -0.662 0.784 0.224 0.684 0.614 -0.608 -0.901 0.033 -0.393 |
| Root L-homocitrulline | 0.813 | 0.860 | ##### | 0.729 | -0.900 | 0.837 | 0.858 | 0.892 | 0.880 | 0.908 | 0.846 | 0.937 | 0.834 | 0.784 | 0.752 | 0.729 | 0.940 | 0.762 | 0.798 | 0.891 | 0.585 | 0.894 | ##### | 0.879 | 0.707 | 0.701 | 0.891 | 0.830 | #### | 0.878 | 0.801 | 0.750 | 0.886 | 0.938 | -0.909 | 0.889 | 0.834 | 0.894 | 0.869 | 0.907 | 0.852 | 0.880 | 0.768 | 0.813 | 0.905 | 0.956 | 0.810 | 0.827 | 0.938 | 0.824 | 0.865 | 0.869 | 0.843 | 0.846 | 0.663 | 0.713 | ##### | -0.688 | 0.924 | 0.917 | -0.887 | 0.654 -0.910 0.577 ##### 0.849 ##### -0.614 0.801 0.701 0.637 -0.452 0.849 0.670 0.946 0.902 0.894 0.199 0.681 ##### 0.898 0.898 0.929 0.027 0.766 -0.440 0.724 -0.729 0.882 ##### 0.833 0.844 0.871 -0.748 0.617 -0.862 -0.918 0.907 0.620 -0.331 0.869 |
| Root 3-N-methyl-L-histidine | -0.877 | ##### | 0.985 | ##### | 0.976 | -0.976 | ##### | ##### | -0.968 | ##### | -0.940 | -0.997 | -0.913 | -0.906 | -0.884 | ##### | -0.996 | -0.808 | -0.796 | -0.963 | -0.762 | ##### | 0.992 | -0.961 | -0.779 | ##### | -0.972 | -0.969 | #### | ##### | -0.959 | ##### | -0.980 | -0.992 | 0.975 | -0.983 | -0.972 | -0.935 | ##### | -0.896 | -0.920 | -0.984 | ##### | -0.952 | -0.876 | -0.992 | -0.966 | -0.967 | -0.993 | -0.956 | ##### | -0.968 | -0.962 | -0.979 | -0.668 | ##### | 0.547 | 0.886 | -0.970 | -0.975 | 0.945 | -0.685 0.955 -0.762 0.330 ##### 0.922 0.561 -0.956 -0.870 ##### 0.711 -0.820 -0.833 -0.985 ##### -0.970 -0.423 ##### 0.891 -0.986 -0.995 -0.993 -0.140 ##### 0.488 -0.804 0.823 -0.944 0.256 -0.968 -0.876 -0.947 0.837 -0.626 0.937 0.857 -0.977 -0.661 0.638 -0.989 |
| Root L-cystathionine | -0.638 | ##### | 0.903 | ##### | 0.828 | -0.784 | ##### | ##### | -0.932 | ##### | -0.975 | -0.829 | -0.562 | -0.573 | -0.550 | ##### | -0.872 | -0.388 | -0.873 | -0.693 | -0.366 | ##### | 0.858 | -0.675 | -0.989 | ##### | -0.884 | -0.750 | #### | ##### | -0.761 | ##### | -0.740 | -0.788 | 0.733 | -0.752 | -0.856 | -0.975 | ##### | -0.563 | -0.986 | -0.916 | ##### | -0.902 | -0.537 | -0.796 | -0.870 | -0.854 | -0.901 | -0.910 | ##### | -0.934 | -0.917 | -0.826 | -0.948 | ##### | 0.873 | 0.900 | -0.933 | -0.937 | 0.963 | -0.961 0.690 -0.389 0.675 ##### 0.983 0.880 -0.742 -0.543 ##### 0.583 -0.433 -0.473 -0.809 ##### -0.752 -0.469 ##### 0.601 -0.760 -0.824 -0.904 0.102 ##### 0.253 -0.388 0.407 -0.972 0.645 -0.743 -0.967 -0.967 0.998 -0.161 0.975 0.804 -0.941 -0.954 0.580 -0.844 |
| Root N6-acetyl-L-lysine | 0.600 | 0.733 | ##### | 0.794 | -0.779 | 0.691 | 0.483 | 0.852 | 0.890 | 0.801 | 0.929 | 0.767 | 0.463 | 0.470 | 0.453 | 0.349 | 0.817 | 0.269 | 0.908 | 0.592 | 0.229 | 0.871 | ##### | 0.587 | 0.976 | 0.393 | 0.843 | 0.653 | #### | 0.886 | 0.655 | 0.973 | 0.656 | 0.721 | -0.664 | 0.667 | 0.763 | 0.947 | 0.592 | 0.504 | 0.960 | 0.853 | 0.761 | 0.817 | 0.484 | 0.726 | 0.788 | 0.759 | 0.847 | 0.829 | 0.892 | 0.888 | 0.840 | 0.731 | 0.986 | 0.996 | ##### | -0.818 | 0.901 | 0.897 | -0.938 | 0.990 -0.628 0.274 ##### 0.795 ##### -0.945 0.647 0.416 0.466 -0.513 0.375 0.338 0.758 0.611 0.689 0.473 0.976 ##### 0.669 0.747 0.850 -0.095 0.990 -0.111 0.295 -0.298 0.942 ##### 0.650 0.972 0.935 -0.982 0.033 -0.945 -0.824 0.893 0.983 -0.497 0.766 |
| Root L-tyrosine-methyl-ester | -0.234 | ##### | 0.615 | ##### | 0.486 | -0.437 | ##### | ##### | -0.667 | ##### | -0.779 | -0.494 | -0.142 | -0.152 | -0.120 | ##### | -0.563 | 0.017 | -0.678 | -0.331 | 0.047 | ##### | 0.533 | -0.277 | -0.943 | ##### | -0.579 | -0.388 | #### | ##### | -0.423 | ##### | -0.363 | -0.434 | 0.350 | -0.381 | -0.575 | -0.787 | ##### | -0.149 | -0.805 | -0.642 | ##### | -0.668 | -0.124 | -0.462 | -0.579 | -0.578 | -0.618 | -0.677 | ##### | -0.672 | -0.684 | -0.516 | -0.948 | ##### | 0.957 | 0.701 | -0.677 | -0.681 | 0.742 | -0.952 0.292 0.044 0.832 ##### 0.795 0.928 -0.378 -0.148 ##### 0.265 -0.001 -0.077 -0.462 ##### -0.372 -0.275 ##### 0.220 -0.407 -0.487 -0.621 0.407 ##### 0.119 0.068 -0.036 -0.763 0.888 -0.375 -0.793 -0.748 0.892 0.202 0.770 0.537 -0.691 -0.959 0.331 -0.521 |
| Root N-acetylaspartate | 0.986 | 0.956 | ##### | 0.953 | -0.970 | 0.934 | 0.895 | 0.933 | 0.908 | 0.914 | 0.811 | 0.932 | 0.902 | 0.930 | 0.947 | 0.874 | 0.916 | 0.742 | 0.811 | 0.864 | 0.799 | 0.924 | ##### | 0.938 | 0.597 | 0.896 | 0.948 | 0.937 | #### | 0.913 | 0.901 | 0.769 | 0.948 | 0.933 | -0.958 | 0.941 | 0.858 | 0.790 | 0.975 | 0.894 | 0.803 | 0.896 | 0.775 | 0.814 | 0.876 | 0.888 | 0.907 | 0.847 | 0.887 | 0.816 | 0.858 | 0.904 | 0.817 | 0.885 | 0.528 | 0.580 | ##### | -0.791 | 0.883 | 0.881 | -0.854 | 0.552 -0.958 0.877 ##### 0.963 ##### -0.416 0.949 0.866 0.901 -0.886 0.849 0.819 0.943 0.960 0.976 0.699 0.722 ##### 0.904 0.934 0.893 0.538 0.737 -0.191 0.885 -0.858 0.835 0.081 0.946 0.802 0.854 -0.709 0.525 -0.837 -0.842 0.868 0.531 -0.779 0.933 |
| Root glycyl-L-proline | 0.968 | 0.888 | ##### | 0.908 | -0.955 | 0.843 | 0.840 | 0.917 | 0.898 | 0.882 | 0.800 | 0.922 | 0.832 | 0.826 | 0.836 | 0.754 | 0.918 | 0.672 | 0.907 | 0.812 | 0.617 | 0.919 | ##### | 0.883 | 0.624 | 0.750 | 0.939 | 0.841 | #### | 0.902 | 0.787 | 0.771 | 0.892 | 0.920 | -0.931 | 0.886 | 0.776 | 0.828 | 0.945 | 0.914 | 0.824 | 0.861 | 0.674 | 0.740 | 0.911 | 0.889 | 0.818 | 0.762 | 0.893 | 0.750 | 0.832 | 0.884 | 0.764 | 0.802 | 0.631 | 0.669 | ##### | -0.675 | 0.910 | 0.895 | -0.880 | 0.631 -0.946 0.703 ##### 0.911 ##### -0.571 0.841 0.711 0.720 -0.698 0.872 0.658 0.953 0.956 0.946 0.568 0.749 ##### 0.851 0.888 0.892 0.440 0.772 -0.100 0.801 -0.757 0.856 ##### 0.854 0.865 0.863 -0.721 0.459 -0.849 -0.968 0.869 0.602 -0.547 0.870 |
| Root N8-acetylspermidine | 0.902 | 0.959 | ##### | 0.964 | -0.982 | 0.930 | 0.814 | 0.994 | 0.994 | 0.966 | 0.955 | 0.960 | 0.808 | 0.826 | 0.825 | 0.739 | 0.972 | 0.627 | 0.921 | 0.848 | 0.639 | 0.996 | ##### | 0.885 | 0.836 | 0.771 | 0.997 | 0.915 | #### | 0.996 | 0.896 | 0.938 | 0.922 | 0.941 | -0.929 | 0.924 | 0.918 | 0.948 | 0.902 | 0.815 | 0.960 | 0.976 | 0.868 | 0.915 | 0.793 | 0.919 | 0.954 | 0.911 | 0.968 | 0.920 | 0.969 | 0.992 | 0.926 | 0.920 | 0.784 | 0.825 | ##### | -0.895 | 0.984 | 0.983 | -0.981 | 0.801 -0.910 0.699 ##### 0.986 ##### -0.689 0.918 0.766 0.804 -0.795 0.726 0.704 0.960 0.902 0.948 0.635 0.900 ##### 0.905 0.954 0.973 0.270 0.924 -0.223 0.714 -0.704 0.972 ##### 0.918 0.955 0.980 -0.907 0.387 -0.974 -0.910 0.975 0.785 -0.723 0.961 |
| Root methionine-sulfoxide | 0.592 | 0.711 | ##### | 0.778 | -0.767 | 0.666 | 0.466 | 0.838 | 0.876 | 0.784 | 0.913 | 0.755 | 0.444 | 0.446 | 0.429 | 0.324 | 0.806 | 0.251 | 0.914 | 0.574 | 0.194 | 0.859 | ##### | 0.570 | 0.966 | 0.363 | 0.830 | 0.628 | #### | 0.872 | 0.626 | 0.960 | 0.638 | 0.708 | -0.650 | 0.648 | 0.737 | 0.939 | 0.580 | 0.501 | 0.950 | 0.835 | 0.731 | 0.791 | 0.484 | 0.715 | 0.762 | 0.732 | 0.836 | 0.804 | 0.874 | 0.872 | 0.817 | 0.705 | 0.990 | 0.997 | ##### | -0.785 | 0.893 | 0.887 | -0.929 | 0.989 -0.618 0.242 ##### 0.775 ##### -0.958 0.620 0.382 0.428 -0.477 0.375 0.305 0.749 0.602 0.675 0.449 0.968 ##### 0.649 0.729 0.837 -0.104 0.983 -0.087 0.278 -0.277 0.932 ##### 0.625 0.969 0.923 -0.970 0.019 -0.934 -0.836 0.880 0.982 -0.452 0.744 |
| Root Asp-Phe | 0.897 | 0.867 | ##### | 0.919 | -0.942 | 0.816 | 0.746 | 0.937 | 0.936 | 0.888 | 0.875 | 0.909 | 0.733 | 0.728 | 0.732 | 0.632 | 0.925 | 0.546 | 0.976 | 0.759 | 0.485 | 0.947 | ##### | 0.814 | 0.771 | 0.642 | 0.952 | 0.801 | #### | 0.939 | 0.757 | 0.878 | 0.844 | 0.891 | -0.880 | 0.842 | 0.788 | 0.908 | 0.869 | 0.820 | 0.910 | 0.891 | 0.710 | 0.782 | 0.814 | 0.869 | 0.831 | 0.776 | 0.916 | 0.794 | 0.884 | 0.924 | 0.809 | 0.796 | 0.797 | 0.823 | ##### | -0.733 | 0.953 | 0.938 | -0.945 | 0.795 -0.882 0.570 ##### 0.909 ##### -0.749 0.798 0.617 0.642 -0.656 0.748 0.550 0.933 0.886 0.901 0.571 0.872 ##### 0.815 0.872 0.916 0.296 0.889 -0.063 0.657 -0.620 0.927 ##### 0.809 0.954 0.928 -0.844 0.310 -0.922 -0.990 0.915 0.772 -0.534 0.864 |
| Root Nα-acetyl-L-arginine | -0.521 | ##### | 0.722 | ##### | 0.634 | -0.780 | ##### | ##### | -0.680 | ##### | -0.702 | -0.629 | -0.605 | -0.685 | -0.684 | ##### | -0.626 | -0.498 | -0.382 | -0.646 | -0.724 | ##### | 0.664 | -0.640 | -0.598 | ##### | -0.652 | -0.773 | #### | ##### | -0.819 | ##### | -0.680 | -0.613 | 0.613 | -0.687 | -0.801 | -0.591 | ##### | -0.403 | -0.634 | -0.728 | ##### | -0.805 | -0.352 | -0.588 | -0.821 | -0.807 | -0.632 | -0.791 | ##### | -0.703 | -0.769 | -0.788 | -0.391 | ##### | 0.215 | 0.887 | -0.598 | -0.629 | 0.612 | -0.451 0.551 -0.718 ##### ##### 0.626 0.218 -0.794 -0.777 ##### 0.842 -0.314 -0.748 -0.573 ##### -0.628 -0.604 ##### 0.815 -0.686 -0.707 -0.649 -0.179 ##### 0.425 -0.524 0.576 -0.628 0.125 -0.762 -0.520 -0.654 0.645 -0.339 0.650 0.291 -0.661 -0.462 0.926 -0.753 |
| Root N-glycyl-L-leucine | 0.829 | 0.688 | ##### | 0.803 | -0.811 | 0.616 | 0.605 | 0.781 | 0.777 | 0.710 | 0.687 | 0.760 | 0.588 | 0.567 | 0.583 | 0.472 | 0.775 | 0.403 | 0.939 | 0.580 | 0.295 | 0.797 | ##### | 0.663 | 0.596 | 0.461 | 0.812 | 0.605 | #### | 0.782 | 0.536 | 0.716 | 0.681 | 0.746 | -0.746 | 0.674 | 0.562 | 0.753 | 0.764 | 0.750 | 0.749 | 0.704 | 0.455 | 0.548 | 0.760 | 0.718 | 0.617 | 0.545 | 0.757 | 0.565 | 0.692 | 0.756 | 0.587 | 0.579 | 0.703 | 0.712 | ##### | -0.483 | 0.813 | 0.786 | -0.801 | 0.683 -0.773 0.411 ##### 0.745 ##### -0.706 0.603 0.410 0.429 -0.487 0.709 0.344 0.810 0.792 0.771 0.503 0.749 ##### 0.634 0.696 0.753 0.370 0.749 0.157 0.556 -0.487 0.771 ##### 0.621 0.846 0.769 -0.673 0.188 -0.762 -0.972 0.746 0.656 -0.320 0.675 |
| Root D-alanyl-D-alanine | 0.089 | 0.222 | ##### | 0.359 | -0.300 | 0.170 | ##### | 0.410 | 0.477 | 0.337 | 0.577 | 0.292 | -0.094 | -0.105 | -0.128 | ##### | 0.370 | -0.254 | 0.640 | 0.082 | -0.350 | 0.445 | ##### | 0.047 | 0.798 | ##### | 0.397 | 0.119 | #### | 0.470 | 0.133 | 0.691 | 0.128 | 0.231 | -0.146 | 0.144 | 0.301 | 0.628 | 0.058 | 0.001 | 0.641 | 0.419 | 0.341 | 0.401 | -0.006 | 0.260 | 0.315 | 0.300 | 0.425 | 0.416 | 0.494 | 0.473 | 0.433 | 0.243 | 0.905 | 0.871 | ##### | -0.419 | 0.514 | 0.503 | -0.584 | 0.887 -0.110 -0.318 ##### 0.312 ##### -0.952 0.106 -0.157 ##### -0.015 -0.125 -0.231 0.286 0.094 0.173 0.160 0.753 0.088 0.161 0.253 0.423 -0.423 0.745 0.139 -0.277 0.276 0.596 ##### 0.112 0.688 0.573 -0.735 -0.416 -0.599 -0.503 0.497 0.892 -0.047 0.276 |
| Root L-carnosine | 0.845 | 0.801 | ##### | 0.833 | -0.892 | 0.746 | 0.713 | 0.885 | 0.884 | 0.841 | 0.827 | 0.874 | 0.692 | 0.664 | 0.660 | 0.572 | 0.891 | 0.530 | 0.953 | 0.727 | 0.402 | 0.898 | ##### | 0.770 | 0.736 | 0.563 | 0.901 | 0.731 | #### | 0.886 | 0.682 | 0.819 | 0.793 | 0.859 | -0.840 | 0.792 | 0.725 | 0.884 | 0.825 | 0.816 | 0.871 | 0.837 | 0.641 | 0.719 | 0.817 | 0.849 | 0.753 | 0.713 | 0.885 | 0.734 | 0.831 | 0.868 | 0.755 | 0.734 | 0.785 | 0.808 | ##### | -0.640 | 0.922 | 0.903 | -0.908 | 0.771 -0.850 0.473 ##### 0.840 ##### -0.763 0.719 0.537 0.535 -0.514 0.752 0.477 0.904 0.855 0.853 0.431 0.815 ##### 0.773 0.821 0.880 0.206 0.846 -0.076 0.609 -0.572 0.890 ##### 0.740 0.925 0.884 -0.796 0.320 -0.879 -0.998 0.876 0.743 -0.375 0.802 |
| Root argininosuccinic acid | -0.689 | ##### | 0.932 | ##### | 0.866 | -0.826 | ##### | ##### | -0.956 | ##### | -0.987 | -0.864 | -0.616 | -0.630 | -0.610 | ##### | -0.901 | -0.440 | -0.884 | -0.734 | -0.429 | ##### | 0.892 | -0.724 | -0.977 | ##### | -0.915 | -0.795 | #### | ##### | -0.803 | ##### | -0.785 | -0.826 | 0.778 | -0.795 | -0.886 | -0.983 | ##### | -0.612 | -0.995 | -0.941 | ##### | -0.924 | -0.585 | -0.829 | -0.902 | -0.883 | -0.925 | -0.931 | ##### | -0.958 | -0.937 | -0.860 | -0.927 | ##### | 0.836 | 0.920 | -0.953 | -0.957 | 0.978 | -0.942 0.736 -0.456 0.625 ##### 0.992 0.850 -0.789 -0.599 ##### 0.636 -0.486 -0.531 -0.845 ##### -0.796 -0.505 ##### 0.655 -0.800 -0.861 -0.929 0.042 ##### 0.265 -0.453 0.470 -0.984 0.587 -0.789 -0.974 -0.982 0.996 -0.209 0.988 0.823 -0.960 -0.934 0.625 -0.880 |
| Root succinic acid | 0.614 | 0.745 | ##### | 0.827 | -0.785 | 0.705 | 0.477 | 0.858 | 0.896 | 0.801 | 0.930 | 0.762 | 0.462 | 0.483 | 0.472 | 0.360 | 0.811 | 0.253 | 0.908 | 0.583 | 0.253 | 0.876 | ##### | 0.588 | 0.974 | 0.415 | 0.850 | 0.667 | #### | 0.893 | 0.671 | 0.985 | 0.660 | 0.714 | -0.663 | 0.670 | 0.770 | 0.936 | 0.597 | 0.486 | 0.959 | 0.858 | 0.771 | 0.825 | 0.462 | 0.710 | 0.806 | 0.767 | 0.839 | 0.834 | 0.895 | 0.895 | 0.842 | 0.738 | 0.978 | 0.987 | ##### | -0.844 | 0.895 | 0.893 | -0.936 | 0.987 -0.624 0.310 ##### 0.811 ##### -0.925 0.667 0.435 0.503 -0.579 0.355 0.355 0.751 0.607 0.694 0.550 0.991 ##### 0.665 0.750 0.844 -0.035 0.995 -0.077 0.303 -0.304 0.939 ##### 0.664 0.969 0.936 -0.987 0.006 -0.946 -0.804 0.889 0.984 -0.572 0.775 |
| Root 5-aminovaleric acid | 0.988 | 0.949 | ##### | 0.889 | -0.967 | 0.929 | 0.950 | 0.918 | 0.885 | 0.917 | 0.784 | 0.951 | 0.950 | 0.953 | 0.959 | 0.912 | 0.929 | 0.831 | 0.777 | 0.912 | 0.821 | 0.908 | ##### | 0.971 | 0.546 | 0.910 | 0.933 | 0.937 | #### | 0.890 | 0.895 | 0.706 | 0.967 | 0.961 | -0.984 | 0.961 | 0.850 | 0.781 | 0.999 | 0.965 | 0.774 | 0.881 | 0.756 | 0.794 | 0.954 | 0.929 | 0.877 | 0.839 | 0.897 | 0.798 | 0.836 | 0.878 | 0.804 | 0.883 | 0.475 | 0.533 | ##### | -0.730 | 0.879 | 0.875 | -0.834 | 0.490 -0.993 0.875 ##### 0.939 ##### -0.374 0.938 0.879 0.875 -0.788 0.934 0.845 0.966 0.996 0.989 0.553 0.643 ##### 0.932 0.941 0.898 0.478 0.682 -0.286 0.932 -0.910 0.815 0.095 0.947 0.770 0.828 -0.653 0.647 -0.808 -0.864 0.860 0.462 -0.656 0.925 |
| Root α-aminoadipic acid | -0.873 | ##### | 0.995 | ##### | 0.979 | -0.943 | ##### | ##### | -0.999 | ##### | -0.976 | -0.975 | -0.823 | -0.831 | -0.820 | ##### | -0.988 | -0.664 | -0.895 | -0.882 | -0.650 | ##### | 0.989 | -0.898 | -0.861 | ##### | -0.995 | -0.927 | #### | ##### | -0.915 | ##### | -0.934 | -0.956 | 0.935 | -0.938 | -0.947 | -0.972 | ##### | -0.822 | -0.974 | -0.992 | ##### | -0.946 | -0.800 | -0.946 | -0.966 | -0.941 | -0.989 | -0.952 | ##### | -0.998 | -0.958 | -0.946 | -0.791 | ##### | 0.659 | 0.912 | -0.995 | -0.997 | 0.990 | -0.808 0.912 -0.685 0.435 ##### 0.977 0.695 -0.923 -0.782 ##### 0.750 -0.729 -0.725 -0.968 ##### -0.947 -0.551 ##### 0.818 -0.930 -0.970 -0.992 -0.173 ##### 0.320 -0.709 0.713 -0.986 0.332 -0.928 -0.954 -0.991 0.919 -0.440 0.985 0.900 -0.994 -0.790 0.686 -0.974 |
| Root 2-aminobutyric acid | 0.648 | 0.759 | ##### | 0.849 | -0.805 | 0.715 | 0.498 | 0.871 | 0.906 | 0.811 | 0.930 | 0.777 | 0.483 | 0.503 | 0.496 | 0.380 | 0.824 | 0.269 | 0.930 | 0.593 | 0.268 | 0.889 | ##### | 0.607 | 0.962 | 0.432 | 0.866 | 0.679 | #### | 0.904 | 0.677 | 0.986 | 0.677 | 0.731 | -0.684 | 0.685 | 0.771 | 0.938 | 0.625 | 0.516 | 0.961 | 0.864 | 0.762 | 0.820 | 0.493 | 0.723 | 0.811 | 0.766 | 0.848 | 0.830 | 0.898 | 0.904 | 0.839 | 0.742 | 0.973 | 0.982 | ##### | -0.837 | 0.905 | 0.902 | -0.944 | 0.981 -0.650 0.333 ##### 0.826 ##### -0.922 0.680 0.447 0.516 -0.597 0.390 0.365 0.770 0.636 0.716 0.574 0.995 ##### 0.677 0.762 0.853 0.011 0.996 -0.052 0.333 -0.328 0.944 ##### 0.677 0.978 0.942 -0.983 0.016 -0.951 -0.831 0.895 0.976 -0.578 0.785 |
| Root 4-acetamidobutyric acid | -0.713 | ##### | 0.619 | ##### | 0.670 | -0.782 | ##### | ##### | -0.539 | ##### | -0.453 | -0.713 | -0.924 | -0.917 | -0.903 | ##### | -0.655 | -0.968 | -0.230 | -0.857 | -0.971 | ##### | 0.672 | -0.861 | -0.143 | ##### | -0.596 | -0.814 | #### | ##### | -0.801 | ##### | -0.817 | -0.756 | 0.795 | -0.811 | -0.696 | -0.413 | ##### | -0.833 | -0.382 | -0.607 | ##### | -0.606 | -0.819 | -0.746 | -0.664 | -0.694 | -0.616 | -0.597 | ##### | -0.543 | -0.591 | -0.743 | 0.055 | ##### | ##### | 0.517 | -0.520 | -0.534 | 0.442 | 0.026 0.799 -0.924 ##### ##### 0.384 -0.188 -0.805 -0.931 ##### 0.596 -0.852 -0.958 -0.701 ##### -0.762 -0.187 ##### 0.897 -0.811 -0.741 -0.616 -0.352 ##### 0.645 -0.942 0.971 -0.437 ##### -0.816 -0.291 -0.453 0.232 -0.938 0.425 0.405 -0.545 0.053 0.513 -0.716 |
| Root 6-aminocaproic acid | 0.285 | 0.467 | ##### | 0.595 | -0.502 | 0.429 | 0.137 | 0.614 | 0.675 | 0.546 | 0.765 | 0.480 | 0.120 | 0.148 | 0.134 | 0.019 | 0.549 | -0.080 | 0.732 | 0.283 | -0.061 | 0.640 | ##### | 0.262 | 0.927 | 0.090 | 0.597 | 0.380 | #### | 0.670 | 0.406 | 0.869 | 0.352 | 0.417 | -0.345 | 0.367 | 0.546 | 0.767 | 0.258 | 0.133 | 0.801 | 0.633 | 0.592 | 0.637 | 0.107 | 0.425 | 0.578 | 0.546 | 0.597 | 0.646 | 0.699 | 0.680 | 0.652 | 0.490 | 0.958 | 0.943 | ##### | -0.697 | 0.672 | 0.673 | -0.744 | 0.965 -0.292 -0.017 ##### 0.551 ##### -0.937 0.381 0.130 0.219 -0.357 -0.012 0.048 0.457 0.270 0.382 0.430 0.905 ##### 0.375 0.474 0.604 -0.254 0.888 0.038 -0.063 0.051 0.758 ##### 0.371 0.812 0.749 -0.895 -0.297 -0.771 -0.560 0.677 0.975 -0.415 0.514 |
| Root kynurenic acid | 0.214 | 0.217 | ##### | 0.485 | -0.330 | 0.142 | ##### | 0.409 | 0.468 | 0.290 | 0.508 | 0.264 | -0.112 | -0.095 | -0.082 | ##### | 0.335 | -0.341 | 0.734 | -0.006 | -0.360 | 0.445 | ##### | 0.032 | 0.699 | ##### | 0.418 | 0.095 | #### | 0.467 | 0.081 | 0.678 | 0.111 | 0.201 | -0.149 | 0.118 | 0.212 | 0.554 | 0.112 | 0.012 | 0.593 | 0.374 | 0.215 | 0.298 | 0.009 | 0.190 | 0.282 | 0.206 | 0.368 | 0.314 | 0.435 | 0.459 | 0.330 | 0.170 | 0.867 | 0.819 | ##### | -0.355 | 0.487 | 0.466 | -0.559 | 0.849 -0.133 -0.246 ##### 0.336 ##### -0.918 0.104 -0.187 ##### -0.172 -0.094 -0.278 0.282 0.135 0.201 0.435 0.786 0.115 0.097 0.219 0.372 -0.076 0.727 0.455 -0.223 0.269 0.553 ##### 0.098 0.693 0.542 -0.683 -0.554 -0.563 -0.559 0.443 0.857 -0.165 0.246 |
| Root 2-aminoethanesulfonic acid | 0.956 | 0.924 | ##### | 0.820 | -0.949 | 0.906 | 0.962 | 0.899 | 0.864 | 0.911 | 0.771 | 0.953 | 0.955 | 0.934 | 0.928 | 0.899 | 0.931 | 0.868 | 0.755 | 0.931 | 0.790 | 0.890 | ##### | 0.970 | 0.536 | 0.879 | 0.911 | 0.914 | #### | 0.868 | 0.870 | 0.670 | 0.959 | 0.968 | -0.982 | 0.954 | 0.836 | 0.788 | 0.987 | 0.993 | 0.763 | 0.866 | 0.740 | 0.779 | 0.988 | 0.952 | 0.842 | 0.825 | 0.903 | 0.785 | 0.822 | 0.855 | 0.795 | 0.870 | 0.469 | 0.529 | ##### | -0.680 | 0.877 | 0.871 | -0.825 | 0.475 -0.994 0.821 ##### 0.904 ##### -0.384 0.904 0.853 0.816 -0.672 0.966 0.826 0.971 0.996 0.975 0.408 0.597 ##### 0.937 0.932 0.900 0.371 0.658 -0.363 0.922 -0.907 0.807 0.042 0.923 0.757 0.813 -0.629 0.711 -0.794 -0.880 0.857 0.441 -0.528 0.905 |
| Root Cys | -0.034 | 0.079 | ##### | 0.023 | -0.162 | 0.045 | 0.008 | 0.235 | 0.277 | 0.228 | 0.374 | 0.230 | -0.041 | -0.132 | -0.192 | ##### | 0.285 | -0.043 | 0.393 | 0.135 | -0.351 | 0.265 | ##### | 0.041 | 0.539 | ##### | 0.217 | 0.011 | #### | 0.267 | 0.013 | 0.372 | 0.075 | 0.201 | -0.107 | 0.090 | 0.172 | 0.481 | 0.012 | 0.114 | 0.427 | 0.253 | 0.191 | 0.237 | 0.131 | 0.276 | 0.108 | 0.172 | 0.336 | 0.257 | 0.309 | 0.265 | 0.285 | 0.133 | 0.631 | 0.616 | ##### | -0.126 | 0.373 | 0.359 | -0.394 | 0.586 -0.098 -0.411 ##### 0.109 ##### -0.723 -0.039 -0.199 ##### 0.398 0.041 -0.224 0.233 0.083 0.086 -0.396 0.383 0.175 0.142 0.159 0.318 -0.674 0.453 -0.171 -0.222 0.198 0.409 ##### 0.003 0.458 0.366 -0.447 -0.065 -0.386 -0.433 0.353 0.569 0.424 0.135 |
| Root creatine | 0.846 | 0.812 | ##### | 0.791 | -0.766 | 0.819 | 0.799 | 0.709 | 0.667 | 0.711 | 0.554 | 0.718 | 0.827 | 0.890 | 0.923 | 0.880 | 0.678 | 0.710 | 0.481 | 0.729 | 0.898 | 0.685 | ##### | 0.816 | 0.291 | 0.917 | 0.722 | 0.837 | #### | 0.675 | 0.815 | 0.499 | 0.809 | 0.733 | -0.793 | 0.798 | 0.708 | 0.480 | 0.838 | 0.725 | 0.511 | 0.683 | 0.640 | 0.642 | 0.698 | 0.669 | 0.765 | 0.701 | 0.634 | 0.632 | 0.625 | 0.673 | 0.619 | 0.746 | 0.158 | 0.217 | 0.084 | -0.668 | 0.600 | 0.610 | -0.564 | 0.203 -0.789 0.968 0.360 0.792 ##### -0.007 0.866 0.885 0.942 -0.942 0.715 0.862 0.716 0.792 0.811 0.732 0.434 ##### 0.752 0.760 0.647 0.700 0.428 -0.197 0.878 -0.860 0.546 0.407 0.845 0.473 0.582 -0.420 0.547 -0.558 -0.498 0.609 0.193 -0.878 0.771 |
| Root GS | 0.234 | 0.480 | ##### | 0.549 | -0.482 | 0.459 | 0.159 | 0.606 | 0.667 | 0.561 | 0.779 | 0.481 | 0.143 | 0.172 | 0.147 | 0.056 | 0.548 | -0.027 | 0.648 | 0.324 | 0.001 | 0.627 | ##### | 0.277 | 0.938 | 0.130 | 0.577 | 0.410 | #### | 0.660 | 0.453 | 0.856 | 0.367 | 0.420 | -0.342 | 0.385 | 0.594 | 0.766 | 0.239 | 0.114 | 0.796 | 0.647 | 0.661 | 0.687 | 0.082 | 0.439 | 0.607 | 0.598 | 0.602 | 0.693 | 0.715 | 0.676 | 0.694 | 0.534 | 0.920 | 0.914 | ##### | -0.747 | 0.658 | 0.667 | -0.729 | 0.934 -0.278 0.011 ##### 0.546 ##### -0.880 0.408 0.188 0.265 -0.352 -0.037 0.116 0.443 0.246 0.370 0.361 0.865 ##### 0.406 0.490 0.609 -0.350 0.865 -0.112 -0.059 0.023 0.750 ##### 0.397 0.771 0.742 -0.893 -0.221 -0.764 -0.482 0.682 0.947 -0.440 0.534 |
| Root malate | 0.797 | 0.931 | ##### | 0.872 | -0.943 | 0.912 | 0.787 | 0.979 | 0.988 | 0.971 | 0.992 | 0.954 | 0.772 | 0.770 | 0.747 | 0.685 | 0.975 | 0.628 | 0.877 | 0.864 | 0.581 | 0.983 | ##### | 0.855 | 0.913 | 0.711 | 0.968 | 0.891 | #### | 0.986 | 0.889 | 0.952 | 0.897 | 0.932 | -0.894 | 0.904 | 0.945 | 0.995 | 0.833 | 0.773 | 0.990 | 0.986 | 0.919 | 0.957 | 0.751 | 0.936 | 0.948 | 0.941 | 0.988 | 0.963 | 0.993 | 0.988 | 0.971 | 0.934 | 0.841 | 0.884 | ##### | -0.915 | 0.994 | 0.996 | -0.994 | 0.854 -0.864 0.595 ##### 0.948 ##### -0.754 0.880 0.730 0.738 -0.661 0.668 0.674 0.940 0.845 0.901 0.454 0.896 ##### 0.909 0.947 0.989 0.025 0.947 -0.378 0.627 -0.644 0.997 ##### 0.888 0.962 0.995 -0.949 0.416 -0.994 -0.880 0.998 0.837 -0.614 0.951 |
| Root citrate | -0.784 | ##### | 0.969 | ##### | 0.928 | -0.882 | ##### | ##### | -0.987 | ##### | -0.992 | -0.922 | -0.709 | -0.721 | -0.706 | ##### | -0.949 | -0.534 | -0.912 | -0.801 | -0.520 | ##### | 0.945 | -0.805 | -0.937 | ##### | -0.963 | -0.857 | #### | ##### | -0.854 | ##### | -0.856 | -0.892 | 0.856 | -0.863 | -0.915 | -0.990 | ##### | -0.713 | -0.998 | -0.972 | ##### | -0.937 | -0.689 | -0.888 | -0.936 | -0.911 | -0.963 | -0.944 | ##### | -0.987 | -0.951 | -0.900 | -0.885 | ##### | 0.774 | 0.920 | -0.985 | -0.986 | 0.997 | -0.900 0.823 -0.556 0.559 ##### 0.999 0.802 -0.852 -0.676 ##### 0.692 -0.601 -0.611 -0.910 ##### -0.873 -0.540 ##### 0.724 -0.861 -0.916 -0.967 -0.064 ##### 0.270 -0.568 0.576 -0.998 0.479 -0.855 -0.983 -0.999 0.975 -0.301 1.000 0.878 -0.985 -0.887 0.653 -0.928 |
| Root NAD-ME | 0.653 | 0.843 | ##### | 0.836 | -0.842 | 0.825 | 0.612 | 0.914 | 0.942 | 0.890 | 0.984 | 0.843 | 0.600 | 0.621 | 0.600 | 0.520 | 0.881 | 0.428 | 0.842 | 0.725 | 0.441 | 0.922 | ##### | 0.707 | 0.981 | 0.573 | 0.895 | 0.793 | #### | 0.938 | 0.810 | 0.989 | 0.771 | 0.804 | -0.755 | 0.782 | 0.893 | 0.969 | 0.677 | 0.571 | 0.984 | 0.935 | 0.904 | 0.935 | 0.540 | 0.808 | 0.908 | 0.893 | 0.908 | 0.940 | 0.963 | 0.947 | 0.943 | 0.864 | 0.912 | 0.941 | ##### | -0.944 | 0.932 | 0.939 | -0.960 | 0.932 -0.706 0.459 ##### 0.880 ##### -0.824 0.789 0.608 0.655 -0.650 0.440 0.542 0.816 0.681 0.773 0.508 0.952 ##### 0.790 0.850 0.914 -0.068 0.981 -0.298 0.431 -0.456 0.970 ##### 0.786 0.949 0.970 -0.995 0.201 -0.977 -0.768 0.948 0.927 -0.659 0.875 |
