# Supplementary material for: Adaptive responses of carbon and nitrogen metabolisms to nitrogen-deficiency in Citrus sinensis seedlings
Source: BMC Plant Biol. 2022 Jul 26;22:370. doi: 10.1186/s12870-022-03759-7 (PMC9316421; doi:10.1186/s12870-022-03759-7)
Supplement: Supplementary file 11 — Additional file 11: Table S6. Pearson correlation coefficient matrix for the mean values of 105 physiological parameters in Citrus sinensis roots. [file 12870_2022_3759_MOESM11_ESM.docx]

| **Additional file 11: Table S6.** Pearson correlation coefficient matrix for the mean values of 105 physiological parameters in *Citrus sinensis* roots | | | | | | | | |  |  |  |  |  |  |  |  |  |  |  |  |  |  |  |  |  |  |  |  |  |  |  |  |  |  |  |  |  |  |  |  |  |  |  |  |  |  |  |  |  |  |  |  |  |  |  |  |  |  |  |  |  |  |
| --- | --- | --- | --- | --- | --- | --- | --- | --- | --- | --- | --- | --- | --- | --- | --- | --- | --- | --- | --- | --- | --- | --- | --- | --- | --- | --- | --- | --- | --- | --- | --- | --- | --- | --- | --- | --- | --- | --- | --- | --- | --- | --- | --- | --- | --- | --- | --- | --- | --- | --- | --- | --- | --- | --- | --- | --- | --- | --- | --- | --- | --- | --- |
|  | Root C | Root N | Root C/N | C distribution in roots | N distribution in roots | Root NH_4_^+^-N | Root NO_3_^-^-N | Root NH_4_^+^-N/NO_3_^-^-N | Root TSP | Root Gly | Root Ala | Root Val | Root Leu | Root Met | Root lle | Root Pro | Root Ser | Root Trp | Root Phe | Root Tyr | Root Glu | Root Asp | Root Asn | Root Gln | Root Lys | Root Arg | Root Thr | Root L-citrulline | Root 5-hydroxy-tryptamine | Root L-homocitrulline | Root β-Ala | Root L-pipecolic-acid | Root 3-N-methyl-L-histidine | Root homoserine | Root L-cystathionine | Root N6-acetyl-L-lysine | Root trans-4-hydroxy-L-proline | Root L-ornithine | Root L-tyrosine-methyl-ester | Root N-acetylaspartate | Root (5-L-glutamyl)-L-amino-acid | Root glycyl-L-proline | Root trimethylamine-N-oxide | Root N8-acetylspermidine | Root glutathione-oxidized | Root methionine-sulfoxide | Root Asp-Phe | Root Nα-acetyl-L-arginine | Root N-glycyl-L-leucine | Root γ-glutamate-cysteine | Root Nα-acetyl-L-glutamine | Root N-acetyl-L-tyrosine | Root D-alanyl-D-alanine | Root homo-Arg | Root L-carnosine | Root glycylphenylalanine | Root S-(5-adenosy)-L-Homocysteine | Root argininosuccinic acid | Root succinic acid | Root 5-aminovaleric acid | Root α-aminoadipic acid | Root 2-aminobutyric acidRoot (S)-β-aminoisobutyric acid Root γ-aminobutyric acid Root 4-acetamidobutyric acid Root 6-Aminocaproic acid Root creatine-phosphate Root kynurenic acid Root N'-formylkynurenine Root 2-aminoethanesulfonic-acid Root ethanolamine Root Cys Root creatine Root N,N-dimethylglycine Root 5-hydroxylysine Root TFAADs Root TFAADs/N Root molar ratio of C/N in TFAADs Root TFAADs/C Root NR Root GOGAT Root GOT Root GPT Root GS Root glucose Root fructose Root sucrose Root total soluble sugars Root starch Root TNC Root sucrose/starch Root malate Root citrate Root isocitrate Root malate + citrate + isocitrate Root NADP-ME Root NAD-ME Root NADP-MDH Root NAD-MDH Leaf PEPC Root PEPP Root PK Root CS Root ACO Root NADP-IDH |
| Root C | ##### | 0.8888 | -0.7570 | -0.4712 | -0.5391 | 0.9229 | 0.9099 | -0.7203 | ##### | 0.8557 | 0.7008 | 0.1033 | 0.7238 | 0.4336 | 0.3945 | 0.8479 | 0.7961 | -0.5773 | 0.9911 | 0.6454 | 0.8145 | 0.7001 | 0.9600 | 0.9828 | 0.9034 | 0.9902 | 0.7931 | 0.9759 | 0.0046 | 0.8106 | 0.7818 | 0.9683 | -0.8681 | 0.6972 | -0.4824 | 0.3927 | 0.8211 | 0.9941 | -0.0413 | 0.8979 | 0.5919 | 0.8419 | -0.9248 | 0.7696 | 0.8893 | 0.3782 | 0.7205 | -0.5238 | 0.6117 | 0.4680 | 0.9952 | 0.7927 | -0.1652 | 0.9806 | 0.6860 | 0.3533 | 0.5845 | -0.5408 | 0.3912 | 0.9541 | -0.7751 | 0.4184 0.7338 0.6806 -0.9280 0.0303 0.6794 -0.1474 0.9071 0.9594 0.2783 -0.0872 0.8331 0.5728 0.7883 0.8947 0.8277 -0.9733 0.8553 0.8828 0.6792 0.9305 0.9446 0.0369 0.7019 0.7243 0.6980 0.7141 -0.6740 -0.5294 0.7201 0.7107 -0.6475 0.9438 -0.5688 0.7900 0.5159 0.8552 0.8098 0.9703 0.6705 0.9449 0.9631 0.7363 0.7637 |
| Root N | ##### | 1.0000 | -0.9717 | -0.8115 | -0.8571 | 0.8509 | 0.9845 | -0.9567 | ##### | 0.9843 | 0.8771 | 0.5380 | 0.8982 | 0.7683 | 0.7470 | 0.9913 | 0.9742 | -0.4708 | 0.8731 | 0.8977 | 0.9884 | 0.9223 | 0.9809 | 0.9110 | 0.9850 | 0.9350 | 0.9664 | 0.9663 | -0.3963 | 0.9033 | 0.9634 | 0.9701 | -0.9895 | 0.9299 | -0.8259 | 0.7632 | 0.9835 | 0.9311 | -0.4821 | 0.9586 | 0.8522 | 0.9281 | -0.9807 | 0.9725 | 0.9895 | 0.7476 | 0.9118 | -0.6715 | 0.7589 | 0.7955 | 0.8844 | 0.9613 | 0.2723 | 0.9545 | 0.8613 | 0.6688 | 0.8884 | -0.8637 | 0.7675 | 0.9613 | -0.9780 | 0.7842 0.9521 0.9300 -0.7122 0.4845 0.9005 0.2724 0.9924 0.9475 0.6622 0.1564 0.7703 0.8779 0.9818 0.9937 0.9781 -0.9688 0.9910 0.9387 0.9396 0.9793 0.9499 0.4820 0.9451 0.9566 0.9431 0.9519 -0.9372 -0.8526 0.9577 0.9469 -0.9237 0.9350 -0.8824 0.9542 0.8461 0.9957 0.9859 0.9481 0.9335 0.9856 0.9773 0.9521 0.9741 |
| Root C/N | ##### | ###### | 1.0000 | 0.9237 | 0.9514 | -0.7419 | -0.9331 | 0.9942 | ##### | -0.9704 | -0.9074 | -0.7210 | -0.9210 | -0.8859 | -0.8743 | -0.9828 | -0.9879 | 0.4034 | -0.7346 | -0.9592 | -0.9930 | -0.9464 | -0.9083 | -0.7912 | -0.9486 | -0.8299 | -0.9796 | -0.8796 | 0.5851 | -0.8904 | -0.9806 | -0.8860 | 0.9712 | -0.9726 | 0.9346 | -0.8929 | -0.9865 | -0.8213 | 0.6724 | -0.9006 | -0.9258 | -0.8966 | 0.9313 | -0.9918 | -0.9579 | -0.8797 | -0.9370 | 0.6679 | -0.7788 | -0.9022 | -0.7564 | -0.9561 | -0.4851 | -0.8625 | -0.8893 | -0.7823 | -0.9707 | 0.9574 | -0.8959 | -0.8820 | 0.9995 | -0.9056 -0.9872 -0.9814 0.5384 -0.6761 -0.9231 -0.4675 -0.9480 -0.8662 -0.8133 -0.3002 -0.6518 -0.9627 -0.9985 -0.9607 -0.9748 0.8858 -0.9775 -0.8832 -0.9908 -0.9169 -0.8636 -0.6687 -0.9913 -0.9968 -0.9928 -0.9956 0.9928 0.9397 -0.9963 -0.9918 0.9880 -0.8611 0.9673 -0.9511 -0.9429 -0.9846 -0.9891 -0.8588 -0.9845 -0.9205 -0.9022 -0.9779 -0.9969 |
| C distribution in roots | ##### | ###### | 0.9237 | 1.0000 | 0.9925 | -0.5016 | -0.7273 | 0.9217 | ##### | -0.8403 | -0.8792 | -0.9253 | -0.8639 | -0.9901 | -0.9850 | -0.8559 | -0.9027 | 0.3471 | -0.4268 | -0.9557 | -0.8768 | -0.8366 | -0.6901 | -0.5028 | -0.7940 | -0.5803 | -0.8970 | -0.6464 | 0.8435 | -0.8104 | -0.9025 | -0.6457 | 0.8402 | -0.9195 | 0.9941 | -0.9900 | -0.8790 | -0.5638 | 0.8942 | -0.6775 | -0.9565 | -0.7411 | 0.7588 | -0.8917 | -0.7947 | -0.9861 | -0.8658 | 0.5298 | -0.7241 | -0.9779 | -0.4876 | -0.8036 | -0.7747 | -0.6300 | -0.8469 | -0.8694 | -0.9836 | 0.9875 | -0.9789 | -0.6489 | 0.9128 | -0.9755 -0.9287 -0.9425 0.2145 -0.8801 -0.8026 -0.6983 -0.7547 -0.6525 -0.9695 -0.5729 -0.3340 -0.9781 -0.9045 -0.7949 -0.8556 0.6604 -0.8395 -0.6897 -0.9413 -0.6976 -0.6049 -0.8701 -0.9491 -0.9450 -0.9593 -0.9501 0.9576 0.9353 -0.9291 -0.9537 0.9687 -0.6758 0.9744 -0.8203 -0.9754 -0.8551 -0.8635 -0.6260 -0.9288 -0.7076 -0.6846 -0.8916 -0.9043 |
| N distribution in roots | ##### | ###### | 0.9514 | 0.9925 | 1.0000 | -0.5892 | -0.7898 | 0.9472 | ##### | -0.8660 | -0.8670 | -0.8911 | -0.8555 | -0.9775 | -0.9572 | -0.8861 | -0.9229 | 0.3738 | -0.4978 | -0.9494 | -0.9092 | -0.8869 | -0.7442 | -0.5813 | -0.8278 | -0.6368 | -0.9124 | -0.7076 | 0.7774 | -0.8205 | -0.9163 | -0.7078 | 0.8842 | -0.9240 | 0.9966 | -0.9724 | -0.9034 | -0.6266 | 0.8634 | -0.7277 | -0.9400 | -0.7543 | 0.7959 | -0.9190 | -0.8290 | -0.9621 | -0.8630 | 0.6205 | -0.6930 | -0.9863 | -0.5529 | -0.8546 | -0.7023 | -0.6907 | -0.8295 | -0.8191 | -0.9963 | 0.9968 | -0.9674 | -0.6967 | 0.9418 | -0.9634 -0.9396 -0.9486 0.3103 -0.8473 -0.8481 -0.6211 -0.8051 -0.6923 -0.9332 -0.4925 -0.4230 -0.9759 -0.9339 -0.8325 -0.8774 0.7166 -0.8706 -0.7679 -0.9671 -0.7497 -0.6729 -0.8520 -0.9767 -0.9700 -0.9754 -0.9736 0.9780 0.9462 -0.9587 -0.9753 0.9842 -0.7137 0.9847 -0.8819 -0.9914 -0.8962 -0.9012 -0.6719 -0.9566 -0.7690 -0.7438 -0.9380 -0.9385 |
| Root NH_4_^+^-N | ##### | 0.8509 | -0.7419 | -0.5016 | -0.5892 | 1.0000 | 0.8822 | -0.6913 | ##### | 0.7713 | 0.5951 | 0.1777 | 0.5858 | 0.4895 | 0.3820 | 0.7844 | 0.7454 | -0.6863 | 0.8898 | 0.5816 | 0.7674 | 0.7218 | 0.8934 | 0.9357 | 0.8321 | 0.9058 | 0.7263 | 0.9269 | -0.0208 | 0.7523 | 0.7118 | 0.9059 | -0.8705 | 0.6044 | -0.5287 | 0.3965 | 0.7571 | 0.9275 | -0.1556 | 0.7855 | 0.5351 | 0.6662 | -0.8464 | 0.7145 | 0.7971 | 0.3685 | 0.5770 | -0.6924 | 0.3710 | 0.5755 | 0.9364 | 0.7665 | -0.1389 | 0.9361 | 0.5292 | 0.2018 | 0.6163 | -0.5777 | 0.3961 | 0.8349 | -0.7528 | 0.4028 0.6697 0.6142 -0.9405 0.0980 0.6580 -0.2424 0.8420 0.8453 0.2880 -0.0565 0.7652 0.5383 0.7589 0.8141 0.7349 -0.9130 0.7792 0.9549 0.6816 0.8404 0.8732 0.1718 0.7337 0.7322 0.6913 0.7237 -0.6760 -0.5096 0.7192 0.7253 -0.6490 0.8695 -0.5742 0.8538 0.5839 0.8396 0.7697 0.8542 0.6694 0.9140 0.9240 0.7882 0.7516 |
| Root NO_3_^-^-N | ##### | 0.9845 | -0.9331 | -0.7273 | -0.7898 | 0.8822 | 1.0000 | -0.9241 | ##### | 0.9438 | 0.7822 | 0.4396 | 0.8185 | 0.6698 | 0.6420 | 0.9562 | 0.9216 | -0.4035 | 0.9101 | 0.8151 | 0.9598 | 0.9332 | 0.9791 | 0.9516 | 0.9494 | 0.9362 | 0.9083 | 0.9716 | -0.2547 | 0.8237 | 0.9047 | 0.9833 | -0.9613 | 0.8724 | -0.7579 | 0.6774 | 0.9404 | 0.9400 | -0.3887 | 0.9740 | 0.7525 | 0.8880 | -0.9503 | 0.9431 | 0.9647 | 0.6538 | 0.8480 | -0.7531 | 0.6753 | 0.7233 | 0.8945 | 0.9723 | 0.1425 | 0.9554 | 0.7728 | 0.5606 | 0.8312 | -0.8046 | 0.6962 | 0.9618 | -0.9414 | 0.7133 0.8941 0.8711 -0.7755 0.4036 0.9172 0.1666 0.9928 0.9264 0.5499 -0.0142 0.8652 0.8092 0.9458 0.9719 0.9365 -0.9666 0.9607 0.9621 0.9052 0.9862 0.9834 0.4124 0.9012 0.9102 0.8857 0.9035 -0.8915 -0.8168 0.9256 0.8930 -0.8716 0.8992 -0.8309 0.9613 0.7968 0.9695 0.9685 0.9394 0.9063 0.9925 0.9779 0.9388 0.9484 |
| Root NH_4_^+^-N/NO_3_^-^-N | ##### | ###### | 0.9942 | 0.9217 | 0.9472 | -0.6913 | -0.9241 | 1.0000 | ##### | -0.9562 | -0.8847 | -0.7366 | -0.9112 | -0.8718 | -0.8753 | -0.9705 | -0.9730 | 0.3026 | -0.7099 | -0.9520 | -0.9882 | -0.9629 | -0.8853 | -0.7672 | -0.9232 | -0.7957 | -0.9641 | -0.8502 | 0.5856 | -0.8455 | -0.9676 | -0.8658 | 0.9445 | -0.9781 | 0.9381 | -0.9044 | -0.9750 | -0.7859 | 0.6865 | -0.9058 | -0.9120 | -0.8926 | 0.9040 | -0.9958 | -0.9464 | -0.8901 | -0.9400 | 0.6851 | -0.7863 | -0.8889 | -0.7107 | -0.9678 | -0.5053 | -0.8264 | -0.8804 | -0.8032 | -0.9707 | 0.9609 | -0.9159 | -0.8680 | 0.9931 | -0.9266 -0.9825 -0.9850 0.4886 -0.7089 -0.9517 -0.5196 -0.9406 -0.8360 -0.8172 -0.2570 -0.6680 -0.9703 -0.9913 -0.9481 -0.9671 0.8558 -0.9684 -0.8562 -0.9960 -0.9096 -0.8574 -0.6922 -0.9815 -0.9872 -0.9840 -0.9862 0.9929 0.9669 -0.9972 -0.9783 0.9885 -0.8167 0.9780 -0.9424 -0.9479 -0.9668 -0.9894 -0.8329 -0.9950 -0.8995 -0.8736 -0.9732 -0.9961 |
| Root TSP | ##### | 0.9375 | -0.8455 | -0.5884 | -0.6692 | 0.9425 | 0.9781 | -0.8270 | ##### | 0.8719 | 0.6703 | 0.2694 | 0.7048 | 0.5341 | 0.4820 | 0.8853 | 0.8353 | -0.4511 | 0.9486 | 0.6886 | 0.8832 | 0.8659 | 0.9631 | 0.9867 | 0.8994 | 0.9448 | 0.8183 | 0.9728 | -0.0716 | 0.7586 | 0.8107 | 0.9797 | -0.9157 | 0.7543 | -0.6266 | 0.5205 | 0.8599 | 0.9580 | -0.2264 | 0.9376 | 0.6190 | 0.8106 | -0.9098 | 0.8536 | 0.9064 | 0.4916 | 0.7317 | -0.7667 | 0.5460 | 0.6104 | 0.9320 | 0.9144 | -0.0513 | 0.9624 | 0.6515 | 0.3810 | 0.7150 | -0.6817 | 0.5417 | 0.9393 | -0.8572 | 0.5583 0.7880 0.7525 -0.8832 0.2296 0.8421 -0.0412 0.9539 0.9055 0.3805 -0.1483 0.9076 0.6738 0.8637 0.9177 0.8542 -0.9612 0.8912 0.9721 0.8058 0.9570 0.9825 0.2604 0.8120 0.8189 0.7828 0.8099 -0.7874 -0.6878 0.8347 0.7994 -0.7605 0.8817 -0.7067 0.9243 0.6812 0.9134 0.8961 0.9256 0.8072 0.9800 0.9713 0.8737 0.8671 |
| Root Gly | ##### | 0.9843 | -0.9704 | -0.8403 | -0.8660 | 0.7713 | 0.9438 | -0.9562 | ##### | 1.0000 | 0.9427 | 0.5787 | 0.9609 | 0.8016 | 0.8050 | 0.9976 | 0.9911 | -0.4661 | 0.8358 | 0.9447 | 0.9868 | 0.8783 | 0.9613 | 0.8562 | 0.9922 | 0.9177 | 0.9918 | 0.9367 | -0.4854 | 0.9416 | 0.9905 | 0.9387 | -0.9725 | 0.9654 | -0.8383 | 0.8031 | 0.9966 | 0.9034 | -0.5108 | 0.9417 | 0.9115 | 0.9694 | -0.9856 | 0.9752 | 0.9953 | 0.7974 | 0.9648 | -0.5456 | 0.8572 | 0.7949 | 0.8542 | 0.9271 | 0.3589 | 0.9266 | 0.9370 | 0.7689 | 0.8935 | -0.8704 | 0.7970 | 0.9549 | -0.9766 | 0.8179 0.9786 0.9596 -0.6288 0.5173 0.8650 0.3747 0.9724 0.9548 0.7236 0.2692 0.6891 0.9096 0.9816 0.9939 0.9978 -0.9475 0.9973 0.8664 0.9331 0.9611 0.9076 0.4885 0.9366 0.9556 0.9549 0.9523 -0.9390 -0.8587 0.9491 0.9499 -0.9311 0.9424 -0.8912 0.8983 0.8383 0.9825 0.9748 0.9469 0.9233 0.9505 0.9486 0.9156 0.9626 |
| Root Ala | ##### | 0.8771 | -0.9074 | -0.8792 | -0.8670 | 0.5951 | 0.7822 | -0.8847 | ##### | 0.9427 | 1.0000 | 0.6778 | 0.9890 | 0.8712 | 0.8925 | 0.9284 | 0.9552 | -0.5201 | 0.6552 | 0.9740 | 0.9052 | 0.7299 | 0.8338 | 0.6609 | 0.9257 | 0.7926 | 0.9679 | 0.7978 | -0.6851 | 0.9657 | 0.9681 | 0.7855 | -0.8881 | 0.9462 | -0.8447 | 0.8523 | 0.9429 | 0.7639 | -0.6065 | 0.7880 | 0.9779 | 0.9238 | -0.9108 | 0.8973 | 0.9093 | 0.8641 | 0.9623 | -0.2960 | 0.9166 | 0.8103 | 0.7188 | 0.7759 | 0.5537 | 0.7973 | 0.9872 | 0.8798 | 0.8728 | -0.8561 | 0.8192 | 0.8286 | -0.9095 | 0.8381 0.9559 0.9414 -0.4173 0.5918 0.7110 0.5318 0.8389 0.8696 0.8345 0.5554 0.4149 0.9145 0.9134 0.9012 0.9436 -0.8224 0.9201 0.6918 0.8655 0.8191 0.7205 0.5398 0.8843 0.9076 0.9294 0.9085 -0.8925 -0.8062 0.8752 0.9166 -0.8979 0.8838 -0.8621 0.7508 0.8069 0.8941 0.8727 0.8393 0.8436 0.8046 0.8161 0.8059 0.8756 |
| Root Val | ##### | 0.5380 | -0.7210 | -0.9253 | -0.8911 | 0.1777 | 0.4396 | -0.7366 | ##### | 0.5787 | 0.6778 | 1.0000 | 0.6536 | 0.9269 | 0.9360 | 0.6021 | 0.6748 | -0.1263 | 0.0599 | 0.7953 | 0.6433 | 0.6597 | 0.3688 | 0.1534 | 0.5052 | 0.2286 | 0.6678 | 0.3131 | -0.9410 | 0.5537 | 0.6790 | 0.3178 | -0.5774 | 0.7367 | -0.9182 | 0.9482 | 0.6386 | 0.2101 | -0.9936 | 0.3862 | 0.8154 | 0.4689 | -0.4562 | 0.6804 | 0.5150 | 0.9477 | 0.6617 | -0.4042 | 0.5395 | 0.9054 | 0.1216 | 0.5794 | 0.9365 | 0.2909 | 0.6481 | 0.8180 | 0.8630 | -0.8867 | 0.9400 | 0.3237 | -0.7006 | 0.9237 0.7311 0.7708 0.1493 0.9852 0.6307 0.8452 0.4680 0.3195 0.9684 0.6562 0.0397 0.8580 0.6852 0.5138 0.6086 -0.3283 0.5809 0.4115 0.7776 0.3928 0.2867 0.9745 0.7742 0.7584 0.7835 0.7683 -0.7956 -0.8372 0.7457 0.7725 -0.8186 0.3499 -0.8607 0.5999 0.8869 0.6009 0.6330 0.2874 0.7690 0.3987 0.3619 0.7007 0.6990 |
| Root Leu | ##### | 0.8982 | -0.9210 | -0.8639 | -0.8555 | 0.5858 | 0.8185 | -0.9112 | ##### | 0.9609 | 0.9890 | 0.6536 | 1.0000 | 0.8355 | 0.8756 | 0.9472 | 0.9620 | -0.4147 | 0.6972 | 0.9736 | 0.9295 | 0.7733 | 0.8587 | 0.6974 | 0.9355 | 0.8117 | 0.9743 | 0.8176 | -0.6349 | 0.9379 | 0.9766 | 0.8177 | -0.8882 | 0.9715 | -0.8383 | 0.8492 | 0.9587 | 0.7825 | -0.5750 | 0.8477 | 0.9629 | 0.9627 | -0.9224 | 0.9285 | 0.9375 | 0.8584 | 0.9908 | -0.3376 | 0.9508 | 0.7787 | 0.7274 | 0.8247 | 0.5190 | 0.8092 | 0.9969 | 0.8963 | 0.8726 | -0.8561 | 0.8284 | 0.8691 | -0.9249 | 0.8522 0.9695 0.9605 -0.4279 0.5872 0.7721 0.5504 0.8764 0.8877 0.8104 0.4520 0.5028 0.9256 0.9297 0.9281 0.9673 -0.8405 0.9452 0.7005 0.8852 0.8630 0.7724 0.5220 0.8843 0.9109 0.9306 0.9107 -0.9036 -0.8424 0.8937 0.9125 -0.9063 0.8783 -0.8763 0.7681 0.8066 0.9049 0.9043 0.8661 0.8706 0.8291 0.8332 0.8195 0.8980 |
| Root Met | ##### | 0.7683 | -0.8859 | -0.9901 | -0.9775 | 0.4895 | 0.6698 | -0.8718 | ##### | 0.8016 | 0.8712 | 0.9269 | 0.8355 | 1.0000 | 0.9795 | 0.8155 | 0.8732 | -0.4360 | 0.3721 | 0.9343 | 0.8301 | 0.7695 | 0.6460 | 0.4497 | 0.7637 | 0.5446 | 0.8688 | 0.6074 | -0.8760 | 0.8177 | 0.8724 | 0.5949 | -0.8157 | 0.8739 | -0.9747 | 0.9700 | 0.8414 | 0.5283 | -0.9026 | 0.6013 | 0.9499 | 0.6877 | -0.7276 | 0.8385 | 0.7470 | 0.9694 | 0.8202 | -0.4657 | 0.6807 | 0.9814 | 0.4632 | 0.7330 | 0.7945 | 0.5990 | 0.8212 | 0.8391 | 0.9578 | -0.9617 | 0.9467 | 0.5925 | -0.8735 | 0.9392 0.8930 0.9017 -0.1909 0.8641 0.7205 0.6712 0.6970 0.6180 0.9717 0.6646 0.2351 0.9442 0.8647 0.7479 0.8117 -0.6233 0.7936 0.6564 0.8969 0.6356 0.5358 0.8645 0.9228 0.9163 0.9333 0.9225 -0.9215 -0.8768 0.8859 0.9320 -0.9353 0.6629 -0.9356 0.7771 0.9490 0.8206 0.8094 0.5834 0.8776 0.6622 0.6471 0.8516 0.8578 |
| Root lle | ##### | 0.7470 | -0.8743 | -0.9850 | -0.9572 | 0.3820 | 0.6420 | -0.8753 | ##### | 0.8050 | 0.8925 | 0.9360 | 0.8756 | 0.9795 | 1.0000 | 0.8121 | 0.8686 | -0.3058 | 0.3488 | 0.9531 | 0.8269 | 0.7543 | 0.6224 | 0.4070 | 0.7496 | 0.5156 | 0.8708 | 0.5695 | -0.9028 | 0.7949 | 0.8783 | 0.5684 | -0.7731 | 0.9069 | -0.9646 | 0.9894 | 0.8415 | 0.4898 | -0.8965 | 0.6173 | 0.9661 | 0.7335 | -0.7124 | 0.8475 | 0.7508 | 0.9950 | 0.8700 | -0.3857 | 0.7775 | 0.9355 | 0.4122 | 0.7279 | 0.8404 | 0.5544 | 0.8722 | 0.9296 | 0.9441 | -0.9510 | 0.9698 | 0.5956 | -0.8629 | 0.9698 0.9055 0.9243 -0.0992 0.8889 0.7309 0.7832 0.6880 0.6098 0.9919 0.6593 0.2271 0.9645 0.8555 0.7455 0.8251 -0.5908 0.7961 0.5753 0.8901 0.6334 0.5208 0.8537 0.8936 0.8959 0.9223 0.9024 -0.9132 -0.9026 0.8746 0.9080 -0.9300 0.6316 -0.9402 0.7221 0.9268 0.7924 0.8057 0.5764 0.8753 0.6259 0.6079 0.8113 0.8463 |
| Root Pro | ##### | 0.9913 | -0.9828 | -0.8559 | -0.8861 | 0.7844 | 0.9562 | -0.9705 | ##### | 0.9976 | 0.9284 | 0.6021 | 0.9472 | 0.8155 | 0.8121 | 1.0000 | 0.9933 | -0.4528 | 0.8290 | 0.9443 | 0.9951 | 0.9071 | 0.9609 | 0.8598 | 0.9883 | 0.9094 | 0.9906 | 0.9370 | -0.4896 | 0.9285 | 0.9895 | 0.9407 | -0.9811 | 0.9668 | -0.8600 | 0.8182 | 0.9985 | 0.8981 | -0.5396 | 0.9445 | 0.9080 | 0.9542 | -0.9800 | 0.9850 | 0.9936 | 0.8091 | 0.9561 | -0.5986 | 0.8294 | 0.8199 | 0.8455 | 0.9467 | 0.3688 | 0.9248 | 0.9191 | 0.7580 | 0.9133 | -0.8917 | 0.8164 | 0.9486 | -0.9877 | 0.8345 0.9816 0.9648 -0.6303 0.5459 0.8908 0.3771 0.9784 0.9419 0.7334 0.2517 0.7043 0.9201 0.9914 0.9940 0.9962 -0.9453 0.9990 0.8887 0.9521 0.9629 0.9140 0.5257 0.9546 0.9697 0.9659 0.9665 -0.9554 -0.8803 0.9665 0.9631 -0.9469 0.9299 -0.9106 0.9253 0.8656 0.9915 0.9867 0.9360 0.9440 0.9575 0.9508 0.9408 0.9782 |
| Root Ser | ##### | 0.9742 | -0.9879 | -0.9027 | -0.9229 | 0.7454 | 0.9216 | -0.9730 | ##### | 0.9911 | 0.9552 | 0.6748 | 0.9620 | 0.8732 | 0.8686 | 0.9933 | 1.0000 | -0.4781 | 0.7664 | 0.9729 | 0.9886 | 0.8912 | 0.9283 | 0.8024 | 0.9772 | 0.8698 | 0.9987 | 0.9007 | -0.5826 | 0.9452 | 0.9982 | 0.8997 | -0.9756 | 0.9771 | -0.8993 | 0.8667 | 0.9976 | 0.8557 | -0.6155 | 0.9018 | 0.9483 | 0.9377 | -0.9640 | 0.9806 | 0.9760 | 0.8609 | 0.9627 | -0.5613 | 0.8390 | 0.8671 | 0.8013 | 0.9215 | 0.4616 | 0.8906 | 0.9380 | 0.8029 | 0.9411 | -0.9230 | 0.8575 | 0.9095 | -0.9901 | 0.8723 0.9932 0.9794 -0.5626 0.6115 0.8683 0.4446 0.9488 0.9139 0.8004 0.3571 0.6207 0.9482 0.9921 0.9756 0.9911 -0.9122 0.9881 0.8593 0.9606 0.9255 0.8621 0.5911 0.9697 0.9828 0.9847 0.9813 -0.9701 -0.8942 0.9714 0.9813 -0.9663 0.9141 -0.9337 0.9106 0.8956 0.9843 0.9751 0.9005 0.9487 0.9249 0.9193 0.9394 0.9763 |
| Root Trp | ##### | ###### | 0.4034 | 0.3471 | 0.3738 | -0.6863 | -0.4035 | 0.3026 | ##### | -0.4661 | -0.5201 | -0.1263 | -0.4147 | -0.4360 | -0.3058 | -0.4528 | -0.4781 | 1.0000 | -0.4668 | -0.4051 | -0.3918 | -0.1904 | -0.5155 | -0.4791 | -0.5529 | -0.5833 | -0.4809 | -0.5593 | 0.2114 | -0.7005 | -0.4609 | -0.4823 | 0.5697 | -0.3008 | 0.3024 | -0.2219 | -0.4483 | -0.5890 | 0.1205 | -0.2731 | -0.4502 | -0.3552 | 0.5625 | -0.3176 | -0.4376 | -0.2280 | -0.3108 | 0.0790 | -0.2209 | -0.4319 | -0.6538 | -0.2365 | -0.0078 | -0.6095 | -0.3979 | -0.1077 | -0.3444 | 0.3100 | -0.1497 | -0.4309 | 0.4086 | -0.1445 -0.3914 -0.3194 0.6071 0.0372 -0.0802 0.2686 -0.3965 -0.5642 -0.2605 -0.4841 -0.0855 -0.2789 -0.4142 -0.4476 -0.4127 0.5671 -0.4245 -0.5354 -0.3065 -0.3847 -0.3545 -0.0375 -0.4340 -0.4341 -0.4278 -0.4330 0.3510 0.1019 -0.3448 -0.4660 0.3463 -0.6832 0.2529 -0.4059 -0.2907 -0.4993 -0.3463 -0.5240 -0.2589 -0.5008 -0.5602 -0.3827 -0.3587 |
| Root Phe | ##### | 0.8731 | -0.7346 | -0.4268 | -0.4978 | 0.8898 | 0.9101 | -0.7099 | ##### | 0.8358 | 0.6552 | 0.0599 | 0.6972 | 0.3721 | 0.3488 | 0.8290 | 0.7664 | -0.4668 | 1.0000 | 0.6099 | 0.8012 | 0.7090 | 0.9487 | 0.9867 | 0.8765 | 0.9752 | 0.7627 | 0.9612 | 0.0696 | 0.7510 | 0.7532 | 0.9646 | -0.8343 | 0.6842 | -0.4465 | 0.3600 | 0.7993 | 0.9794 | 0.0058 | 0.9182 | 0.5441 | 0.8407 | -0.8998 | 0.7627 | 0.8789 | 0.3431 | 0.7097 | -0.5458 | 0.6083 | 0.4116 | 0.9735 | 0.8044 | -0.2119 | 0.9591 | 0.6587 | 0.3373 | 0.5524 | -0.5096 | 0.3701 | 0.9564 | -0.7541 | 0.4008 0.7103 0.6624 -0.9179 0.0068 0.7044 -0.1475 0.9066 0.9412 0.2289 -0.1929 0.8898 0.5492 0.7680 0.8833 0.8127 -0.9564 0.8420 0.8622 0.6637 0.9362 0.9601 0.0035 0.6688 0.6934 0.6647 0.6821 -0.6505 -0.5309 0.7031 0.6732 -0.6217 0.9050 -0.5495 0.7753 0.4861 0.8303 0.8037 0.9605 0.6620 0.9341 0.9456 0.7171 0.7498 |
| Root Tyr | ##### | 0.8977 | -0.9592 | -0.9557 | -0.9494 | 0.5816 | 0.8151 | -0.9520 | ##### | 0.9447 | 0.9740 | 0.7953 | 0.9736 | 0.9343 | 0.9531 | 0.9443 | 0.9729 | -0.4051 | 0.6099 | 1.0000 | 0.9435 | 0.8360 | 0.8210 | 0.6453 | 0.9103 | 0.7445 | 0.9769 | 0.7783 | -0.7436 | 0.9165 | 0.9804 | 0.7780 | -0.9050 | 0.9834 | -0.9393 | 0.9410 | 0.9607 | 0.7208 | -0.7358 | 0.8077 | 0.9929 | 0.8990 | -0.8872 | 0.9488 | 0.9115 | 0.9438 | 0.9695 | -0.4481 | 0.8783 | 0.8984 | 0.6548 | 0.8513 | 0.6434 | 0.7661 | 0.9629 | 0.9094 | 0.9560 | -0.9476 | 0.9244 | 0.8074 | -0.9567 | 0.9366 0.9876 0.9877 -0.3606 0.7358 0.8190 0.6258 0.8597 0.8192 0.9068 0.5136 0.4558 0.9823 0.9559 0.9065 0.9547 -0.7974 0.9358 0.7318 0.9457 0.8263 0.7335 0.6954 0.9494 0.9627 0.9795 0.9647 -0.9633 -0.9156 0.9458 0.9672 -0.9693 0.8244 -0.9534 0.8277 0.9140 0.9208 0.9234 0.7954 0.9317 0.8126 0.8045 0.8878 0.9377 |
| Root Glu | ##### | 0.9884 | -0.9930 | -0.8768 | -0.9092 | 0.7674 | 0.9598 | -0.9882 | ##### | 0.9868 | 0.9052 | 0.6433 | 0.9295 | 0.8301 | 0.8269 | 0.9951 | 0.9886 | -0.3918 | 0.8012 | 0.9435 | 1.0000 | 0.9428 | 0.9444 | 0.8439 | 0.9696 | 0.8782 | 0.9822 | 0.9176 | -0.5059 | 0.8942 | 0.9827 | 0.9271 | -0.9756 | 0.9720 | -0.8889 | 0.8456 | 0.9943 | 0.8690 | -0.5862 | 0.9440 | 0.9022 | 0.9339 | -0.9580 | 0.9961 | 0.9834 | 0.8326 | 0.9489 | -0.6572 | 0.8051 | 0.8450 | 0.8076 | 0.9692 | 0.4009 | 0.9001 | 0.8980 | 0.7625 | 0.9370 | -0.9192 | 0.8514 | 0.9296 | -0.9960 | 0.8668 0.9835 0.9734 -0.5980 0.6002 0.9286 0.4142 0.9767 0.9094 0.7537 0.2254 0.7172 0.9380 0.9977 0.9851 0.9897 -0.9234 0.9943 0.8942 0.9752 0.9561 0.9105 0.5842 0.9700 0.9809 0.9749 0.9781 -0.9739 -0.9177 0.9851 0.9720 -0.9656 0.8924 -0.9386 0.9467 0.9000 0.9907 0.9973 0.9067 0.9708 0.9498 0.9344 0.9641 0.9932 |
| Root Asp | ##### | 0.9223 | -0.9464 | -0.8366 | -0.8869 | 0.7218 | 0.9332 | -0.9629 | ##### | 0.8783 | 0.7299 | 0.6597 | 0.7733 | 0.7695 | 0.7543 | 0.9071 | 0.8912 | -0.1904 | 0.7090 | 0.8360 | 0.9428 | 1.0000 | 0.8521 | 0.7885 | 0.8473 | 0.7522 | 0.8703 | 0.8261 | -0.4312 | 0.7081 | 0.8737 | 0.8533 | -0.8997 | 0.8913 | -0.8811 | 0.8184 | 0.9025 | 0.7562 | -0.6267 | 0.8977 | 0.7733 | 0.7932 | -0.8306 | 0.9529 | 0.8863 | 0.7896 | 0.8280 | -0.8501 | 0.6226 | 0.8345 | 0.6772 | 0.9866 | 0.3782 | 0.7939 | 0.7265 | 0.6480 | 0.9195 | -0.9115 | 0.8531 | 0.8269 | -0.9449 | 0.8588 0.8973 0.9037 -0.5358 0.6623 0.9904 0.4045 0.9215 0.7599 0.6961 0.0381 0.7734 0.8901 0.9411 0.8945 0.8898 -0.8190 0.9086 0.8933 0.9663 0.8901 0.8755 0.6733 0.9394 0.9349 0.9145 0.9320 -0.9462 -0.9435 0.9661 0.9165 -0.9344 0.7260 -0.9326 0.9708 0.9207 0.9249 0.9628 0.7737 0.9762 0.8893 0.8478 0.9768 0.9678 |
| Root Asn | ##### | 0.9809 | -0.9083 | -0.6901 | -0.7442 | 0.8934 | 0.9791 | -0.8853 | ##### | 0.9613 | 0.8338 | 0.3688 | 0.8587 | 0.6460 | 0.6224 | 0.9609 | 0.9283 | -0.5155 | 0.9487 | 0.8210 | 0.9444 | 0.8521 | 1.0000 | 0.9646 | 0.9799 | 0.9851 | 0.9235 | 0.9961 | -0.2410 | 0.8907 | 0.9173 | 0.9964 | -0.9628 | 0.8643 | -0.7023 | 0.6305 | 0.9453 | 0.9829 | -0.3060 | 0.9649 | 0.7707 | 0.9280 | -0.9865 | 0.9170 | 0.9788 | 0.6160 | 0.8664 | -0.6145 | 0.7352 | 0.6728 | 0.9539 | 0.9174 | 0.0994 | 0.9902 | 0.8221 | 0.5681 | 0.7834 | -0.7501 | 0.6326 | 0.9897 | -0.9200 | 0.6555 0.8905 0.8555 -0.8147 0.3068 0.8337 0.1163 0.9857 0.9827 0.5212 0.0584 0.8190 0.7773 0.9283 0.9826 0.9468 -0.9974 0.9647 0.9320 0.8556 0.9891 0.9752 0.3027 0.8659 0.8839 0.8660 0.8767 -0.8520 -0.7431 0.8836 0.8718 -0.8330 0.9654 -0.7754 0.9048 0.7267 0.9628 0.9405 0.9877 0.8487 0.9941 0.9969 0.8827 0.9123 |
| Root Gln | ##### | 0.9110 | -0.7912 | -0.5028 | -0.5813 | 0.9357 | 0.9516 | -0.7672 | ##### | 0.8562 | 0.6609 | 0.1534 | 0.6974 | 0.4497 | 0.4070 | 0.8598 | 0.8024 | -0.4791 | 0.9867 | 0.6453 | 0.8439 | 0.7885 | 0.9646 | 1.0000 | 0.8937 | 0.9714 | 0.7914 | 0.9781 | 0.0121 | 0.7604 | 0.7822 | 0.9817 | -0.8824 | 0.7148 | -0.5323 | 0.4316 | 0.8312 | 0.9813 | -0.1000 | 0.9311 | 0.5773 | 0.8246 | -0.9114 | 0.8075 | 0.8960 | 0.4079 | 0.7157 | -0.6649 | 0.5675 | 0.5108 | 0.9679 | 0.8614 | -0.1487 | 0.9730 | 0.6500 | 0.3441 | 0.6315 | -0.5924 | 0.4467 | 0.9541 | -0.8071 | 0.4700 0.7474 0.7036 -0.9222 0.1044 0.7715 -0.1177 0.9355 0.9312 0.2947 -0.1772 0.9072 0.6056 0.8175 0.9045 0.8341 -0.9692 0.8689 0.9305 0.7337 0.9522 0.9795 0.1221 0.7423 0.7575 0.7236 0.7471 -0.7179 -0.6023 0.7692 0.7378 -0.6893 0.9034 -0.6237 0.8571 0.5813 0.8773 0.8516 0.9512 0.7330 0.9665 0.9690 0.7994 0.8098 |
| Root Lys | ##### | 0.9850 | -0.9486 | -0.7940 | -0.8278 | 0.8321 | 0.9494 | -0.9232 | ##### | 0.9922 | 0.9257 | 0.5052 | 0.9355 | 0.7637 | 0.7496 | 0.9883 | 0.9772 | -0.5529 | 0.8765 | 0.9103 | 0.9696 | 0.8473 | 0.9799 | 0.8937 | 1.0000 | 0.9536 | 0.9774 | 0.9656 | -0.4181 | 0.9561 | 0.9730 | 0.9599 | -0.9804 | 0.9266 | -0.7907 | 0.7413 | 0.9830 | 0.9430 | -0.4396 | 0.9347 | 0.8785 | 0.9551 | -0.9984 | 0.9473 | 0.9910 | 0.7348 | 0.9300 | -0.5380 | 0.8165 | 0.7634 | 0.9076 | 0.9047 | 0.2731 | 0.9620 | 0.9085 | 0.6965 | 0.8549 | -0.8265 | 0.7303 | 0.9665 | -0.9568 | 0.7510 0.9505 0.9211 -0.7080 0.4304 0.8239 0.2657 0.9717 0.9779 0.6614 0.2485 0.7014 0.8606 0.9633 0.9916 0.9819 -0.9747 0.9867 0.8927 0.8986 0.9647 0.9204 0.4141 0.9153 0.9341 0.9289 0.9298 -0.9062 -0.7980 0.9209 0.9300 -0.8953 0.9751 -0.8430 0.8948 0.7949 0.9810 0.9554 0.9698 0.8853 0.9679 0.9737 0.8991 0.9397 |
| Root Arg | ##### | 0.9350 | -0.8299 | -0.5803 | -0.6368 | 0.9058 | 0.9362 | -0.7957 | ##### | 0.9177 | 0.7926 | 0.2286 | 0.8117 | 0.5446 | 0.5156 | 0.9094 | 0.8698 | -0.5833 | 0.9752 | 0.7445 | 0.8782 | 0.7522 | 0.9851 | 0.9714 | 0.9536 | 1.0000 | 0.8690 | 0.9911 | -0.1317 | 0.8777 | 0.8597 | 0.9835 | -0.9183 | 0.7854 | -0.5850 | 0.5091 | 0.8892 | 0.9982 | -0.1622 | 0.9268 | 0.6980 | 0.9007 | -0.9685 | 0.8394 | 0.9404 | 0.4981 | 0.8062 | -0.5206 | 0.7005 | 0.5645 | 0.9884 | 0.8383 | -0.0286 | 0.9938 | 0.7782 | 0.4765 | 0.6773 | -0.6372 | 0.5031 | 0.9789 | -0.8456 | 0.5298 0.8178 0.7707 -0.8729 0.1521 0.7315 -0.0153 0.9438 0.9883 0.4054 0.0258 0.8016 0.6744 0.8574 0.9437 0.8948 -0.9931 0.9143 0.8951 0.7576 0.9593 0.9530 0.1482 0.7788 0.8019 0.7831 0.7933 -0.7573 -0.6205 0.7937 0.7914 -0.7356 0.9761 -0.6629 0.8301 0.6076 0.9096 0.8691 0.9926 0.7468 0.9682 0.9842 0.7950 0.8307 |
| Root Thr | ##### | 0.9664 | -0.9796 | -0.8970 | -0.9124 | 0.7263 | 0.9083 | -0.9641 | ##### | 0.9918 | 0.9679 | 0.6678 | 0.9743 | 0.8688 | 0.8708 | 0.9906 | 0.9987 | -0.4809 | 0.7627 | 0.9769 | 0.9822 | 0.8703 | 0.9235 | 0.7914 | 0.9774 | 0.8690 | 1.0000 | 0.8942 | -0.5908 | 0.9535 | 0.9997 | 0.8923 | -0.9667 | 0.9789 | -0.8888 | 0.8630 | 0.9960 | 0.8521 | -0.6045 | 0.8964 | 0.9553 | 0.9480 | -0.9646 | 0.9742 | 0.9751 | 0.8602 | 0.9716 | -0.5190 | 0.8631 | 0.8531 | 0.7990 | 0.9060 | 0.4675 | 0.8852 | 0.9540 | 0.8195 | 0.9303 | -0.9118 | 0.8506 | 0.9094 | -0.9824 | 0.8673 0.9929 0.9787 -0.5489 0.6014 0.8501 0.4566 0.9411 0.9184 0.8020 0.3784 0.6019 0.9454 0.9851 0.9732 0.9921 -0.9080 0.9857 0.8371 0.9488 0.9201 0.8516 0.5735 0.9577 0.9736 0.9786 0.9723 -0.9605 -0.8833 0.9599 0.9730 -0.9576 0.9181 -0.9238 0.8884 0.8798 0.9758 0.9659 0.9031 0.9357 0.9146 0.9119 0.9206 0.9653 |
| Root L-citrulline | ##### | 0.9663 | -0.8796 | -0.6464 | -0.7076 | 0.9269 | 0.9716 | -0.8502 | ##### | 0.9367 | 0.7978 | 0.3131 | 0.8176 | 0.6074 | 0.5695 | 0.9370 | 0.9007 | -0.5593 | 0.9612 | 0.7783 | 0.9176 | 0.8261 | 0.9961 | 0.9781 | 0.9656 | 0.9911 | 0.8942 | 1.0000 | -0.1832 | 0.8770 | 0.8861 | 0.9959 | -0.9533 | 0.8199 | -0.6603 | 0.5767 | 0.9181 | 0.9929 | -0.2550 | 0.9457 | 0.7278 | 0.8954 | -0.9753 | 0.8837 | 0.9575 | 0.5603 | 0.8210 | -0.6207 | 0.6807 | 0.6434 | 0.9730 | 0.8934 | 0.0335 | 0.9978 | 0.7776 | 0.4983 | 0.7463 | -0.7103 | 0.5775 | 0.9793 | -0.8923 | 0.5991 0.8536 0.8126 -0.8605 0.2455 0.8004 0.0330 0.9707 0.9770 0.4657 0.0274 0.8244 0.7294 0.9011 0.9634 0.9170 -0.9988 0.9400 0.9434 0.8213 0.9761 0.9718 0.2536 0.8397 0.8560 0.8337 0.8482 -0.8175 -0.6932 0.8529 0.8445 -0.7962 0.9660 -0.7321 0.8963 0.6903 0.9471 0.9140 0.9831 0.8130 0.9923 0.9986 0.8638 0.8845 |
| Root 5-hydroxy-tryptamine | ##### | ###### | 0.5851 | 0.8435 | 0.7774 | -0.0208 | -0.2547 | 0.5856 | ##### | -0.4854 | -0.6851 | -0.9410 | -0.6349 | -0.8760 | -0.9028 | -0.4896 | -0.5826 | 0.2114 | 0.0696 | -0.7436 | -0.5059 | -0.4312 | -0.2410 | 0.0121 | -0.4181 | -0.1317 | -0.5908 | -0.1832 | 1.0000 | -0.5532 | -0.6010 | -0.1703 | 0.4516 | -0.6454 | 0.8010 | -0.8683 | -0.5350 | -0.0999 | 0.9253 | -0.2215 | -0.7978 | -0.4102 | 0.3704 | -0.5370 | -0.4034 | -0.8871 | -0.6064 | 0.0861 | -0.5707 | -0.7977 | -0.0319 | -0.3714 | -0.9769 | -0.1760 | -0.6530 | -0.8333 | -0.7339 | 0.7569 | -0.8306 | -0.2066 | 0.5655 | -0.8179 -0.6390 -0.6723 -0.2856 -0.8947 -0.4005 -0.8641 -0.3111 -0.2504 -0.9455 -0.8578 0.2077 -0.7602 -0.5529 -0.3948 -0.5113 0.2115 -0.4641 -0.2114 -0.6191 -0.2424 -0.1043 -0.8579 -0.6349 -0.6295 -0.6741 -0.6415 0.6576 0.6732 -0.5893 -0.6565 0.6895 -0.3033 0.7219 -0.3895 -0.7344 -0.4658 -0.4735 -0.1994 -0.5976 -0.2422 -0.2291 -0.5113 -0.5401 |
| Root L-homocitrulline | ##### | 0.9033 | -0.8904 | -0.8104 | -0.8205 | 0.7523 | 0.8237 | -0.8455 | ##### | 0.9416 | 0.9657 | 0.5537 | 0.9379 | 0.8177 | 0.7949 | 0.9285 | 0.9452 | -0.7005 | 0.7510 | 0.9165 | 0.8942 | 0.7081 | 0.8907 | 0.7604 | 0.9561 | 0.8777 | 0.9535 | 0.8770 | -0.5532 | 1.0000 | 0.9473 | 0.8498 | -0.9309 | 0.8818 | -0.7790 | 0.7490 | 0.9336 | 0.8597 | -0.4915 | 0.7930 | 0.9195 | 0.8956 | -0.9502 | 0.8647 | 0.9164 | 0.7554 | 0.8960 | -0.3429 | 0.8168 | 0.7818 | 0.8401 | 0.7645 | 0.3856 | 0.8879 | 0.9248 | 0.7272 | 0.8256 | -0.7988 | 0.7097 | 0.8646 | -0.8957 | 0.7259 0.9161 0.8810 -0.5999 0.4432 0.6648 0.3073 0.8610 0.9233 0.7236 0.4975 0.4696 0.8361 0.9015 0.9143 0.9258 -0.8955 0.9170 0.7861 0.8271 0.8462 0.7708 0.4260 0.8732 0.8927 0.9018 0.8915 -0.8552 -0.7132 0.8491 0.9039 -0.8535 0.9582 -0.7942 0.7866 0.7563 0.9175 0.8606 0.8945 0.7994 0.8661 0.8892 0.8123 0.8592 |
| Roor beta-alanine | ##### | 0.9634 | -0.9806 | -0.9025 | -0.9163 | 0.7118 | 0.9047 | -0.9676 | ##### | 0.9905 | 0.9681 | 0.6790 | 0.9766 | 0.8724 | 0.8783 | 0.9895 | 0.9982 | -0.4609 | 0.7532 | 0.9804 | 0.9827 | 0.8737 | 0.9173 | 0.7822 | 0.9730 | 0.8597 | 0.9997 | 0.8861 | -0.6010 | 0.9473 | 1.0000 | 0.8858 | -0.9619 | 0.9837 | -0.8946 | 0.8719 | 0.9956 | 0.8421 | -0.6153 | 0.8958 | 0.9584 | 0.9487 | -0.9593 | 0.9768 | 0.9733 | 0.8692 | 0.9759 | -0.5186 | 0.8693 | 0.8554 | 0.7863 | 0.9079 | 0.4818 | 0.8757 | 0.9569 | 0.8314 | 0.9344 | -0.9170 | 0.8606 | 0.9050 | -0.9831 | 0.8774 0.9954 0.9832 -0.5319 0.6158 0.8559 0.4765 0.9388 0.9115 0.8108 0.3797 0.5989 0.9518 0.9855 0.9709 0.9924 -0.9001 0.9850 0.8290 0.9524 0.9171 0.8471 0.5851 0.9584 0.9744 0.9803 0.9733 -0.9636 -0.8925 0.9623 0.9734 -0.9613 0.9089 -0.9300 0.8860 0.8848 0.9730 0.9669 0.8964 0.9401 0.9085 0.9044 0.9206 0.9667 |
| Root L-pipecolic-acid | ##### | 0.9701 | -0.8860 | -0.6457 | -0.7078 | 0.9059 | 0.9833 | -0.8658 | ##### | 0.9387 | 0.7855 | 0.3178 | 0.8177 | 0.5949 | 0.5684 | 0.9407 | 0.8997 | -0.4823 | 0.9646 | 0.7780 | 0.9271 | 0.8533 | 0.9964 | 0.9817 | 0.9599 | 0.9835 | 0.8923 | 0.9959 | -0.1703 | 0.8498 | 0.8858 | 1.0000 | -0.9458 | 0.8332 | -0.6656 | 0.5855 | 0.9212 | 0.9850 | -0.2574 | 0.9694 | 0.7195 | 0.9063 | -0.9691 | 0.8993 | 0.9638 | 0.5673 | 0.8328 | -0.6500 | 0.6933 | 0.6340 | 0.9569 | 0.9190 | 0.0345 | 0.9881 | 0.7770 | 0.5135 | 0.7518 | -0.7175 | 0.5944 | 0.9874 | -0.8987 | 0.6176 0.8592 0.8237 -0.8439 0.2642 0.8379 0.0632 0.9827 0.9703 0.4637 -0.0246 0.8624 0.7405 0.9074 0.9691 0.9237 -0.9937 0.9469 0.9399 0.8351 0.9895 0.9885 0.2653 0.8408 0.8577 0.8347 0.8496 -0.8261 -0.7219 0.8643 0.8421 -0.8040 0.9460 -0.7466 0.9043 0.6988 0.9468 0.9285 0.9812 0.8316 0.9944 0.9944 0.8722 0.8962 |
| Root 3-N-methyl-L-histidine | ##### | ###### | 0.9712 | 0.8402 | 0.8842 | -0.8705 | -0.9613 | 0.9445 | ##### | -0.9725 | -0.8881 | -0.5774 | -0.8882 | -0.8157 | -0.7731 | -0.9811 | -0.9756 | 0.5697 | -0.8343 | -0.9050 | -0.9756 | -0.8997 | -0.9628 | -0.8824 | -0.9804 | -0.9183 | -0.9667 | -0.9533 | 0.4516 | -0.9309 | -0.9619 | -0.9458 | 1.0000 | -0.9132 | 0.8487 | -0.7797 | -0.9762 | -0.9163 | 0.5306 | -0.9083 | -0.8722 | -0.8901 | 0.9736 | -0.9537 | -0.9682 | -0.7649 | -0.8872 | 0.6591 | -0.7193 | -0.8438 | -0.8772 | -0.9294 | -0.3145 | -0.9480 | -0.8506 | -0.6540 | -0.9045 | 0.8803 | -0.7740 | -0.9255 | 0.9752 | -0.7847 -0.9474 -0.9210 0.7057 -0.5069 -0.8593 -0.2594 -0.9653 -0.9303 -0.6962 -0.2516 -0.6961 -0.8790 -0.9775 -0.9744 -0.9614 0.9564 -0.9744 -0.9461 -0.9361 -0.9440 -0.9083 -0.5210 -0.9603 -0.9668 -0.9541 -0.9634 0.9408 0.8340 -0.9545 -0.9639 0.9297 -0.9408 0.8843 -0.9560 -0.8655 -0.9963 -0.9676 -0.9232 -0.9236 -0.9713 -0.9679 -0.9571 -0.9664 |
| Root momoserine | ##### | 0.9299 | -0.9726 | -0.9195 | -0.9240 | 0.6044 | 0.8724 | -0.9781 | ##### | 0.9654 | 0.9462 | 0.7367 | 0.9715 | 0.8739 | 0.9069 | 0.9668 | 0.9771 | -0.3008 | 0.6842 | 0.9834 | 0.9720 | 0.8913 | 0.8643 | 0.7148 | 0.9266 | 0.7854 | 0.9789 | 0.8199 | -0.6454 | 0.8818 | 0.9837 | 0.8332 | -0.9132 | 1.0000 | -0.9166 | 0.9133 | 0.9770 | 0.7638 | -0.6706 | 0.8865 | 0.9560 | 0.9393 | -0.9076 | 0.9820 | 0.9476 | 0.9109 | 0.9882 | -0.5215 | 0.8944 | 0.8511 | 0.6898 | 0.9146 | 0.5592 | 0.7994 | 0.9559 | 0.8928 | 0.9454 | -0.9353 | 0.9126 | 0.8658 | -0.9729 | 0.9302 0.9943 0.9975 -0.4146 0.6990 0.8920 0.5992 0.9131 0.8506 0.8485 0.3578 0.5865 0.9782 0.9736 0.9426 0.9791 -0.8349 0.9659 0.7658 0.9636 0.8888 0.8129 0.6501 0.9467 0.9632 0.9736 0.9630 -0.9695 -0.9439 0.9647 0.9580 -0.9701 0.8289 -0.9574 0.8605 0.9039 0.9389 0.9619 0.8391 0.9584 0.8575 0.8415 0.9087 0.9635 |
| Root L-cystathionine | ##### | ###### | 0.9346 | 0.9941 | 0.9966 | -0.5287 | -0.7579 | 0.9381 | ##### | -0.8383 | -0.8447 | -0.9182 | -0.8383 | -0.9747 | -0.9646 | -0.8600 | -0.8993 | 0.3024 | -0.4465 | -0.9393 | -0.8889 | -0.8811 | -0.7023 | -0.5323 | -0.7907 | -0.5850 | -0.8888 | -0.6603 | 0.8010 | -0.7790 | -0.8946 | -0.6656 | 0.8487 | -0.9166 | 1.0000 | -0.9848 | -0.8797 | -0.5733 | 0.8917 | -0.7031 | -0.9294 | -0.7307 | 0.7558 | -0.9059 | -0.7997 | -0.9749 | -0.8523 | 0.6141 | -0.6874 | -0.9811 | -0.4923 | -0.8412 | -0.7422 | -0.6394 | -0.8144 | -0.8368 | -0.9925 | 0.9971 | -0.9838 | -0.6590 | 0.9235 | -0.9791 -0.9253 -0.9416 0.2428 -0.8856 -0.8489 -0.6750 -0.7754 -0.6464 -0.9474 -0.4915 -0.3971 -0.9771 -0.9144 -0.8022 -0.8549 0.6697 -0.8453 -0.7265 -0.9600 -0.7175 -0.6378 -0.8842 -0.9609 -0.9533 -0.9611 -0.9577 0.9692 0.9579 -0.9470 -0.9576 0.9773 -0.6618 0.9870 -0.8581 -0.9930 -0.8664 -0.8832 -0.6269 -0.9523 -0.7290 -0.6985 -0.9211 -0.9232 |
| Root N6-acetyl-L-lysine | ##### | 0.7632 | -0.8929 | -0.9900 | -0.9724 | 0.3965 | 0.6774 | -0.9044 | ##### | 0.8031 | 0.8523 | 0.9482 | 0.8492 | 0.9700 | 0.9894 | 0.8182 | 0.8667 | -0.2219 | 0.3600 | 0.9410 | 0.8456 | 0.8184 | 0.6305 | 0.4316 | 0.7413 | 0.5091 | 0.8630 | 0.5767 | -0.8683 | 0.7490 | 0.8719 | 0.5855 | -0.7797 | 0.9133 | -0.9848 | 1.0000 | 0.8447 | 0.4882 | -0.9139 | 0.6502 | 0.9400 | 0.7230 | -0.7029 | 0.8721 | 0.7566 | 0.9982 | 0.8618 | -0.4937 | 0.7419 | 0.9476 | 0.4005 | 0.7807 | 0.8244 | 0.5538 | 0.8380 | 0.9089 | 0.9655 | -0.9748 | 0.9948 | 0.6031 | -0.8807 | 0.9930 0.9080 0.9330 -0.1098 0.9212 0.8004 0.7861 0.7128 0.5935 0.9812 0.5619 0.3020 0.9764 0.8718 0.7537 0.8272 -0.5927 0.8049 0.6124 0.9226 0.6559 0.5570 0.8932 0.9133 0.9112 0.9308 0.9170 -0.9362 -0.9480 0.9041 0.9173 -0.9501 0.6023 -0.9691 0.7694 0.9607 0.8054 0.8348 0.5689 0.9149 0.6460 0.6163 0.8520 0.8755 |
| Root trans-4-hydroxy-L-proline | ##### | 0.9835 | -0.9865 | -0.8790 | -0.9034 | 0.7571 | 0.9404 | -0.9750 | ##### | 0.9966 | 0.9429 | 0.6386 | 0.9587 | 0.8414 | 0.8415 | 0.9985 | 0.9976 | -0.4483 | 0.7993 | 0.9607 | 0.9943 | 0.9025 | 0.9453 | 0.8312 | 0.9830 | 0.8892 | 0.9960 | 0.9181 | -0.5350 | 0.9336 | 0.9956 | 0.9212 | -0.9762 | 0.9770 | -0.8797 | 0.8447 | 1.0000 | 0.8758 | -0.5761 | 0.9293 | 0.9289 | 0.9524 | -0.9722 | 0.9866 | 0.9877 | 0.8375 | 0.9656 | -0.5787 | 0.8437 | 0.8392 | 0.8206 | 0.9384 | 0.4168 | 0.9058 | 0.9332 | 0.7886 | 0.9274 | -0.9078 | 0.8405 | 0.9327 | -0.9903 | 0.8578 0.9901 0.9760 -0.5908 0.5815 0.8860 0.4209 0.9664 0.9289 0.7675 0.2965 0.6690 0.9374 0.9932 0.9873 0.9971 -0.9282 0.9963 0.8698 0.9585 0.9479 0.8914 0.5578 0.9612 0.9761 0.9756 0.9737 -0.9641 -0.8930 0.9706 0.9712 -0.9579 0.9196 -0.9247 0.9165 0.8804 0.9876 0.9839 0.9200 0.9493 0.9411 0.9341 0.9395 0.9791 |
| Root L-ornithine | ##### | 0.9311 | -0.8213 | -0.5638 | -0.6266 | 0.9275 | 0.9400 | -0.7859 | ##### | 0.9034 | 0.7639 | 0.2101 | 0.7825 | 0.5283 | 0.4898 | 0.8981 | 0.8557 | -0.5890 | 0.9794 | 0.7208 | 0.8690 | 0.7562 | 0.9829 | 0.9813 | 0.9430 | 0.9982 | 0.8521 | 0.9929 | -0.0999 | 0.8597 | 0.8421 | 0.9850 | -0.9163 | 0.7638 | -0.5733 | 0.4882 | 0.8758 | 1.0000 | -0.1486 | 0.9214 | 0.6715 | 0.8771 | -0.9587 | 0.8283 | 0.9290 | 0.4742 | 0.7789 | -0.5545 | 0.6589 | 0.5592 | 0.9923 | 0.8387 | -0.0599 | 0.9957 | 0.7450 | 0.4351 | 0.6674 | -0.6269 | 0.4849 | 0.9715 | -0.8369 | 0.5098 0.7999 0.7514 -0.8965 0.1352 0.7317 -0.0554 0.9405 0.9779 0.3793 -0.0079 0.8180 0.6550 0.8482 0.9341 0.8787 -0.9922 0.9027 0.9118 0.7502 0.9552 0.9570 0.1411 0.7735 0.7938 0.7708 0.7849 -0.7477 -0.6080 0.7871 0.7825 -0.7243 0.9673 -0.6508 0.8400 0.6023 0.9054 0.8627 0.9846 0.7402 0.9711 0.9857 0.7983 0.8249 |
| Root L-tyrosine-methyl-ester | ##### | ###### | 0.6724 | 0.8942 | 0.8634 | -0.1556 | -0.3887 | 0.6865 | ##### | -0.5108 | -0.6065 | -0.9936 | -0.5750 | -0.9026 | -0.8965 | -0.5396 | -0.6155 | 0.1205 | 0.0058 | -0.7358 | -0.5862 | -0.6267 | -0.3060 | -0.1000 | -0.4396 | -0.1622 | -0.6045 | -0.2550 | 0.9253 | -0.4915 | -0.6153 | -0.2574 | 0.5306 | -0.6706 | 0.8917 | -0.9139 | -0.5761 | -0.1486 | 1.0000 | -0.3162 | -0.7585 | -0.3812 | 0.3890 | -0.6228 | -0.4458 | -0.9102 | -0.5830 | 0.4223 | -0.4425 | -0.8940 | -0.0631 | -0.5319 | -0.9290 | -0.2337 | -0.5661 | -0.7483 | -0.8301 | 0.8565 | -0.9071 | -0.2493 | 0.6499 | -0.8850 -0.6703 -0.7112 -0.1806 -0.9819 -0.5891 -0.8108 -0.4068 -0.2447 -0.9383 -0.6469 0.0068 -0.8090 -0.6325 -0.4473 -0.5395 0.2670 -0.5158 -0.3865 -0.7362 -0.3261 -0.2270 -0.9879 -0.7381 -0.7157 -0.7374 -0.7262 0.7533 0.7962 -0.7023 -0.7312 0.7765 -0.2838 0.8225 -0.5735 -0.8658 -0.5502 -0.5787 -0.2129 -0.7277 -0.3454 -0.3058 -0.6711 -0.6520 |
| Root N-acetylaspartate | ##### | 0.9586 | -0.9006 | -0.6775 | -0.7277 | 0.7855 | 0.9740 | -0.9058 | ##### | 0.9417 | 0.7880 | 0.3862 | 0.8477 | 0.6013 | 0.6173 | 0.9445 | 0.9018 | -0.2731 | 0.9182 | 0.8077 | 0.9440 | 0.8977 | 0.9649 | 0.9311 | 0.9347 | 0.9268 | 0.8964 | 0.9457 | -0.2215 | 0.7930 | 0.8958 | 0.9694 | -0.9083 | 0.8865 | -0.7031 | 0.6502 | 0.9293 | 0.9214 | -0.3162 | 1.0000 | 0.7388 | 0.9402 | -0.9385 | 0.9368 | 0.9685 | 0.6331 | 0.8871 | -0.6549 | 0.7714 | 0.6289 | 0.8681 | 0.9574 | 0.1246 | 0.9238 | 0.8131 | 0.6206 | 0.7801 | -0.7535 | 0.6735 | 0.9802 | -0.9120 | 0.7011 0.8873 0.8706 -0.7188 0.3657 0.9115 0.2290 0.9859 0.9330 0.5181 -0.0662 0.8846 0.7960 0.9196 0.9692 0.9424 -0.9457 0.9573 0.8773 0.8710 0.9929 0.9833 0.3355 0.8432 0.8641 0.8475 0.8566 -0.8543 -0.8090 0.8902 0.8413 -0.8352 0.8776 -0.7995 0.8893 0.7318 0.9304 0.9525 0.9498 0.8773 0.9613 0.9468 0.8744 0.9176 |
| Root (5-L-glutamyl)-L-amino-acid | ##### | 0.8522 | -0.9258 | -0.9565 | -0.9400 | 0.5351 | 0.7525 | -0.9120 | ##### | 0.9115 | 0.9779 | 0.8154 | 0.9629 | 0.9499 | 0.9661 | 0.9080 | 0.9483 | -0.4502 | 0.5441 | 0.9929 | 0.9022 | 0.7733 | 0.7707 | 0.5773 | 0.8785 | 0.6980 | 0.9553 | 0.7278 | -0.7978 | 0.9195 | 0.9584 | 0.7195 | -0.8722 | 0.9560 | -0.9294 | 0.9400 | 0.9289 | 0.6715 | -0.7585 | 0.7388 | 1.0000 | 0.8620 | -0.8531 | 0.9058 | 0.8684 | 0.9479 | 0.9453 | -0.3673 | 0.8682 | 0.9001 | 0.6110 | 0.7859 | 0.6923 | 0.7208 | 0.9580 | 0.9169 | 0.9373 | -0.9304 | 0.9129 | 0.7521 | -0.9218 | 0.9227 0.9634 0.9626 -0.3021 0.7439 0.7489 0.6471 0.8027 0.7815 0.9285 0.6110 0.3532 0.9654 0.9204 0.8624 0.9201 -0.7504 0.8955 0.6769 0.9091 0.7656 0.6606 0.7032 0.9234 0.9364 0.9591 0.9397 -0.9344 -0.8769 0.9081 0.9469 -0.9445 0.8014 -0.9276 0.7747 0.8933 0.8831 0.8749 0.7496 0.8898 0.7580 0.7559 0.8442 0.8958 |
| Root glycyl-L-proline | ##### | 0.9281 | -0.8966 | -0.7411 | -0.7543 | 0.6662 | 0.8880 | -0.8926 | ##### | 0.9694 | 0.9238 | 0.4689 | 0.9627 | 0.6877 | 0.7335 | 0.9542 | 0.9377 | -0.3552 | 0.8407 | 0.8990 | 0.9339 | 0.7932 | 0.9280 | 0.8246 | 0.9551 | 0.9007 | 0.9480 | 0.8954 | -0.4102 | 0.8956 | 0.9487 | 0.9063 | -0.8901 | 0.9393 | -0.7307 | 0.7230 | 0.9524 | 0.8771 | -0.3812 | 0.9402 | 0.8620 | 1.0000 | -0.9545 | 0.9277 | 0.9720 | 0.7256 | 0.9712 | -0.4014 | 0.9325 | 0.6515 | 0.8277 | 0.8696 | 0.2937 | 0.8831 | 0.9517 | 0.7890 | 0.7925 | -0.7674 | 0.7181 | 0.9561 | -0.9073 | 0.7506 0.9334 0.9167 -0.5817 0.4180 0.8090 0.3882 0.9364 0.9509 0.6431 0.2073 0.6891 0.8484 0.9167 0.9637 0.9722 -0.9117 0.9625 0.7542 0.8508 0.9432 0.8854 0.3499 0.8346 0.8673 0.8746 0.8628 -0.8553 -0.7951 0.8683 0.8569 -0.8475 0.9125 -0.8076 0.7854 0.7190 0.9107 0.9194 0.9463 0.8447 0.8959 0.8992 0.8060 0.8890 |
| Root trimethylamine-N-oxide | ##### | ###### | 0.9313 | 0.7588 | 0.7959 | -0.8464 | -0.9503 | 0.9040 | ##### | -0.9856 | -0.9108 | -0.4562 | -0.9224 | -0.7276 | -0.7124 | -0.9800 | -0.9640 | 0.5625 | -0.8998 | -0.8872 | -0.9580 | -0.8306 | -0.9865 | -0.9114 | -0.9984 | -0.9685 | -0.9646 | -0.9753 | 0.3704 | -0.9502 | -0.9593 | -0.9691 | 0.9736 | -0.9076 | 0.7558 | -0.7029 | -0.9722 | -0.9587 | 0.3890 | -0.9385 | -0.8531 | -0.9545 | 1.0000 | -0.9326 | -0.9885 | -0.6963 | -0.9161 | 0.5281 | -0.8070 | -0.7286 | -0.9281 | -0.8951 | -0.2211 | -0.9731 | -0.8950 | -0.6652 | -0.8255 | 0.7947 | -0.6920 | -0.9755 | 0.9411 | -0.7145 -0.9327 -0.8998 0.7396 -0.3799 -0.8084 -0.2192 -0.9715 -0.9877 -0.6194 -0.2143 -0.7202 -0.8318 -0.9488 -0.9891 -0.9730 0.9837 -0.9797 -0.8937 -0.8760 -0.9691 -0.9302 -0.3638 -0.8936 -0.9140 -0.9071 -0.9089 0.8828 0.7684 -0.9012 -0.9089 0.8701 -0.9829 0.8134 -0.8836 -0.7621 -0.9725 -0.9439 -0.9813 -0.8629 -0.9721 -0.9805 -0.8816 -0.9235 |
| Root N8-acetylspermidine | ##### | 0.9725 | -0.9918 | -0.8917 | -0.9190 | 0.7145 | 0.9431 | -0.9958 | ##### | 0.9752 | 0.8973 | 0.6804 | 0.9285 | 0.8385 | 0.8475 | 0.9850 | 0.9806 | -0.3176 | 0.7627 | 0.9488 | 0.9961 | 0.9529 | 0.9170 | 0.8075 | 0.9473 | 0.8394 | 0.9742 | 0.8837 | -0.5370 | 0.8647 | 0.9768 | 0.8993 | -0.9537 | 0.9820 | -0.9059 | 0.8721 | 0.9866 | 0.8283 | -0.6228 | 0.9368 | 0.9058 | 0.9277 | -0.9326 | 1.0000 | 0.9705 | 0.8594 | 0.9558 | -0.6599 | 0.8167 | 0.8506 | 0.7577 | 0.9726 | 0.4479 | 0.8614 | 0.8990 | 0.7961 | 0.9475 | -0.9337 | 0.8826 | 0.9078 | -0.9935 | 0.8979 0.9855 0.9832 -0.5365 0.6491 0.9467 0.4797 0.9632 0.8778 0.7798 0.2242 0.7052 0.9551 0.9940 0.9709 0.9839 -0.8901 0.9854 0.8641 0.9839 0.9402 0.8908 0.6251 0.9684 0.9791 0.9756 0.9769 -0.9806 -0.9463 0.9892 0.9686 -0.9741 0.8528 -0.9565 0.9364 0.9152 0.9756 0.9959 0.8763 0.9827 0.9240 0.9027 0.9615 0.9939 |
| Root glutathione-oxidized | ##### | 0.9895 | -0.9579 | -0.7947 | -0.8290 | 0.7971 | 0.9647 | -0.9464 | ##### | 0.9953 | 0.9093 | 0.5150 | 0.9375 | 0.7470 | 0.7508 | 0.9936 | 0.9760 | -0.4376 | 0.8789 | 0.9115 | 0.9834 | 0.8863 | 0.9788 | 0.8960 | 0.9910 | 0.9404 | 0.9751 | 0.9575 | -0.4034 | 0.9164 | 0.9733 | 0.9638 | -0.9682 | 0.9476 | -0.7997 | 0.7566 | 0.9877 | 0.9290 | -0.4458 | 0.9685 | 0.8684 | 0.9720 | -0.9885 | 0.9705 | 1.0000 | 0.7477 | 0.9490 | -0.5775 | 0.8380 | 0.7502 | 0.8812 | 0.9413 | 0.2792 | 0.9456 | 0.9100 | 0.7230 | 0.8637 | -0.8381 | 0.7572 | 0.9774 | -0.9662 | 0.7805 0.9597 0.9387 -0.6787 0.4610 0.8785 0.3156 0.9882 0.9670 0.6617 0.1760 0.7541 0.8785 0.9724 0.9994 0.9921 -0.9653 0.9967 0.8845 0.9191 0.9831 0.9428 0.4335 0.9168 0.9368 0.9309 0.9321 -0.9194 -0.8415 0.9377 0.9267 -0.9076 0.9444 -0.8655 0.9046 0.8081 0.9797 0.9762 0.9657 0.9128 0.9690 0.9656 0.9105 0.9565 |
| Root methionine-sulfoxide | ##### | 0.7476 | -0.8797 | -0.9861 | -0.9621 | 0.3685 | 0.6538 | -0.8901 | ##### | 0.7974 | 0.8641 | 0.9477 | 0.8584 | 0.9694 | 0.9950 | 0.8091 | 0.8609 | -0.2280 | 0.3431 | 0.9438 | 0.8326 | 0.7896 | 0.6160 | 0.4079 | 0.7348 | 0.4981 | 0.8602 | 0.5603 | -0.8871 | 0.7554 | 0.8692 | 0.5673 | -0.7649 | 0.9109 | -0.9749 | 0.9982 | 0.8375 | 0.4742 | -0.9102 | 0.6331 | 0.9479 | 0.7256 | -0.6963 | 0.8594 | 0.7477 | 1.0000 | 0.8661 | -0.4421 | 0.7625 | 0.9358 | 0.3880 | 0.7558 | 0.8410 | 0.5391 | 0.8517 | 0.9272 | 0.9534 | -0.9629 | 0.9886 | 0.5922 | -0.8678 | 0.9881 0.9039 0.9288 -0.0840 0.9166 0.7732 0.8044 0.6954 0.5893 0.9881 0.5979 0.2668 0.9722 0.8593 0.7432 0.8219 -0.5789 0.7955 0.5813 0.9063 0.6397 0.5339 0.8813 0.8982 0.8985 0.9220 0.9047 -0.9231 -0.9333 0.8878 0.9063 -0.9387 0.5997 -0.9570 0.7399 0.9440 0.7906 0.8182 0.5616 0.8969 0.6260 0.5996 0.8276 0.8586 |
| Root Asp-Phe | ##### | 0.9118 | -0.9370 | -0.8658 | -0.8630 | 0.5770 | 0.8480 | -0.9400 | ##### | 0.9648 | 0.9623 | 0.6617 | 0.9908 | 0.8202 | 0.8700 | 0.9561 | 0.9627 | -0.3108 | 0.7097 | 0.9695 | 0.9489 | 0.8280 | 0.8664 | 0.7157 | 0.9300 | 0.8062 | 0.9716 | 0.8210 | -0.6064 | 0.8960 | 0.9759 | 0.8328 | -0.8872 | 0.9882 | -0.8523 | 0.8618 | 0.9656 | 0.7789 | -0.5830 | 0.8871 | 0.9453 | 0.9712 | -0.9161 | 0.9558 | 0.9490 | 0.8661 | 1.0000 | -0.4128 | 0.9486 | 0.7758 | 0.7122 | 0.8735 | 0.5103 | 0.8042 | 0.9835 | 0.9012 | 0.8880 | -0.8738 | 0.8546 | 0.8836 | -0.9407 | 0.8792 0.9777 0.9761 -0.4221 0.6165 0.8384 0.5768 0.9015 0.8784 0.8034 0.3607 0.5738 0.9419 0.9448 0.9401 0.9770 -0.8406 0.9582 0.7168 0.9135 0.8886 0.8072 0.5480 0.8956 0.9211 0.9369 0.9202 -0.9230 -0.8891 0.9188 0.9162 -0.9237 0.8511 -0.9031 0.7984 0.8298 0.9131 0.9327 0.8651 0.9063 0.8434 0.8369 0.8469 0.9235 |
| Root Nα-acetyl-L-arginine | ##### | ###### | 0.6679 | 0.5298 | 0.6205 | -0.6924 | -0.7531 | 0.6851 | ##### | -0.5456 | -0.2960 | -0.4042 | -0.3376 | -0.4657 | -0.3857 | -0.5986 | -0.5613 | 0.0790 | -0.5458 | -0.4481 | -0.6572 | -0.8501 | -0.6145 | -0.6649 | -0.5380 | -0.5206 | -0.5190 | -0.6207 | 0.0861 | -0.3429 | -0.5186 | -0.6500 | 0.6591 | -0.5215 | 0.6141 | -0.4937 | -0.5787 | -0.5545 | 0.4223 | -0.6549 | -0.3673 | -0.4014 | 0.5281 | -0.6599 | -0.5775 | -0.4421 | -0.4128 | 1.0000 | -0.1245 | -0.6161 | -0.4967 | -0.7957 | -0.0748 | -0.5879 | -0.2659 | -0.1789 | -0.6524 | 0.6479 | -0.5543 | -0.5557 | 0.6642 | -0.5421 -0.5472 -0.5515 0.5506 -0.4416 -0.8204 -0.0484 -0.6814 -0.4577 -0.3449 0.2983 -0.7568 -0.5502 -0.6557 -0.6000 -0.5476 0.5901 -0.5994 -0.8183 -0.7117 -0.6471 -0.7054 -0.5286 -0.6916 -0.6620 -0.6110 -0.6571 0.6707 0.6787 -0.7117 -0.6367 0.6481 -0.4310 0.6548 -0.8473 -0.7027 -0.6709 -0.7035 -0.4931 -0.7327 -0.6940 -0.6379 -0.8021 -0.7153 |
| Root N-glycyl-L-leucine | ##### | 0.7589 | -0.7788 | -0.7241 | -0.6930 | 0.3710 | 0.6753 | -0.7863 | ##### | 0.8572 | 0.9166 | 0.5395 | 0.9508 | 0.6807 | 0.7775 | 0.8294 | 0.8390 | -0.2209 | 0.6083 | 0.8783 | 0.8051 | 0.6226 | 0.7352 | 0.5675 | 0.8165 | 0.7005 | 0.8631 | 0.6807 | -0.5707 | 0.8168 | 0.8693 | 0.6933 | -0.7193 | 0.8944 | -0.6874 | 0.7419 | 0.8437 | 0.6589 | -0.4425 | 0.7714 | 0.8682 | 0.9325 | -0.8070 | 0.8167 | 0.8380 | 0.7625 | 0.9486 | -0.1245 | 1.0000 | 0.5841 | 0.6003 | 0.6961 | 0.4834 | 0.6680 | 0.9671 | 0.9119 | 0.7183 | -0.7044 | 0.7230 | 0.7875 | -0.7858 | 0.7580 0.8648 0.8653 -0.2740 0.4937 0.6588 0.6087 0.7586 0.7981 0.7135 0.4006 0.4254 0.8212 0.7942 0.8195 0.8737 -0.7110 0.8369 0.4892 0.7424 0.7629 0.6627 0.3790 0.7142 0.7550 0.7878 0.7546 -0.7598 -0.7401 0.7476 0.7523 -0.7659 0.7585 -0.7454 0.5714 0.6375 0.7514 0.7775 0.7789 0.7333 0.6821 0.6892 0.6352 0.7551 |
| Root γ-glutamate-cysteine | ##### | 0.7955 | -0.9022 | -0.9779 | -0.9863 | 0.5755 | 0.7233 | -0.8889 | ##### | 0.7949 | 0.8103 | 0.9054 | 0.7787 | 0.9814 | 0.9355 | 0.8199 | 0.8671 | -0.4319 | 0.4116 | 0.8984 | 0.8450 | 0.8345 | 0.6728 | 0.5108 | 0.7634 | 0.5645 | 0.8531 | 0.6434 | -0.7977 | 0.7818 | 0.8554 | 0.6340 | -0.8438 | 0.8511 | -0.9811 | 0.9476 | 0.8392 | 0.5592 | -0.8940 | 0.6289 | 0.9001 | 0.6515 | -0.7286 | 0.8506 | 0.7502 | 0.9358 | 0.7758 | -0.6161 | 0.5841 | 1.0000 | 0.4938 | 0.7848 | 0.7211 | 0.6318 | 0.7501 | 0.7482 | 0.9707 | -0.9739 | 0.9367 | 0.6048 | -0.8894 | 0.9242 0.8787 0.8859 -0.2741 0.8529 0.7769 0.5823 0.7283 0.6131 0.9249 0.5485 0.3278 0.9284 0.8789 0.7571 0.8020 -0.6503 0.7980 0.7422 0.9214 0.6633 0.5880 0.8812 0.9477 0.9325 0.9361 0.9375 -0.9365 -0.8913 0.9115 0.9437 -0.9448 0.6569 -0.9457 0.8509 0.9774 0.8460 0.8354 0.5877 0.9067 0.7070 0.6833 0.9067 0.8854 |
| Root Nα-acetyl-L-glutamine | ##### | 0.8844 | -0.7564 | -0.4876 | -0.5529 | 0.9364 | 0.8945 | -0.7107 | ##### | 0.8542 | 0.7188 | 0.1216 | 0.7274 | 0.4632 | 0.4122 | 0.8455 | 0.8013 | -0.6538 | 0.9735 | 0.6548 | 0.8076 | 0.6772 | 0.9539 | 0.9679 | 0.9076 | 0.9884 | 0.7990 | 0.9730 | -0.0319 | 0.8401 | 0.7863 | 0.9569 | -0.8772 | 0.6898 | -0.4923 | 0.4005 | 0.8206 | 0.9923 | -0.0631 | 0.8681 | 0.6110 | 0.8277 | -0.9281 | 0.7577 | 0.8812 | 0.3880 | 0.7122 | -0.4967 | 0.6003 | 0.4938 | 1.0000 | 0.7679 | -0.1411 | 0.9830 | 0.6907 | 0.3503 | 0.5911 | -0.5469 | 0.3904 | 0.9383 | -0.7736 | 0.4149 0.7341 0.6768 -0.9284 0.0344 0.6450 -0.1576 0.8918 0.9590 0.2996 -0.0152 0.7812 0.5730 0.7864 0.8872 0.8223 -0.9718 0.8488 0.8841 0.6734 0.9115 0.9194 0.0487 0.7093 0.7301 0.7055 0.7207 -0.6742 -0.5113 0.7154 0.7215 -0.6494 0.9588 -0.5659 0.7850 0.5213 0.8573 0.7976 0.9639 0.6596 0.9382 0.9618 0.7340 0.7570 |
| Root N-acetyl-L-tyrosine | ##### | 0.9613 | -0.9561 | -0.8036 | -0.8546 | 0.7665 | 0.9723 | -0.9678 | ##### | 0.9271 | 0.7759 | 0.5794 | 0.8247 | 0.7330 | 0.7279 | 0.9467 | 0.9215 | -0.2365 | 0.8044 | 0.8513 | 0.9692 | 0.9866 | 0.9174 | 0.8614 | 0.9047 | 0.8383 | 0.9060 | 0.8934 | -0.3714 | 0.7645 | 0.9079 | 0.9190 | -0.9294 | 0.9146 | -0.8412 | 0.7807 | 0.9384 | 0.8387 | -0.5319 | 0.9574 | 0.7859 | 0.8696 | -0.8951 | 0.9726 | 0.9413 | 0.7558 | 0.8735 | -0.7957 | 0.6961 | 0.7848 | 0.7679 | 1.0000 | 0.2989 | 0.8649 | 0.7814 | 0.6576 | 0.8946 | -0.8794 | 0.8108 | 0.9056 | -0.9593 | 0.8245 0.9196 0.9171 -0.6180 0.5715 0.9841 0.3509 0.9695 0.8463 0.6533 0.0115 0.8289 0.8809 0.9596 0.9469 0.9345 -0.8891 0.9520 0.9117 0.9573 0.9511 0.9367 0.5680 0.9310 0.9358 0.9167 0.9313 -0.9389 -0.9179 0.9645 0.9160 -0.9243 0.8053 -0.9092 0.9664 0.8769 0.9534 0.9846 0.8607 0.9655 0.9401 0.9083 0.9655 0.9756 |
| Root D-alanyl-D-alanine | ##### | 0.2723 | -0.4851 | -0.7747 | -0.7023 | -0.1389 | 0.1425 | -0.5053 | ##### | 0.3589 | 0.5537 | 0.9365 | 0.5190 | 0.7945 | 0.8404 | 0.3688 | 0.4616 | -0.0078 | -0.2119 | 0.6434 | 0.4009 | 0.3782 | 0.0994 | -0.1487 | 0.2731 | -0.0286 | 0.4675 | 0.0335 | -0.9769 | 0.3856 | 0.4818 | 0.0345 | -0.3145 | 0.5592 | -0.7422 | 0.8244 | 0.4168 | -0.0599 | -0.9290 | 0.1246 | 0.6923 | 0.2937 | -0.2211 | 0.4479 | 0.2792 | 0.8410 | 0.5103 | -0.0748 | 0.4834 | 0.7211 | -0.1411 | 0.2989 | 1.0000 | 0.0166 | 0.5396 | 0.7933 | 0.6601 | -0.6926 | 0.8007 | 0.0749 | -0.4626 | 0.7853 0.5368 0.5867 0.4428 0.9240 0.3644 0.9248 0.1965 0.0931 0.8998 0.7812 -0.2659 0.6919 0.4473 0.2697 0.3959 -0.0594 0.3468 0.0857 0.5441 0.1251 -0.0073 0.8799 0.5374 0.5281 0.5738 0.5411 -0.5743 -0.6425 0.5043 0.5505 -0.6086 0.1285 -0.6635 0.2948 0.6784 0.3412 0.3782 0.0487 0.5316 0.1086 0.0817 0.4235 0.4485 |
| Root homo-Arg | ##### | 0.9545 | -0.8625 | -0.6300 | -0.6907 | 0.9361 | 0.9554 | -0.8264 | ##### | 0.9266 | 0.7973 | 0.2909 | 0.8092 | 0.5990 | 0.5544 | 0.9248 | 0.8906 | -0.6095 | 0.9591 | 0.7661 | 0.9001 | 0.7939 | 0.9902 | 0.9730 | 0.9620 | 0.9938 | 0.8852 | 0.9978 | -0.1760 | 0.8879 | 0.8757 | 0.9881 | -0.9480 | 0.7994 | -0.6394 | 0.5538 | 0.9058 | 0.9957 | -0.2337 | 0.9238 | 0.7208 | 0.8831 | -0.9731 | 0.8614 | 0.9456 | 0.5391 | 0.8042 | -0.5879 | 0.6680 | 0.6318 | 0.9830 | 0.8649 | 0.0166 | 1.0000 | 0.7703 | 0.4770 | 0.7264 | -0.6886 | 0.5489 | 0.9698 | -0.8756 | 0.5701 0.8381 0.7925 -0.8737 0.2136 0.7624 -0.0006 0.9548 0.9783 0.4502 0.0534 0.7969 0.7072 0.8849 0.9516 0.9033 -0.9975 0.9262 0.9362 0.7974 0.9610 0.9556 0.2253 0.8243 0.8409 0.8192 0.8331 -0.7971 -0.6582 0.8313 0.8320 -0.7759 0.9755 -0.7063 0.8780 0.6669 0.9366 0.8930 0.9815 0.7859 0.9838 0.9954 0.8438 0.8638 |
| Root L-carnosine | ##### | 0.8613 | -0.8893 | -0.8469 | -0.8295 | 0.5292 | 0.7728 | -0.8804 | ##### | 0.9370 | 0.9872 | 0.6481 | 0.9969 | 0.8212 | 0.8722 | 0.9191 | 0.9380 | -0.3979 | 0.6587 | 0.9629 | 0.8980 | 0.7265 | 0.8221 | 0.6500 | 0.9085 | 0.7782 | 0.9540 | 0.7776 | -0.6530 | 0.9248 | 0.9569 | 0.7770 | -0.8506 | 0.9559 | -0.8144 | 0.8380 | 0.9332 | 0.7450 | -0.5661 | 0.8131 | 0.9580 | 0.9517 | -0.8950 | 0.8990 | 0.9100 | 0.8517 | 0.9835 | -0.2659 | 0.9671 | 0.7501 | 0.6907 | 0.7814 | 0.5396 | 0.7703 | 1.0000 | 0.9134 | 0.8446 | -0.8292 | 0.8131 | 0.8389 | -0.8932 | 0.8383 0.9497 0.9423 -0.3761 0.5803 0.7297 0.5789 0.8388 0.8632 0.8103 0.4876 0.4500 0.9089 0.8985 0.8982 0.9450 -0.8037 0.9173 0.6430 0.8520 0.8271 0.7283 0.5044 0.8498 0.8794 0.9044 0.8797 -0.8736 -0.8164 0.8594 0.8826 -0.8788 0.8536 -0.8502 0.7156 0.7742 0.8686 0.8691 0.8385 0.8362 0.7858 0.7929 0.7741 0.8624 |
| Root glycylphenylalanine | ##### | 0.6688 | -0.7823 | -0.8694 | -0.8191 | 0.2018 | 0.5606 | -0.8032 | ##### | 0.7689 | 0.8798 | 0.8180 | 0.8963 | 0.8391 | 0.9296 | 0.7580 | 0.8029 | -0.1077 | 0.3373 | 0.9094 | 0.7625 | 0.6480 | 0.5681 | 0.3441 | 0.6965 | 0.4765 | 0.8195 | 0.4983 | -0.8333 | 0.7272 | 0.8314 | 0.5135 | -0.6540 | 0.8928 | -0.8368 | 0.9089 | 0.7886 | 0.4351 | -0.7483 | 0.6206 | 0.9169 | 0.7890 | -0.6652 | 0.7961 | 0.7230 | 0.9272 | 0.9012 | -0.1789 | 0.9119 | 0.7482 | 0.3503 | 0.6576 | 0.7933 | 0.4770 | 0.9134 | 1.0000 | 0.8214 | -0.8280 | 0.8928 | 0.5945 | -0.7765 | 0.9097 0.8587 0.8859 0.0078 0.7916 0.6713 0.8603 0.6390 0.5967 0.9117 0.5872 0.2194 0.9047 0.7746 0.7067 0.8002 -0.5288 0.7549 0.4019 0.7915 0.6083 0.4838 0.6930 0.7606 0.7831 0.8237 0.7884 -0.8123 -0.8436 0.7751 0.7879 -0.8309 0.5768 -0.8470 0.5627 0.7793 0.6930 0.7384 0.5676 0.7835 0.5409 0.5270 0.6637 0.7546 |
| Root S-(5-adenosy)-L-homocysteine | ##### | 0.8884 | -0.9707 | -0.9836 | -0.9963 | 0.6163 | 0.8312 | -0.9707 | ##### | 0.8935 | 0.8728 | 0.8630 | 0.8726 | 0.9578 | 0.9441 | 0.9133 | 0.9411 | -0.3444 | 0.5524 | 0.9560 | 0.9370 | 0.9195 | 0.7834 | 0.6315 | 0.8549 | 0.6773 | 0.9303 | 0.7463 | -0.7339 | 0.8256 | 0.9344 | 0.7518 | -0.9045 | 0.9454 | -0.9925 | 0.9655 | 0.9274 | 0.6674 | -0.8301 | 0.7801 | 0.9373 | 0.7925 | -0.8255 | 0.9475 | 0.8637 | 0.9534 | 0.8880 | -0.6524 | 0.7183 | 0.9707 | 0.5911 | 0.8946 | 0.6601 | 0.7264 | 0.8446 | 0.8214 | 1.0000 | -0.9985 | 0.9664 | 0.7434 | -0.9629 | 0.9659 0.9574 0.9660 -0.3534 0.8260 0.8885 0.6055 0.8461 0.7289 0.9118 0.4272 0.4938 0.9840 0.9563 0.8669 0.9057 -0.7540 0.9014 0.7969 0.9852 0.7960 0.7259 0.8255 0.9868 0.9826 0.9854 0.9851 -0.9915 -0.9649 0.9784 0.9837 -0.9950 0.7383 -0.9944 0.9073 0.9923 0.9204 0.9325 0.7137 0.9780 0.8071 0.7799 0.9575 0.9626 |
| Root argininosuccinic acid | ##### | ###### | 0.9574 | 0.9875 | 0.9968 | -0.5777 | -0.8046 | 0.9609 | ##### | -0.8704 | -0.8561 | -0.8867 | -0.8561 | -0.9617 | -0.9510 | -0.8917 | -0.9230 | 0.3100 | -0.5096 | -0.9476 | -0.9192 | -0.9115 | -0.7501 | -0.5924 | -0.8265 | -0.6372 | -0.9118 | -0.7103 | 0.7569 | -0.7988 | -0.9170 | -0.7175 | 0.8803 | -0.9353 | 0.9971 | -0.9748 | -0.9078 | -0.6269 | 0.8565 | -0.7535 | -0.9304 | -0.7674 | 0.7947 | -0.9337 | -0.8381 | -0.9629 | -0.8738 | 0.6479 | -0.7044 | -0.9739 | -0.5469 | -0.8794 | -0.6926 | -0.6886 | -0.8292 | -0.8280 | -0.9985 | 1.0000 | -0.9771 | -0.7098 | 0.9482 | -0.9750 -0.9445 -0.9575 0.3059 -0.8550 -0.8818 -0.6383 -0.8199 -0.6924 -0.9249 -0.4392 -0.4643 -0.9830 -0.9404 -0.8411 -0.8853 0.7181 -0.8793 -0.7694 -0.9784 -0.7666 -0.6940 -0.8536 -0.9770 -0.9712 -0.9755 -0.9744 0.9845 0.9687 -0.9685 -0.9726 0.9896 -0.7014 0.9948 -0.8909 -0.9955 -0.8984 -0.9157 -0.6766 -0.9722 -0.7761 -0.7458 -0.9459 -0.9494 |
| Root succinic acid | ##### | 0.7675 | -0.8959 | -0.9789 | -0.9674 | 0.3961 | 0.6962 | -0.9159 | ##### | 0.7970 | 0.8192 | 0.9400 | 0.8284 | 0.9467 | 0.9698 | 0.8164 | 0.8575 | -0.1497 | 0.3701 | 0.9244 | 0.8514 | 0.8531 | 0.6326 | 0.4467 | 0.7303 | 0.5031 | 0.8506 | 0.5775 | -0.8306 | 0.7097 | 0.8606 | 0.5944 | -0.7740 | 0.9126 | -0.9838 | 0.9948 | 0.8405 | 0.4849 | -0.9071 | 0.6735 | 0.9129 | 0.7181 | -0.6920 | 0.8826 | 0.7572 | 0.9886 | 0.8546 | -0.5543 | 0.7230 | 0.9367 | 0.3904 | 0.8108 | 0.8007 | 0.5489 | 0.8131 | 0.8928 | 0.9664 | -0.9771 | 1.0000 | 0.6088 | -0.8838 | 0.9984 0.9025 0.9319 -0.1147 0.9285 0.8423 0.7851 0.7259 0.5815 0.9604 0.4816 0.3572 0.9750 0.8743 0.7554 0.8244 -0.5901 0.8060 0.6261 0.9342 0.6698 0.5808 0.9014 0.9136 0.9100 0.9253 0.9151 -0.9406 -0.9704 0.9140 0.9111 -0.9524 0.5779 -0.9774 0.7893 0.9684 0.8053 0.8480 0.5637 0.9320 0.6541 0.6168 0.8670 0.8863 |
| Rott 5-aminovaleric acid | ##### | 0.9613 | -0.8820 | -0.6489 | -0.6967 | 0.8349 | 0.9618 | -0.8680 | ##### | 0.9549 | 0.8286 | 0.3237 | 0.8691 | 0.5925 | 0.5956 | 0.9486 | 0.9095 | -0.4309 | 0.9564 | 0.8074 | 0.9296 | 0.8269 | 0.9897 | 0.9541 | 0.9665 | 0.9789 | 0.9094 | 0.9793 | -0.2066 | 0.8646 | 0.9050 | 0.9874 | -0.9255 | 0.8658 | -0.6590 | 0.6031 | 0.9327 | 0.9715 | -0.2493 | 0.9802 | 0.7521 | 0.9561 | -0.9755 | 0.9078 | 0.9774 | 0.5922 | 0.8836 | -0.5557 | 0.7875 | 0.6048 | 0.9383 | 0.9056 | 0.0749 | 0.9698 | 0.8389 | 0.5945 | 0.7434 | -0.7098 | 0.6088 | 1.0000 | -0.8960 | 0.6383 0.8790 0.8477 -0.7849 0.2722 0.8274 0.1436 0.9793 0.9846 0.4893 0.0127 0.8367 0.7603 0.9064 0.9773 0.9451 -0.9832 0.9580 0.8788 0.8280 0.9920 0.9760 0.2447 0.8231 0.8485 0.8348 0.8407 -0.8219 -0.7313 0.8557 0.8325 -0.8028 0.9488 -0.7480 0.8572 0.6783 0.9341 0.9262 0.9929 0.8247 0.9734 0.9763 0.8380 0.8879 |
| Root α-aminoadipic acid | ##### | ###### | 0.9995 | 0.9128 | 0.9418 | -0.7528 | -0.9414 | 0.9931 | ##### | -0.9766 | -0.9095 | -0.7006 | -0.9249 | -0.8735 | -0.8629 | -0.9877 | -0.9901 | 0.4086 | -0.7541 | -0.9567 | -0.9960 | -0.9449 | -0.9200 | -0.8071 | -0.9568 | -0.8456 | -0.9824 | -0.8923 | 0.5655 | -0.8957 | -0.9831 | -0.8987 | 0.9752 | -0.9729 | 0.9235 | -0.8807 | -0.9903 | -0.8369 | 0.6499 | -0.9120 | -0.9218 | -0.9073 | 0.9411 | -0.9935 | -0.9662 | -0.8678 | -0.9407 | 0.6642 | -0.7858 | -0.8894 | -0.7736 | -0.9593 | -0.4626 | -0.8756 | -0.8932 | -0.7765 | -0.9629 | 0.9482 | -0.8838 | -0.8960 | 1.0000 | -0.8949 -0.9875 -0.9800 0.5579 -0.6548 -0.9230 -0.4504 -0.9567 -0.8804 -0.7986 -0.2849 -0.6676 -0.9567 -0.9997 -0.9688 -0.9801 0.8985 -0.9834 -0.8887 -0.9873 -0.9282 -0.8767 -0.6459 -0.9875 -0.9943 -0.9898 -0.9928 0.9889 0.9328 -0.9942 -0.9887 0.9831 -0.8733 0.9600 -0.9514 -0.9323 -0.9884 -0.9920 -0.8738 -0.9811 -0.9303 -0.9134 -0.9755 -0.9969 |
| Root 2-aminobutyric acid | ##### | 0.7842 | -0.9056 | -0.9755 | -0.9634 | 0.4028 | 0.7133 | -0.9266 | ##### | 0.8179 | 0.8381 | 0.9237 | 0.8522 | 0.9392 | 0.9698 | 0.8345 | 0.8723 | -0.1445 | 0.4008 | 0.9366 | 0.8668 | 0.8588 | 0.6555 | 0.4700 | 0.7510 | 0.5298 | 0.8673 | 0.5991 | -0.8179 | 0.7259 | 0.8774 | 0.6176 | -0.7847 | 0.9302 | -0.9791 | 0.9930 | 0.8578 | 0.5098 | -0.8850 | 0.7011 | 0.9227 | 0.7506 | -0.7145 | 0.8979 | 0.7805 | 0.9881 | 0.8792 | -0.5421 | 0.7580 | 0.9242 | 0.4149 | 0.8245 | 0.7853 | 0.5701 | 0.8383 | 0.9097 | 0.9659 | -0.9750 | 0.9984 | 0.6383 | -0.8949 | 1.0000 0.9173 0.9458 -0.1323 0.9122 0.8532 0.7859 0.7470 0.6101 0.9551 0.4681 0.3823 0.9822 0.8866 0.7774 0.8456 -0.6128 0.8261 0.6309 0.9401 0.6950 0.6053 0.8774 0.9161 0.9159 0.9321 0.9205 -0.9461 -0.9758 0.9212 0.9157 -0.9571 0.6008 -0.9800 0.7917 0.9622 0.8180 0.8628 0.5933 0.9384 0.6724 0.6365 0.8689 0.8962 |
| Root (S)-β-aminoisobutyric acid | ##### | 0.9521 | -0.9872 | -0.9287 | -0.9396 | 0.6697 | 0.8941 | -0.9825 | ##### | 0.9786 | 0.9559 | 0.7311 | 0.9695 | 0.8930 | 0.9055 | 0.9816 | 0.9932 | -0.3914 | 0.7103 | 0.9876 | 0.9835 | 0.8973 | 0.8905 | 0.7474 | 0.9505 | 0.8178 | 0.9929 | 0.8536 | -0.6390 | 0.9161 | 0.9954 | 0.8592 | -0.9474 | 0.9943 | -0.9253 | 0.9080 | 0.9901 | 0.7999 | -0.6703 | 0.8873 | 0.9634 | 0.9334 | -0.9327 | 0.9855 | 0.9597 | 0.9039 | 0.9777 | -0.5472 | 0.8648 | 0.8787 | 0.7341 | 0.9196 | 0.5368 | 0.8381 | 0.9497 | 0.8587 | 0.9574 | -0.9445 | 0.9025 | 0.8790 | -0.9875 | 0.9173 1.0000 0.9961 -0.4732 0.6804 0.8841 0.5411 0.9278 0.8743 0.8453 0.3786 0.5894 0.9753 0.9881 0.9572 0.9862 -0.8671 0.9774 0.8127 0.9716 0.9011 0.8289 0.6475 0.9687 0.9821 0.9890 0.9818 -0.9801 -0.9308 0.9762 0.9797 -0.9794 0.8663 -0.9587 0.8910 0.9162 0.9645 0.9714 0.8607 0.9626 0.8872 0.8753 0.9324 0.9760 |
| Root γ-aminobutyric acid | ##### | 0.9300 | -0.9814 | -0.9425 | -0.9486 | 0.6142 | 0.8711 | -0.9850 | ##### | 0.9596 | 0.9414 | 0.7708 | 0.9605 | 0.9017 | 0.9243 | 0.9648 | 0.9794 | -0.3194 | 0.6624 | 0.9877 | 0.9734 | 0.9037 | 0.8555 | 0.7036 | 0.9211 | 0.7707 | 0.9787 | 0.8126 | -0.6723 | 0.8810 | 0.9832 | 0.8237 | -0.9210 | 0.9975 | -0.9416 | 0.9330 | 0.9760 | 0.7514 | -0.7112 | 0.8706 | 0.9626 | 0.9167 | -0.8998 | 0.9832 | 0.9387 | 0.9288 | 0.9761 | -0.5515 | 0.8653 | 0.8859 | 0.6768 | 0.9171 | 0.5867 | 0.7925 | 0.9423 | 0.8859 | 0.9660 | -0.9575 | 0.9319 | 0.8477 | -0.9800 | 0.9458 0.9961 1.0000 -0.4066 0.7317 0.8969 0.6046 0.9068 0.8337 0.8710 0.3811 0.5691 0.9885 0.9791 0.9354 0.9728 -0.8263 0.9613 0.7799 0.9766 0.8767 0.8008 0.6931 0.9646 0.9768 0.9859 0.9772 -0.9832 -0.9555 0.9762 0.9732 -0.9845 0.8196 -0.9737 0.8781 0.9310 0.9443 0.9640 0.8209 0.9705 0.8550 0.8369 0.9273 0.9719 |
| Root 4-acetamidobutyric acid | ##### | ###### | 0.5384 | 0.2145 | 0.3103 | -0.9405 | -0.7755 | 0.4886 | ##### | -0.6288 | -0.4173 | 0.1493 | -0.4279 | -0.1909 | -0.0992 | -0.6303 | -0.5626 | 0.6071 | -0.9179 | -0.3606 | -0.5980 | -0.5358 | -0.8147 | -0.9222 | -0.7080 | -0.8729 | -0.5489 | -0.8605 | -0.2856 | -0.5999 | -0.5319 | -0.8439 | 0.7057 | -0.4146 | 0.2428 | -0.1098 | -0.5908 | -0.8965 | -0.1806 | -0.7188 | -0.3021 | -0.5817 | 0.7396 | -0.5365 | -0.6787 | -0.0840 | -0.4221 | 0.5506 | -0.2740 | -0.2741 | -0.9284 | -0.6180 | 0.4428 | -0.8737 | -0.3761 | 0.0078 | -0.3534 | 0.3059 | -0.1147 | -0.7849 | 0.5579 | -0.1323 -0.4732 -0.4066 1.0000 0.2193 -0.4941 0.4792 -0.7349 -0.7901 0.0130 0.2911 -0.8013 -0.2936 -0.5706 -0.6934 -0.5860 0.8439 -0.6359 -0.8286 -0.4574 -0.7636 -0.8271 0.1596 -0.4998 -0.5093 -0.4629 -0.4971 0.4442 0.2737 -0.5058 -0.4948 0.4092 -0.7905 0.3209 -0.6722 -0.3049 -0.6739 -0.6037 -0.8116 -0.4502 -0.8182 -0.8397 -0.5764 -0.5565 |
| Root 6-aminocaproic acid | ##### | 0.4845 | -0.6761 | -0.8801 | -0.8473 | 0.0980 | 0.4036 | -0.7089 | ##### | 0.5173 | 0.5918 | 0.9852 | 0.5872 | 0.8641 | 0.8889 | 0.5459 | 0.6115 | 0.0372 | 0.0068 | 0.7358 | 0.6002 | 0.6623 | 0.3068 | 0.1044 | 0.4304 | 0.1521 | 0.6014 | 0.2455 | -0.8947 | 0.4432 | 0.6158 | 0.2642 | -0.5069 | 0.6990 | -0.8856 | 0.9212 | 0.5815 | 0.1352 | -0.9819 | 0.3657 | 0.7439 | 0.4180 | -0.3799 | 0.6491 | 0.4610 | 0.9166 | 0.6165 | -0.4416 | 0.4937 | 0.8529 | 0.0344 | 0.5715 | 0.9240 | 0.2136 | 0.5803 | 0.7916 | 0.8260 | -0.8550 | 0.9285 | 0.2722 | -0.6548 | 0.9122 0.6804 0.7317 0.2193 1.0000 0.6496 0.8762 0.4279 0.2406 0.9267 0.5485 0.0631 0.8249 0.6381 0.4597 0.5556 -0.2572 0.5296 0.3598 0.7508 0.3536 0.2579 0.9832 0.7256 0.7080 0.7306 0.7176 -0.7588 -0.8415 0.7129 0.7152 -0.7810 0.2501 -0.8385 0.5681 0.8621 0.5419 0.6005 0.2176 0.7514 0.3438 0.2945 0.6685 0.6646 |
| Root creatine-phosphate | ##### | 0.9005 | -0.9231 | -0.8026 | -0.8481 | 0.6580 | 0.9172 | -0.9517 | ##### | 0.8650 | 0.7110 | 0.6307 | 0.7721 | 0.7205 | 0.7309 | 0.8908 | 0.8683 | -0.0802 | 0.7044 | 0.8190 | 0.9286 | 0.9904 | 0.8337 | 0.7715 | 0.8239 | 0.7315 | 0.8501 | 0.8004 | -0.4005 | 0.6648 | 0.8559 | 0.8379 | -0.8593 | 0.8920 | -0.8489 | 0.8004 | 0.8860 | 0.7317 | -0.5891 | 0.9115 | 0.7489 | 0.8090 | -0.8084 | 0.9467 | 0.8785 | 0.7732 | 0.8384 | -0.8204 | 0.6588 | 0.7769 | 0.6450 | 0.9841 | 0.3644 | 0.7624 | 0.7297 | 0.6713 | 0.8885 | -0.8818 | 0.8423 | 0.8274 | -0.9230 | 0.8532 0.8841 0.8969 -0.4941 0.6496 1.0000 0.4432 0.9133 0.7459 0.6702 -0.0224 0.7977 0.8783 0.9204 0.8836 0.8828 -0.7946 0.8980 0.8440 0.9474 0.8888 0.8744 0.6402 0.9024 0.9031 0.8854 0.8997 -0.9227 -0.9460 0.9451 0.8797 -0.9107 0.6915 -0.9157 0.9334 0.8865 0.8948 0.9519 0.7645 0.9627 0.8650 0.8196 0.9423 0.9489 |
| Root kynurenic acid | ##### | 0.2724 | -0.4675 | -0.6983 | -0.6211 | -0.2424 | 0.1666 | -0.5196 | ##### | 0.3747 | 0.5318 | 0.8452 | 0.5504 | 0.6712 | 0.7832 | 0.3771 | 0.4446 | 0.2686 | -0.1475 | 0.6258 | 0.4142 | 0.4045 | 0.1163 | -0.1177 | 0.2657 | -0.0153 | 0.4566 | 0.0330 | -0.8641 | 0.3073 | 0.4765 | 0.0632 | -0.2594 | 0.5992 | -0.6750 | 0.7861 | 0.4209 | -0.0554 | -0.8108 | 0.2290 | 0.6471 | 0.3882 | -0.2192 | 0.4797 | 0.3156 | 0.8044 | 0.5768 | -0.0484 | 0.6087 | 0.5823 | -0.1576 | 0.3509 | 0.9248 | -0.0006 | 0.5789 | 0.8603 | 0.6055 | -0.6383 | 0.7851 | 0.1436 | -0.4504 | 0.7859 0.5411 0.6046 0.4792 0.8762 0.4432 1.0000 0.2384 0.1177 0.8260 0.5741 -0.1207 0.6867 0.4393 0.2986 0.4268 -0.0605 0.3720 0.0180 0.5342 0.1889 0.0637 0.7789 0.4772 0.4832 0.5322 0.4937 -0.5489 -0.6808 0.4917 0.4894 -0.5795 0.0929 -0.6477 0.2501 0.6119 0.3140 0.4024 0.0901 0.5375 0.1103 0.0725 0.3791 0.4458 |
| Root N'-formylkynurenine | ##### | 0.9924 | -0.9480 | -0.7547 | -0.8051 | 0.8420 | 0.9928 | -0.9406 | ##### | 0.9724 | 0.8389 | 0.4680 | 0.8764 | 0.6970 | 0.6880 | 0.9784 | 0.9488 | -0.3965 | 0.9066 | 0.8597 | 0.9767 | 0.9215 | 0.9857 | 0.9355 | 0.9717 | 0.9438 | 0.9411 | 0.9707 | -0.3111 | 0.8610 | 0.9388 | 0.9827 | -0.9653 | 0.9131 | -0.7754 | 0.7128 | 0.9664 | 0.9405 | -0.4068 | 0.9859 | 0.8027 | 0.9364 | -0.9715 | 0.9632 | 0.9882 | 0.6954 | 0.9015 | -0.6814 | 0.7586 | 0.7283 | 0.8918 | 0.9695 | 0.1965 | 0.9548 | 0.8388 | 0.6390 | 0.8461 | -0.8199 | 0.7259 | 0.9793 | -0.9567 | 0.7470 0.9278 0.9068 -0.7349 0.4279 0.9133 0.2384 1.0000 0.9505 0.5954 0.0496 0.8348 0.8436 0.9622 0.9915 0.9682 -0.9712 0.9837 0.9291 0.9162 0.9957 0.9775 0.4169 0.9086 0.9235 0.9071 0.9174 -0.9070 -0.8361 0.9356 0.9078 -0.8902 0.9200 -0.8499 0.9395 0.8017 0.9773 0.9797 0.9589 0.9155 0.9882 0.9772 0.9291 0.9575 |
| Root 2-aminoethanesulfonic acid | ##### | 0.9475 | -0.8662 | -0.6525 | -0.6923 | 0.8453 | 0.9264 | -0.8360 | ##### | 0.9548 | 0.8696 | 0.3195 | 0.8877 | 0.6180 | 0.6098 | 0.9419 | 0.9139 | -0.5642 | 0.9412 | 0.8192 | 0.9094 | 0.7599 | 0.9827 | 0.9312 | 0.9779 | 0.9883 | 0.9184 | 0.9770 | -0.2504 | 0.9233 | 0.9115 | 0.9703 | -0.9303 | 0.8506 | -0.6464 | 0.5935 | 0.9289 | 0.9779 | -0.2447 | 0.9330 | 0.7815 | 0.9509 | -0.9877 | 0.8778 | 0.9670 | 0.5893 | 0.8784 | -0.4577 | 0.7981 | 0.6131 | 0.9590 | 0.8463 | 0.0931 | 0.9783 | 0.8632 | 0.5967 | 0.7289 | -0.6924 | 0.5815 | 0.9846 | -0.8804 | 0.6101 0.8743 0.8337 -0.7901 0.2406 0.7459 0.1177 0.9505 1.0000 0.5053 0.1358 0.7451 0.7457 0.8917 0.9661 0.9379 -0.9856 0.9460 0.8586 0.7964 0.9624 0.9332 0.2160 0.8129 0.8402 0.8320 0.8332 -0.8020 -0.6769 0.8280 0.8326 -0.7858 0.9871 -0.7191 0.8205 0.6532 0.9273 0.8941 0.9973 0.7836 0.9574 0.9739 0.8061 0.8596 |
| Root ethanolamine | ##### | 0.6622 | -0.8133 | -0.9695 | -0.9332 | 0.2880 | 0.5499 | -0.8172 | ##### | 0.7236 | 0.8345 | 0.9684 | 0.8104 | 0.9717 | 0.9919 | 0.7334 | 0.8004 | -0.2605 | 0.2289 | 0.9068 | 0.7537 | 0.6961 | 0.5212 | 0.2947 | 0.6614 | 0.4054 | 0.8020 | 0.4657 | -0.9455 | 0.7236 | 0.8108 | 0.4637 | -0.6962 | 0.8485 | -0.9474 | 0.9812 | 0.7675 | 0.3793 | -0.9383 | 0.5181 | 0.9285 | 0.6431 | -0.6194 | 0.7798 | 0.6617 | 0.9881 | 0.8034 | -0.3449 | 0.7135 | 0.9249 | 0.2996 | 0.6533 | 0.8998 | 0.4502 | 0.8103 | 0.9117 | 0.9118 | -0.9249 | 0.9604 | 0.4893 | -0.7986 | 0.9551 0.8453 0.8710 0.0130 0.9267 0.6702 0.8260 0.5954 0.5053 1.0000 0.7063 0.1213 0.9291 0.7886 0.6565 0.7470 -0.4876 0.7145 0.4927 0.8403 0.5335 0.4153 0.8949 0.8445 0.8422 0.8721 0.8504 -0.8656 -0.8695 0.8190 0.8574 -0.8865 0.5343 -0.9069 0.6561 0.9050 0.7159 0.7323 0.4684 0.8254 0.5292 0.5082 0.7550 0.7830 |
| Root Cys | ##### | 0.1564 | -0.3002 | -0.5729 | -0.4925 | -0.0565 | -0.0142 | -0.2570 | ##### | 0.2692 | 0.5554 | 0.6562 | 0.4520 | 0.6646 | 0.6593 | 0.2517 | 0.3571 | -0.4841 | -0.1929 | 0.5136 | 0.2254 | 0.0381 | 0.0584 | -0.1772 | 0.2485 | 0.0258 | 0.3784 | 0.0274 | -0.8578 | 0.4975 | 0.3797 | -0.0246 | -0.2516 | 0.3578 | -0.4915 | 0.5619 | 0.2965 | -0.0079 | -0.6469 | -0.0662 | 0.6110 | 0.2073 | -0.2143 | 0.2242 | 0.1760 | 0.5979 | 0.3607 | 0.2983 | 0.4006 | 0.5485 | -0.0152 | 0.0115 | 0.7812 | 0.0534 | 0.4876 | 0.5872 | 0.4272 | -0.4392 | 0.4816 | 0.0127 | -0.2849 | 0.4681 0.3786 0.3811 0.2911 0.5485 -0.0224 0.5741 0.0496 0.1358 0.7063 1.0000 -0.5082 0.4522 0.2777 0.1658 0.2703 -0.0632 0.2175 -0.0049 0.2844 -0.0034 -0.1464 0.5248 0.3557 0.3565 0.4094 0.3689 -0.3481 -0.2731 0.2706 0.4023 -0.3829 0.2415 -0.3790 0.0858 0.4004 0.2302 0.1662 0.0648 0.2413 0.0310 0.0612 0.1952 0.2289 |
| Root creatine | ##### | 0.7703 | -0.6518 | -0.3340 | -0.4230 | 0.7652 | 0.8652 | -0.6680 | ##### | 0.6891 | 0.4149 | 0.0397 | 0.5028 | 0.2351 | 0.2271 | 0.7043 | 0.6207 | -0.0855 | 0.8898 | 0.4558 | 0.7172 | 0.7734 | 0.8190 | 0.9072 | 0.7014 | 0.8016 | 0.6019 | 0.8244 | 0.2077 | 0.4696 | 0.5989 | 0.8624 | -0.6961 | 0.5865 | -0.3971 | 0.3020 | 0.6690 | 0.8180 | 0.0068 | 0.8846 | 0.3532 | 0.6891 | -0.7202 | 0.7052 | 0.7541 | 0.2668 | 0.5738 | -0.7568 | 0.4254 | 0.3278 | 0.7812 | 0.8289 | -0.2659 | 0.7969 | 0.4500 | 0.2194 | 0.4938 | -0.4643 | 0.3572 | 0.8367 | -0.6676 | 0.3823 0.5894 0.5691 -0.8013 0.0631 0.7977 -0.1207 0.8348 0.7451 0.1213 -0.5082 1.0000 0.4754 0.6763 0.7631 0.6840 -0.8047 0.7273 0.8093 0.6325 0.8599 0.9240 0.0694 0.5884 0.6002 0.5562 0.5881 -0.5896 -0.5670 0.6572 0.5618 -0.5557 0.6624 -0.5222 0.7658 0.4708 0.7171 0.7524 0.7913 0.6552 0.8371 0.8114 0.6954 0.6992 |
| Root N,N-dimethylglycine | ##### | 0.8779 | -0.9627 | -0.9781 | -0.9759 | 0.5383 | 0.8092 | -0.9703 | ##### | 0.9096 | 0.9145 | 0.8580 | 0.9256 | 0.9442 | 0.9645 | 0.9201 | 0.9482 | -0.2789 | 0.5492 | 0.9823 | 0.9380 | 0.8901 | 0.7773 | 0.6056 | 0.8606 | 0.6744 | 0.9454 | 0.7294 | -0.7602 | 0.8361 | 0.9518 | 0.7405 | -0.8790 | 0.9782 | -0.9771 | 0.9764 | 0.9374 | 0.6550 | -0.8090 | 0.7960 | 0.9654 | 0.8484 | -0.8318 | 0.9551 | 0.8785 | 0.9722 | 0.9419 | -0.5502 | 0.8212 | 0.9284 | 0.5730 | 0.8809 | 0.6919 | 0.7072 | 0.9089 | 0.9047 | 0.9840 | -0.9830 | 0.9750 | 0.7603 | -0.9567 | 0.9822 0.9753 0.9885 -0.2936 0.8249 0.8783 0.6867 0.8436 0.7457 0.9291 0.4522 0.4754 1.0000 0.9522 0.8758 0.9277 -0.7436 0.9125 0.7291 0.9735 0.8014 0.7166 0.7920 0.9617 0.9674 0.9800 0.9700 -0.9821 -0.9718 0.9654 0.9673 -0.9883 0.7402 -0.9904 0.8537 0.9626 0.9039 0.9290 0.7287 0.9673 0.7850 0.7607 0.9164 0.9509 |
| Root 5-hydroxylysine | ##### | 0.9818 | -0.9985 | -0.9045 | -0.9339 | 0.7589 | 0.9458 | -0.9913 | ##### | 0.9816 | 0.9134 | 0.6852 | 0.9297 | 0.8647 | 0.8555 | 0.9914 | 0.9921 | -0.4142 | 0.7680 | 0.9559 | 0.9977 | 0.9411 | 0.9283 | 0.8175 | 0.9633 | 0.8574 | 0.9851 | 0.9011 | -0.5529 | 0.9015 | 0.9855 | 0.9074 | -0.9775 | 0.9736 | -0.9144 | 0.8718 | 0.9932 | 0.8482 | -0.6325 | 0.9196 | 0.9204 | 0.9167 | -0.9488 | 0.9940 | 0.9724 | 0.8593 | 0.9448 | -0.6557 | 0.7942 | 0.8789 | 0.7864 | 0.9596 | 0.4473 | 0.8849 | 0.8985 | 0.7746 | 0.9563 | -0.9404 | 0.8743 | 0.9064 | -0.9997 | 0.8866 0.9881 0.9791 -0.5706 0.6381 0.9204 0.4393 0.9622 0.8917 0.7886 0.2777 0.6763 0.9522 1.0000 0.9746 0.9844 -0.9076 0.9877 0.8903 0.9834 0.9359 0.8850 0.6272 0.9836 0.9918 0.9873 0.9899 -0.9850 -0.9263 0.9915 0.9858 -0.9788 0.8834 -0.9537 0.9490 0.9229 0.9905 0.9931 0.8854 0.9770 0.9365 0.9210 0.9716 0.9957 |
| Root TFAADs | ##### | 0.9937 | -0.9607 | -0.7949 | -0.8325 | 0.8141 | 0.9719 | -0.9481 | ##### | 0.9939 | 0.9012 | 0.5138 | 0.9281 | 0.7479 | 0.7455 | 0.9940 | 0.9756 | -0.4476 | 0.8833 | 0.9065 | 0.9851 | 0.8945 | 0.9826 | 0.9045 | 0.9916 | 0.9437 | 0.9732 | 0.9634 | -0.3948 | 0.9143 | 0.9709 | 0.9691 | -0.9744 | 0.9426 | -0.8022 | 0.7537 | 0.9873 | 0.9341 | -0.4473 | 0.9692 | 0.8624 | 0.9637 | -0.9891 | 0.9709 | 0.9994 | 0.7432 | 0.9401 | -0.6000 | 0.8195 | 0.7571 | 0.8872 | 0.9469 | 0.2697 | 0.9516 | 0.8982 | 0.7067 | 0.8669 | -0.8411 | 0.7554 | 0.9773 | -0.9688 | 0.7774 0.9572 0.9354 -0.6934 0.4597 0.8836 0.2986 0.9915 0.9661 0.6565 0.1658 0.7631 0.8758 0.9746 1.0000 0.9896 -0.9698 0.9966 0.8997 0.9226 0.9853 0.9484 0.4380 0.9222 0.9405 0.9325 0.9356 -0.9220 -0.8415 0.9415 0.9302 -0.9095 0.9455 -0.8668 0.9168 0.8138 0.9844 0.9790 0.9656 0.9164 0.9760 0.9717 0.9198 0.9604 |
| Root TFAADs/N | ##### | 0.9781 | -0.9748 | -0.8556 | -0.8774 | 0.7349 | 0.9365 | -0.9671 | ##### | 0.9978 | 0.9436 | 0.6086 | 0.9673 | 0.8117 | 0.8251 | 0.9962 | 0.9911 | -0.4127 | 0.8127 | 0.9547 | 0.9897 | 0.8898 | 0.9468 | 0.8341 | 0.9819 | 0.8948 | 0.9921 | 0.9170 | -0.5113 | 0.9258 | 0.9924 | 0.9237 | -0.9614 | 0.9791 | -0.8549 | 0.8272 | 0.9971 | 0.8787 | -0.5395 | 0.9424 | 0.9201 | 0.9722 | -0.9730 | 0.9839 | 0.9921 | 0.8219 | 0.9770 | -0.5476 | 0.8737 | 0.8020 | 0.8223 | 0.9345 | 0.3959 | 0.9033 | 0.9450 | 0.8002 | 0.9057 | -0.8853 | 0.8244 | 0.9451 | -0.9801 | 0.8456 0.9862 0.9728 -0.5860 0.5556 0.8828 0.4268 0.9682 0.9379 0.7470 0.2703 0.6840 0.9277 0.9844 0.9896 1.0000 -0.9285 0.9969 0.8474 0.9444 0.9553 0.8983 0.5198 0.9400 0.9592 0.9606 0.9563 -0.9489 -0.8846 0.9571 0.9523 -0.9423 0.9192 -0.9091 0.8943 0.8531 0.9767 0.9792 0.9307 0.9369 0.9366 0.9305 0.9176 0.9682 |
| Root molar ratio of C/N in TFAADs | ##### | ###### | 0.8858 | 0.6604 | 0.7166 | -0.9130 | -0.9666 | 0.8558 | ##### | -0.9475 | -0.8224 | -0.3283 | -0.8405 | -0.6233 | -0.5908 | -0.9453 | -0.9122 | 0.5671 | -0.9564 | -0.7974 | -0.9234 | -0.8190 | -0.9974 | -0.9692 | -0.9747 | -0.9931 | -0.9080 | -0.9988 | 0.2115 | -0.8955 | -0.9001 | -0.9937 | 0.9564 | -0.8349 | 0.6697 | -0.5927 | -0.9282 | -0.9922 | 0.2670 | -0.9457 | -0.7504 | -0.9117 | 0.9837 | -0.8901 | -0.9653 | -0.5789 | -0.8406 | 0.5901 | -0.7110 | -0.6503 | -0.9718 | -0.8891 | -0.0594 | -0.9975 | -0.8037 | -0.5288 | -0.7540 | 0.7181 | -0.5901 | -0.9832 | 0.8985 | -0.6128 -0.8671 -0.8263 0.8439 -0.2572 -0.7946 -0.0605 -0.9712 -0.9856 -0.4876 -0.0632 -0.8047 -0.7436 -0.9076 -0.9698 -0.9285 1.0000 -0.9480 -0.9310 -0.8255 -0.9768 -0.9655 -0.2590 -0.8444 -0.8626 -0.8436 -0.8551 0.8243 0.6991 -0.8567 -0.8524 0.8044 -0.9757 0.7400 -0.8877 -0.6941 -0.9506 -0.9169 -0.9891 -0.8158 -0.9895 -0.9978 -0.8604 -0.8876 |
| Root TFAADs/C | ##### | 0.9910 | -0.9775 | -0.8395 | -0.8706 | 0.7792 | 0.9607 | -0.9684 | ##### | 0.9973 | 0.9201 | 0.5809 | 0.9452 | 0.7936 | 0.7961 | 0.9990 | 0.9881 | -0.4245 | 0.8420 | 0.9358 | 0.9943 | 0.9086 | 0.9647 | 0.8689 | 0.9867 | 0.9143 | 0.9857 | 0.9400 | -0.4641 | 0.9170 | 0.9850 | 0.9469 | -0.9744 | 0.9659 | -0.8453 | 0.8049 | 0.9963 | 0.9027 | -0.5158 | 0.9573 | 0.8955 | 0.9625 | -0.9797 | 0.9854 | 0.9967 | 0.7955 | 0.9582 | -0.5994 | 0.8369 | 0.7980 | 0.8488 | 0.9520 | 0.3468 | 0.9262 | 0.9173 | 0.7549 | 0.9014 | -0.8793 | 0.8060 | 0.9580 | -0.9834 | 0.8261 0.9774 0.9613 -0.6359 0.5296 0.8980 0.3720 0.9837 0.9460 0.7145 0.2175 0.7273 0.9125 0.9877 0.9966 0.9969 -0.9480 1.0000 0.8846 0.9470 0.9715 0.9254 0.5047 0.9442 0.9609 0.9566 0.9573 -0.9480 -0.8785 0.9616 0.9522 -0.9385 0.9270 -0.9028 0.9206 0.8524 0.9874 0.9875 0.9426 0.9407 0.9600 0.9523 0.9341 0.9752 |
| Root NR | ##### | 0.9387 | -0.8832 | -0.6897 | -0.7679 | 0.9549 | 0.9621 | -0.8562 | ##### | 0.8664 | 0.6918 | 0.4115 | 0.7005 | 0.6564 | 0.5753 | 0.8887 | 0.8593 | -0.5354 | 0.8622 | 0.7318 | 0.8942 | 0.8933 | 0.9320 | 0.9305 | 0.8927 | 0.8951 | 0.8371 | 0.9434 | -0.2114 | 0.7861 | 0.8290 | 0.9399 | -0.9461 | 0.7658 | -0.7265 | 0.6124 | 0.8698 | 0.9118 | -0.3865 | 0.8773 | 0.6769 | 0.7542 | -0.8937 | 0.8641 | 0.8845 | 0.5813 | 0.7168 | -0.8183 | 0.4892 | 0.7422 | 0.8841 | 0.9117 | 0.0857 | 0.9362 | 0.6430 | 0.4019 | 0.7969 | -0.7694 | 0.6261 | 0.8788 | -0.8887 | 0.6309 0.8127 0.7799 -0.8286 0.3598 0.8440 0.0180 0.9291 0.8586 0.4927 -0.0049 0.8093 0.7291 0.8903 0.8997 0.8474 -0.9310 0.8846 1.0000 0.8545 0.9118 0.9263 0.4171 0.8796 0.8746 0.8391 0.8683 -0.8430 -0.7328 0.8779 0.8635 -0.8211 0.8642 -0.7728 0.9692 0.7794 0.9354 0.9042 0.8692 0.8501 0.9639 0.9521 0.9302 0.8979 |
| Root GOGAT | ##### | 0.9396 | -0.9908 | -0.9413 | -0.9671 | 0.6816 | 0.9052 | -0.9960 | ##### | 0.9331 | 0.8655 | 0.7776 | 0.8852 | 0.8969 | 0.8901 | 0.9521 | 0.9606 | -0.3065 | 0.6637 | 0.9457 | 0.9752 | 0.9663 | 0.8556 | 0.7337 | 0.8986 | 0.7576 | 0.9488 | 0.8213 | -0.6191 | 0.8271 | 0.9524 | 0.8351 | -0.9361 | 0.9636 | -0.9600 | 0.9226 | 0.9585 | 0.7502 | -0.7362 | 0.8710 | 0.9091 | 0.8508 | -0.8760 | 0.9839 | 0.9191 | 0.9063 | 0.9135 | -0.7117 | 0.7424 | 0.9214 | 0.6734 | 0.9573 | 0.5441 | 0.7974 | 0.8520 | 0.7915 | 0.9852 | -0.9784 | 0.9342 | 0.8280 | -0.9873 | 0.9401 0.9716 0.9766 -0.4574 0.7508 0.9474 0.5342 0.9162 0.7964 0.8403 0.2844 0.6325 0.9735 0.9834 0.9226 0.9444 -0.8255 0.9470 0.8545 1.0000 0.8778 0.8251 0.7452 0.9896 0.9900 0.9857 0.9898 -0.9974 -0.9738 0.9983 0.9830 -0.9945 0.7853 -0.9884 0.9481 0.9716 0.9565 0.9775 0.7917 0.9984 0.8776 0.8484 0.9817 0.9925 |
| Root GOT | ##### | 0.9793 | -0.9169 | -0.6976 | -0.7497 | 0.8404 | 0.9862 | -0.9096 | ##### | 0.9611 | 0.8191 | 0.3928 | 0.8630 | 0.6356 | 0.6334 | 0.9629 | 0.9255 | -0.3847 | 0.9362 | 0.8263 | 0.9561 | 0.8901 | 0.9891 | 0.9522 | 0.9647 | 0.9593 | 0.9201 | 0.9761 | -0.2424 | 0.8462 | 0.9171 | 0.9895 | -0.9440 | 0.8888 | -0.7175 | 0.6559 | 0.9479 | 0.9552 | -0.3261 | 0.9929 | 0.7656 | 0.9432 | -0.9691 | 0.9402 | 0.9831 | 0.6397 | 0.8886 | -0.6471 | 0.7629 | 0.6633 | 0.9115 | 0.9511 | 0.1251 | 0.9610 | 0.8271 | 0.6083 | 0.7960 | -0.7666 | 0.6698 | 0.9920 | -0.9282 | 0.6950 0.9011 0.8767 -0.7636 0.3536 0.8888 0.1889 0.9957 0.9624 0.5335 -0.0034 0.8599 0.8014 0.9359 0.9853 0.9553 -0.9768 0.9715 0.9118 0.8778 1.0000 0.9874 0.3354 0.8670 0.8863 0.8692 0.8790 -0.8670 -0.7935 0.9006 0.8684 -0.8481 0.9244 -0.8030 0.9085 0.7444 0.9565 0.9591 0.9737 0.8781 0.9852 0.9778 0.8914 0.9280 |
| Root GPT | ##### | 0.9499 | -0.8636 | -0.6049 | -0.6729 | 0.8732 | 0.9834 | -0.8574 | ##### | 0.9076 | 0.7205 | 0.2867 | 0.7724 | 0.5358 | 0.5208 | 0.9140 | 0.8621 | -0.3545 | 0.9601 | 0.7335 | 0.9105 | 0.8755 | 0.9752 | 0.9795 | 0.9204 | 0.9530 | 0.8516 | 0.9718 | -0.1043 | 0.7708 | 0.8471 | 0.9885 | -0.9083 | 0.8129 | -0.6378 | 0.5570 | 0.8914 | 0.9570 | -0.2270 | 0.9833 | 0.6606 | 0.8854 | -0.9302 | 0.8908 | 0.9428 | 0.5339 | 0.8072 | -0.7054 | 0.6627 | 0.5880 | 0.9194 | 0.9367 | -0.0073 | 0.9556 | 0.7283 | 0.4838 | 0.7259 | -0.6940 | 0.5808 | 0.9760 | -0.8767 | 0.6053 0.8289 0.8008 -0.8271 0.2579 0.8744 0.0637 0.9775 0.9332 0.4153 -0.1464 0.9240 0.7166 0.8850 0.9484 0.8983 -0.9655 0.9254 0.9263 0.8251 0.9874 1.0000 0.2551 0.8119 0.8280 0.8004 0.8190 -0.8059 -0.7322 0.8513 0.8050 -0.7812 0.8882 -0.7336 0.9004 0.6813 0.9188 0.9221 0.9541 0.8301 0.9792 0.9688 0.8626 0.8847 |
| Root GS | ##### | 0.4820 | -0.6687 | -0.8701 | -0.8520 | 0.1718 | 0.4124 | -0.6922 | ##### | 0.4885 | 0.5398 | 0.9745 | 0.5220 | 0.8645 | 0.8537 | 0.5257 | 0.5911 | -0.0375 | 0.0035 | 0.6954 | 0.5842 | 0.6733 | 0.3027 | 0.1221 | 0.4141 | 0.1482 | 0.5735 | 0.2536 | -0.8579 | 0.4260 | 0.5851 | 0.2653 | -0.5210 | 0.6501 | -0.8842 | 0.8932 | 0.5578 | 0.1411 | -0.9879 | 0.3355 | 0.7032 | 0.3499 | -0.3638 | 0.6251 | 0.4335 | 0.8813 | 0.5480 | -0.5286 | 0.3790 | 0.8812 | 0.0487 | 0.5680 | 0.8799 | 0.2253 | 0.5044 | 0.6930 | 0.8255 | -0.8536 | 0.9014 | 0.2447 | -0.6459 | 0.8774 0.6475 0.6931 0.1596 0.9832 0.6402 0.7789 0.4169 0.2160 0.8949 0.5248 0.0694 0.7920 0.6272 0.4380 0.5198 -0.2590 0.5047 0.4171 0.7452 0.3354 0.2551 1.0000 0.7356 0.7082 0.7211 0.7177 -0.7518 -0.8146 0.7094 0.7173 -0.7712 0.2424 -0.8246 0.6071 0.8745 0.5454 0.5881 0.1942 0.7441 0.3548 0.3043 0.6937 0.6601 |
| Root glucose | ##### | 0.9451 | -0.9913 | -0.9491 | -0.9767 | 0.7337 | 0.9012 | -0.9815 | ##### | 0.9366 | 0.8843 | 0.7742 | 0.8843 | 0.9228 | 0.8936 | 0.9546 | 0.9697 | -0.4340 | 0.6688 | 0.9494 | 0.9700 | 0.9394 | 0.8659 | 0.7423 | 0.9153 | 0.7788 | 0.9577 | 0.8397 | -0.6349 | 0.8732 | 0.9584 | 0.8408 | -0.9603 | 0.9467 | -0.9609 | 0.9133 | 0.9612 | 0.7735 | -0.7381 | 0.8432 | 0.9234 | 0.8346 | -0.8936 | 0.9684 | 0.9168 | 0.8982 | 0.8956 | -0.6916 | 0.7142 | 0.9477 | 0.7093 | 0.9310 | 0.5374 | 0.8243 | 0.8498 | 0.7606 | 0.9868 | -0.9770 | 0.9136 | 0.8231 | -0.9875 | 0.9161 0.9687 0.9646 -0.4998 0.7256 0.9024 0.4772 0.9086 0.8129 0.8445 0.3557 0.5884 0.9617 0.9836 0.9222 0.9400 -0.8444 0.9442 0.8796 0.9896 0.8670 0.8119 0.7356 1.0000 0.9980 0.9930 0.9982 -0.9941 -0.9375 0.9919 0.9971 -0.9916 0.8235 -0.9749 0.9538 0.9704 0.9686 0.9659 0.8016 0.9809 0.8877 0.8673 0.9843 0.9859 |
| Root fructose | ##### | 0.9566 | -0.9968 | -0.9450 | -0.9700 | 0.7322 | 0.9102 | -0.9872 | ##### | 0.9556 | 0.9076 | 0.7584 | 0.9109 | 0.9163 | 0.8959 | 0.9697 | 0.9828 | -0.4341 | 0.6934 | 0.9627 | 0.9809 | 0.9349 | 0.8839 | 0.7575 | 0.9341 | 0.8019 | 0.9736 | 0.8560 | -0.6295 | 0.8927 | 0.9744 | 0.8577 | -0.9668 | 0.9632 | -0.9533 | 0.9112 | 0.9761 | 0.7938 | -0.7157 | 0.8641 | 0.9364 | 0.8673 | -0.9140 | 0.9791 | 0.9368 | 0.8985 | 0.9211 | -0.6620 | 0.7550 | 0.9325 | 0.7301 | 0.9358 | 0.5281 | 0.8409 | 0.8794 | 0.7831 | 0.9826 | -0.9712 | 0.9100 | 0.8485 | -0.9943 | 0.9159 0.9821 0.9768 -0.5093 0.7080 0.9031 0.4832 0.9235 0.8402 0.8422 0.3565 0.6002 0.9674 0.9918 0.9405 0.9592 -0.8626 0.9609 0.8746 0.9900 0.8863 0.8280 0.7082 0.9980 1.0000 0.9973 0.9999 -0.9954 -0.9374 0.9938 0.9986 -0.9927 0.8463 -0.9734 0.9467 0.9591 0.9766 0.9748 0.8286 0.9810 0.8993 0.8816 0.9782 0.9901 |
| Root sucrose | ##### | 0.9431 | -0.9928 | -0.9593 | -0.9754 | 0.6913 | 0.8857 | -0.9840 | ##### | 0.9549 | 0.9294 | 0.7835 | 0.9306 | 0.9333 | 0.9223 | 0.9659 | 0.9847 | -0.4278 | 0.6647 | 0.9795 | 0.9749 | 0.9145 | 0.8660 | 0.7236 | 0.9289 | 0.7831 | 0.9786 | 0.8337 | -0.6741 | 0.9018 | 0.9803 | 0.8347 | -0.9541 | 0.9736 | -0.9611 | 0.9308 | 0.9756 | 0.7708 | -0.7374 | 0.8475 | 0.9591 | 0.8746 | -0.9071 | 0.9756 | 0.9309 | 0.9220 | 0.9369 | -0.6110 | 0.7878 | 0.9361 | 0.7055 | 0.9167 | 0.5738 | 0.8192 | 0.9044 | 0.8237 | 0.9854 | -0.9755 | 0.9253 | 0.8348 | -0.9898 | 0.9321 0.9890 0.9859 -0.4629 0.7306 0.8854 0.5322 0.9071 0.8320 0.8721 0.4094 0.5562 0.9800 0.9873 0.9325 0.9606 -0.8436 0.9566 0.8391 0.9857 0.8692 0.8004 0.7211 0.9930 0.9973 1.0000 0.9979 -0.9947 -0.9402 0.9878 0.9979 -0.9948 0.8392 -0.9774 0.9206 0.9584 0.9656 0.9650 0.8165 0.9752 0.8763 0.8604 0.9612 0.9819 |
| Root total soluble sugars | ##### | 0.9519 | -0.9956 | -0.9501 | -0.9736 | 0.7237 | 0.9035 | -0.9862 | ##### | 0.9523 | 0.9085 | 0.7683 | 0.9107 | 0.9225 | 0.9024 | 0.9665 | 0.9813 | -0.4330 | 0.6821 | 0.9647 | 0.9781 | 0.9320 | 0.8767 | 0.7471 | 0.9298 | 0.7933 | 0.9723 | 0.8482 | -0.6415 | 0.8915 | 0.9733 | 0.8496 | -0.9634 | 0.9630 | -0.9577 | 0.9170 | 0.9737 | 0.7849 | -0.7262 | 0.8566 | 0.9397 | 0.8628 | -0.9089 | 0.9769 | 0.9321 | 0.9047 | 0.9202 | -0.6571 | 0.7546 | 0.9375 | 0.7207 | 0.9313 | 0.5411 | 0.8331 | 0.8797 | 0.7884 | 0.9851 | -0.9744 | 0.9151 | 0.8407 | -0.9928 | 0.9205 0.9818 0.9772 -0.4971 0.7176 0.8997 0.4937 0.9174 0.8332 0.8504 0.3689 0.5881 0.9700 0.9899 0.9356 0.9563 -0.8551 0.9573 0.8683 0.9898 0.8790 0.8190 0.7177 0.9982 0.9999 0.9979 1.0000 -0.9959 -0.9389 0.9929 0.9990 -0.9939 0.8406 -0.9755 0.9431 0.9622 0.9733 0.9716 0.8208 0.9806 0.8924 0.8745 0.9765 0.9882 |
| Root starch | ##### | ###### | 0.9928 | 0.9576 | 0.9780 | -0.6760 | -0.8915 | 0.9929 | ##### | -0.9390 | -0.8925 | -0.7956 | -0.9036 | -0.9215 | -0.9132 | -0.9554 | -0.9701 | 0.3510 | -0.6505 | -0.9633 | -0.9739 | -0.9462 | -0.8520 | -0.7179 | -0.9062 | -0.7573 | -0.9605 | -0.8175 | 0.6576 | -0.8552 | -0.9636 | -0.8261 | 0.9408 | -0.9695 | 0.9692 | -0.9362 | -0.9641 | -0.7477 | 0.7533 | -0.8543 | -0.9344 | -0.8553 | 0.8828 | -0.9806 | -0.9194 | -0.9231 | -0.9230 | 0.6707 | -0.7598 | -0.9365 | -0.6742 | -0.9389 | -0.5743 | -0.7971 | -0.8736 | -0.8123 | -0.9915 | 0.9845 | -0.9406 | -0.8219 | 0.9889 | -0.9461 -0.9801 -0.9832 0.4442 -0.7588 -0.9227 -0.5489 -0.9070 -0.8020 -0.8656 -0.3481 -0.5896 -0.9821 -0.9850 -0.9220 -0.9489 0.8243 -0.9480 -0.8430 -0.9974 -0.8670 -0.8059 -0.7518 -0.9941 -0.9954 -0.9947 -0.9959 1.0000 0.9663 -0.9962 -0.9920 0.9991 -0.7994 0.9902 -0.9360 -0.9740 -0.9580 -0.9712 -0.7922 -0.9922 -0.8701 -0.8455 -0.9748 -0.9888 |
| Root TNC | ##### | ###### | 0.9397 | 0.9353 | 0.9462 | -0.5096 | -0.8168 | 0.9669 | ##### | -0.8587 | -0.8062 | -0.8372 | -0.8424 | -0.8768 | -0.9026 | -0.8803 | -0.8942 | 0.1019 | -0.5309 | -0.9156 | -0.9177 | -0.9435 | -0.7431 | -0.6023 | -0.7980 | -0.6205 | -0.8833 | -0.6932 | 0.6732 | -0.7132 | -0.8925 | -0.7219 | 0.8340 | -0.9439 | 0.9579 | -0.9480 | -0.8930 | -0.6080 | 0.7962 | -0.8090 | -0.8769 | -0.7951 | 0.7684 | -0.9463 | -0.8415 | -0.9333 | -0.8891 | 0.6787 | -0.7401 | -0.8913 | -0.5113 | -0.9179 | -0.6425 | -0.6582 | -0.8164 | -0.8436 | -0.9649 | 0.9687 | -0.9704 | -0.7313 | 0.9328 | -0.9758 -0.9308 -0.9555 0.2737 -0.8415 -0.9460 -0.6808 -0.8361 -0.6769 -0.8695 -0.2731 -0.5670 -0.9718 -0.9263 -0.8415 -0.8846 0.6991 -0.8785 -0.7328 -0.9738 -0.7935 -0.7322 -0.8146 -0.9375 -0.9374 -0.9402 -0.9389 0.9663 1.0000 -0.9601 -0.9269 0.9683 -0.6462 0.9868 -0.8734 -0.9633 -0.8710 -0.9259 -0.6749 -0.9801 -0.7673 -0.7246 -0.9258 -0.9456 |
| Root sucrose/starch | ##### | 0.9577 | -0.9963 | -0.9291 | -0.9587 | 0.7192 | 0.9256 | -0.9972 | ##### | 0.9491 | 0.8752 | 0.7457 | 0.8937 | 0.8859 | 0.8746 | 0.9665 | 0.9714 | -0.3448 | 0.7031 | 0.9458 | 0.9851 | 0.9661 | 0.8836 | 0.7692 | 0.9209 | 0.7937 | 0.9599 | 0.8529 | -0.5893 | 0.8491 | 0.9623 | 0.8643 | -0.9545 | 0.9647 | -0.9470 | 0.9041 | 0.9706 | 0.7871 | -0.7023 | 0.8902 | 0.9081 | 0.8683 | -0.9012 | 0.9892 | 0.9377 | 0.8878 | 0.9188 | -0.7117 | 0.7476 | 0.9115 | 0.7154 | 0.9645 | 0.5043 | 0.8313 | 0.8594 | 0.7751 | 0.9784 | -0.9685 | 0.9140 | 0.8557 | -0.9942 | 0.9212 0.9762 0.9762 -0.5058 0.7129 0.9451 0.4917 0.9356 0.8280 0.8190 0.2706 0.6572 0.9654 0.9915 0.9415 0.9571 -0.8567 0.9616 0.8779 0.9983 0.9006 0.8513 0.7094 0.9919 0.9938 0.9878 0.9929 -0.9962 -0.9601 1.0000 0.9867 -0.9915 0.8183 -0.9790 0.9584 0.9602 0.9719 0.9864 0.8237 0.9955 0.9033 0.8777 0.9861 0.9977 |
| Root malate | ##### | 0.9469 | -0.9918 | -0.9537 | -0.9753 | 0.7253 | 0.8930 | -0.9783 | ##### | 0.9499 | 0.9166 | 0.7725 | 0.9125 | 0.9320 | 0.9080 | 0.9631 | 0.9813 | -0.4660 | 0.6732 | 0.9672 | 0.9720 | 0.9165 | 0.8718 | 0.7378 | 0.9300 | 0.7914 | 0.9730 | 0.8445 | -0.6565 | 0.9039 | 0.9734 | 0.8421 | -0.9639 | 0.9580 | -0.9576 | 0.9173 | 0.9712 | 0.7825 | -0.7312 | 0.8413 | 0.9469 | 0.8569 | -0.9089 | 0.9686 | 0.9267 | 0.9063 | 0.9162 | -0.6367 | 0.7523 | 0.9437 | 0.7215 | 0.9160 | 0.5505 | 0.8320 | 0.8826 | 0.7879 | 0.9837 | -0.9726 | 0.9111 | 0.8325 | -0.9887 | 0.9157 0.9797 0.9732 -0.4948 0.7152 0.8797 0.4894 0.9078 0.8326 0.8574 0.4023 0.5618 0.9673 0.9858 0.9302 0.9523 -0.8524 0.9522 0.8635 0.9830 0.8684 0.8050 0.7173 0.9971 0.9986 0.9979 0.9990 -0.9920 -0.9269 0.9867 1.0000 -0.9909 0.8464 -0.9702 0.9352 0.9594 0.9708 0.9627 0.8173 0.9714 0.8862 0.8712 0.9701 0.9813 |
| Root citrate | ##### | ###### | 0.9880 | 0.9687 | 0.9842 | -0.6490 | -0.8716 | 0.9885 | ##### | -0.9311 | -0.8979 | -0.8186 | -0.9063 | -0.9353 | -0.9300 | -0.9469 | -0.9663 | 0.3463 | -0.6217 | -0.9693 | -0.9656 | -0.9344 | -0.8330 | -0.6893 | -0.8953 | -0.7356 | -0.9576 | -0.7962 | 0.6895 | -0.8535 | -0.9613 | -0.8040 | 0.9297 | -0.9701 | 0.9773 | -0.9501 | -0.9579 | -0.7243 | 0.7765 | -0.8352 | -0.9445 | -0.8475 | 0.8701 | -0.9741 | -0.9076 | -0.9387 | -0.9237 | 0.6481 | -0.7659 | -0.9448 | -0.6494 | -0.9243 | -0.6086 | -0.7759 | -0.8788 | -0.8309 | -0.9950 | 0.9896 | -0.9524 | -0.8028 | 0.9831 | -0.9571 -0.9794 -0.9845 0.4092 -0.7810 -0.9107 -0.5795 -0.8902 -0.7858 -0.8865 -0.3829 -0.5557 -0.9883 -0.9788 -0.9095 -0.9423 0.8044 -0.9385 -0.8211 -0.9945 -0.8481 -0.7812 -0.7712 -0.9916 -0.9927 -0.9948 -0.9939 0.9991 0.9683 -0.9915 -0.9909 1.0000 -0.7857 0.9935 -0.9212 -0.9779 -0.9474 -0.9612 -0.7736 -0.9883 -0.8501 -0.8256 -0.9658 -0.9817 |
| Root isocitrate | ##### | 0.9350 | -0.8611 | -0.6758 | -0.7137 | 0.8695 | 0.8992 | -0.8167 | ##### | 0.9424 | 0.8838 | 0.3499 | 0.8783 | 0.6629 | 0.6316 | 0.9299 | 0.9141 | -0.6832 | 0.9050 | 0.8244 | 0.8924 | 0.7260 | 0.9654 | 0.9034 | 0.9751 | 0.9761 | 0.9181 | 0.9660 | -0.3033 | 0.9582 | 0.9089 | 0.9460 | -0.9408 | 0.8289 | -0.6618 | 0.6023 | 0.9196 | 0.9673 | -0.2838 | 0.8776 | 0.8014 | 0.9125 | -0.9829 | 0.8528 | 0.9444 | 0.5997 | 0.8511 | -0.4310 | 0.7585 | 0.6569 | 0.9588 | 0.8053 | 0.1285 | 0.9755 | 0.8536 | 0.5768 | 0.7383 | -0.7014 | 0.5779 | 0.9488 | -0.8733 | 0.6008 0.8663 0.8196 -0.7905 0.2501 0.6915 0.0929 0.9200 0.9871 0.5343 0.2415 0.6624 0.7402 0.8834 0.9455 0.9192 -0.9757 0.9270 0.8642 0.7853 0.9244 0.8882 0.2424 0.8235 0.8463 0.8392 0.8406 -0.7994 -0.6462 0.8183 0.8464 -0.7857 1.0000 -0.7124 0.8162 0.6640 0.9261 0.8700 0.9756 0.7647 0.9423 0.9660 0.8042 0.8458 |
| Root malate + citrate + isocitrate | ##### | ###### | 0.9673 | 0.9744 | 0.9847 | -0.5742 | -0.8309 | 0.9780 | ##### | -0.8912 | -0.8621 | -0.8607 | -0.8763 | -0.9356 | -0.9402 | -0.9106 | -0.9337 | 0.2529 | -0.5495 | -0.9534 | -0.9386 | -0.9326 | -0.7754 | -0.6237 | -0.8430 | -0.6629 | -0.9238 | -0.7321 | 0.7219 | -0.7942 | -0.9300 | -0.7466 | 0.8843 | -0.9574 | 0.9870 | -0.9691 | -0.9247 | -0.6508 | 0.8225 | -0.7995 | -0.9276 | -0.8076 | 0.8134 | -0.9565 | -0.8655 | -0.9570 | -0.9031 | 0.6548 | -0.7454 | -0.9457 | -0.5659 | -0.9092 | -0.6635 | -0.7063 | -0.8502 | -0.8470 | -0.9944 | 0.9948 | -0.9774 | -0.7480 | 0.9600 | -0.9800 -0.9587 -0.9737 0.3209 -0.8385 -0.9157 -0.6477 -0.8499 -0.7191 -0.9069 -0.3790 -0.5222 -0.9904 -0.9537 -0.8668 -0.9091 0.7400 -0.9028 -0.7728 -0.9884 -0.8030 -0.7336 -0.8246 -0.9749 -0.9734 -0.9774 -0.9755 0.9902 0.9868 -0.9790 -0.9702 0.9935 -0.7124 1.0000 -0.8967 -0.9870 -0.9090 -0.9381 -0.7083 -0.9861 -0.7975 -0.7649 -0.9496 -0.9637 |
| Root NADP-ME | ##### | 0.9542 | -0.9511 | -0.8203 | -0.8819 | 0.8538 | 0.9613 | -0.9424 | ##### | 0.8983 | 0.7508 | 0.5999 | 0.7681 | 0.7771 | 0.7221 | 0.9253 | 0.9106 | -0.4059 | 0.7753 | 0.8277 | 0.9467 | 0.9708 | 0.9048 | 0.8571 | 0.8948 | 0.8301 | 0.8884 | 0.8963 | -0.3895 | 0.7866 | 0.8860 | 0.9043 | -0.9560 | 0.8605 | -0.8581 | 0.7694 | 0.9165 | 0.8400 | -0.5735 | 0.8893 | 0.7747 | 0.7854 | -0.8836 | 0.9364 | 0.9046 | 0.7399 | 0.7984 | -0.8473 | 0.5714 | 0.8509 | 0.7850 | 0.9664 | 0.2948 | 0.8780 | 0.7156 | 0.5627 | 0.9073 | -0.8909 | 0.7893 | 0.8572 | -0.9514 | 0.7917 0.8910 0.8781 -0.6722 0.5681 0.9334 0.2501 0.9395 0.8205 0.6561 0.0858 0.7658 0.8537 0.9490 0.9168 0.8943 -0.8877 0.9206 0.9692 0.9481 0.9085 0.9004 0.6071 0.9538 0.9467 0.9206 0.9431 -0.9360 -0.8734 0.9584 0.9352 -0.9212 0.8162 -0.8967 1.0000 0.9011 0.9609 0.9589 0.8282 0.9480 0.9417 0.9152 0.9899 0.9653 |
| Root NAD-ME | ##### | 0.8461 | -0.9429 | -0.9754 | -0.9914 | 0.5839 | 0.7968 | -0.9479 | ##### | 0.8383 | 0.8069 | 0.8869 | 0.8066 | 0.9490 | 0.9268 | 0.8656 | 0.8956 | -0.2907 | 0.4861 | 0.9140 | 0.9000 | 0.9207 | 0.7267 | 0.5813 | 0.7949 | 0.6076 | 0.8798 | 0.6903 | -0.7344 | 0.7563 | 0.8848 | 0.6988 | -0.8655 | 0.9039 | -0.9930 | 0.9607 | 0.8804 | 0.6023 | -0.8658 | 0.7318 | 0.8933 | 0.7190 | -0.7621 | 0.9152 | 0.8081 | 0.9440 | 0.8298 | -0.7027 | 0.6375 | 0.9774 | 0.5213 | 0.8769 | 0.6784 | 0.6669 | 0.7742 | 0.7793 | 0.9923 | -0.9955 | 0.9684 | 0.6783 | -0.9323 | 0.9622 0.9162 0.9310 -0.3049 0.8621 0.8865 0.6119 0.8017 0.6532 0.9050 0.4004 0.4708 0.9626 0.9229 0.8138 0.8531 -0.6941 0.8524 0.7794 0.9716 0.7444 0.6813 0.8745 0.9704 0.9591 0.9584 0.9622 -0.9740 -0.9633 0.9602 0.9594 -0.9779 0.6640 -0.9870 0.9011 1.0000 0.8826 0.9016 0.6407 0.9675 0.7626 0.7272 0.9509 0.9394 |
| Root NADP-MDH | ##### | 0.9957 | -0.9846 | -0.8551 | -0.8962 | 0.8396 | 0.9695 | -0.9668 | ##### | 0.9825 | 0.8941 | 0.6009 | 0.9049 | 0.8206 | 0.7924 | 0.9915 | 0.9843 | -0.4993 | 0.8303 | 0.9208 | 0.9907 | 0.9249 | 0.9628 | 0.8773 | 0.9810 | 0.9096 | 0.9758 | 0.9471 | -0.4658 | 0.9175 | 0.9730 | 0.9468 | -0.9963 | 0.9389 | -0.8664 | 0.8054 | 0.9876 | 0.9054 | -0.5502 | 0.9304 | 0.8831 | 0.9107 | -0.9725 | 0.9756 | 0.9797 | 0.7906 | 0.9131 | -0.6709 | 0.7514 | 0.8460 | 0.8573 | 0.9534 | 0.3412 | 0.9366 | 0.8686 | 0.6930 | 0.9204 | -0.8984 | 0.8053 | 0.9341 | -0.9884 | 0.8180 0.9645 0.9443 -0.6739 0.5419 0.8948 0.3140 0.9773 0.9273 0.7159 0.2302 0.7171 0.9039 0.9905 0.9844 0.9767 -0.9506 0.9874 0.9354 0.9565 0.9565 0.9188 0.5454 0.9686 0.9766 0.9656 0.9733 -0.9580 -0.8710 0.9719 0.9708 -0.9474 0.9261 -0.9090 0.9609 0.8826 1.0000 0.9856 0.9229 0.9476 0.9710 0.9624 0.9668 0.9828 |
| Root NAD-MDH | ##### | 0.9859 | -0.9891 | -0.8635 | -0.9012 | 0.7697 | 0.9685 | -0.9894 | ##### | 0.9748 | 0.8727 | 0.6330 | 0.9043 | 0.8094 | 0.8057 | 0.9867 | 0.9751 | -0.3463 | 0.8037 | 0.9234 | 0.9973 | 0.9628 | 0.9405 | 0.8516 | 0.9554 | 0.8691 | 0.9659 | 0.9140 | -0.4735 | 0.8606 | 0.9669 | 0.9285 | -0.9676 | 0.9619 | -0.8832 | 0.8348 | 0.9839 | 0.8627 | -0.5787 | 0.9525 | 0.8749 | 0.9194 | -0.9439 | 0.9959 | 0.9762 | 0.8182 | 0.9327 | -0.7035 | 0.7775 | 0.8354 | 0.7976 | 0.9846 | 0.3782 | 0.8930 | 0.8691 | 0.7384 | 0.9325 | -0.9157 | 0.8480 | 0.9262 | -0.9920 | 0.8628 0.9714 0.9640 -0.6037 0.6005 0.9519 0.4024 0.9797 0.8941 0.7323 0.1662 0.7524 0.9290 0.9931 0.9790 0.9792 -0.9169 0.9875 0.9042 0.9775 0.9591 0.9221 0.5881 0.9659 0.9748 0.9650 0.9716 -0.9712 -0.9259 0.9864 0.9627 -0.9612 0.8700 -0.9381 0.9589 0.9016 0.9856 1.0000 0.8963 0.9769 0.9514 0.9307 0.9716 0.9950 |
| Root PEPC | ##### | 0.9481 | -0.8588 | -0.6260 | -0.6719 | 0.8542 | 0.9394 | -0.8329 | ##### | 0.9469 | 0.8393 | 0.2874 | 0.8661 | 0.5834 | 0.5764 | 0.9360 | 0.9005 | -0.5240 | 0.9605 | 0.7954 | 0.9067 | 0.7737 | 0.9877 | 0.9512 | 0.9698 | 0.9926 | 0.9031 | 0.9831 | -0.1994 | 0.8945 | 0.8964 | 0.9812 | -0.9232 | 0.8391 | -0.6269 | 0.5689 | 0.9200 | 0.9846 | -0.2129 | 0.9498 | 0.7496 | 0.9463 | -0.9813 | 0.8763 | 0.9657 | 0.5616 | 0.8651 | -0.4931 | 0.7789 | 0.5877 | 0.9639 | 0.8607 | 0.0487 | 0.9815 | 0.8385 | 0.5676 | 0.7137 | -0.6766 | 0.5637 | 0.9929 | -0.8738 | 0.5933 0.8607 0.8209 -0.8116 0.2176 0.7645 0.0901 0.9589 0.9973 0.4684 0.0648 0.7913 0.7287 0.8854 0.9656 0.9307 -0.9891 0.9426 0.8692 0.7917 0.9737 0.9541 0.1942 0.8016 0.8286 0.8165 0.8208 -0.7922 -0.6749 0.8237 0.8173 -0.7736 0.9756 -0.7083 0.8282 0.6407 0.9229 0.8963 1.0000 0.7825 0.9653 0.9783 0.8068 0.8578 |
| Root PEPP | ##### | 0.9335 | -0.9845 | -0.9288 | -0.9566 | 0.6694 | 0.9063 | -0.9950 | ##### | 0.9233 | 0.8436 | 0.7690 | 0.8706 | 0.8776 | 0.8753 | 0.9440 | 0.9487 | -0.2589 | 0.6620 | 0.9317 | 0.9708 | 0.9762 | 0.8487 | 0.7330 | 0.8853 | 0.7468 | 0.9357 | 0.8130 | -0.5976 | 0.7994 | 0.9401 | 0.8316 | -0.9236 | 0.9584 | -0.9523 | 0.9149 | 0.9493 | 0.7402 | -0.7277 | 0.8773 | 0.8898 | 0.8447 | -0.8629 | 0.9827 | 0.9128 | 0.8969 | 0.9063 | -0.7327 | 0.7333 | 0.9067 | 0.6596 | 0.9655 | 0.5316 | 0.7859 | 0.8362 | 0.7835 | 0.9780 | -0.9722 | 0.9320 | 0.8247 | -0.9811 | 0.9384 0.9626 0.9705 -0.4502 0.7514 0.9627 0.5375 0.9155 0.7836 0.8254 0.2413 0.6552 0.9673 0.9770 0.9164 0.9369 -0.8158 0.9407 0.8501 0.9984 0.8781 0.8301 0.7441 0.9809 0.9810 0.9752 0.9806 -0.9922 -0.9801 0.9955 0.9714 -0.9883 0.7647 -0.9861 0.9480 0.9675 0.9476 0.9769 0.7825 1.0000 0.8728 0.8397 0.9798 0.9901 |
| Root PK | ##### | 0.9856 | -0.9205 | -0.7076 | -0.7690 | 0.9140 | 0.9925 | -0.8995 | ##### | 0.9505 | 0.8046 | 0.3987 | 0.8291 | 0.6622 | 0.6259 | 0.9575 | 0.9249 | -0.5008 | 0.9341 | 0.8126 | 0.9498 | 0.8893 | 0.9941 | 0.9665 | 0.9679 | 0.9682 | 0.9146 | 0.9923 | -0.2422 | 0.8661 | 0.9085 | 0.9944 | -0.9713 | 0.8575 | -0.7290 | 0.6460 | 0.9411 | 0.9711 | -0.3454 | 0.9613 | 0.7580 | 0.8959 | -0.9721 | 0.9240 | 0.9690 | 0.6260 | 0.8434 | -0.6940 | 0.6821 | 0.7070 | 0.9382 | 0.9401 | 0.1086 | 0.9838 | 0.7858 | 0.5409 | 0.8071 | -0.7761 | 0.6541 | 0.9734 | -0.9303 | 0.6724 0.8872 0.8550 -0.8182 0.3438 0.8650 0.1103 0.9882 0.9574 0.5292 0.0310 0.8371 0.7850 0.9365 0.9760 0.9366 -0.9895 0.9600 0.9639 0.8776 0.9852 0.9792 0.3548 0.8877 0.8993 0.8763 0.8924 -0.8701 -0.7673 0.9033 0.8862 -0.8501 0.9423 -0.7975 0.9417 0.7626 0.9710 0.9514 0.9653 0.8728 1.0000 0.9960 0.9161 0.9292 |
| Root CS | ##### | 0.9773 | -0.9022 | -0.6846 | -0.7438 | 0.9240 | 0.9779 | -0.8736 | ##### | 0.9486 | 0.8161 | 0.3619 | 0.8332 | 0.6471 | 0.6079 | 0.9508 | 0.9193 | -0.5602 | 0.9456 | 0.8045 | 0.9344 | 0.8478 | 0.9969 | 0.9690 | 0.9737 | 0.9842 | 0.9119 | 0.9986 | -0.2291 | 0.8892 | 0.9044 | 0.9944 | -0.9679 | 0.8415 | -0.6985 | 0.6163 | 0.9341 | 0.9857 | -0.3058 | 0.9468 | 0.7559 | 0.8992 | -0.9805 | 0.9027 | 0.9656 | 0.5996 | 0.8369 | -0.6379 | 0.6892 | 0.6833 | 0.9618 | 0.9083 | 0.0817 | 0.9954 | 0.7929 | 0.5270 | 0.7799 | -0.7458 | 0.6168 | 0.9763 | -0.9134 | 0.6365 0.8753 0.8369 -0.8397 0.2945 0.8196 0.0725 0.9772 0.9739 0.5082 0.0612 0.8114 0.7607 0.9210 0.9717 0.9305 -0.9978 0.9523 0.9521 0.8484 0.9778 0.9688 0.3043 0.8673 0.8816 0.8604 0.8745 -0.8455 -0.7246 0.8777 0.8712 -0.8256 0.9660 -0.7649 0.9152 0.7272 0.9624 0.9307 0.9783 0.8397 0.9960 1.0000 0.8880 0.9061 |
| Root ACO | ##### | 0.9521 | -0.9779 | -0.8916 | -0.9380 | 0.7882 | 0.9388 | -0.9732 | ##### | 0.9156 | 0.8059 | 0.7007 | 0.8195 | 0.8516 | 0.8113 | 0.9408 | 0.9394 | -0.3827 | 0.7171 | 0.8878 | 0.9641 | 0.9768 | 0.8827 | 0.7994 | 0.8991 | 0.7950 | 0.9206 | 0.8638 | -0.5113 | 0.8123 | 0.9206 | 0.8722 | -0.9571 | 0.9087 | -0.9211 | 0.8520 | 0.9395 | 0.7983 | -0.6711 | 0.8744 | 0.8442 | 0.8060 | -0.8816 | 0.9615 | 0.9105 | 0.8276 | 0.8469 | -0.8021 | 0.6352 | 0.9067 | 0.7340 | 0.9655 | 0.4235 | 0.8438 | 0.7741 | 0.6637 | 0.9575 | -0.9459 | 0.8670 | 0.8380 | -0.9755 | 0.8689 0.9324 0.9273 -0.5764 0.6685 0.9423 0.3791 0.9291 0.8061 0.7550 0.1952 0.6954 0.9164 0.9716 0.9198 0.9176 -0.8604 0.9341 0.9302 0.9817 0.8914 0.8626 0.6937 0.9843 0.9782 0.9612 0.9765 -0.9748 -0.9258 0.9861 0.9701 -0.9658 0.8042 -0.9496 0.9899 0.9509 0.9668 0.9716 0.8068 0.9798 0.9161 0.8880 1.0000 0.9856 |
| Root NADP-IDH | ##### | 0.9741 | -0.9969 | -0.9043 | -0.9385 | 0.7516 | 0.9484 | -0.9961 | ##### | 0.9626 | 0.8756 | 0.6990 | 0.8980 | 0.8578 | 0.8463 | 0.9782 | 0.9763 | -0.3587 | 0.7498 | 0.9377 | 0.9932 | 0.9678 | 0.9123 | 0.8098 | 0.9397 | 0.8307 | 0.9653 | 0.8845 | -0.5401 | 0.8592 | 0.9667 | 0.8962 | -0.9664 | 0.9635 | -0.9232 | 0.8755 | 0.9791 | 0.8249 | -0.6520 | 0.9176 | 0.8958 | 0.8890 | -0.9235 | 0.9939 | 0.9565 | 0.8586 | 0.9235 | -0.7153 | 0.7551 | 0.8854 | 0.7570 | 0.9756 | 0.4485 | 0.8638 | 0.8624 | 0.7546 | 0.9626 | -0.9494 | 0.8863 | 0.8879 | -0.9969 | 0.8962 0.9760 0.9719 -0.5565 0.6646 0.9489 0.4458 0.9575 0.8596 0.7830 0.2289 0.6992 0.9509 0.9957 0.9604 0.9682 -0.8876 0.9752 0.8979 0.9925 0.9280 0.8847 0.6601 0.9859 0.9901 0.9819 0.9882 -0.9888 -0.9456 0.9977 0.9813 -0.9817 0.8458 -0.9637 0.9653 0.9394 0.9828 0.9950 0.8578 0.9901 0.9292 0.9061 0.9856 1.0000 |
