# Supplementary material for: Adaptive responses of carbon and nitrogen metabolisms to nitrogen-deficiency in Citrus sinensis seedlings
Source: BMC Plant Biol. 2022 Jul 26;22:370. doi: 10.1186/s12870-022-03759-7 (PMC9316421; doi:10.1186/s12870-022-03759-7)
Supplement: Supplementary file 12 — Additional file 12: Table S7. Pearson correlation coefficient matrix for the mean values of 102 physiological parameters in Citrus sinensis leaves. [file 12870_2022_3759_MOESM12_ESM.docx]

| **Additional file 12: Table S7.** Pearson correlation coefficient matrix for the mean values of 102 physiological parameters in *Citrus sinensis* leaves | | | | | | | | |  |  |  |  |  |  |  |  |  |  |  |  |  |  |  |  |  |  |  |  |  |  |  |  |  |  |  |  |  |  |  |  |  |  |  |  |  |  |  |  |  |  |  |  |  |  |  |  |  |  |  |  |  |  |
| --- | --- | --- | --- | --- | --- | --- | --- | --- | --- | --- | --- | --- | --- | --- | --- | --- | --- | --- | --- | --- | --- | --- | --- | --- | --- | --- | --- | --- | --- | --- | --- | --- | --- | --- | --- | --- | --- | --- | --- | --- | --- | --- | --- | --- | --- | --- | --- | --- | --- | --- | --- | --- | --- | --- | --- | --- | --- | --- | --- | --- | --- | --- |
|  | Leaf C | Leaf N | Leaf C/N | C distribution in leaves | N distribution in leaves | Leaf NH_4_^+^-N | Leaf NO_3_^-^-N | Leaf NH_4_^+^-N/NO_3_^-^-N | Leaf TSP | Leaf Gly | Leaf Ala | Leaf Val | Leaf Leu | Leaf Met | Leaf Ile | Leaf Pro | Leaf Ser | Leaf Trp | Leaf Phe | Leaf Tyr | Leaf Glu | Leaf Asp | Leaf Asn | Leaf Gln | Leaf Lys | Leaf Arg | Leaf Thr | Leaf L-citrulline | Leaf 5-hydroxy-tryptamine | Leaf L-homocitrulline | Leaf beta-alanine | Leaf L-pipecolic-acid | Leaf 3-N-methyl-L-histidine | Leaf homoserine | Leaf L-cystathionine | Leaf N6-acetyl-L-lysine | Leaf trans-4-hydroxy-L-proline | Leaf L-ornithine | Leaf L-tyrosine-methyl-ester | Leaf N-acetylaspartate | Leaf (5-L-glutamyl)-L-amino-acid | Leaf glycyl-L-proline | Leaf trimethylamine-N-oxide | Leaf N8-acetylspermidine | Leaf glutathione-oxidized | Leaf methionine-sulfoxide | Leaf Asp-Phe | Leaf Nα-acetyl-L-arginine | Leaf N-glycyl-L-leucine | Leaf γ-glutamate-cysteine | Leaf Nα-acetyl-L-glutamine | Leaf N-acetyl-L-tyrosine | Leaf D-alanyl-D-alanine | Leaf homo-Arg | Leaf L-carnosine | Leaf S-(5-adenosy)-L-homocysteine | Leaf argininosuccinic-acid | Leaf succinic-acid | Leaf 5-aminovaleric-acid | Leaf α-aminoadipic-acid | Leaf 2-aminobutyric-acid | Leaf γ-aminobutyric-acid Leaf 4-acetamidobutyric-acid Leaf 6-aminocaproic-acid Leaf creatine-phosphate Leaf kynurenic-acid Leaf N'-formylkynurenine Leaf 2-aminoethanesulfonic-acid Leaf 3,7-dimethyluric-acid Leaf ethanolamine Leaf Cys Leaf creatine Leaf TFAADs Leaf TFAADs/N Leaf molar ratio of C/N in TFAADs Leaf TFAADs/C Leaf NR Leaf GOGAT Leaf GOT Leaf GPT Leaf GS Leaf glucose Leaf fructose Leaf sucrose Leaf total soluble sugars Leaf starch Leaf TNC Leaf sucrose/starch Leaf malate Leaf citrate Leaf isocitrate Leaf malate + citrate + isocitrate Leaf NADP-ME Leaf NAD-ME Leaf NADP-MDH Leaf NAD-MDH Leaf PEPC Leaf PEPP Leaf PK Leaf CS Leaf ACO Leaf NADP-IDH |
| Leaf C | 1.0000 | 0.9147 | -0.8711 | 0.4816 | 0.5301 | -0.9634 | 0.9190 | -0.9509 | 0.8839 | 0.8925 | 0.8963 | -0.0978 | 0.8495 | 0.9216 | -0.2921 | 0.8647 | 0.8733 | -0.7654 | -0.3895 | 0.9001 | 0.7474 | 0.9108 | 0.8962 | 0.9095 | 0.8193 | 0.9293 | 0.8396 | 0.8581 | -0.8177 | 0.9001 | 0.8908 | 0.7459 | 0.7757 | 0.8125 | 0.9356 | 0.9732 | 0.8386 | 0.7623 | 0.9648 | 0.6492 | 0.8889 | 0.6637 | -0.9169 | -0.8283 | 0.9237 | 0.8715 | 0.8971 | 0.8577 | 0.5808 | 0.5188 | 0.8628 | 0.9190 | 0.6800 | 0.8909 | -0.1348 | 0.8382 | 0.8996 | -0.8587 | 0.7892 | 0.1482 | 0.9039 | 0.8713 0.7708 0.8063 -0.6896 -0.6371 0.8396 0.5413 0.9349 0.7022 -0.7865 -0.8739 0.9232 0.9173 -0.9491 0.9141 0.7889 0.7459 0.9799 0.9273 0.8234 0.7506 0.8432 0.6857 0.7348 0.8530 0.8446 -0.8148 0.4697 -0.6766 0.9181 0.4952 0.8717 0.8845 0.8387 0.7762 0.8543 0.7394 0.7981 0.8555 0.7454 0.8245 |
| Leaf N | 0.9147 | 1.0000 | -0.9829 | 0.6101 | 0.6716 | -0.9588 | 0.9103 | -0.9827 | 0.9951 | 0.9282 | 0.9780 | -0.1962 | 0.8466 | 0.9910 | -0.4490 | 0.9617 | 0.9879 | -0.8938 | -0.4846 | 0.9908 | 0.9159 | 0.9840 | 0.9279 | 0.9433 | 0.9155 | 0.9348 | 0.9245 | 0.8904 | -0.8263 | 0.8143 | 0.9766 | 0.8032 | 0.8756 | 0.7800 | 0.9726 | 0.9457 | 0.9499 | 0.8246 | 0.9834 | 0.5538 | 0.9683 | 0.7593 | -0.9889 | -0.9374 | 0.9703 | 0.9797 | 0.9953 | 0.9696 | 0.2831 | 0.7310 | 0.9133 | 0.9743 | 0.8077 | 0.9917 | -0.4250 | 0.9473 | 0.8477 | -0.8586 | 0.9146 | -0.1330 | 0.9926 | 0.9630 0.8450 0.9264 -0.9053 -0.8089 0.9807 0.6061 0.7697 0.8403 -0.9138 -0.8184 0.9888 0.9806 -0.9793 0.9898 0.9696 0.8901 0.9537 0.8853 0.9524 0.8903 0.9759 0.9211 0.9426 0.9430 0.9501 -0.9191 0.6171 -0.7597 0.8603 0.6366 0.9631 0.9969 0.9833 0.9645 0.9654 0.9433 0.9517 0.9635 0.9438 0.9818 |
| Leaf C/N | -0.8711 | -0.9829 | 1.0000 | -0.7433 | -0.7955 | 0.9204 | -0.9187 | 0.9788 | -0.9700 | -0.8621 | -0.9965 | 0.3630 | -0.7362 | -0.9900 | 0.6045 | -0.9940 | -0.9941 | 0.9607 | 0.6329 | -0.9532 | -0.9726 | -0.9807 | -0.8559 | -0.8681 | -0.8351 | -0.8544 | -0.8517 | -0.7956 | 0.7769 | -0.7390 | -0.9885 | -0.7115 | -0.7845 | -0.8473 | -0.9674 | -0.9042 | -0.9125 | -0.7130 | -0.9556 | -0.5424 | -0.9938 | -0.8536 | 0.9921 | 0.8686 | -0.9219 | -0.9543 | -0.9851 | -0.9927 | -0.1736 | -0.8411 | -0.8246 | -0.9894 | -0.8994 | -0.9570 | 0.3882 | -0.9873 | -0.7432 | 0.7562 | -0.9729 | 0.2988 | -0.9561 | -0.9937 -0.9219 -0.9794 0.9352 0.9008 -0.9510 -0.4977 -0.6805 -0.9204 0.9726 0.8580 -0.9548 -0.9660 0.9476 -0.9590 -0.9731 -0.9569 -0.9048 -0.8304 -0.9907 -0.9588 -0.9984 -0.9372 -0.9671 -0.9832 -0.9887 0.9740 -0.7481 0.7233 -0.8050 -0.7653 -0.9563 -0.9910 -0.9866 -0.9686 -0.9890 -0.9705 -0.9904 -0.9954 -0.9738 -0.9731 |
| C distribution in leaves | 0.4816 | 0.6101 | -0.7433 | 1.0000 | 0.9959 | -0.4994 | 0.7080 | -0.6704 | 0.5622 | 0.3358 | 0.7554 | -0.8404 | 0.1150 | 0.6856 | -0.9652 | 0.8026 | 0.6913 | -0.8995 | -0.9832 | 0.5126 | 0.8571 | 0.6539 | 0.3129 | 0.3179 | 0.2596 | 0.3017 | 0.3173 | 0.1896 | -0.4007 | 0.2408 | 0.7139 | 0.1150 | 0.1784 | 0.8726 | 0.6467 | 0.4834 | 0.4564 | 0.0650 | 0.5648 | 0.4242 | 0.7801 | 0.9535 | -0.6926 | -0.3268 | 0.4480 | 0.5387 | 0.6275 | 0.7527 | -0.1684 | 0.9566 | 0.2357 | 0.7460 | 0.9572 | 0.5189 | -0.0147 | 0.8279 | 0.1393 | -0.1383 | 0.8740 | -0.7440 | 0.5149 | 0.7985 0.9273 0.8628 -0.7291 -0.9458 0.5227 -0.1129 0.2113 0.9291 -0.8783 -0.7648 0.5237 0.6008 -0.5358 0.5356 0.6500 0.8844 0.4603 0.3729 0.8070 0.9008 0.7554 0.6580 0.7193 0.8195 0.8131 -0.8688 0.9949 -0.3961 0.3559 0.9976 0.6096 0.6490 0.6777 0.6466 0.7508 0.7322 0.8054 0.7997 0.7450 0.6095 |
| N distribution in leaves | 0.5301 | 0.6716 | -0.7955 | 0.9959 | 1.0000 | -0.5618 | 0.7394 | -0.7222 | 0.6290 | 0.4122 | 0.8038 | -0.8126 | 0.1881 | 0.7378 | -0.9539 | 0.8466 | 0.7500 | -0.9324 | -0.9668 | 0.5798 | 0.8986 | 0.7131 | 0.3897 | 0.3929 | 0.3400 | 0.3733 | 0.3889 | 0.2698 | -0.4420 | 0.3060 | 0.7686 | 0.2002 | 0.2623 | 0.8819 | 0.7028 | 0.5438 | 0.5325 | 0.1478 | 0.6245 | 0.4230 | 0.8256 | 0.9610 | -0.7474 | -0.4044 | 0.5192 | 0.6094 | 0.6904 | 0.8055 | -0.1626 | 0.9746 | 0.3136 | 0.7924 | 0.9749 | 0.5880 | -0.0910 | 0.8682 | 0.2127 | -0.2178 | 0.9115 | -0.7291 | 0.5834 | 0.8424 0.9487 0.9010 -0.7781 -0.9679 0.5932 -0.0267 0.2543 0.9516 -0.9145 -0.7899 0.5916 0.6636 -0.5997 0.6036 0.7140 0.9218 0.5217 0.4380 0.8545 0.9343 0.8080 0.7202 0.7769 0.8623 0.8581 -0.9053 0.9915 -0.4348 0.4193 0.9963 0.6741 0.7099 0.7354 0.7110 0.8039 0.7889 0.8534 0.8446 0.8010 0.6762 |
| Leaf NH_4_^+^-N | -0.9634 | -0.9588 | 0.9204 | -0.4994 | -0.5618 | 1.0000 | -0.8592 | 0.9711 | -0.9447 | -0.9696 | -0.9304 | 0.1580 | -0.8529 | -0.9410 | 0.3498 | -0.8991 | -0.9424 | 0.8091 | 0.3999 | -0.9442 | -0.8142 | -0.9729 | -0.9656 | -0.9567 | -0.8899 | -0.9515 | -0.8667 | -0.9180 | 0.7358 | -0.9416 | -0.9531 | -0.8666 | -0.8598 | -0.7658 | -0.9827 | -0.9985 | -0.9488 | -0.8225 | -0.9924 | -0.4654 | -0.9204 | -0.6420 | 0.9610 | 0.8935 | -0.9862 | -0.9615 | -0.9620 | -0.9267 | -0.3720 | -0.5846 | -0.9100 | -0.9390 | -0.6988 | -0.9510 | 0.3806 | -0.8653 | -0.9241 | 0.9149 | -0.8405 | -0.0163 | -0.9579 | -0.9023 -0.7830 -0.8493 0.7498 0.6778 -0.9151 -0.6963 -0.8346 -0.7187 0.8309 0.8685 -0.9826 -0.9836 0.9959 -0.9787 -0.8821 -0.8171 -0.9954 -0.9786 -0.8891 -0.8002 -0.9045 -0.7971 -0.8307 -0.9012 -0.8991 0.8550 -0.4824 0.6105 -0.9677 -0.5121 -0.9647 -0.9427 -0.8926 -0.8730 -0.9277 -0.8349 -0.8635 -0.8927 -0.8421 -0.9118 |
| Leaf NO_3_^-^-N | 0.9190 | 0.9103 | -0.9187 | 0.7080 | 0.7394 | -0.8592 | 1.0000 | -0.9410 | 0.8730 | 0.7422 | 0.9376 | -0.2663 | 0.7282 | 0.9535 | -0.5104 | 0.9345 | 0.8838 | -0.8868 | -0.6087 | 0.8814 | 0.8647 | 0.8807 | 0.7475 | 0.7929 | 0.7260 | 0.8164 | 0.8069 | 0.7026 | -0.8942 | 0.6725 | 0.8877 | 0.5286 | 0.6559 | 0.9078 | 0.8896 | 0.8600 | 0.7425 | 0.6210 | 0.9074 | 0.8116 | 0.9385 | 0.8770 | -0.9186 | -0.7669 | 0.8227 | 0.8138 | 0.8814 | 0.8818 | 0.4843 | 0.7347 | 0.7504 | 0.9515 | 0.8624 | 0.8613 | -0.0217 | 0.9384 | 0.7030 | -0.6690 | 0.8878 | -0.0973 | 0.8693 | 0.9393 0.8947 0.9067 -0.8385 -0.8316 0.8326 0.2659 0.8257 0.8871 -0.8979 -0.8574 0.8599 0.8601 -0.8712 0.8563 0.8275 0.8421 0.8754 0.7482 0.8845 0.8761 0.8991 0.7648 0.8172 0.9029 0.8989 -0.9054 0.7206 -0.8176 0.7255 0.7312 0.8107 0.8972 0.8992 0.8177 0.8717 0.8209 0.8848 0.9317 0.8224 0.8364 |
| Leaf NH_4_^+^-N/NO_3_^-^-N | -0.9509 | -0.9827 | 0.9788 | -0.6704 | -0.7222 | 0.9711 | -0.9410 | 1.0000 | -0.9604 | -0.9033 | -0.9884 | 0.2943 | -0.7898 | -0.9900 | 0.5140 | -0.9742 | -0.9778 | 0.9161 | 0.5695 | -0.9538 | -0.9134 | -0.9876 | -0.8989 | -0.9066 | -0.8440 | -0.9042 | -0.8568 | -0.8397 | 0.7939 | -0.8379 | -0.9851 | -0.7505 | -0.7949 | -0.8691 | -0.9911 | -0.9654 | -0.9137 | -0.7416 | -0.9905 | -0.5790 | -0.9846 | -0.8037 | 0.9948 | 0.8677 | -0.9520 | -0.9517 | -0.9805 | -0.9738 | -0.3271 | -0.7405 | -0.8556 | -0.9935 | -0.8422 | -0.9548 | 0.3001 | -0.9573 | -0.8270 | 0.8178 | -0.9340 | 0.1419 | -0.9597 | -0.9765 -0.8972 -0.9432 0.8536 0.8238 -0.9283 -0.5360 -0.7957 -0.8605 0.9311 0.9078 -0.9716 -0.9812 0.9806 -0.9707 -0.9251 -0.9091 -0.9651 -0.9055 -0.9581 -0.9088 -0.9668 -0.8585 -0.8996 -0.9678 -0.9665 0.9453 -0.6644 0.6974 -0.8878 -0.6869 -0.9594 -0.9764 -0.9510 -0.9168 -0.9712 -0.9045 -0.9440 -0.9702 -0.9105 -0.9397 |
| Leaf TSP | 0.8839 | 0.9951 | -0.9700 | 0.5622 | 0.6290 | -0.9447 | 0.8730 | -0.9604 | 1.0000 | 0.9372 | 0.9575 | -0.1428 | 0.8656 | 0.9751 | -0.4046 | 0.9392 | 0.9809 | -0.8660 | -0.4270 | 0.9960 | 0.9013 | 0.9719 | 0.9383 | 0.9540 | 0.9437 | 0.9408 | 0.9449 | 0.9113 | -0.8189 | 0.8034 | 0.9611 | 0.8336 | 0.9110 | 0.7174 | 0.9534 | 0.9279 | 0.9637 | 0.8613 | 0.9685 | 0.5126 | 0.9447 | 0.7143 | -0.9729 | -0.9625 | 0.9721 | 0.9858 | 0.9913 | 0.9555 | 0.2476 | 0.7090 | 0.9342 | 0.9491 | 0.7709 | 0.9985 | -0.4995 | 0.9242 | 0.8545 | -0.8766 | 0.8894 | -0.1242 | 0.9974 | 0.9399 0.8015 0.9016 -0.9122 -0.7824 0.9951 0.6531 0.7429 0.8095 -0.8885 -0.7630 0.9878 0.9716 -0.9696 0.9899 0.9794 0.8670 0.9386 0.8731 0.9358 0.8646 0.9659 0.9397 0.9504 0.9168 0.9281 -0.8902 0.5737 -0.7657 0.8457 0.5926 0.9584 0.9937 0.9834 0.9761 0.9511 0.9490 0.9403 0.9436 0.9472 0.9913 |
| Leaf Gly | 0.8925 | 0.9282 | -0.8621 | 0.3358 | 0.4122 | -0.9696 | 0.7422 | -0.9033 | 0.9372 | 1.0000 | 0.8567 | -0.0019 | 0.9018 | 0.8792 | -0.1987 | 0.8156 | 0.9042 | -0.7050 | -0.2187 | 0.9392 | 0.7396 | 0.9317 | 0.9987 | 0.9841 | 0.9518 | 0.9681 | 0.8995 | 0.9767 | -0.6879 | 0.9455 | 0.8991 | 0.9600 | 0.9438 | 0.5923 | 0.9291 | 0.9621 | 0.9785 | 0.9147 | 0.9507 | 0.3314 | 0.8382 | 0.4847 | -0.9049 | -0.9420 | 0.9902 | 0.9661 | 0.9356 | 0.8704 | 0.3078 | 0.4778 | 0.9598 | 0.8568 | 0.5645 | 0.9515 | -0.5584 | 0.7750 | 0.9608 | -0.9747 | 0.7463 | 0.0876 | 0.9549 | 0.8177 0.6447 0.7553 -0.7170 -0.5638 0.9280 0.8418 0.7817 0.5966 -0.7339 -0.7323 0.9726 0.9545 -0.9696 0.9698 0.8762 0.7284 0.9632 0.9688 0.8206 0.7000 0.8511 0.8035 0.8120 0.8138 0.8198 -0.7541 0.3240 -0.5807 0.9556 0.3546 0.9463 0.9126 0.8606 0.8704 0.8725 0.8119 0.8014 0.8137 0.8148 0.9100 |
| Leaf Ala | 0.8963 | 0.9780 | -0.9965 | 0.7554 | 0.8038 | -0.9304 | 0.9376 | -0.9884 | 0.9575 | 0.8567 | 1.0000 | -0.3832 | 0.7267 | 0.9918 | -0.6130 | 0.9968 | 0.9880 | -0.9631 | -0.6537 | 0.9422 | 0.9642 | 0.9818 | 0.8499 | 0.8607 | 0.8121 | 0.8517 | 0.8324 | 0.7835 | -0.7768 | 0.7568 | 0.9900 | 0.6938 | 0.7577 | 0.8832 | 0.9759 | 0.9184 | 0.8972 | 0.6888 | 0.9630 | 0.5681 | 0.9991 | 0.8663 | -0.9946 | -0.8455 | 0.9185 | 0.9426 | 0.9791 | 0.9907 | 0.2183 | 0.8316 | 0.8098 | 0.9979 | 0.9056 | 0.9446 | -0.3250 | 0.9887 | 0.7468 | -0.7491 | 0.9746 | -0.2768 | 0.9460 | 0.9974 0.9378 0.9811 -0.9095 -0.8974 0.9306 0.4716 0.7100 0.9221 -0.9738 -0.8944 0.9506 0.9667 -0.9517 0.9533 0.9513 0.9562 0.9170 0.8443 0.9879 0.9591 0.9915 0.9051 0.9434 0.9893 0.9911 -0.9796 0.7553 -0.7080 0.8220 0.7738 0.9526 0.9826 0.9725 0.9451 0.9874 0.9483 0.9820 0.9956 0.9536 0.9540 |
| Leaf Val | -0.0978 | -0.1962 | 0.3630 | -0.8404 | -0.8126 | 0.1580 | -0.2663 | 0.2943 | -0.1428 | -0.0019 | -0.3832 | 1.0000 | 0.3481 | -0.2670 | 0.9432 | -0.4387 | -0.3296 | 0.5915 | 0.9147 | -0.0684 | -0.5269 | -0.3135 | 0.0394 | 0.0902 | 0.1692 | 0.1269 | 0.1762 | 0.1986 | -0.1498 | -0.0041 | -0.3833 | 0.1459 | 0.2276 | -0.6129 | -0.3193 | -0.1464 | -0.1331 | 0.3541 | -0.1854 | 0.0525 | -0.4185 | -0.6442 | 0.3255 | -0.1193 | -0.0819 | -0.1847 | -0.2507 | -0.4237 | 0.4982 | -0.7191 | 0.1979 | -0.3726 | -0.6685 | -0.0980 | -0.0444 | -0.4527 | 0.2204 | -0.2192 | -0.5647 | 0.8615 | -0.0929 | -0.4313 -0.6512 -0.5316 0.3013 0.6500 -0.0976 0.2633 0.2136 -0.5960 0.5544 0.5587 -0.1428 -0.2652 0.1765 -0.1574 -0.2647 -0.6137 -0.0899 -0.1073 -0.4801 -0.5954 -0.3852 -0.2826 -0.3478 -0.5081 -0.4917 0.5550 -0.7985 -0.1639 -0.1137 -0.8055 -0.3118 -0.2488 -0.2508 -0.2624 -0.4336 -0.3678 -0.4511 -0.4277 -0.3927 -0.2175 |
| Leaf Leu | 0.8495 | 0.8466 | -0.7362 | 0.1150 | 0.1881 | -0.8529 | 0.7282 | -0.7898 | 0.8656 | 0.9018 | 0.7267 | 0.3481 | 1.0000 | 0.8001 | 0.0934 | 0.6774 | 0.7622 | -0.5247 | 0.0361 | 0.9026 | 0.5727 | 0.7780 | 0.9224 | 0.9579 | 0.9584 | 0.9717 | 0.9640 | 0.9668 | -0.8534 | 0.8201 | 0.7307 | 0.8617 | 0.9524 | 0.4323 | 0.7718 | 0.8508 | 0.8387 | 0.9751 | 0.8530 | 0.5480 | 0.6991 | 0.3621 | -0.7727 | -0.9482 | 0.8938 | 0.8367 | 0.8146 | 0.6963 | 0.5759 | 0.2802 | 0.9798 | 0.7335 | 0.3958 | 0.8887 | -0.4011 | 0.6507 | 0.9547 | -0.9560 | 0.5614 | 0.3768 | 0.8946 | 0.6833 0.4558 0.5897 -0.6638 -0.3981 0.8694 0.7386 0.8816 0.4612 -0.5638 -0.5145 0.8733 0.8040 -0.8546 0.8648 0.7635 0.5111 0.8852 0.8235 0.6443 0.5171 0.7142 0.6947 0.6864 0.6286 0.6407 -0.5747 0.1365 -0.7771 0.8006 0.1546 0.7604 0.8111 0.7859 0.7583 0.6884 0.6777 0.6510 0.6821 0.6673 0.8058 |
| Leaf Met | 0.9216 | 0.9910 | -0.9900 | 0.6856 | 0.7378 | -0.9410 | 0.9535 | -0.9900 | 0.9751 | 0.8792 | 0.9918 | -0.2670 | 0.8001 | 1.0000 | -0.5185 | 0.9825 | 0.9817 | -0.9284 | -0.5692 | 0.9704 | 0.9363 | 0.9755 | 0.8786 | 0.9009 | 0.8604 | 0.8990 | 0.8901 | 0.8316 | -0.8447 | 0.7728 | 0.9761 | 0.7214 | 0.8088 | 0.8483 | 0.9689 | 0.9305 | 0.9038 | 0.7551 | 0.9750 | 0.6274 | 0.9867 | 0.8314 | -0.9908 | -0.8909 | 0.9370 | 0.9483 | 0.9820 | 0.9723 | 0.3098 | 0.7772 | 0.8628 | 0.9917 | 0.8637 | 0.9668 | -0.3172 | 0.9746 | 0.7969 | -0.7968 | 0.9421 | -0.1735 | 0.9691 | 0.9843 0.8966 0.9543 -0.9129 -0.8564 0.9521 0.4988 0.7739 0.8913 -0.9441 -0.8570 0.9647 0.9644 -0.9614 0.9655 0.9519 0.9141 0.9383 0.8521 0.9641 0.9234 0.9811 0.9020 0.9346 0.9626 0.9664 -0.9492 0.6932 -0.7774 0.8272 0.7103 0.9406 0.9884 0.9808 0.9456 0.9667 0.9369 0.9635 0.9829 0.9386 0.9601 |
| Leaf Ile | -0.2921 | -0.4490 | 0.6045 | -0.9652 | -0.9539 | 0.3498 | -0.5104 | 0.5140 | -0.4046 | -0.1987 | -0.6130 | 0.9432 | 0.0934 | -0.5185 | 1.0000 | -0.6678 | -0.5618 | 0.7980 | 0.9817 | -0.3382 | -0.7550 | -0.5242 | -0.1662 | -0.1457 | -0.0924 | -0.1129 | -0.1172 | -0.0255 | 0.1546 | -0.1185 | -0.5919 | -0.0155 | -0.0222 | -0.7535 | -0.5142 | -0.3301 | -0.3432 | 0.1073 | -0.4034 | -0.1847 | -0.6421 | -0.8421 | 0.5503 | 0.1550 | -0.2992 | -0.4108 | -0.4869 | -0.6378 | 0.3924 | -0.9068 | -0.0551 | -0.5967 | -0.8638 | -0.3579 | 0.0619 | -0.6906 | 0.0324 | -0.0197 | -0.7713 | 0.8703 | -0.3505 | -0.6609 -0.8272 -0.7487 0.5923 0.8597 -0.3694 0.1614 0.0191 -0.8177 0.7692 0.6654 -0.3730 -0.4709 0.3865 -0.3886 -0.5251 -0.8042 -0.2917 -0.2487 -0.6972 -0.8032 -0.6264 -0.5505 -0.6073 -0.7075 -0.7002 0.7590 -0.9475 0.1629 -0.2385 -0.9510 -0.5040 -0.5009 -0.5248 -0.5240 -0.6412 -0.6226 -0.6887 -0.6637 -0.6398 -0.4759 |
| Leaf Pro | 0.8647 | 0.9617 | -0.9940 | 0.8026 | 0.8466 | -0.8991 | 0.9345 | -0.9742 | 0.9392 | 0.8156 | 0.9968 | -0.4387 | 0.6774 | 0.9825 | -0.6678 | 1.0000 | 0.9796 | -0.9809 | -0.7051 | 0.9197 | 0.9796 | 0.9667 | 0.8074 | 0.8200 | 0.7736 | 0.8096 | 0.8007 | 0.7352 | -0.7603 | 0.7059 | 0.9814 | 0.6426 | 0.7149 | 0.8974 | 0.9584 | 0.8852 | 0.8691 | 0.6389 | 0.9385 | 0.5702 | 0.9984 | 0.9000 | -0.9838 | -0.8127 | 0.8857 | 0.9204 | 0.9638 | 0.9878 | 0.1733 | 0.8721 | 0.7668 | 0.9926 | 0.9360 | 0.9222 | -0.3059 | 0.9969 | 0.6916 | -0.6957 | 0.9879 | -0.3430 | 0.9224 | 0.9999 0.9579 0.9930 -0.9219 -0.9297 0.9122 0.4139 0.6650 0.9496 -0.9885 -0.8910 0.9248 0.9463 -0.9247 0.9290 0.9460 0.9721 0.8827 0.8026 0.9926 0.9779 0.9915 0.9082 0.9481 0.9929 0.9948 -0.9910 0.8043 -0.7026 0.7791 0.8206 0.9346 0.9712 0.9683 0.9405 0.9841 0.9535 0.9894 0.9994 0.9589 0.9428 |
| Leaf Ser | 0.8733 | 0.9879 | -0.9941 | 0.6913 | 0.7500 | -0.9424 | 0.8838 | -0.9778 | 0.9809 | 0.9042 | 0.9880 | -0.3296 | 0.7622 | 0.9817 | -0.5618 | 0.9796 | 1.0000 | -0.9368 | -0.5796 | 0.9629 | 0.9556 | 0.9926 | 0.8967 | 0.8988 | 0.8683 | 0.8794 | 0.8642 | 0.8381 | -0.7448 | 0.7872 | 0.9944 | 0.7783 | 0.8266 | 0.8075 | 0.9787 | 0.9255 | 0.9501 | 0.7557 | 0.9667 | 0.4708 | 0.9827 | 0.7978 | -0.9939 | -0.8939 | 0.9504 | 0.9795 | 0.9960 | 0.9946 | 0.1510 | 0.8035 | 0.8564 | 0.9795 | 0.8578 | 0.9718 | -0.4640 | 0.9656 | 0.7856 | -0.8044 | 0.9552 | -0.2702 | 0.9705 | 0.9790 0.8896 0.9600 -0.9148 -0.8623 0.9653 0.5851 0.6789 0.8789 -0.9512 -0.8486 0.9747 0.9844 -0.9668 0.9789 0.9805 0.9435 0.9232 0.8702 0.9843 0.9355 0.9934 0.9413 0.9659 0.9744 0.9808 -0.9554 0.6912 -0.6835 0.8469 0.7119 0.9808 0.9953 0.9813 0.9763 0.9930 0.9690 0.9798 0.9806 0.9730 0.9847 |
| Leaf Trp | -0.7654 | -0.8938 | 0.9607 | -0.8995 | -0.9324 | 0.8091 | -0.8868 | 0.9161 | -0.8660 | -0.7050 | -0.9631 | 0.5915 | -0.5247 | -0.9284 | 0.7980 | -0.9809 | -0.9368 | 1.0000 | 0.8214 | -0.8333 | -0.9907 | -0.9130 | -0.6907 | -0.6984 | -0.6527 | -0.6813 | -0.6839 | -0.5988 | 0.6629 | -0.5860 | -0.9430 | -0.5179 | -0.5869 | -0.9121 | -0.9012 | -0.7916 | -0.7890 | -0.4918 | -0.8578 | -0.5149 | -0.9715 | -0.9484 | 0.9350 | 0.7017 | -0.7891 | -0.8489 | -0.9042 | -0.9618 | -0.0311 | -0.9461 | -0.6351 | -0.9541 | -0.9824 | -0.8397 | 0.2709 | -0.9861 | -0.5431 | 0.5524 | -0.9980 | 0.5101 | -0.8372 | -0.9788 -0.9855 -0.9969 0.9103 0.9791 -0.8375 -0.2867 -0.5248 -0.9835 0.9989 0.8784 -0.8414 -0.8818 0.8418 -0.8493 -0.9058 -0.9934 -0.7812 -0.7003 -0.9825 -0.9996 -0.9652 -0.8859 -0.9305 -0.9826 -0.9835 0.9962 -0.8992 0.6253 -0.6771 -0.9121 -0.8809 -0.9159 -0.9236 -0.9016 -0.9591 -0.9382 -0.9808 -0.9804 -0.9461 -0.8890 |
| Leaf Phe | -0.3895 | -0.4846 | 0.6329 | -0.9832 | -0.9668 | 0.3999 | -0.6087 | 0.5695 | -0.4270 | -0.2187 | -0.6537 | 0.9147 | 0.0361 | -0.5692 | 0.9817 | -0.7051 | -0.5796 | 0.8214 | 1.0000 | -0.3719 | -0.7616 | -0.5516 | -0.1906 | -0.1823 | -0.1049 | -0.1661 | -0.1549 | -0.0514 | 0.2543 | -0.1675 | -0.6170 | 0.0002 | -0.0240 | -0.8408 | -0.5542 | -0.3887 | -0.3363 | 0.0914 | -0.4566 | -0.3347 | -0.6839 | -0.8955 | 0.5877 | 0.1701 | -0.3285 | -0.4174 | -0.5096 | -0.6552 | 0.2197 | -0.8949 | -0.0866 | -0.6485 | -0.8947 | -0.3818 | -0.0812 | -0.7289 | -0.0218 | 0.0108 | -0.7926 | 0.7715 | -0.3794 | -0.7008 -0.8722 -0.7758 0.5934 0.8724 -0.3783 0.2108 -0.1123 -0.8502 0.7938 0.7370 -0.4026 -0.4971 0.4276 -0.4141 -0.5187 -0.8106 -0.3555 -0.2914 -0.7127 -0.8216 -0.6448 -0.5229 -0.5938 -0.7375 -0.7250 0.7896 -0.9657 0.2381 -0.2827 -0.9692 -0.5110 -0.5251 -0.5454 -0.5142 -0.6561 -0.6100 -0.7005 -0.6980 -0.6276 -0.4767 |
| Leaf Tyr | 0.9001 | 0.9908 | -0.9532 | 0.5126 | 0.5798 | -0.9442 | 0.8814 | -0.9538 | 0.9960 | 0.9392 | 0.9422 | -0.0684 | 0.9026 | 0.9704 | -0.3382 | 0.9197 | 0.9629 | -0.8333 | -0.3719 | 1.0000 | 0.8689 | 0.9565 | 0.9444 | 0.9676 | 0.9575 | 0.9613 | 0.9670 | 0.9290 | -0.8572 | 0.8105 | 0.9407 | 0.8355 | 0.9257 | 0.6984 | 0.9403 | 0.9302 | 0.9510 | 0.8879 | 0.9680 | 0.5583 | 0.9273 | 0.6874 | -0.9600 | -0.9742 | 0.9720 | 0.9738 | 0.9796 | 0.9310 | 0.3244 | 0.6605 | 0.9543 | 0.9371 | 0.7360 | 0.9977 | -0.4645 | 0.9047 | 0.8798 | -0.8961 | 0.8576 | -0.0440 | 0.9981 | 0.9215 0.7699 0.8736 -0.8954 -0.7446 0.9911 0.6534 0.7891 0.7803 -0.8585 -0.7413 0.9844 0.9586 -0.9662 0.9845 0.9607 0.8279 0.9465 0.8726 0.9077 0.8305 0.9452 0.9153 0.9250 0.8901 0.9013 -0.8612 0.5278 -0.8004 0.8447 0.5458 0.9372 0.9831 0.9730 0.9567 0.9251 0.9224 0.9140 0.9243 0.9188 0.9771 |
| Leaf Glu | 0.7474 | 0.9159 | -0.9726 | 0.8571 | 0.8986 | -0.8142 | 0.8647 | -0.9134 | 0.9013 | 0.7396 | 0.9642 | -0.5269 | 0.5727 | 0.9363 | -0.7550 | 0.9796 | 0.9556 | -0.9907 | -0.7616 | 0.8689 | 1.0000 | 0.9237 | 0.7273 | 0.7373 | 0.7155 | 0.7138 | 0.7385 | 0.6490 | -0.6803 | 0.5876 | 0.9493 | 0.5762 | 0.6577 | 0.8511 | 0.9012 | 0.7911 | 0.8321 | 0.5625 | 0.8641 | 0.4808 | 0.9682 | 0.9147 | -0.9415 | -0.7619 | 0.8167 | 0.8837 | 0.9268 | 0.9706 | -0.0102 | 0.9426 | 0.6875 | 0.9485 | 0.9601 | 0.8770 | -0.3812 | 0.9846 | 0.5717 | -0.5967 | 0.9922 | -0.5074 | 0.8716 | 0.9766 0.9533 0.9921 -0.9504 -0.9712 0.8855 0.3601 0.5083 0.9707 -0.9934 -0.8249 0.8667 0.8957 -0.8543 0.8762 0.9483 0.9894 0.7852 0.7036 0.9886 0.9931 0.9810 0.9401 0.9705 0.9748 0.9817 -0.9852 0.8642 -0.6611 0.6758 0.8762 0.9000 0.9407 0.9541 0.9463 0.9673 0.9754 0.9948 0.9830 0.9799 0.9305 |
| Leaf Asp | 0.9108 | 0.9840 | -0.9807 | 0.6539 | 0.7131 | -0.9729 | 0.8807 | -0.9876 | 0.9719 | 0.9317 | 0.9818 | -0.3135 | 0.7780 | 0.9755 | -0.5242 | 0.9667 | 0.9926 | -0.9130 | -0.5516 | 0.9565 | 0.9237 | 1.0000 | 0.9231 | 0.9165 | 0.8677 | 0.8999 | 0.8529 | 0.8602 | -0.7249 | 0.8512 | 0.9965 | 0.8102 | 0.8286 | 0.8173 | 0.9957 | 0.9614 | 0.9581 | 0.7640 | 0.9846 | 0.4538 | 0.9763 | 0.7623 | -0.9955 | -0.8863 | 0.9688 | 0.9820 | 0.9940 | 0.9880 | 0.2056 | 0.7499 | 0.8666 | 0.9792 | 0.8241 | 0.9669 | -0.4368 | 0.9448 | 0.8297 | -0.8382 | 0.9358 | -0.2018 | 0.9685 | 0.9672 0.8767 0.9400 -0.8603 -0.8179 0.9487 0.6230 0.7246 0.8405 -0.9285 -0.8834 0.9844 0.9974 -0.9867 0.9862 0.9536 0.9230 0.9552 0.9206 0.9692 0.9091 0.9754 0.8971 0.9282 0.9692 0.9714 -0.9410 0.6440 -0.6369 0.9029 0.6691 0.9913 0.9850 0.9557 0.9474 0.9883 0.9328 0.9541 0.9642 0.9396 0.9657 |
| Leaf Asn | 0.8962 | 0.9279 | -0.8559 | 0.3129 | 0.3897 | -0.9656 | 0.7475 | -0.8989 | 0.9383 | 0.9987 | 0.8499 | 0.0394 | 0.9224 | 0.8786 | -0.1662 | 0.8074 | 0.8967 | -0.6907 | -0.1906 | 0.9444 | 0.7273 | 0.9231 | 1.0000 | 0.9910 | 0.9624 | 0.9783 | 0.9162 | 0.9856 | -0.7138 | 0.9404 | 0.8883 | 0.9586 | 0.9546 | 0.5789 | 0.9199 | 0.9586 | 0.9722 | 0.9312 | 0.9487 | 0.3593 | 0.8301 | 0.4747 | -0.8984 | -0.9524 | 0.9890 | 0.9608 | 0.9309 | 0.8587 | 0.3417 | 0.4593 | 0.9720 | 0.8507 | 0.5501 | 0.9538 | -0.5465 | 0.7680 | 0.9698 | -0.9825 | 0.7319 | 0.1223 | 0.9575 | 0.8100 0.6287 0.7433 -0.7179 -0.5498 0.9306 0.8388 0.8009 0.5864 -0.7211 -0.7138 0.9708 0.9465 -0.9658 0.9673 0.8719 0.7101 0.9636 0.9616 0.8081 0.6854 0.8435 0.7990 0.8055 0.8001 0.8069 -0.7404 0.3048 -0.6092 0.9471 0.3344 0.9341 0.9098 0.8605 0.8661 0.8597 0.8044 0.7917 0.8063 0.8058 0.9070 |
| Leaf Gln | 0.9095 | 0.9433 | -0.8681 | 0.3179 | 0.3929 | -0.9567 | 0.7929 | -0.9066 | 0.9540 | 0.9841 | 0.8607 | 0.0902 | 0.9579 | 0.9009 | -0.1457 | 0.8200 | 0.8988 | -0.6984 | -0.1823 | 0.9676 | 0.7373 | 0.9165 | 0.9910 | 1.0000 | 0.9804 | 0.9956 | 0.9590 | 0.9899 | -0.7979 | 0.9052 | 0.8822 | 0.9272 | 0.9669 | 0.5874 | 0.9104 | 0.9496 | 0.9554 | 0.9515 | 0.9526 | 0.4628 | 0.8398 | 0.5077 | -0.9026 | -0.9761 | 0.9844 | 0.9543 | 0.9343 | 0.8548 | 0.4092 | 0.4702 | 0.9911 | 0.8622 | 0.5677 | 0.9687 | -0.4974 | 0.7882 | 0.9689 | -0.9787 | 0.7356 | 0.1532 | 0.9725 | 0.8234 0.6345 0.7525 -0.7587 -0.5689 0.9487 0.7886 0.8405 0.6134 -0.7304 -0.6916 0.9721 0.9363 -0.9616 0.9679 0.8832 0.7041 0.9644 0.9313 0.8088 0.6927 0.8536 0.8146 0.8193 0.7975 0.8064 -0.7448 0.3217 -0.7069 0.9117 0.3466 0.9133 0.9224 0.8862 0.8776 0.8521 0.8162 0.8022 0.8213 0.8137 0.9170 |
| Leaf Lys | 0.8193 | 0.9155 | -0.8351 | 0.2596 | 0.3400 | -0.8899 | 0.7260 | -0.8440 | 0.9437 | 0.9518 | 0.8121 | 0.1692 | 0.9584 | 0.8604 | -0.0924 | 0.7736 | 0.8683 | -0.6527 | -0.1049 | 0.9575 | 0.7155 | 0.8677 | 0.9624 | 0.9804 | 1.0000 | 0.9691 | 0.9787 | 0.9825 | -0.7934 | 0.8199 | 0.8322 | 0.9228 | 0.9951 | 0.4730 | 0.8447 | 0.8744 | 0.9420 | 0.9799 | 0.8946 | 0.4187 | 0.7868 | 0.4532 | -0.8539 | -0.9964 | 0.9487 | 0.9346 | 0.9046 | 0.8131 | 0.3268 | 0.4574 | 0.9936 | 0.8032 | 0.5197 | 0.9583 | -0.6018 | 0.7480 | 0.9271 | -0.9588 | 0.6894 | 0.1290 | 0.9573 | 0.7755 0.5602 0.7079 -0.7881 -0.5429 0.9577 0.8057 0.7589 0.5778 -0.6873 -0.5646 0.9388 0.8874 -0.9083 0.9375 0.8917 0.6606 0.8971 0.8524 0.7706 0.6517 0.8276 0.8491 0.8332 0.7388 0.7564 -0.6901 0.2782 -0.7433 0.8253 0.2979 0.8710 0.9009 0.8821 0.8901 0.8084 0.8258 0.7801 0.7814 0.8175 0.9191 |
| Leaf Arg | 0.9293 | 0.9348 | -0.8544 | 0.3017 | 0.3733 | -0.9515 | 0.8164 | -0.9042 | 0.9408 | 0.9681 | 0.8517 | 0.1269 | 0.9717 | 0.8990 | -0.1129 | 0.8096 | 0.8794 | -0.6813 | -0.1661 | 0.9613 | 0.7138 | 0.8999 | 0.9783 | 0.9956 | 0.9691 | 1.0000 | 0.9620 | 0.9829 | -0.8352 | 0.9005 | 0.8641 | 0.8985 | 0.9519 | 0.5991 | 0.8987 | 0.9484 | 0.9260 | 0.9464 | 0.9498 | 0.5292 | 0.8310 | 0.5096 | -0.8917 | -0.9658 | 0.9720 | 0.9308 | 0.9179 | 0.8335 | 0.4907 | 0.4414 | 0.9883 | 0.8582 | 0.5563 | 0.9566 | -0.4210 | 0.7796 | 0.9739 | -0.9732 | 0.7167 | 0.2124 | 0.9625 | 0.8143 0.6263 0.7371 -0.7411 -0.5505 0.9313 0.7490 0.8857 0.6043 -0.7140 -0.6915 0.9597 0.9192 -0.9527 0.9534 0.8566 0.6775 0.9674 0.9219 0.7872 0.6735 0.8354 0.7809 0.7898 0.7813 0.7883 -0.7300 0.3079 -0.7382 0.9026 0.3315 0.8864 0.9082 0.8729 0.8498 0.8292 0.7864 0.7811 0.8101 0.7832 0.8934 |
| Leaf Thr | 0.8396 | 0.9245 | -0.8517 | 0.3173 | 0.3889 | -0.8667 | 0.8069 | -0.8568 | 0.9449 | 0.8995 | 0.8324 | 0.1762 | 0.9640 | 0.8901 | -0.1172 | 0.8007 | 0.8642 | -0.6839 | -0.1549 | 0.9670 | 0.7385 | 0.8529 | 0.9162 | 0.9590 | 0.9787 | 0.9620 | 1.0000 | 0.9443 | -0.8993 | 0.7517 | 0.8234 | 0.8282 | 0.9592 | 0.5313 | 0.8305 | 0.8533 | 0.8884 | 0.9514 | 0.8921 | 0.5871 | 0.8092 | 0.5399 | -0.8585 | -0.9876 | 0.9187 | 0.9030 | 0.8971 | 0.8088 | 0.4242 | 0.4996 | 0.9776 | 0.8273 | 0.5773 | 0.9552 | -0.4664 | 0.7881 | 0.8899 | -0.9098 | 0.7111 | 0.1377 | 0.9554 | 0.8039 0.6022 0.7361 -0.8357 -0.5955 0.9550 0.6719 0.8076 0.6421 -0.7172 -0.5678 0.9216 0.8632 -0.8898 0.9192 0.8888 0.6688 0.8866 0.7951 0.7776 0.6814 0.8403 0.8494 0.8397 0.7483 0.7655 -0.7147 0.3493 -0.8629 0.7625 0.3621 0.8301 0.9069 0.9066 0.8865 0.7990 0.8313 0.7978 0.8100 0.8197 0.9121 |
| Leaf L-citrulline | 0.8581 | 0.8904 | -0.7956 | 0.1896 | 0.2698 | -0.9180 | 0.7026 | -0.8397 | 0.9113 | 0.9767 | 0.7835 | 0.1986 | 0.9668 | 0.8316 | -0.0255 | 0.7352 | 0.8381 | -0.5988 | -0.0514 | 0.9290 | 0.6490 | 0.8602 | 0.9856 | 0.9899 | 0.9825 | 0.9829 | 0.9443 | 1.0000 | -0.7466 | 0.9022 | 0.8167 | 0.9576 | 0.9827 | 0.4712 | 0.8520 | 0.9108 | 0.9369 | 0.9781 | 0.9030 | 0.3802 | 0.7581 | 0.3832 | -0.8371 | -0.9678 | 0.9587 | 0.9204 | 0.8827 | 0.7845 | 0.4029 | 0.3612 | 0.9924 | 0.7836 | 0.4519 | 0.9322 | -0.5552 | 0.6977 | 0.9786 | -0.9948 | 0.6419 | 0.2357 | 0.9356 | 0.7386 0.5232 0.6592 -0.6879 -0.4590 0.9143 0.8540 0.8129 0.5023 -0.6347 -0.6009 0.9349 0.8886 -0.9199 0.9299 0.8327 0.6119 0.9273 0.9124 0.7290 0.5939 0.7814 0.7649 0.7581 0.7123 0.7236 -0.6499 0.1933 -0.6572 0.8943 0.2192 0.8689 0.8663 0.8244 0.8282 0.7827 0.7529 0.7226 0.7375 0.7488 0.8719 |
| Leaf 5-hydroxy-tryptamine | -0.8177 | -0.8263 | 0.7769 | -0.4007 | -0.4420 | 0.7358 | -0.8942 | 0.7939 | -0.8189 | -0.6879 | -0.7768 | -0.1498 | -0.8534 | -0.8447 | 0.1546 | -0.7603 | -0.7448 | 0.6629 | 0.2543 | -0.8572 | -0.6803 | -0.7249 | -0.7138 | -0.7979 | -0.7934 | -0.8352 | -0.8993 | -0.7466 | 1.0000 | -0.5606 | -0.7102 | -0.5273 | -0.7431 | -0.6283 | -0.7168 | -0.7339 | -0.6599 | -0.7554 | -0.7936 | -0.8804 | -0.7627 | -0.6560 | 0.7690 | 0.8246 | -0.7498 | -0.7196 | -0.7683 | -0.7004 | -0.6322 | -0.5022 | -0.8148 | -0.7868 | -0.6238 | -0.8202 | 0.0614 | -0.7718 | -0.7236 | 0.7029 | -0.6676 | -0.1792 | -0.8263 | -0.7669 -0.6301 -0.7028 0.7904 0.6165 -0.8093 -0.3027 -0.8530 -0.6882 0.6884 0.5561 -0.7752 -0.7158 0.7563 -0.7687 -0.7465 -0.6005 -0.7815 -0.6129 -0.6929 -0.6543 -0.7541 -0.7028 -0.7176 -0.6843 -0.6938 0.6824 -0.4481 0.9723 -0.5781 -0.4481 -0.6476 -0.7993 -0.8261 -0.7408 -0.6824 -0.7101 -0.7232 -0.7679 -0.6961 -0.7636 |
| Leaf L-homocitrulline | 0.9001 | 0.8143 | -0.7390 | 0.2408 | 0.3060 | -0.9416 | 0.6725 | -0.8379 | 0.8034 | 0.9455 | 0.7568 | -0.0041 | 0.8201 | 0.7728 | -0.1185 | 0.7059 | 0.7872 | -0.5860 | -0.1675 | 0.8105 | 0.5876 | 0.8512 | 0.9404 | 0.9052 | 0.8199 | 0.9005 | 0.7517 | 0.9022 | -0.5606 | 1.0000 | 0.8120 | 0.9069 | 0.8144 | 0.5908 | 0.8773 | 0.9502 | 0.8765 | 0.8024 | 0.8944 | 0.2744 | 0.7425 | 0.3733 | -0.8154 | -0.7962 | 0.9194 | 0.8572 | 0.8258 | 0.7647 | 0.4232 | 0.3093 | 0.8578 | 0.7744 | 0.4436 | 0.8244 | -0.3804 | 0.6510 | 0.9510 | -0.9311 | 0.6314 | 0.2329 | 0.8358 | 0.7107 0.5697 0.6374 -0.4965 -0.4101 0.7704 0.8062 0.8172 0.4557 -0.6122 -0.7718 0.8844 0.8828 -0.9126 0.8755 0.7034 0.6121 0.9393 0.9883 0.7015 0.5738 0.7164 0.5912 0.6219 0.7263 0.7186 -0.6527 0.2031 -0.3913 0.9923 0.2415 0.8673 0.7843 0.6985 0.6921 0.7714 0.6270 0.6550 0.6937 0.6381 0.7525 |
| Leaf beta-alanine | 0.8908 | 0.9766 | -0.9885 | 0.7139 | 0.7686 | -0.9531 | 0.8877 | -0.9851 | 0.9611 | 0.8991 | 0.9900 | -0.3833 | 0.7307 | 0.9761 | -0.5919 | 0.9814 | 0.9944 | -0.9430 | -0.6170 | 0.9407 | 0.9493 | 0.9965 | 0.8883 | 0.8822 | 0.8322 | 0.8641 | 0.8234 | 0.8167 | -0.7102 | 0.8120 | 1.0000 | 0.7646 | 0.7878 | 0.8471 | 0.9910 | 0.9402 | 0.9384 | 0.7148 | 0.9710 | 0.4583 | 0.9875 | 0.8074 | -0.9960 | -0.8567 | 0.9452 | 0.9687 | 0.9885 | 0.9968 | 0.1619 | 0.8002 | 0.8274 | 0.9859 | 0.8664 | 0.9516 | -0.4155 | 0.9639 | 0.7810 | -0.7904 | 0.9614 | -0.2740 | 0.9524 | 0.9812 0.9108 0.9638 -0.8773 -0.8606 0.9362 0.5682 0.6868 0.8785 -0.9550 -0.8974 0.9679 0.9884 -0.9705 0.9711 0.9555 0.9515 0.9316 0.8911 0.9854 0.9398 0.9855 0.9054 0.9399 0.9858 0.9876 -0.9650 0.7043 -0.6298 0.8727 0.7278 0.9848 0.9825 0.9588 0.9495 0.9970 0.9455 0.9716 0.9789 0.9531 0.9618 |
| Leaf L-pipecolic-acid | 0.7459 | 0.8032 | -0.7115 | 0.1150 | 0.2002 | -0.8666 | 0.5286 | -0.7505 | 0.8336 | 0.9600 | 0.6938 | 0.1459 | 0.8617 | 0.7214 | -0.0155 | 0.6426 | 0.7783 | -0.5179 | 0.0002 | 0.8355 | 0.5762 | 0.8102 | 0.9586 | 0.9272 | 0.9228 | 0.8985 | 0.8282 | 0.9576 | -0.5273 | 0.9069 | 0.7646 | 1.0000 | 0.9424 | 0.3552 | 0.8000 | 0.8555 | 0.9327 | 0.9234 | 0.8262 | 0.1038 | 0.6684 | 0.2446 | -0.7626 | -0.8939 | 0.9147 | 0.8881 | 0.8209 | 0.7316 | 0.2022 | 0.2949 | 0.9210 | 0.6869 | 0.3497 | 0.8572 | -0.7084 | 0.5916 | 0.9311 | -0.9656 | 0.5697 | 0.1682 | 0.8575 | 0.6434 0.4322 0.5753 -0.5761 -0.3654 0.8427 0.9588 0.6549 0.3853 -0.5516 -0.5468 0.8776 0.8477 -0.8643 0.8755 0.7747 0.5633 0.8548 0.9057 0.6676 0.5166 0.7058 0.7124 0.6971 0.6472 0.6584 -0.5714 0.1001 -0.4315 0.8960 0.1329 0.8556 0.7882 0.7266 0.7719 0.7371 0.6941 0.6480 0.6430 0.6951 0.8135 |
| Leaf 3-N-methyl-L-histidine | 0.7757 | 0.8756 | -0.7845 | 0.1784 | 0.2623 | -0.8598 | 0.6559 | -0.7949 | 0.9110 | 0.9438 | 0.7577 | 0.2276 | 0.9524 | 0.8088 | -0.0222 | 0.7149 | 0.8266 | -0.5869 | -0.0240 | 0.9257 | 0.6577 | 0.8286 | 0.9546 | 0.9669 | 0.9951 | 0.9519 | 0.9592 | 0.9827 | -0.7431 | 0.8144 | 0.7878 | 0.9424 | 1.0000 | 0.3924 | 0.8037 | 0.8436 | 0.9296 | 0.9896 | 0.8564 | 0.3446 | 0.7298 | 0.3681 | -0.8074 | -0.9844 | 0.9277 | 0.9108 | 0.8677 | 0.7663 | 0.3010 | 0.3892 | 0.9865 | 0.7470 | 0.4430 | 0.9294 | -0.6516 | 0.6851 | 0.9253 | -0.9632 | 0.6278 | 0.1677 | 0.9276 | 0.7165 0.4860 0.6454 -0.7382 -0.4711 0.9309 0.8516 0.7248 0.5028 -0.6236 -0.5034 0.9102 0.8541 -0.8767 0.9089 0.8573 0.6021 0.8659 0.8372 0.7179 0.5870 0.7782 0.8166 0.7928 0.6820 0.7012 -0.6269 0.1958 -0.6949 0.8114 0.2165 0.8423 0.8603 0.8372 0.8566 0.7628 0.7843 0.7268 0.7233 0.7754 0.8874 |
| Leaf homoserine | 0.8125 | 0.7800 | -0.8473 | 0.8726 | 0.8819 | -0.7658 | 0.9078 | -0.8691 | 0.7174 | 0.5923 | 0.8832 | -0.6129 | 0.4323 | 0.8483 | -0.7535 | 0.8974 | 0.8075 | -0.9121 | -0.8408 | 0.6984 | 0.8511 | 0.8173 | 0.5789 | 0.5874 | 0.4730 | 0.5991 | 0.5313 | 0.4712 | -0.6283 | 0.5908 | 0.8471 | 0.3552 | 0.3924 | 1.0000 | 0.8419 | 0.7704 | 0.6190 | 0.3232 | 0.8029 | 0.6357 | 0.8999 | 0.9303 | -0.8497 | -0.5200 | 0.6832 | 0.6959 | 0.7785 | 0.8484 | 0.2752 | 0.8090 | 0.4981 | 0.9000 | 0.9216 | 0.6935 | 0.0961 | 0.8991 | 0.4968 | -0.4509 | 0.9043 | -0.3408 | 0.7024 | 0.8999 0.9652 0.9059 -0.7067 -0.8713 0.6534 0.0972 0.6287 0.9040 -0.9056 -0.9560 0.7294 0.7855 -0.7682 0.7289 0.7062 0.8839 0.7554 0.6766 0.8660 0.9009 0.8335 0.6404 0.7213 0.9087 0.8920 -0.9225 0.8538 -0.5269 0.6688 0.8693 0.7579 0.7819 0.7674 0.6943 0.8460 0.7349 0.8412 0.8854 0.7499 0.7031 |
| Leaf L-cystathionine | 0.9356 | 0.9726 | -0.9674 | 0.6467 | 0.7028 | -0.9827 | 0.8896 | -0.9911 | 0.9534 | 0.9291 | 0.9759 | -0.3193 | 0.7718 | 0.9689 | -0.5142 | 0.9584 | 0.9787 | -0.9012 | -0.5542 | 0.9403 | 0.9012 | 0.9957 | 0.9199 | 0.9104 | 0.8447 | 0.8987 | 0.8305 | 0.8520 | -0.7168 | 0.8773 | 0.9910 | 0.8000 | 0.8037 | 0.8419 | 1.0000 | 0.9761 | 0.9417 | 0.7439 | 0.9890 | 0.4688 | 0.9717 | 0.7566 | -0.9909 | -0.8613 | 0.9650 | 0.9668 | 0.9819 | 0.9773 | 0.2576 | 0.7211 | 0.8533 | 0.9789 | 0.8131 | 0.9497 | -0.3758 | 0.9334 | 0.8403 | -0.8374 | 0.9245 | -0.1611 | 0.9539 | 0.9598 0.8785 0.9287 -0.8194 -0.7964 0.9221 0.6089 0.7576 0.8248 -0.9161 -0.9145 0.9773 0.9946 -0.9888 0.9772 0.9224 0.9091 0.9675 0.9390 0.9554 0.8948 0.9580 0.8533 0.8925 0.9652 0.9634 -0.9341 0.6307 -0.6102 0.9254 0.6577 0.9839 0.9690 0.9313 0.9143 0.9782 0.8986 0.9333 0.9529 0.9075 0.9388 |
| Leaf N6-acetyl-L-lysine | 0.9732 | 0.9457 | -0.9042 | 0.4834 | 0.5438 | -0.9985 | 0.8600 | -0.9654 | 0.9279 | 0.9621 | 0.9184 | -0.1464 | 0.8508 | 0.9305 | -0.3301 | 0.8852 | 0.9255 | -0.7916 | -0.3887 | 0.9302 | 0.7911 | 0.9614 | 0.9586 | 0.9496 | 0.8744 | 0.9484 | 0.8533 | 0.9108 | -0.7339 | 0.9502 | 0.9402 | 0.8555 | 0.8436 | 0.7704 | 0.9761 | 1.0000 | 0.9314 | 0.8113 | 0.9881 | 0.4791 | 0.9088 | 0.6301 | -0.9500 | -0.8765 | 0.9776 | 0.9448 | 0.9471 | 0.9104 | 0.4114 | 0.5567 | 0.9006 | 0.9305 | 0.6821 | 0.9355 | -0.3390 | 0.8501 | 0.9281 | -0.9119 | 0.8232 | 0.0525 | 0.9441 | 0.8891 0.7731 0.8327 -0.7204 -0.6551 0.8943 0.6839 0.8551 0.7005 -0.8134 -0.8752 0.9717 0.9731 -0.9898 0.9664 0.8564 0.7973 0.9968 0.9816 0.8709 0.7812 0.8856 0.7643 0.8017 0.8882 0.8841 -0.8405 0.4639 -0.5990 0.9729 0.4944 0.9504 0.9261 0.8720 0.8462 0.9115 0.8065 0.8421 0.8774 0.8144 0.8890 |
| Leaf trans-4-hydroxy-L-proline | 0.8386 | 0.9499 | -0.9125 | 0.4564 | 0.5325 | -0.9488 | 0.7425 | -0.9137 | 0.9637 | 0.9785 | 0.8972 | -0.1331 | 0.8387 | 0.9038 | -0.3432 | 0.8691 | 0.9501 | -0.7890 | -0.3363 | 0.9510 | 0.8321 | 0.9581 | 0.9722 | 0.9554 | 0.9420 | 0.9260 | 0.8884 | 0.9369 | -0.6599 | 0.8765 | 0.9384 | 0.9327 | 0.9296 | 0.6190 | 0.9417 | 0.9314 | 1.0000 | 0.8750 | 0.9434 | 0.2857 | 0.8818 | 0.5706 | -0.9338 | -0.9428 | 0.9819 | 0.9927 | 0.9665 | 0.9251 | 0.1372 | 0.6153 | 0.9290 | 0.8859 | 0.6614 | 0.9690 | -0.6452 | 0.8366 | 0.8857 | -0.9195 | 0.8247 | -0.1069 | 0.9671 | 0.8684 0.7150 0.8287 -0.8096 -0.6764 0.9625 0.8087 0.6722 0.6902 -0.8130 -0.7373 0.9787 0.9713 -0.9647 0.9813 0.9436 0.8188 0.9283 0.9267 0.8895 0.7890 0.9120 0.8970 0.9005 0.8701 0.8806 -0.8231 0.4494 -0.5855 0.9079 0.4770 0.9780 0.9504 0.9142 0.9408 0.9274 0.9007 0.8787 0.8707 0.9034 0.9624 |
| Leaf L-ornithine | 0.7623 | 0.8246 | -0.7130 | 0.0650 | 0.1478 | -0.8225 | 0.6210 | -0.7416 | 0.8613 | 0.9147 | 0.6888 | 0.3541 | 0.9751 | 0.7551 | 0.1073 | 0.6389 | 0.7557 | -0.4918 | 0.0914 | 0.8879 | 0.5625 | 0.7640 | 0.9312 | 0.9515 | 0.9799 | 0.9464 | 0.9514 | 0.9781 | -0.7554 | 0.8024 | 0.7148 | 0.9234 | 0.9896 | 0.3232 | 0.7439 | 0.8113 | 0.8750 | 1.0000 | 0.8149 | 0.3738 | 0.6578 | 0.2813 | -0.7440 | -0.9631 | 0.8903 | 0.8532 | 0.8071 | 0.6859 | 0.4035 | 0.2729 | 0.9815 | 0.6835 | 0.3445 | 0.8855 | -0.5908 | 0.6078 | 0.9360 | -0.9647 | 0.5344 | 0.3041 | 0.8861 | 0.6422 0.3944 0.5569 -0.6639 -0.3677 0.8816 0.8432 0.7626 0.4109 -0.5321 -0.4358 0.8648 0.7950 -0.8326 0.8603 0.7846 0.4996 0.8407 0.8078 0.6307 0.4897 0.7010 0.7355 0.7089 0.5977 0.6163 -0.5378 0.0857 -0.7011 0.7834 0.1053 0.7725 0.7995 0.7741 0.7830 0.6809 0.6986 0.6405 0.6471 0.6874 0.8223 |
| Leaf L-tyrosine-methyl-ester | 0.9648 | 0.9834 | -0.9556 | 0.5648 | 0.6245 | -0.9924 | 0.9074 | -0.9905 | 0.9685 | 0.9507 | 0.9630 | -0.1854 | 0.8530 | 0.9750 | -0.4034 | 0.9385 | 0.9667 | -0.8578 | -0.4566 | 0.9680 | 0.8641 | 0.9846 | 0.9487 | 0.9526 | 0.8946 | 0.9498 | 0.8921 | 0.9030 | -0.7936 | 0.8944 | 0.9710 | 0.8262 | 0.8564 | 0.8029 | 0.9890 | 0.9881 | 0.9434 | 0.8149 | 1.0000 | 0.5401 | 0.9543 | 0.7151 | -0.9830 | -0.9077 | 0.9822 | 0.9680 | 0.9807 | 0.9517 | 0.3693 | 0.6539 | 0.9097 | 0.9692 | 0.7621 | 0.9702 | -0.3563 | 0.9138 | 0.8956 | -0.8885 | 0.8833 | -0.0373 | 0.9758 | 0.9415 0.8294 0.8943 -0.8170 -0.7454 0.9410 0.6341 0.8331 0.7863 -0.8782 -0.8721 0.9893 0.9880 -0.9967 0.9867 0.9166 0.8565 0.9901 0.9460 0.9237 0.8500 0.9414 0.8423 0.8757 0.9311 0.9312 -0.8959 0.5573 -0.6871 0.9297 0.5827 0.9654 0.9708 0.9356 0.9083 0.9500 0.8790 0.9073 0.9346 0.8839 0.9398 |
| Leaf N-acetylaspartate | 0.6492 | 0.5538 | -0.5424 | 0.4242 | 0.4230 | -0.4654 | 0.8116 | -0.5790 | 0.5126 | 0.3314 | 0.5681 | 0.0525 | 0.5480 | 0.6274 | -0.1847 | 0.5702 | 0.4708 | -0.5149 | -0.3347 | 0.5583 | 0.4808 | 0.4538 | 0.3593 | 0.4628 | 0.4187 | 0.5292 | 0.5871 | 0.3802 | -0.8804 | 0.2744 | 0.4583 | 0.1038 | 0.3446 | 0.6357 | 0.4688 | 0.4791 | 0.2857 | 0.3738 | 0.5401 | 1.0000 | 0.5684 | 0.6532 | -0.5274 | -0.4656 | 0.4249 | 0.3809 | 0.4787 | 0.4524 | 0.7330 | 0.4019 | 0.4649 | 0.5955 | 0.5548 | 0.5042 | 0.4067 | 0.6015 | 0.4079 | -0.3418 | 0.4958 | 0.1862 | 0.5167 | 0.5794 0.5510 0.5326 -0.5580 -0.5143 0.4771 -0.1494 0.7401 0.5990 -0.5256 -0.4735 0.4669 0.4266 -0.4734 0.4566 0.4329 0.4174 0.5271 0.3222 0.4697 0.5003 0.5103 0.3844 0.4294 0.4926 0.4884 -0.5219 0.4706 -0.8514 0.2960 0.4596 0.3356 0.5211 0.5627 0.4235 0.4310 0.4264 0.4972 0.5718 0.4154 0.4422 |
| Leaf (5-L-glutamyl)-L-amino-acid | 0.8889 | 0.9683 | -0.9938 | 0.7801 | 0.8256 | -0.9204 | 0.9385 | -0.9846 | 0.9447 | 0.8382 | 0.9991 | -0.4185 | 0.6991 | 0.9867 | -0.6421 | 0.9984 | 0.9827 | -0.9715 | -0.6839 | 0.9273 | 0.9682 | 0.9763 | 0.8301 | 0.8398 | 0.7868 | 0.8310 | 0.8092 | 0.7581 | -0.7627 | 0.7425 | 0.9875 | 0.6684 | 0.7298 | 0.8999 | 0.9717 | 0.9088 | 0.8818 | 0.6578 | 0.9543 | 0.5684 | 1.0000 | 0.8828 | -0.9906 | -0.8223 | 0.9035 | 0.9301 | 0.9705 | 0.9896 | 0.2059 | 0.8464 | 0.7848 | 0.9975 | 0.9201 | 0.9300 | -0.3018 | 0.9915 | 0.7234 | -0.7236 | 0.9812 | -0.3027 | 0.9315 | 0.9989 0.9514 0.9866 -0.9045 -0.9097 0.9151 0.4422 0.6956 0.9332 -0.9804 -0.9059 0.9380 0.9590 -0.9415 0.9411 0.9419 0.9640 0.9055 0.8325 0.9896 0.9674 0.9890 0.8960 0.9377 0.9934 0.9939 -0.9865 0.7784 -0.6932 0.8110 0.7966 0.9455 0.9744 0.9647 0.9355 0.9865 0.9434 0.9823 0.9965 0.9497 0.9428 |
| Leaf glycyl-L-proline | 0.6637 | 0.7593 | -0.8536 | 0.9535 | 0.9610 | -0.6420 | 0.8770 | -0.8037 | 0.7143 | 0.4847 | 0.8663 | -0.6442 | 0.3621 | 0.8314 | -0.8421 | 0.9000 | 0.7978 | -0.9484 | -0.8955 | 0.6874 | 0.9147 | 0.7623 | 0.4747 | 0.5077 | 0.4532 | 0.5096 | 0.5399 | 0.3832 | -0.6560 | 0.3733 | 0.8074 | 0.2446 | 0.3681 | 0.9303 | 0.7566 | 0.6301 | 0.5706 | 0.2813 | 0.7151 | 0.6532 | 0.8828 | 1.0000 | -0.8106 | -0.5193 | 0.5991 | 0.6603 | 0.7529 | 0.8352 | 0.0900 | 0.9410 | 0.4440 | 0.8640 | 0.9874 | 0.6790 | 0.0058 | 0.9253 | 0.3406 | -0.3282 | 0.9279 | -0.5411 | 0.6786 | 0.8992 0.9666 0.9306 -0.8419 -0.9717 0.6758 -0.0199 0.4590 0.9840 -0.9387 -0.8136 0.6700 0.7149 -0.6766 0.6770 0.7592 0.9127 0.6268 0.4974 0.8781 0.9458 0.8545 0.7486 0.8068 0.8890 0.8854 -0.9300 0.9641 -0.6396 0.4730 0.9660 0.6971 0.7809 0.8128 0.7539 0.8274 0.8154 0.8856 0.8993 0.8218 0.7321 |
| Leaf trimethylamine-N-oxide | -0.9169 | -0.9889 | 0.9921 | -0.6926 | -0.7474 | 0.9610 | -0.9186 | 0.9948 | -0.9729 | -0.9049 | -0.9946 | 0.3255 | -0.7727 | -0.9908 | 0.5503 | -0.9838 | -0.9939 | 0.9350 | 0.5877 | -0.9600 | -0.9415 | -0.9955 | -0.8984 | -0.9026 | -0.8539 | -0.8917 | -0.8585 | -0.8371 | 0.7690 | -0.8154 | -0.9960 | -0.7626 | -0.8074 | -0.8497 | -0.9909 | -0.9500 | -0.9338 | -0.7440 | -0.9830 | -0.5274 | -0.9906 | -0.8106 | 1.0000 | 0.8792 | -0.9535 | -0.9685 | -0.9923 | -0.9906 | -0.2375 | -0.7808 | -0.8540 | -0.9932 | -0.8596 | -0.9651 | 0.3770 | -0.9684 | -0.8055 | 0.8094 | -0.9528 | 0.2144 | -0.9670 | -0.9847 -0.9037 -0.9595 0.8871 0.8517 -0.9479 -0.5564 -0.7386 -0.8782 0.9493 0.8891 -0.9762 -0.9876 0.9777 -0.9778 -0.9557 -0.9349 -0.9478 -0.8918 -0.9781 -0.9306 -0.9857 -0.9021 -0.9364 -0.9791 -0.9812 0.9588 -0.6887 0.6887 -0.8715 -0.7105 -0.9750 -0.9897 -0.9696 -0.9493 -0.9886 -0.9408 -0.9680 -0.9820 -0.9463 -0.9648 |
| Leaf N8-acetylspermidine | -0.8283 | -0.9374 | 0.8686 | -0.3268 | -0.4044 | 0.8935 | -0.7669 | 0.8677 | -0.9625 | -0.9420 | -0.8455 | -0.1193 | -0.9482 | -0.8909 | 0.1550 | -0.8127 | -0.8939 | 0.7017 | 0.1701 | -0.9742 | -0.7619 | -0.8863 | -0.9524 | -0.9761 | -0.9964 | -0.9658 | -0.9876 | -0.9678 | 0.8246 | -0.7962 | -0.8567 | -0.8939 | -0.9844 | -0.5200 | -0.8613 | -0.8765 | -0.9428 | -0.9631 | -0.9077 | -0.4656 | -0.8223 | -0.5193 | 0.8792 | 1.0000 | -0.9504 | -0.9446 | -0.9246 | -0.8426 | -0.3163 | -0.5205 | -0.9872 | -0.8355 | -0.5800 | -0.9730 | 0.5788 | -0.7925 | -0.9057 | 0.9374 | -0.7341 | -0.0735 | -0.9714 | -0.8143 -0.6113 -0.7529 0.8354 0.6039 -0.9752 -0.7588 -0.7569 -0.6379 0.7343 0.5903 -0.9486 -0.9003 0.9167 -0.9483 -0.9193 -0.7044 -0.9002 -0.8401 -0.8073 -0.7012 -0.8623 -0.8820 -0.8692 -0.7748 -0.7926 0.7339 -0.3491 0.7830 -0.8103 -0.3666 -0.8825 -0.9260 -0.9154 -0.9179 -0.8366 -0.8621 -0.8205 -0.8212 -0.8535 -0.9418 |
| Leaf glutathione-oxidized | 0.9237 | 0.9703 | -0.9219 | 0.4480 | 0.5192 | -0.9862 | 0.8227 | -0.9520 | 0.9721 | 0.9902 | 0.9185 | -0.0819 | 0.8938 | 0.9370 | -0.2992 | 0.8857 | 0.9504 | -0.7891 | -0.3285 | 0.9720 | 0.8167 | 0.9688 | 0.9890 | 0.9844 | 0.9487 | 0.9720 | 0.9187 | 0.9587 | -0.7498 | 0.9194 | 0.9452 | 0.9147 | 0.9277 | 0.6832 | 0.9650 | 0.9776 | 0.9819 | 0.8903 | 0.9822 | 0.4249 | 0.9035 | 0.5991 | -0.9535 | -0.9504 | 1.0000 | 0.9858 | 0.9733 | 0.9231 | 0.3144 | 0.5786 | 0.9552 | 0.9185 | 0.6673 | 0.9801 | -0.5026 | 0.8536 | 0.9368 | -0.9471 | 0.8234 | 0.0115 | 0.9832 | 0.8878 0.7358 0.8335 -0.7925 -0.6646 0.9586 0.7630 0.7983 0.6986 -0.8149 -0.7886 0.9952 0.9822 -0.9922 0.9934 0.9209 0.8023 0.9805 0.9598 0.8843 0.7840 0.9113 0.8530 0.8689 0.8789 0.8844 -0.8312 0.4414 -0.6513 0.9430 0.4689 0.9695 0.9582 0.9177 0.9148 0.9231 0.8696 0.8698 0.8844 0.8723 0.9480 |
| Leaf methionine-sulfoxide | 0.8715 | 0.9797 | -0.9543 | 0.5387 | 0.6094 | -0.9615 | 0.8138 | -0.9517 | 0.9858 | 0.9661 | 0.9426 | -0.1847 | 0.8367 | 0.9483 | -0.4108 | 0.9204 | 0.9795 | -0.8489 | -0.4174 | 0.9738 | 0.8837 | 0.9820 | 0.9608 | 0.9543 | 0.9346 | 0.9308 | 0.9030 | 0.9204 | -0.7196 | 0.8572 | 0.9687 | 0.8881 | 0.9108 | 0.6959 | 0.9668 | 0.9448 | 0.9927 | 0.8532 | 0.9680 | 0.3809 | 0.9301 | 0.6603 | -0.9685 | -0.9446 | 0.9858 | 1.0000 | 0.9903 | 0.9592 | 0.1720 | 0.6831 | 0.9239 | 0.9332 | 0.7376 | 0.9867 | -0.5732 | 0.8948 | 0.8699 | -0.8965 | 0.8786 | -0.1447 | 0.9854 | 0.9201 0.7842 0.8844 -0.8602 -0.7480 0.9792 0.7340 0.7022 0.7662 -0.8705 -0.7841 0.9924 0.9879 -0.9802 0.9950 0.9682 0.8676 0.9442 0.9186 0.9318 0.8479 0.9524 0.9221 0.9334 0.9159 0.9251 -0.8786 0.5359 -0.6486 0.8974 0.5609 0.9876 0.9807 0.9531 0.9646 0.9594 0.9341 0.9243 0.9220 0.9366 0.9830 |
| Leaf Asp-Phe | 0.8971 | 0.9953 | -0.9851 | 0.6275 | 0.6904 | -0.9620 | 0.8814 | -0.9805 | 0.9913 | 0.9356 | 0.9791 | -0.2507 | 0.8146 | 0.9820 | -0.4869 | 0.9638 | 0.9960 | -0.9042 | -0.5096 | 0.9796 | 0.9268 | 0.9940 | 0.9309 | 0.9343 | 0.9046 | 0.9179 | 0.8971 | 0.8827 | -0.7683 | 0.8258 | 0.9885 | 0.8209 | 0.8677 | 0.7785 | 0.9819 | 0.9471 | 0.9665 | 0.8071 | 0.9807 | 0.4787 | 0.9705 | 0.7529 | -0.9923 | -0.9246 | 0.9733 | 0.9903 | 1.0000 | 0.9829 | 0.2089 | 0.7485 | 0.8976 | 0.9727 | 0.8135 | 0.9870 | -0.4749 | 0.9459 | 0.8366 | -0.8533 | 0.9267 | -0.1870 | 0.9868 | 0.9641 0.8536 0.9341 -0.8969 -0.8169 0.9771 0.6352 0.7252 0.8397 -0.9224 -0.8331 0.9906 0.9914 -0.9825 0.9930 0.9760 0.9106 0.9481 0.8984 0.9651 0.9018 0.9814 0.9295 0.9510 0.9552 0.9620 -0.9290 0.6279 -0.6990 0.8756 0.6499 0.9835 0.9969 0.9777 0.9713 0.9810 0.9530 0.9597 0.9648 0.9559 0.9865 |
| Leaf Nα-acetyl-L-arginine | 0.8577 | 0.9696 | -0.9927 | 0.7527 | 0.8055 | -0.9267 | 0.8818 | -0.9738 | 0.9555 | 0.8704 | 0.9907 | -0.4237 | 0.6963 | 0.9723 | -0.6378 | 0.9878 | 0.9946 | -0.9618 | -0.6552 | 0.9310 | 0.9706 | 0.9880 | 0.8587 | 0.8548 | 0.8131 | 0.8335 | 0.8088 | 0.7845 | -0.7004 | 0.7647 | 0.9968 | 0.7316 | 0.7663 | 0.8484 | 0.9773 | 0.9104 | 0.9251 | 0.6859 | 0.9517 | 0.4524 | 0.9896 | 0.8352 | -0.9906 | -0.8426 | 0.9231 | 0.9592 | 0.9829 | 1.0000 | 0.1061 | 0.8428 | 0.8010 | 0.9827 | 0.8941 | 0.9424 | -0.4270 | 0.9751 | 0.7361 | -0.7515 | 0.9768 | -0.3408 | 0.9412 | 0.9867 0.9259 0.9780 -0.9033 -0.8940 0.9338 0.5321 0.6398 0.9058 -0.9717 -0.8836 0.9523 0.9754 -0.9503 0.9574 0.9648 0.9700 0.9018 0.8538 0.9954 0.9604 0.9933 0.9259 0.9585 0.9911 0.9945 -0.9771 0.7467 -0.6355 0.8329 0.7679 0.9756 0.9811 0.9659 0.9601 0.9996 0.9639 0.9863 0.9870 0.9709 0.9650 |
| Leaf N-glycyl-L-leucine | 0.5808 | 0.2831 | -0.1736 | -0.1684 | -0.1626 | -0.3720 | 0.4843 | -0.3271 | 0.2476 | 0.3078 | 0.2183 | 0.4982 | 0.5759 | 0.3098 | 0.3924 | 0.1733 | 0.1510 | -0.0311 | 0.2197 | 0.3244 | -0.0102 | 0.2056 | 0.3417 | 0.4092 | 0.3268 | 0.4907 | 0.4242 | 0.4029 | -0.6322 | 0.4232 | 0.1619 | 0.2022 | 0.3010 | 0.2752 | 0.2576 | 0.4114 | 0.1372 | 0.4035 | 0.3693 | 0.7330 | 0.2059 | 0.0900 | -0.2375 | -0.3163 | 0.3144 | 0.1720 | 0.2089 | 0.1061 | 1.0000 | -0.2211 | 0.4215 | 0.2705 | 0.0020 | 0.2745 | 0.4758 | 0.1624 | 0.5307 | -0.4310 | 0.0414 | 0.7729 | 0.2990 | 0.1879 0.0890 0.0809 -0.0438 0.0701 0.2042 0.0769 0.8196 0.0428 -0.0563 -0.2618 0.2884 0.2243 -0.3251 0.2643 0.0631 -0.0447 0.4550 0.3610 0.0652 0.0066 0.1201 -0.0522 -0.0136 0.1167 0.1012 -0.0872 -0.1557 -0.5066 0.3629 -0.1509 0.1223 0.2100 0.1807 0.0480 0.0929 -0.0172 0.0547 0.1634 -0.0240 0.1203 |
| Leaf γ-glutamate-cysteine | 0.5188 | 0.7310 | -0.8411 | 0.9566 | 0.9746 | -0.5846 | 0.7347 | -0.7405 | 0.7090 | 0.4778 | 0.8316 | -0.7191 | 0.2802 | 0.7772 | -0.9068 | 0.8721 | 0.8035 | -0.9461 | -0.8949 | 0.6605 | 0.9426 | 0.7499 | 0.4593 | 0.4702 | 0.4574 | 0.4414 | 0.4996 | 0.3612 | -0.5022 | 0.3093 | 0.8002 | 0.2949 | 0.3892 | 0.8090 | 0.7211 | 0.5567 | 0.6153 | 0.2729 | 0.6539 | 0.4019 | 0.8464 | 0.9410 | -0.7808 | -0.5205 | 0.5786 | 0.6831 | 0.7485 | 0.8428 | -0.2211 | 1.0000 | 0.4132 | 0.8089 | 0.9707 | 0.6699 | -0.2646 | 0.8957 | 0.2654 | -0.2961 | 0.9295 | -0.7397 | 0.6604 | 0.8664 0.9247 0.9216 -0.8762 -0.9877 0.6936 0.0822 0.2430 0.9644 -0.9343 -0.7198 0.6508 0.7035 -0.6368 0.6656 0.8075 0.9411 0.5434 0.4494 0.8897 0.9527 0.8601 0.8343 0.8688 0.8743 0.8802 -0.9147 0.9669 -0.5281 0.4213 0.9692 0.7205 0.7742 0.8128 0.8082 0.8393 0.8761 0.9038 0.8769 0.8821 0.7667 |
| Leaf Nα-acetyl-L-glutamine | 0.8628 | 0.9133 | -0.8246 | 0.2357 | 0.3136 | -0.9100 | 0.7504 | -0.8556 | 0.9342 | 0.9598 | 0.8098 | 0.1979 | 0.9798 | 0.8628 | -0.0551 | 0.7668 | 0.8564 | -0.6351 | -0.0866 | 0.9543 | 0.6875 | 0.8666 | 0.9720 | 0.9911 | 0.9936 | 0.9883 | 0.9776 | 0.9924 | -0.8148 | 0.8578 | 0.8274 | 0.9210 | 0.9865 | 0.4981 | 0.8533 | 0.9006 | 0.9290 | 0.9815 | 0.9097 | 0.4649 | 0.7848 | 0.4440 | -0.8540 | -0.9872 | 0.9552 | 0.9239 | 0.8976 | 0.8010 | 0.4215 | 0.4132 | 1.0000 | 0.8083 | 0.5012 | 0.9516 | -0.5258 | 0.7380 | 0.9592 | -0.9765 | 0.6728 | 0.2082 | 0.9540 | 0.7702 0.5565 0.6935 -0.7494 -0.5123 0.9398 0.7964 0.8242 0.5580 -0.6706 -0.5945 0.9408 0.8888 -0.9195 0.9365 0.8618 0.6374 0.9239 0.8798 0.7536 0.6309 0.8110 0.8032 0.7953 0.7322 0.7458 -0.6796 0.2501 -0.7436 0.8562 0.2715 0.8633 0.8908 0.8637 0.8580 0.7961 0.7888 0.7570 0.7717 0.7817 0.8959 |
| Leaf N-acetyl-L-tyrosine | 0.9190 | 0.9743 | -0.9894 | 0.7460 | 0.7924 | -0.9390 | 0.9515 | -0.9935 | 0.9491 | 0.8568 | 0.9979 | -0.3726 | 0.7335 | 0.9917 | -0.5967 | 0.9926 | 0.9795 | -0.9541 | -0.6485 | 0.9371 | 0.9485 | 0.9792 | 0.8507 | 0.8622 | 0.8032 | 0.8582 | 0.8273 | 0.7836 | -0.7868 | 0.7744 | 0.9859 | 0.6869 | 0.7470 | 0.9000 | 0.9789 | 0.9305 | 0.8859 | 0.6835 | 0.9692 | 0.5955 | 0.9975 | 0.8640 | -0.9932 | -0.8355 | 0.9185 | 0.9332 | 0.9727 | 0.9827 | 0.2705 | 0.8089 | 0.8083 | 1.0000 | 0.8975 | 0.9375 | -0.2765 | 0.9831 | 0.7599 | -0.7535 | 0.9657 | -0.2362 | 0.9408 | 0.9940 0.9376 0.9733 -0.8865 -0.8820 0.9169 0.4595 0.7443 0.9127 -0.9650 -0.9125 0.9485 0.9651 -0.9552 0.9497 0.9319 0.9437 0.9293 0.8564 0.9778 0.9482 0.9809 0.8769 0.9203 0.9855 0.9848 -0.9742 0.7435 -0.7062 0.8362 0.7628 0.9457 0.9747 0.9602 0.9244 0.9794 0.9258 0.9683 0.9897 0.9319 0.9380 |
| Leaf D-alanyl-D-alanine | 0.6800 | 0.8077 | -0.8994 | 0.9572 | 0.9749 | -0.6988 | 0.8624 | -0.8422 | 0.7709 | 0.5645 | 0.9056 | -0.6685 | 0.3958 | 0.8637 | -0.8638 | 0.9360 | 0.8578 | -0.9824 | -0.8947 | 0.7360 | 0.9601 | 0.8241 | 0.5501 | 0.5677 | 0.5197 | 0.5563 | 0.5773 | 0.4519 | -0.6238 | 0.4436 | 0.8664 | 0.3497 | 0.4430 | 0.9216 | 0.8131 | 0.6821 | 0.6614 | 0.3445 | 0.7621 | 0.5548 | 0.9201 | 0.9874 | -0.8596 | -0.5800 | 0.6673 | 0.7376 | 0.8135 | 0.8941 | 0.0020 | 0.9707 | 0.5012 | 0.8975 | 1.0000 | 0.7371 | -0.1382 | 0.9535 | 0.3964 | -0.3986 | 0.9687 | -0.5865 | 0.7344 | 0.9338 0.9846 0.9671 -0.8766 -0.9931 0.7380 0.1034 0.4407 0.9950 -0.9743 -0.8402 0.7329 0.7817 -0.7355 0.7418 0.8258 0.9636 0.6723 0.5690 0.9308 0.9821 0.9051 0.8176 0.8693 0.9348 0.9337 -0.9666 0.9617 -0.6046 0.5451 0.9682 0.7775 0.8344 0.8575 0.8217 0.8894 0.8781 0.9343 0.9357 0.8860 0.7992 |
| Leaf homo-Arg | 0.8909 | 0.9917 | -0.9570 | 0.5189 | 0.5880 | -0.9510 | 0.8613 | -0.9548 | 0.9985 | 0.9515 | 0.9446 | -0.0980 | 0.8887 | 0.9668 | -0.3579 | 0.9222 | 0.9718 | -0.8397 | -0.3818 | 0.9977 | 0.8770 | 0.9669 | 0.9538 | 0.9687 | 0.9583 | 0.9566 | 0.9552 | 0.9322 | -0.8202 | 0.8244 | 0.9516 | 0.8572 | 0.9294 | 0.6935 | 0.9497 | 0.9355 | 0.9690 | 0.8855 | 0.9702 | 0.5042 | 0.9300 | 0.6790 | -0.9651 | -0.9730 | 0.9801 | 0.9867 | 0.9870 | 0.9424 | 0.2745 | 0.6699 | 0.9516 | 0.9375 | 0.7371 | 1.0000 | -0.5083 | 0.9042 | 0.8800 | -0.9011 | 0.8658 | -0.0758 | 0.9995 | 0.9233 0.7734 0.8789 -0.8920 -0.7478 0.9944 0.6841 0.7622 0.7778 -0.8642 -0.7499 0.9909 0.9703 -0.9730 0.9919 0.9695 0.8418 0.9473 0.8870 0.9178 0.8377 0.9513 0.9254 0.9341 0.8992 0.9105 -0.8677 0.5297 -0.7615 0.8604 0.5496 0.9556 0.9870 0.9726 0.9659 0.9382 0.9322 0.9210 0.9263 0.9300 0.9852 |
| Leaf L-carnosine | -0.1348 | -0.4250 | 0.3882 | -0.0147 | -0.0910 | 0.3806 | -0.0217 | 0.3001 | -0.4995 | -0.5584 | -0.3250 | -0.0444 | -0.4011 | -0.3172 | 0.0619 | -0.3059 | -0.4640 | 0.2709 | -0.0812 | -0.4645 | -0.3812 | -0.4368 | -0.5465 | -0.4974 | -0.6018 | -0.4210 | -0.4664 | -0.5552 | 0.0614 | -0.3804 | -0.4155 | -0.7084 | -0.6516 | 0.0961 | -0.3758 | -0.3390 | -0.6452 | -0.5908 | -0.3563 | 0.4067 | -0.3018 | 0.0058 | 0.3770 | 0.5788 | -0.5026 | -0.5732 | -0.4749 | -0.4270 | 0.4758 | -0.2646 | -0.5258 | -0.2765 | -0.1382 | -0.5083 | 1.0000 | -0.2764 | -0.4172 | 0.5323 | -0.3100 | 0.2473 | -0.4886 | -0.2968 -0.1233 -0.2969 0.4398 0.2238 -0.5626 -0.7924 0.0329 -0.1672 0.2912 0.0606 -0.4830 -0.4625 0.4212 -0.4974 -0.5701 -0.3538 -0.3289 -0.4114 -0.3964 -0.2899 -0.4215 -0.6181 -0.5488 -0.3198 -0.3512 0.2745 -0.0182 0.1106 -0.3923 -0.0349 -0.5326 -0.4577 -0.4460 -0.5821 -0.4359 -0.5407 -0.4099 -0.3215 -0.5353 -0.5656 |
| Leaf S-(5-adenosy)-L-homocysteine | 0.8382 | 0.9473 | -0.9873 | 0.8279 | 0.8682 | -0.8653 | 0.9384 | -0.9573 | 0.9242 | 0.7750 | 0.9887 | -0.4527 | 0.6507 | 0.9746 | -0.6906 | 0.9969 | 0.9656 | -0.9861 | -0.7289 | 0.9047 | 0.9846 | 0.9448 | 0.7680 | 0.7882 | 0.7480 | 0.7796 | 0.7881 | 0.6977 | -0.7718 | 0.6510 | 0.9639 | 0.5916 | 0.6851 | 0.8991 | 0.9334 | 0.8501 | 0.8366 | 0.6078 | 0.9138 | 0.6015 | 0.9915 | 0.9253 | -0.9684 | -0.7925 | 0.8536 | 0.8948 | 0.9459 | 0.9751 | 0.1624 | 0.8957 | 0.7380 | 0.9831 | 0.9535 | 0.9042 | -0.2764 | 1.0000 | 0.6469 | -0.6520 | 0.9887 | -0.3749 | 0.9036 | 0.9966 0.9635 0.9948 -0.9377 -0.9497 0.8988 0.3545 0.6405 0.9687 -0.9921 -0.8699 0.8991 0.9197 -0.8957 0.9041 0.9388 0.9715 0.8504 0.7549 0.9867 0.9839 0.9861 0.9092 0.9486 0.9845 0.9874 -0.9904 0.8354 -0.7281 0.7288 0.8483 0.9062 0.9592 0.9662 0.9339 0.9699 0.9535 0.9892 0.9978 0.9575 0.9309 |
| Leaf argininosuccinic-acid | 0.8996 | 0.8477 | -0.7432 | 0.1393 | 0.2127 | -0.9241 | 0.7030 | -0.8270 | 0.8545 | 0.9608 | 0.7468 | 0.2204 | 0.9547 | 0.7969 | 0.0324 | 0.6916 | 0.7856 | -0.5431 | -0.0218 | 0.8798 | 0.5717 | 0.8297 | 0.9698 | 0.9689 | 0.9271 | 0.9739 | 0.8899 | 0.9786 | -0.7236 | 0.9510 | 0.7810 | 0.9311 | 0.9253 | 0.4968 | 0.8403 | 0.9281 | 0.8857 | 0.9360 | 0.8956 | 0.4079 | 0.7234 | 0.3406 | -0.8055 | -0.9057 | 0.9368 | 0.8699 | 0.8366 | 0.7361 | 0.5307 | 0.2654 | 0.9592 | 0.7599 | 0.3964 | 0.8800 | -0.4172 | 0.6469 | 1.0000 | -0.9908 | 0.5880 | 0.3554 | 0.8892 | 0.6973 0.4963 0.6064 -0.5771 -0.3808 0.8412 0.8251 0.8841 0.4385 -0.5787 -0.6425 0.9036 0.8628 -0.9080 0.8939 0.7442 0.5513 0.9413 0.9402 0.6723 0.5328 0.7193 0.6484 0.6570 0.6763 0.6790 -0.6064 0.1306 -0.5941 0.9312 0.1605 0.8318 0.8114 0.7510 0.7357 0.7356 0.6545 0.6512 0.6877 0.6543 0.7943 |
| Leaf succinic-acid | -0.8587 | -0.8586 | 0.7562 | -0.1383 | -0.2178 | 0.9149 | -0.6690 | 0.8178 | -0.8766 | -0.9747 | -0.7491 | -0.2192 | -0.9560 | -0.7968 | -0.0197 | -0.6957 | -0.8044 | 0.5524 | 0.0108 | -0.8961 | -0.5967 | -0.8382 | -0.9825 | -0.9787 | -0.9588 | -0.9732 | -0.9098 | -0.9948 | 0.7029 | -0.9311 | -0.7904 | -0.9656 | -0.9632 | -0.4509 | -0.8374 | -0.9119 | -0.9195 | -0.9647 | -0.8885 | -0.3418 | -0.7236 | -0.3282 | 0.8094 | 0.9374 | -0.9471 | -0.8965 | -0.8533 | -0.7515 | -0.4310 | -0.2961 | -0.9765 | -0.7535 | -0.3986 | -0.9011 | 0.5323 | -0.6520 | -0.9908 | 1.0000 | -0.5988 | -0.2924 | -0.9064 | -0.6998 -0.4852 -0.6151 0.6186 0.3982 -0.8749 -0.8746 -0.8231 -0.4444 0.5887 0.6016 -0.9152 -0.8717 0.9080 -0.9085 -0.7853 -0.5694 -0.9248 -0.9283 -0.6893 -0.5457 -0.7390 -0.7058 -0.7025 -0.6800 -0.6880 0.6103 -0.1340 0.5948 -0.9154 -0.1630 -0.8515 -0.8299 -0.7758 -0.7795 -0.7516 -0.6985 -0.6746 -0.6953 -0.6965 -0.8304 |
| Leaf 5-aminovaleric-acid | 0.7892 | 0.9146 | -0.9729 | 0.8740 | 0.9115 | -0.8405 | 0.8878 | -0.9340 | 0.8894 | 0.7463 | 0.9746 | -0.5647 | 0.5614 | 0.9421 | -0.7713 | 0.9879 | 0.9552 | -0.9980 | -0.7926 | 0.8576 | 0.9922 | 0.9358 | 0.7319 | 0.7356 | 0.6894 | 0.7167 | 0.7111 | 0.6419 | -0.6676 | 0.6314 | 0.9614 | 0.5697 | 0.6278 | 0.9043 | 0.9245 | 0.8232 | 0.8247 | 0.5344 | 0.8833 | 0.4958 | 0.9812 | 0.9279 | -0.9528 | -0.7341 | 0.8234 | 0.8786 | 0.9267 | 0.9768 | 0.0414 | 0.9295 | 0.6728 | 0.9657 | 0.9687 | 0.8658 | -0.3100 | 0.9887 | 0.5880 | -0.5988 | 1.0000 | -0.4802 | 0.8636 | 0.9860 0.9792 0.9988 -0.9111 -0.9659 0.8618 0.3453 0.5494 0.9713 -0.9991 -0.8878 0.8705 0.9087 -0.8711 0.8779 0.9234 0.9957 0.8122 0.7406 0.9919 0.9974 0.9768 0.8988 0.9414 0.9914 0.9926 -0.9986 0.8715 -0.6226 0.7182 0.8866 0.9090 0.9348 0.9365 0.9189 0.9749 0.9489 0.9876 0.9871 0.9569 0.9102 |
| Leaf α-aminoadipic-acid | 0.1482 | -0.1330 | 0.2988 | -0.7440 | -0.7291 | -0.0163 | -0.0973 | 0.1419 | -0.1242 | 0.0876 | -0.2768 | 0.8615 | 0.3768 | -0.1735 | 0.8703 | -0.3430 | -0.2702 | 0.5101 | 0.7715 | -0.0440 | -0.5074 | -0.2018 | 0.1223 | 0.1532 | 0.1290 | 0.2124 | 0.1377 | 0.2357 | -0.1792 | 0.2329 | -0.2740 | 0.1682 | 0.1677 | -0.3408 | -0.1611 | 0.0525 | -0.1069 | 0.3041 | -0.0373 | 0.1862 | -0.3027 | -0.5411 | 0.2144 | -0.0735 | 0.0115 | -0.1447 | -0.1870 | -0.3408 | 0.7729 | -0.7397 | 0.2082 | -0.2362 | -0.5865 | -0.0758 | 0.2473 | -0.3749 | 0.3554 | -0.2924 | -0.4802 | 1.0000 | -0.0566 | -0.3308 -0.4947 -0.4485 0.4051 0.6279 -0.1292 0.2338 0.4618 -0.5425 0.4772 0.2488 -0.0606 -0.1501 0.0415 -0.0845 -0.3090 -0.5412 0.0919 0.1037 -0.4113 -0.5276 -0.3425 -0.4006 -0.4135 -0.3841 -0.3908 0.4458 -0.7395 -0.0809 0.1176 -0.7327 -0.2192 -0.2075 -0.2579 -0.3175 -0.3463 -0.4234 -0.4243 -0.3491 -0.4341 -0.2401 |
| Leaf 2-aminobutyric-acid | 0.9039 | 0.9926 | -0.9561 | 0.5149 | 0.5834 | -0.9579 | 0.8693 | -0.9597 | 0.9974 | 0.9549 | 0.9460 | -0.0929 | 0.8946 | 0.9691 | -0.3505 | 0.9224 | 0.9705 | -0.8372 | -0.3794 | 0.9981 | 0.8716 | 0.9685 | 0.9575 | 0.9725 | 0.9573 | 0.9625 | 0.9554 | 0.9356 | -0.8263 | 0.8358 | 0.9524 | 0.8575 | 0.9276 | 0.7024 | 0.9539 | 0.9441 | 0.9671 | 0.8861 | 0.9758 | 0.5167 | 0.9315 | 0.6786 | -0.9670 | -0.9714 | 0.9832 | 0.9854 | 0.9868 | 0.9412 | 0.2990 | 0.6604 | 0.9540 | 0.9408 | 0.7344 | 0.9995 | -0.4886 | 0.9036 | 0.8892 | -0.9064 | 0.8636 | -0.0566 | 1.0000 | 0.9239 0.7748 0.8772 -0.8831 -0.7418 0.9909 0.6821 0.7800 0.7746 -0.8619 -0.7603 0.9929 0.9724 -0.9776 0.9932 0.9632 0.8379 0.9559 0.8958 0.9155 0.8344 0.9488 0.9147 0.9257 0.8997 0.9098 -0.8672 0.5245 -0.7617 0.8702 0.5449 0.9555 0.9860 0.9693 0.9590 0.9370 0.9241 0.9169 0.9257 0.9223 0.9806 |
| Leaf γ-aminobutyric-acid | 0.8713 | 0.9630 | -0.9937 | 0.7985 | 0.8424 | -0.9023 | 0.9393 | -0.9765 | 0.9399 | 0.8177 | 0.9974 | -0.4313 | 0.6833 | 0.9843 | -0.6609 | 0.9999 | 0.9790 | -0.9788 | -0.7008 | 0.9215 | 0.9766 | 0.9672 | 0.8100 | 0.8234 | 0.7755 | 0.8143 | 0.8039 | 0.7386 | -0.7669 | 0.7107 | 0.9812 | 0.6434 | 0.7165 | 0.8999 | 0.9598 | 0.8891 | 0.8684 | 0.6422 | 0.9415 | 0.5794 | 0.9989 | 0.8992 | -0.9847 | -0.8143 | 0.8878 | 0.9201 | 0.9641 | 0.9867 | 0.1879 | 0.8664 | 0.7702 | 0.9940 | 0.9338 | 0.9233 | -0.2968 | 0.9966 | 0.6973 | -0.6998 | 0.9860 | -0.3308 | 0.9239 | 1.0000 0.9571 0.9916 -0.9193 -0.9261 0.9121 0.4133 0.6755 0.9477 -0.9867 -0.8935 0.9263 0.9471 -0.9270 0.9301 0.9438 0.9690 0.8871 0.8061 0.9909 0.9754 0.9904 0.9042 0.9447 0.9921 0.9937 -0.9898 0.8002 -0.7071 0.7827 0.8165 0.9339 0.9714 0.9678 0.9380 0.9829 0.9501 0.9874 0.9991 0.9555 0.9415 |
| Leaf 4-acetamidobutyric-Acid | 0.7708 | 0.8450 | -0.9219 | 0.9273 | 0.9487 | -0.7830 | 0.8947 | -0.8972 | 0.8015 | 0.6447 | 0.9378 | -0.6512 | 0.4558 | 0.8966 | -0.8272 | 0.9579 | 0.8896 | -0.9855 | -0.8722 | 0.7699 | 0.9533 | 0.8767 | 0.6287 | 0.6345 | 0.5602 | 0.6263 | 0.6022 | 0.5232 | -0.6301 | 0.5697 | 0.9108 | 0.4322 | 0.4860 | 0.9652 | 0.8785 | 0.7731 | 0.7150 | 0.3944 | 0.8294 | 0.5510 | 0.9514 | 0.9666 | -0.9037 | -0.6113 | 0.7358 | 0.7842 | 0.8536 | 0.9259 | 0.0890 | 0.9247 | 0.5565 | 0.9376 | 0.9846 | 0.7734 | -0.1233 | 0.9635 | 0.4963 | -0.4852 | 0.9792 | -0.4947 | 0.7748 | 0.9571 1.0000 0.9772 -0.8384 -0.9632 0.7578 0.1871 0.5371 0.9746 -0.9799 -0.9219 0.7894 0.8423 -0.8056 0.7950 0.8301 0.9716 0.7582 0.6785 0.9495 0.9815 0.9211 0.7958 0.8569 0.9667 0.9602 -0.9840 0.9185 -0.5706 0.6615 0.9320 0.8341 0.8627 0.8635 0.8231 0.9238 0.8678 0.9382 0.9527 0.8795 0.8152 |
| Leaf 6-aminocaproic-acid | 0.8063 | 0.9264 | -0.9794 | 0.8628 | 0.9010 | -0.8493 | 0.9067 | -0.9432 | 0.9016 | 0.7553 | 0.9811 | -0.5316 | 0.5897 | 0.9543 | -0.7487 | 0.9930 | 0.9600 | -0.9969 | -0.7758 | 0.8736 | 0.9921 | 0.9400 | 0.7433 | 0.7525 | 0.7079 | 0.7371 | 0.7361 | 0.6592 | -0.7028 | 0.6374 | 0.9638 | 0.5753 | 0.6454 | 0.9059 | 0.9287 | 0.8327 | 0.8287 | 0.5569 | 0.8943 | 0.5326 | 0.9866 | 0.9306 | -0.9595 | -0.7529 | 0.8335 | 0.8844 | 0.9341 | 0.9780 | 0.0809 | 0.9216 | 0.6935 | 0.9733 | 0.9671 | 0.8789 | -0.2969 | 0.9948 | 0.6064 | -0.6151 | 0.9988 | -0.4485 | 0.8772 | 0.9916 0.9772 1.0000 -0.9216 -0.9637 0.8744 0.3458 0.5795 0.9735 -0.9994 -0.8844 0.8805 0.9133 -0.8798 0.8871 0.9295 0.9903 0.8253 0.7453 0.9922 0.9957 0.9816 0.9033 0.9452 0.9913 0.9931 -0.9984 0.8635 -0.6581 0.7217 0.8779 0.9089 0.9440 0.9476 0.9249 0.9750 0.9519 0.9902 0.9928 0.9587 0.9178 |
| Leaf creatine-phosphate | -0.6896 | -0.9053 | 0.9352 | -0.7291 | -0.7781 | 0.7498 | -0.8385 | 0.8536 | -0.9122 | -0.7170 | -0.9095 | 0.3013 | -0.6638 | -0.9129 | 0.5923 | -0.9219 | -0.9148 | 0.9103 | 0.5934 | -0.8954 | -0.9504 | -0.8603 | -0.7179 | -0.7587 | -0.7881 | -0.7411 | -0.8357 | -0.6879 | 0.7904 | -0.4965 | -0.8773 | -0.5761 | -0.7382 | -0.7067 | -0.8194 | -0.7204 | -0.8096 | -0.6639 | -0.8170 | -0.5580 | -0.9045 | -0.8419 | 0.8871 | 0.8354 | -0.7925 | -0.8602 | -0.8969 | -0.9033 | -0.0438 | -0.8762 | -0.7494 | -0.8865 | -0.8766 | -0.8920 | 0.4398 | -0.9377 | -0.5771 | 0.6186 | -0.9111 | 0.4051 | -0.8831 | -0.9193 -0.8384 -0.9216 1.0000 0.9105 -0.9202 -0.3699 -0.5149 -0.9159 0.9214 0.6473 -0.8437 -0.8343 0.8046 -0.8534 -0.9571 -0.8954 -0.7371 -0.6126 -0.9220 -0.9160 -0.9451 -0.9727 -0.9765 -0.8838 -0.9022 0.8985 -0.7653 0.8200 -0.5721 -0.7680 -0.8285 -0.9269 -0.9674 -0.9590 -0.8935 -0.9735 -0.9547 -0.9345 -0.9665 -0.9381 |
| Leaf kynurenic-acid | -0.6371 | -0.8089 | 0.9008 | -0.9458 | -0.9679 | 0.6778 | -0.8316 | 0.8238 | -0.7824 | -0.5638 | -0.8974 | 0.6500 | -0.3981 | -0.8564 | 0.8597 | -0.9297 | -0.8623 | 0.9791 | 0.8724 | -0.7446 | -0.9712 | -0.8179 | -0.5498 | -0.5689 | -0.5429 | -0.5505 | -0.5955 | -0.4590 | 0.6165 | -0.4101 | -0.8606 | -0.3654 | -0.4711 | -0.8713 | -0.7964 | -0.6551 | -0.6764 | -0.3677 | -0.7454 | -0.5143 | -0.9097 | -0.9717 | 0.8517 | 0.6039 | -0.6646 | -0.7480 | -0.8169 | -0.8940 | 0.0701 | -0.9877 | -0.5123 | -0.8820 | -0.9931 | -0.7478 | 0.2238 | -0.9497 | -0.3808 | 0.3982 | -0.9659 | 0.6279 | -0.7418 | -0.9261 -0.9632 -0.9637 0.9105 1.0000 -0.7606 -0.1311 -0.3894 -0.9936 0.9721 0.7875 -0.7321 -0.7753 0.7232 -0.7433 -0.8525 -0.9650 -0.6477 -0.5420 -0.9328 -0.9820 -0.9119 -0.8599 -0.9001 -0.9244 -0.9284 0.9578 -0.9569 0.6198 -0.5138 -0.9614 -0.7781 -0.8413 -0.8730 -0.8508 -0.8889 -0.9070 -0.9443 -0.9329 -0.9123 -0.8213 |
| Leaf N'-formylkynurenine | 0.8396 | 0.9807 | -0.9510 | 0.5227 | 0.5932 | -0.9151 | 0.8326 | -0.9283 | 0.9951 | 0.9280 | 0.9306 | -0.0976 | 0.8694 | 0.9521 | -0.3694 | 0.9122 | 0.9653 | -0.8375 | -0.3783 | 0.9911 | 0.8855 | 0.9487 | 0.9306 | 0.9487 | 0.9577 | 0.9313 | 0.9550 | 0.9143 | -0.8093 | 0.7704 | 0.9362 | 0.8427 | 0.9309 | 0.6534 | 0.9221 | 0.8943 | 0.9625 | 0.8816 | 0.9410 | 0.4771 | 0.9151 | 0.6758 | -0.9479 | -0.9752 | 0.9586 | 0.9792 | 0.9771 | 0.9338 | 0.2042 | 0.6936 | 0.9398 | 0.9169 | 0.7380 | 0.9944 | -0.5626 | 0.8988 | 0.8412 | -0.8749 | 0.8618 | -0.1292 | 0.9909 | 0.9121 0.7578 0.8744 -0.9202 -0.7606 1.0000 0.6768 0.7026 0.7826 -0.8617 -0.6988 0.9737 0.9497 -0.9459 0.9771 0.9827 0.8415 0.9082 0.8414 0.9142 0.8384 0.9504 0.9547 0.9546 0.8850 0.9005 -0.8584 0.5403 -0.7741 0.8108 0.5573 0.9408 0.9821 0.9783 0.9813 0.9288 0.9511 0.9255 0.9196 0.9466 0.9923 |
| Leaf 2-aminoethanesulfonic-acid | 0.5413 | 0.6061 | -0.4977 | -0.1129 | -0.0267 | -0.6963 | 0.2659 | -0.5360 | 0.6531 | 0.8418 | 0.4716 | 0.2633 | 0.7386 | 0.4988 | 0.1614 | 0.4139 | 0.5851 | -0.2867 | 0.2108 | 0.6534 | 0.3601 | 0.6230 | 0.8388 | 0.7886 | 0.8057 | 0.7490 | 0.6719 | 0.8540 | -0.3027 | 0.8062 | 0.5682 | 0.9588 | 0.8516 | 0.0972 | 0.6089 | 0.6839 | 0.8087 | 0.8432 | 0.6341 | -0.1494 | 0.4422 | -0.0199 | -0.5564 | -0.7588 | 0.7630 | 0.7340 | 0.6352 | 0.5321 | 0.0769 | 0.0822 | 0.7964 | 0.4595 | 0.1034 | 0.6841 | -0.7924 | 0.3545 | 0.8251 | -0.8746 | 0.3453 | 0.2338 | 0.6821 | 0.4133 0.1871 0.3458 -0.3699 -0.1311 0.6768 1.0000 0.4728 0.1361 -0.3215 -0.3318 0.7079 0.6729 -0.6892 0.7064 0.5995 0.3525 0.6784 0.7784 0.4581 0.2885 0.4972 0.5487 0.5139 0.4290 0.4430 -0.3417 -0.1338 -0.2187 0.7750 -0.1001 0.6974 0.5927 0.5218 0.5995 0.5417 0.5091 0.4356 0.4152 0.5096 0.6412 |
| Leaf 3,7-dimethyluric-Acid | 0.9349 | 0.7697 | -0.6805 | 0.2113 | 0.2543 | -0.8346 | 0.8257 | -0.7957 | 0.7429 | 0.7817 | 0.7100 | 0.2136 | 0.8816 | 0.7739 | 0.0191 | 0.6650 | 0.6789 | -0.5248 | -0.1123 | 0.7891 | 0.5083 | 0.7246 | 0.8009 | 0.8405 | 0.7589 | 0.8857 | 0.8076 | 0.8129 | -0.8530 | 0.8172 | 0.6868 | 0.6549 | 0.7248 | 0.6287 | 0.7576 | 0.8551 | 0.6722 | 0.7626 | 0.8331 | 0.7401 | 0.6956 | 0.4590 | -0.7386 | -0.7569 | 0.7983 | 0.7022 | 0.7252 | 0.6398 | 0.8196 | 0.2430 | 0.8242 | 0.7443 | 0.4407 | 0.7622 | 0.0329 | 0.6405 | 0.8841 | -0.8231 | 0.5494 | 0.4618 | 0.7800 | 0.6755 0.5371 0.5795 -0.5149 -0.3894 0.7026 0.4728 1.0000 0.4813 -0.5539 -0.6754 0.7830 0.7412 -0.8082 0.7668 0.5983 0.4826 0.8823 0.8070 0.5942 0.5056 0.6400 0.4829 0.5230 0.6276 0.6192 -0.5840 0.2133 -0.7111 0.7986 0.2329 0.6682 0.7174 0.6751 0.5844 0.6314 0.5224 0.5770 0.6569 0.5211 0.6488 |
| Leaf ethanolamine | 0.7022 | 0.8403 | -0.9204 | 0.9291 | 0.9516 | -0.7187 | 0.8871 | -0.8605 | 0.8095 | 0.5966 | 0.9221 | -0.5960 | 0.4612 | 0.8913 | -0.8177 | 0.9496 | 0.8789 | -0.9835 | -0.8502 | 0.7803 | 0.9707 | 0.8405 | 0.5864 | 0.6134 | 0.5778 | 0.6043 | 0.6421 | 0.5023 | -0.6882 | 0.4557 | 0.8785 | 0.3853 | 0.5028 | 0.9040 | 0.8248 | 0.7005 | 0.6902 | 0.4109 | 0.7863 | 0.5990 | 0.9332 | 0.9840 | -0.8782 | -0.6379 | 0.6986 | 0.7662 | 0.8397 | 0.9058 | 0.0428 | 0.9644 | 0.5580 | 0.9127 | 0.9950 | 0.7778 | -0.1672 | 0.9687 | 0.4385 | -0.4444 | 0.9713 | -0.5425 | 0.7746 | 0.9477 0.9746 0.9735 -0.9159 -0.9936 0.7826 0.1361 0.4813 1.0000 -0.9792 -0.8176 0.7635 0.7997 -0.7589 0.7721 0.8590 0.9605 0.6979 0.5810 0.9404 0.9837 0.9254 0.8536 0.8984 0.9379 0.9401 -0.9684 0.9418 -0.6775 0.5530 0.9467 0.7908 0.8645 0.8933 0.8557 0.8991 0.9049 0.9507 0.9518 0.9094 0.8340 |
| Leaf Cys | -0.7865 | -0.9138 | 0.9726 | -0.8783 | -0.9145 | 0.8309 | -0.8979 | 0.9311 | -0.8885 | -0.7339 | -0.9738 | 0.5544 | -0.5638 | -0.9441 | 0.7692 | -0.9885 | -0.9512 | 0.9989 | 0.7938 | -0.8585 | -0.9934 | -0.9285 | -0.7211 | -0.7304 | -0.6873 | -0.7140 | -0.7172 | -0.6347 | 0.6884 | -0.6122 | -0.9550 | -0.5516 | -0.6236 | -0.9056 | -0.9161 | -0.8134 | -0.8130 | -0.5321 | -0.8782 | -0.5256 | -0.9804 | -0.9387 | 0.9493 | 0.7343 | -0.8149 | -0.8705 | -0.9224 | -0.9717 | -0.0563 | -0.9343 | -0.6706 | -0.9650 | -0.9743 | -0.8642 | 0.2912 | -0.9921 | -0.5787 | 0.5887 | -0.9991 | 0.4772 | -0.8619 | -0.9867 -0.9799 -0.9994 0.9214 0.9721 -0.8617 -0.3215 -0.5539 -0.9792 1.0000 0.8777 -0.8645 -0.8997 0.8632 -0.8718 -0.9225 -0.9922 -0.8052 -0.7236 -0.9890 -0.9983 -0.9762 -0.9003 -0.9424 -0.9874 -0.9893 0.9976 -0.8795 0.6489 -0.6997 -0.8929 -0.8971 -0.9337 -0.9401 -0.9182 -0.9686 -0.9494 -0.9880 -0.9885 -0.9563 -0.9082 |
| Leaf creatine | -0.8739 | -0.8184 | 0.8580 | -0.7648 | -0.7899 | 0.8685 | -0.8574 | 0.9078 | -0.7630 | -0.7323 | -0.8944 | 0.5587 | -0.5145 | -0.8570 | 0.6654 | -0.8910 | -0.8486 | 0.8784 | 0.7370 | -0.7413 | -0.8249 | -0.8834 | -0.7138 | -0.6916 | -0.5646 | -0.6915 | -0.5678 | -0.6009 | 0.5561 | -0.7718 | -0.8974 | -0.5468 | -0.5034 | -0.9560 | -0.9145 | -0.8752 | -0.7373 | -0.4358 | -0.8721 | -0.4735 | -0.9059 | -0.8136 | 0.8891 | 0.5903 | -0.7886 | -0.7841 | -0.8331 | -0.8836 | -0.2618 | -0.7198 | -0.5945 | -0.9125 | -0.8402 | -0.7499 | 0.0606 | -0.8699 | -0.6425 | 0.6016 | -0.8878 | 0.2488 | -0.7603 | -0.8935 -0.9219 -0.8844 0.6473 0.7875 -0.6988 -0.3318 -0.6754 -0.8176 0.8777 1.0000 -0.8107 -0.8696 0.8569 -0.8085 -0.7323 -0.8768 -0.8491 -0.8323 -0.8767 -0.8663 -0.8429 -0.6441 -0.7210 -0.9229 -0.9045 0.9077 -0.7261 0.4135 -0.8332 -0.7536 -0.8543 -0.8149 -0.7660 -0.7193 -0.8875 -0.7359 -0.8330 -0.8751 -0.7554 -0.7441 |
| Leaf TFAADs | 0.9232 | 0.9888 | -0.9548 | 0.5237 | 0.5916 | -0.9826 | 0.8599 | -0.9716 | 0.9878 | 0.9726 | 0.9506 | -0.1428 | 0.8733 | 0.9647 | -0.3730 | 0.9248 | 0.9747 | -0.8414 | -0.4026 | 0.9844 | 0.8667 | 0.9844 | 0.9708 | 0.9721 | 0.9388 | 0.9597 | 0.9216 | 0.9349 | -0.7752 | 0.8844 | 0.9679 | 0.8776 | 0.9102 | 0.7294 | 0.9773 | 0.9717 | 0.9787 | 0.8648 | 0.9893 | 0.4669 | 0.9380 | 0.6700 | -0.9762 | -0.9486 | 0.9952 | 0.9924 | 0.9906 | 0.9523 | 0.2884 | 0.6508 | 0.9408 | 0.9485 | 0.7329 | 0.9909 | -0.4830 | 0.8991 | 0.9036 | -0.9152 | 0.8705 | -0.0606 | 0.9929 | 0.9263 0.7894 0.8805 -0.8437 -0.7321 0.9737 0.7079 0.7830 0.7635 -0.8645 -0.8107 1.0000 0.9910 -0.9946 0.9996 0.9491 0.8496 0.9752 0.9383 0.9221 0.8371 0.9462 0.8892 0.9077 0.9151 0.9212 -0.8766 0.5213 -0.6887 0.9184 0.5462 0.9781 0.9813 0.9507 0.9435 0.9509 0.9087 0.9123 0.9245 0.9110 0.9698 |
| Leaf TFAADs/N | 0.9173 | 0.9806 | -0.9660 | 0.6008 | 0.6636 | -0.9836 | 0.8601 | -0.9812 | 0.9716 | 0.9545 | 0.9667 | -0.2652 | 0.8040 | 0.9644 | -0.4709 | 0.9463 | 0.9844 | -0.8818 | -0.4971 | 0.9586 | 0.8957 | 0.9974 | 0.9465 | 0.9363 | 0.8874 | 0.9192 | 0.8632 | 0.8886 | -0.7158 | 0.8828 | 0.9884 | 0.8477 | 0.8541 | 0.7855 | 0.9946 | 0.9731 | 0.9713 | 0.7950 | 0.9880 | 0.4266 | 0.9590 | 0.7149 | -0.9876 | -0.9003 | 0.9822 | 0.9879 | 0.9914 | 0.9754 | 0.2243 | 0.7035 | 0.8888 | 0.9651 | 0.7817 | 0.9703 | -0.4625 | 0.9197 | 0.8628 | -0.8717 | 0.9087 | -0.1501 | 0.9724 | 0.9471 0.8423 0.9133 -0.8343 -0.7753 0.9497 0.6729 0.7412 0.7997 -0.8997 -0.8696 0.9910 1.0000 -0.9937 0.9918 0.9445 0.8956 0.9670 0.9432 0.9500 0.8775 0.9594 0.8828 0.9111 0.9500 0.9524 -0.9147 0.5887 -0.6202 0.9271 0.6157 0.9941 0.9784 0.9425 0.9381 0.9764 0.9152 0.9323 0.9433 0.9219 0.9612 |
| Leaf molar ratio of C/N in TFAADs | -0.9491 | -0.9793 | 0.9476 | -0.5358 | -0.5997 | 0.9959 | -0.8712 | 0.9806 | -0.9696 | -0.9696 | -0.9517 | 0.1765 | -0.8546 | -0.9614 | 0.3865 | -0.9247 | -0.9668 | 0.8418 | 0.4276 | -0.9662 | -0.8543 | -0.9867 | -0.9658 | -0.9616 | -0.9083 | -0.9527 | -0.8898 | -0.9199 | 0.7563 | -0.9126 | -0.9705 | -0.8643 | -0.8767 | -0.7682 | -0.9888 | -0.9898 | -0.9647 | -0.8326 | -0.9967 | -0.4734 | -0.9415 | -0.6766 | 0.9777 | 0.9167 | -0.9922 | -0.9802 | -0.9825 | -0.9503 | -0.3251 | -0.6368 | -0.9195 | -0.9552 | -0.7355 | -0.9730 | 0.4212 | -0.8957 | -0.9080 | 0.9080 | -0.8711 | 0.0415 | -0.9776 | -0.9270 -0.8056 -0.8798 0.8046 0.7232 -0.9459 -0.6892 -0.8082 -0.7589 0.8632 0.8569 -0.9946 -0.9937 1.0000 -0.9927 -0.9211 -0.8494 -0.9888 -0.9607 -0.9182 -0.8351 -0.9355 -0.8483 -0.8769 -0.9222 -0.9235 0.8815 -0.5251 0.6493 -0.9456 -0.5525 -0.9780 -0.9685 -0.9284 -0.9135 -0.9503 -0.8801 -0.8997 -0.9209 -0.8855 -0.9452 |
| Leaf TFAADs/C | 0.9141 | 0.9898 | -0.9590 | 0.5356 | 0.6036 | -0.9787 | 0.8563 | -0.9707 | 0.9899 | 0.9698 | 0.9533 | -0.1574 | 0.8648 | 0.9655 | -0.3886 | 0.9290 | 0.9789 | -0.8493 | -0.4141 | 0.9845 | 0.8762 | 0.9862 | 0.9673 | 0.9679 | 0.9375 | 0.9534 | 0.9192 | 0.9299 | -0.7687 | 0.8755 | 0.9711 | 0.8755 | 0.9089 | 0.7289 | 0.9772 | 0.9664 | 0.9813 | 0.8603 | 0.9867 | 0.4566 | 0.9411 | 0.6770 | -0.9778 | -0.9483 | 0.9934 | 0.9950 | 0.9930 | 0.9574 | 0.2643 | 0.6656 | 0.9365 | 0.9497 | 0.7418 | 0.9919 | -0.4974 | 0.9041 | 0.8939 | -0.9085 | 0.8779 | -0.0845 | 0.9932 | 0.9301 0.7950 0.8871 -0.8534 -0.7433 0.9771 0.7064 0.7668 0.7721 -0.8718 -0.8085 0.9996 0.9918 -0.9927 1.0000 0.9565 0.8588 0.9693 0.9324 0.9288 0.8457 0.9520 0.9001 0.9175 0.9199 0.9266 -0.8827 0.5336 -0.6865 0.9118 0.5582 0.9812 0.9844 0.9556 0.9513 0.9561 0.9185 0.9200 0.9292 0.9208 0.9753 |
| Leaf NR | 0.7889 | 0.9696 | -0.9731 | 0.6500 | 0.7140 | -0.8821 | 0.8275 | -0.9251 | 0.9794 | 0.8762 | 0.9513 | -0.2647 | 0.7635 | 0.9519 | -0.5251 | 0.9460 | 0.9805 | -0.9058 | -0.5187 | 0.9607 | 0.9483 | 0.9536 | 0.8719 | 0.8832 | 0.8917 | 0.8566 | 0.8888 | 0.8327 | -0.7465 | 0.7034 | 0.9555 | 0.7747 | 0.8573 | 0.7062 | 0.9224 | 0.8564 | 0.9436 | 0.7846 | 0.9166 | 0.4329 | 0.9419 | 0.7592 | -0.9557 | -0.9193 | 0.9209 | 0.9682 | 0.9760 | 0.9648 | 0.0631 | 0.8075 | 0.8618 | 0.9319 | 0.8258 | 0.9695 | -0.5701 | 0.9388 | 0.7442 | -0.7853 | 0.9234 | -0.3090 | 0.9632 | 0.9438 0.8301 0.9295 -0.9571 -0.8525 0.9827 0.5995 0.5983 0.8590 -0.9225 -0.7323 0.9491 0.9445 -0.9211 0.9565 1.0000 0.9146 0.8617 0.7954 0.9592 0.9094 0.9798 0.9874 0.9923 0.9286 0.9437 -0.9147 0.6648 -0.7269 0.7641 0.6802 0.9477 0.9841 0.9884 0.9998 0.9612 0.9913 0.9710 0.9536 0.9896 0.9969 |
| Leaf GOGAT | 0.7459 | 0.8901 | -0.9569 | 0.8844 | 0.9218 | -0.8171 | 0.8421 | -0.9091 | 0.8670 | 0.7284 | 0.9562 | -0.6137 | 0.5111 | 0.9141 | -0.8042 | 0.9721 | 0.9435 | -0.9934 | -0.8106 | 0.8279 | 0.9894 | 0.9230 | 0.7101 | 0.7041 | 0.6606 | 0.6775 | 0.6688 | 0.6119 | -0.6005 | 0.6121 | 0.9515 | 0.5633 | 0.6021 | 0.8839 | 0.9091 | 0.7973 | 0.8188 | 0.4996 | 0.8565 | 0.4174 | 0.9640 | 0.9127 | -0.9349 | -0.7044 | 0.8023 | 0.8676 | 0.9106 | 0.9700 | -0.0447 | 0.9411 | 0.6374 | 0.9437 | 0.9636 | 0.8418 | -0.3538 | 0.9715 | 0.5513 | -0.5694 | 0.9957 | -0.5412 | 0.8379 | 0.9690 0.9716 0.9903 -0.8954 -0.9650 0.8415 0.3525 0.4826 0.9605 -0.9922 -0.8768 0.8496 0.8956 -0.8494 0.8588 0.9146 1.0000 0.7806 0.7240 0.9866 0.9945 0.9650 0.8964 0.9372 0.9838 0.9854 -0.9911 0.8775 -0.5598 0.7031 0.8937 0.9056 0.9160 0.9159 0.9111 0.9700 0.9455 0.9802 0.9712 0.9552 0.8979 |
| Leaf GOT | 0.9799 | 0.9537 | -0.9048 | 0.4603 | 0.5217 | -0.9954 | 0.8754 | -0.9651 | 0.9386 | 0.9632 | 0.9170 | -0.0899 | 0.8852 | 0.9383 | -0.2917 | 0.8827 | 0.9232 | -0.7812 | -0.3555 | 0.9465 | 0.7852 | 0.9552 | 0.9636 | 0.9644 | 0.8971 | 0.9674 | 0.8866 | 0.9273 | -0.7815 | 0.9393 | 0.9316 | 0.8548 | 0.8659 | 0.7554 | 0.9675 | 0.9968 | 0.9283 | 0.8407 | 0.9901 | 0.5271 | 0.9055 | 0.6268 | -0.9478 | -0.9002 | 0.9805 | 0.9442 | 0.9481 | 0.9018 | 0.4550 | 0.5434 | 0.9239 | 0.9293 | 0.6723 | 0.9473 | -0.3289 | 0.8504 | 0.9413 | -0.9248 | 0.8122 | 0.0919 | 0.9559 | 0.8871 0.7582 0.8253 -0.7371 -0.6477 0.9082 0.6784 0.8823 0.6979 -0.8052 -0.8491 0.9752 0.9670 -0.9888 0.9693 0.8617 0.7806 1.0000 0.9702 0.8623 0.7708 0.8848 0.7716 0.8053 0.8762 0.8740 -0.8298 0.4479 -0.6540 0.9585 0.4762 0.9384 0.9314 0.8833 0.8519 0.9008 0.8083 0.8392 0.8765 0.8136 0.8952 |
| Leaf GPT | 0.9273 | 0.8853 | -0.8304 | 0.3729 | 0.4380 | -0.9786 | 0.7482 | -0.9055 | 0.8731 | 0.9688 | 0.8443 | -0.1073 | 0.8235 | 0.8521 | -0.2487 | 0.8026 | 0.8702 | -0.7003 | -0.2914 | 0.8726 | 0.7036 | 0.9206 | 0.9616 | 0.9313 | 0.8524 | 0.9219 | 0.7951 | 0.9124 | -0.6129 | 0.9883 | 0.8911 | 0.9057 | 0.8372 | 0.6766 | 0.9390 | 0.9816 | 0.9267 | 0.8078 | 0.9460 | 0.3222 | 0.8325 | 0.4974 | -0.8918 | -0.8401 | 0.9598 | 0.9186 | 0.8984 | 0.8538 | 0.3610 | 0.4494 | 0.8798 | 0.8564 | 0.5690 | 0.8870 | -0.4114 | 0.7549 | 0.9402 | -0.9283 | 0.7406 | 0.1037 | 0.8958 | 0.8061 0.6785 0.7453 -0.6126 -0.5420 0.8414 0.7784 0.8070 0.5810 -0.7236 -0.8323 0.9383 0.9432 -0.9607 0.9324 0.7954 0.7240 0.9702 1.0000 0.8016 0.6901 0.8126 0.6958 0.7282 0.8206 0.8152 -0.7577 0.3405 -0.4607 0.9985 0.3767 0.9314 0.8641 0.7907 0.7852 0.8593 0.7338 0.7615 0.7924 0.7445 0.8349 |
| Leaf GS | 0.8234 | 0.9524 | -0.9907 | 0.8070 | 0.8545 | -0.8891 | 0.8845 | -0.9581 | 0.9358 | 0.8206 | 0.9879 | -0.4801 | 0.6443 | 0.9641 | -0.6972 | 0.9926 | 0.9843 | -0.9825 | -0.7127 | 0.9077 | 0.9886 | 0.9692 | 0.8081 | 0.8088 | 0.7706 | 0.7872 | 0.7776 | 0.7290 | -0.6929 | 0.7015 | 0.9854 | 0.6676 | 0.7179 | 0.8660 | 0.9554 | 0.8709 | 0.8895 | 0.6307 | 0.9237 | 0.4697 | 0.9896 | 0.8781 | -0.9781 | -0.8073 | 0.8843 | 0.9318 | 0.9651 | 0.9954 | 0.0652 | 0.8897 | 0.7536 | 0.9778 | 0.9308 | 0.9178 | -0.3964 | 0.9867 | 0.6723 | -0.6893 | 0.9919 | -0.4113 | 0.9155 | 0.9909 0.9495 0.9922 -0.9220 -0.9328 0.9142 0.4581 0.5942 0.9404 -0.9890 -0.8767 0.9221 0.9500 -0.9182 0.9288 0.9592 0.9866 0.8623 0.8016 1.0000 0.9822 0.9939 0.9302 0.9645 0.9942 0.9980 -0.9901 0.8047 -0.6423 0.7787 0.8227 0.9513 0.9689 0.9636 0.9552 0.9940 0.9703 0.9954 0.9927 0.9770 0.9524 |
| Leaf glucose | 0.7506 | 0.8903 | -0.9588 | 0.9008 | 0.9343 | -0.8002 | 0.8761 | -0.9088 | 0.8646 | 0.7000 | 0.9591 | -0.5954 | 0.5171 | 0.9234 | -0.8032 | 0.9779 | 0.9355 | -0.9996 | -0.8216 | 0.8305 | 0.9931 | 0.9091 | 0.6854 | 0.6927 | 0.6517 | 0.6735 | 0.6814 | 0.5939 | -0.6543 | 0.5738 | 0.9398 | 0.5166 | 0.5870 | 0.9009 | 0.8948 | 0.7812 | 0.7890 | 0.4897 | 0.8500 | 0.5003 | 0.9674 | 0.9458 | -0.9306 | -0.7012 | 0.7840 | 0.8479 | 0.9018 | 0.9604 | 0.0066 | 0.9527 | 0.6309 | 0.9482 | 0.9821 | 0.8377 | -0.2899 | 0.9839 | 0.5328 | -0.5457 | 0.9974 | -0.5276 | 0.8344 | 0.9754 0.9815 0.9957 -0.9160 -0.9820 0.8384 0.2885 0.5056 0.9837 -0.9983 -0.8663 0.8371 0.8775 -0.8351 0.8457 0.9094 0.9945 0.7708 0.6901 0.9822 1.0000 0.9649 0.8937 0.9360 0.9795 0.9815 -0.9940 0.9017 -0.6223 0.6661 0.9141 0.8789 0.9143 0.9241 0.9058 0.9577 0.9434 0.9820 0.9782 0.9509 0.8910 |
| Leaf fructose | 0.8432 | 0.9759 | -0.9984 | 0.7554 | 0.8080 | -0.9045 | 0.8991 | -0.9668 | 0.9659 | 0.8511 | 0.9915 | -0.3852 | 0.7142 | 0.9811 | -0.6264 | 0.9915 | 0.9934 | -0.9652 | -0.6448 | 0.9452 | 0.9810 | 0.9754 | 0.8435 | 0.8536 | 0.8276 | 0.8354 | 0.8403 | 0.7814 | -0.7541 | 0.7164 | 0.9855 | 0.7058 | 0.7782 | 0.8335 | 0.9580 | 0.8856 | 0.9120 | 0.7010 | 0.9414 | 0.5103 | 0.9890 | 0.8545 | -0.9857 | -0.8623 | 0.9113 | 0.9524 | 0.9814 | 0.9933 | 0.1201 | 0.8601 | 0.8110 | 0.9809 | 0.9051 | 0.9513 | -0.4215 | 0.9861 | 0.7193 | -0.7390 | 0.9768 | -0.3425 | 0.9488 | 0.9904 0.9211 0.9816 -0.9451 -0.9119 0.9504 0.4972 0.6400 0.9254 -0.9762 -0.8429 0.9462 0.9594 -0.9355 0.9520 0.9798 0.9650 0.8848 0.8126 0.9939 0.9649 1.0000 0.9515 0.9782 0.9821 0.9891 -0.9750 0.7610 -0.7098 0.7862 0.7776 0.9550 0.9882 0.9870 0.9763 0.9900 0.9814 0.9956 0.9939 0.9846 0.9763 |
| Leaf sucrose | 0.6857 | 0.9211 | -0.9372 | 0.6580 | 0.7202 | -0.7971 | 0.7648 | -0.8585 | 0.9397 | 0.8035 | 0.9051 | -0.2826 | 0.6947 | 0.9020 | -0.5505 | 0.9082 | 0.9413 | -0.8859 | -0.5229 | 0.9153 | 0.9401 | 0.8971 | 0.7990 | 0.8146 | 0.8491 | 0.7809 | 0.8494 | 0.7649 | -0.7028 | 0.5912 | 0.9054 | 0.7124 | 0.8166 | 0.6404 | 0.8533 | 0.7643 | 0.8970 | 0.7355 | 0.8423 | 0.3844 | 0.8960 | 0.7486 | -0.9021 | -0.8820 | 0.8530 | 0.9221 | 0.9295 | 0.9259 | -0.0522 | 0.8343 | 0.8032 | 0.8769 | 0.8176 | 0.9254 | -0.6181 | 0.9092 | 0.6484 | -0.7058 | 0.8988 | -0.4006 | 0.9147 | 0.9042 0.7958 0.9033 -0.9727 -0.8599 0.9547 0.5487 0.4829 0.8536 -0.9003 -0.6441 0.8892 0.8828 -0.8483 0.9001 0.9874 0.8964 0.7716 0.6958 0.9302 0.8937 0.9515 1.0000 0.9938 0.8861 0.9065 -0.8824 0.6820 -0.7193 0.6595 0.6923 0.8947 0.9454 0.9665 0.9904 0.9213 0.9911 0.9534 0.9201 0.9866 0.9739 |
| Leaf total soluble sugars | 0.7348 | 0.9426 | -0.9671 | 0.7193 | 0.7769 | -0.8307 | 0.8172 | -0.8996 | 0.9504 | 0.8120 | 0.9434 | -0.3478 | 0.6864 | 0.9346 | -0.6073 | 0.9481 | 0.9659 | -0.9305 | -0.5938 | 0.9250 | 0.9705 | 0.9282 | 0.8055 | 0.8193 | 0.8332 | 0.7898 | 0.8397 | 0.7581 | -0.7176 | 0.6219 | 0.9399 | 0.6971 | 0.7928 | 0.7213 | 0.8925 | 0.8017 | 0.9005 | 0.7089 | 0.8757 | 0.4294 | 0.9377 | 0.8068 | -0.9364 | -0.8692 | 0.8689 | 0.9334 | 0.9510 | 0.9585 | -0.0136 | 0.8688 | 0.7953 | 0.9203 | 0.8693 | 0.9341 | -0.5488 | 0.9486 | 0.6570 | -0.7025 | 0.9414 | -0.4135 | 0.9257 | 0.9447 0.8569 0.9452 -0.9765 -0.9001 0.9546 0.5139 0.5230 0.8984 -0.9424 -0.7210 0.9077 0.9111 -0.8769 0.9175 0.9923 0.9372 0.8053 0.7282 0.9645 0.9360 0.9782 0.9938 1.0000 0.9311 0.9469 -0.9291 0.7372 -0.7168 0.6948 0.7491 0.9188 0.9650 0.9809 0.9932 0.9543 0.9997 0.9810 0.9569 0.9981 0.9797 |
| Leaf starch | 0.8530 | 0.9430 | -0.9832 | 0.8195 | 0.8623 | -0.9012 | 0.9029 | -0.9678 | 0.9168 | 0.8138 | 0.9893 | -0.5081 | 0.6286 | 0.9626 | -0.7075 | 0.9929 | 0.9744 | -0.9826 | -0.7375 | 0.8901 | 0.9748 | 0.9692 | 0.8001 | 0.7975 | 0.7388 | 0.7813 | 0.7483 | 0.7123 | -0.6843 | 0.7263 | 0.9858 | 0.6472 | 0.6820 | 0.9087 | 0.9652 | 0.8882 | 0.8701 | 0.5977 | 0.9311 | 0.4926 | 0.9934 | 0.8890 | -0.9791 | -0.7748 | 0.8789 | 0.9159 | 0.9552 | 0.9911 | 0.1167 | 0.8743 | 0.7322 | 0.9855 | 0.9348 | 0.8992 | -0.3198 | 0.9845 | 0.6763 | -0.6800 | 0.9914 | -0.3841 | 0.8997 | 0.9921 0.9667 0.9913 -0.8838 -0.9244 0.8850 0.4290 0.6276 0.9379 -0.9874 -0.9229 0.9151 0.9500 -0.9222 0.9199 0.9286 0.9838 0.8762 0.8206 0.9942 0.9795 0.9821 0.8861 0.9311 1.0000 0.9989 -0.9945 0.8100 -0.6133 0.8021 0.8299 0.9465 0.9553 0.9416 0.9224 0.9905 0.9390 0.9815 0.9896 0.9484 0.9254 |
| Leaf TNC | 0.8446 | 0.9501 | -0.9887 | 0.8131 | 0.8581 | -0.8991 | 0.8989 | -0.9665 | 0.9281 | 0.8198 | 0.9911 | -0.4917 | 0.6407 | 0.9664 | -0.7002 | 0.9948 | 0.9808 | -0.9835 | -0.7250 | 0.9013 | 0.9817 | 0.9714 | 0.8069 | 0.8064 | 0.7564 | 0.7883 | 0.7655 | 0.7236 | -0.6938 | 0.7186 | 0.9876 | 0.6584 | 0.7012 | 0.8920 | 0.9634 | 0.8841 | 0.8806 | 0.6163 | 0.9312 | 0.4884 | 0.9939 | 0.8854 | -0.9812 | -0.7926 | 0.8844 | 0.9251 | 0.9620 | 0.9945 | 0.1012 | 0.8802 | 0.7458 | 0.9848 | 0.9337 | 0.9105 | -0.3512 | 0.9874 | 0.6790 | -0.6880 | 0.9926 | -0.3908 | 0.9098 | 0.9937 0.9602 0.9931 -0.9022 -0.9284 0.9005 0.4430 0.6192 0.9401 -0.9893 -0.9045 0.9212 0.9524 -0.9235 0.9266 0.9437 0.9854 0.8740 0.8152 0.9980 0.9815 0.9891 0.9065 0.9469 0.9989 1.0000 -0.9939 0.8070 -0.6311 0.7947 0.8261 0.9502 0.9638 0.9537 0.9384 0.9935 0.9538 0.9890 0.9930 0.9619 0.9393 |
| Leaf sucrose/starch | -0.8148 | -0.9191 | 0.9740 | -0.8688 | -0.9053 | 0.8550 | -0.9054 | 0.9453 | -0.8902 | -0.7541 | -0.9796 | 0.5550 | -0.5747 | -0.9492 | 0.7590 | -0.9910 | -0.9554 | 0.9962 | 0.7896 | -0.8612 | -0.9852 | -0.9410 | -0.7404 | -0.7448 | -0.6901 | -0.7300 | -0.7147 | -0.6499 | 0.6824 | -0.6527 | -0.9650 | -0.5714 | -0.6269 | -0.9225 | -0.9341 | -0.8405 | -0.8231 | -0.5378 | -0.8959 | -0.5219 | -0.9865 | -0.9300 | 0.9588 | 0.7339 | -0.8312 | -0.8786 | -0.9290 | -0.9771 | -0.0872 | -0.9147 | -0.6796 | -0.9742 | -0.9666 | -0.8677 | 0.2745 | -0.9904 | -0.6064 | 0.6103 | -0.9986 | 0.4458 | -0.8672 | -0.9898 -0.9840 -0.9984 0.8985 0.9578 -0.8584 -0.3417 -0.5840 -0.9684 0.9976 0.9077 -0.8766 -0.9147 0.8815 -0.8827 -0.9147 -0.9911 -0.8298 -0.7577 -0.9901 -0.9940 -0.9750 -0.8824 -0.9291 -0.9945 -0.9939 1.0000 -0.8643 0.6271 -0.7368 -0.8803 -0.9106 -0.9357 -0.9336 -0.9092 -0.9751 -0.9371 -0.9829 -0.9888 -0.9458 -0.9046 |
| Leaf malate | 0.4697 | 0.6171 | -0.7481 | 0.9949 | 0.9915 | -0.4824 | 0.7206 | -0.6644 | 0.5737 | 0.3240 | 0.7553 | -0.7985 | 0.1365 | 0.6932 | -0.9475 | 0.8043 | 0.6912 | -0.8992 | -0.9657 | 0.5278 | 0.8642 | 0.6440 | 0.3048 | 0.3217 | 0.2782 | 0.3079 | 0.3493 | 0.1933 | -0.4481 | 0.2031 | 0.7043 | 0.1001 | 0.1958 | 0.8538 | 0.6307 | 0.4639 | 0.4494 | 0.0857 | 0.5573 | 0.4706 | 0.7784 | 0.9641 | -0.6887 | -0.3491 | 0.4414 | 0.5359 | 0.6279 | 0.7467 | -0.1557 | 0.9669 | 0.2501 | 0.7435 | 0.9617 | 0.5297 | -0.0182 | 0.8354 | 0.1306 | -0.1340 | 0.8715 | -0.7395 | 0.5245 | 0.8002 0.9185 0.8635 -0.7653 -0.9569 0.5403 -0.1338 0.2133 0.9418 -0.8795 -0.7261 0.5213 0.5887 -0.5251 0.5336 0.6648 0.8775 0.4479 0.3405 0.8047 0.9017 0.7610 0.6820 0.7372 0.8100 0.8070 -0.8643 1.0000 -0.4614 0.3186 0.9989 0.5937 0.6565 0.6968 0.6626 0.7423 0.7480 0.8126 0.8043 0.7574 0.6217 |
| Leaf citrate | -0.6766 | -0.7597 | 0.7233 | -0.3961 | -0.4348 | 0.6105 | -0.8176 | 0.6974 | -0.7657 | -0.5807 | -0.7080 | -0.1639 | -0.7771 | -0.7774 | 0.1629 | -0.7026 | -0.6835 | 0.6253 | 0.2381 | -0.8004 | -0.6611 | -0.6369 | -0.6092 | -0.7069 | -0.7433 | -0.7382 | -0.8629 | -0.6572 | 0.9723 | -0.3913 | -0.6298 | -0.4315 | -0.6949 | -0.5269 | -0.6102 | -0.5990 | -0.5855 | -0.7011 | -0.6871 | -0.8514 | -0.6932 | -0.6396 | 0.6887 | 0.7830 | -0.6513 | -0.6486 | -0.6990 | -0.6355 | -0.5066 | -0.5281 | -0.7436 | -0.7062 | -0.6046 | -0.7615 | 0.1106 | -0.7281 | -0.5941 | 0.5948 | -0.6226 | -0.0809 | -0.7617 | -0.7071 -0.5706 -0.6581 0.8200 0.6198 -0.7741 -0.2187 -0.7111 -0.6775 0.6489 0.4135 -0.6887 -0.6202 0.6493 -0.6865 -0.7269 -0.5598 -0.6540 -0.4607 -0.6423 -0.6223 -0.7098 -0.7193 -0.7168 -0.6133 -0.6311 0.6271 -0.4614 1.0000 -0.4171 -0.4524 -0.5592 -0.7442 -0.7990 -0.7257 -0.6146 -0.7057 -0.6926 -0.7173 -0.6859 -0.7300 |
| Leaf isocitrate | 0.9181 | 0.8603 | -0.8050 | 0.3559 | 0.4193 | -0.9677 | 0.7255 | -0.8878 | 0.8457 | 0.9556 | 0.8220 | -0.1137 | 0.8006 | 0.8272 | -0.2385 | 0.7791 | 0.8469 | -0.6771 | -0.2827 | 0.8447 | 0.6758 | 0.9029 | 0.9471 | 0.9117 | 0.8253 | 0.9026 | 0.7625 | 0.8943 | -0.5781 | 0.9923 | 0.8727 | 0.8960 | 0.8114 | 0.6688 | 0.9254 | 0.9729 | 0.9079 | 0.7834 | 0.9297 | 0.2960 | 0.8110 | 0.4730 | -0.8715 | -0.8103 | 0.9430 | 0.8974 | 0.8756 | 0.8329 | 0.3629 | 0.4213 | 0.8562 | 0.8362 | 0.5451 | 0.8604 | -0.3923 | 0.7288 | 0.9312 | -0.9154 | 0.7182 | 0.1176 | 0.8702 | 0.7827 0.6615 0.7217 -0.5721 -0.5138 0.8108 0.7750 0.7986 0.5530 -0.6997 -0.8332 0.9184 0.9271 -0.9456 0.9118 0.7641 0.7031 0.9585 0.9985 0.7787 0.6661 0.7862 0.6595 0.6948 0.8021 0.7947 -0.7368 0.3186 -0.4171 1.0000 0.3562 0.9160 0.8378 0.7586 0.7533 0.8395 0.7013 0.7339 0.7673 0.7136 0.8055 |
| Leaf malate + citrate + isocitrate | 0.4952 | 0.6366 | -0.7653 | 0.9976 | 0.9963 | -0.5121 | 0.7312 | -0.6869 | 0.5926 | 0.3546 | 0.7738 | -0.8055 | 0.1546 | 0.7103 | -0.9510 | 0.8206 | 0.7119 | -0.9121 | -0.9692 | 0.5458 | 0.8762 | 0.6691 | 0.3344 | 0.3466 | 0.2979 | 0.3315 | 0.3621 | 0.2192 | -0.4481 | 0.2415 | 0.7278 | 0.1329 | 0.2165 | 0.8693 | 0.6577 | 0.4944 | 0.4770 | 0.1053 | 0.5827 | 0.4596 | 0.7966 | 0.9660 | -0.7105 | -0.3666 | 0.4689 | 0.5609 | 0.6499 | 0.7679 | -0.1509 | 0.9692 | 0.2715 | 0.7628 | 0.9682 | 0.5496 | -0.0349 | 0.8483 | 0.1605 | -0.1630 | 0.8866 | -0.7327 | 0.5449 | 0.8165 0.9320 0.8779 -0.7680 -0.9614 0.5573 -0.1001 0.2329 0.9467 -0.8929 -0.7536 0.5462 0.6157 -0.5525 0.5582 0.6802 0.8937 0.4762 0.3767 0.8227 0.9141 0.7776 0.6923 0.7491 0.8299 0.8261 -0.8803 0.9989 -0.4524 0.3562 1.0000 0.6218 0.6752 0.7098 0.6775 0.7644 0.7605 0.8268 0.8196 0.7710 0.6393 |
| Leaf NADP-ME | 0.8717 | 0.9631 | -0.9563 | 0.6096 | 0.6741 | -0.9647 | 0.8107 | -0.9594 | 0.9584 | 0.9463 | 0.9526 | -0.3118 | 0.7604 | 0.9406 | -0.5040 | 0.9346 | 0.9808 | -0.8809 | -0.5110 | 0.9372 | 0.9000 | 0.9913 | 0.9341 | 0.9133 | 0.8710 | 0.8864 | 0.8301 | 0.8689 | -0.6476 | 0.8673 | 0.9848 | 0.8556 | 0.8423 | 0.7579 | 0.9839 | 0.9504 | 0.9780 | 0.7725 | 0.9654 | 0.3356 | 0.9455 | 0.6971 | -0.9750 | -0.8825 | 0.9695 | 0.9876 | 0.9835 | 0.9756 | 0.1223 | 0.7205 | 0.8633 | 0.9457 | 0.7775 | 0.9556 | -0.5326 | 0.9062 | 0.8318 | -0.8515 | 0.9090 | -0.2192 | 0.9555 | 0.9339 0.8341 0.9089 -0.8285 -0.7781 0.9408 0.6974 0.6682 0.7908 -0.8971 -0.8543 0.9781 0.9941 -0.9780 0.9812 0.9477 0.9056 0.9384 0.9314 0.9513 0.8789 0.9550 0.8947 0.9188 0.9465 0.9502 -0.9106 0.5937 -0.5592 0.9160 0.6218 1.0000 0.9677 0.9305 0.9426 0.9788 0.9235 0.9323 0.9321 0.9315 0.9605 |
| Leaf NAD-ME | 0.8845 | 0.9969 | -0.9910 | 0.6490 | 0.7099 | -0.9427 | 0.8972 | -0.9764 | 0.9937 | 0.9126 | 0.9826 | -0.2488 | 0.8111 | 0.9884 | -0.5009 | 0.9712 | 0.9953 | -0.9159 | -0.5251 | 0.9831 | 0.9407 | 0.9850 | 0.9098 | 0.9224 | 0.9009 | 0.9082 | 0.9069 | 0.8663 | -0.7993 | 0.7843 | 0.9825 | 0.7882 | 0.8603 | 0.7819 | 0.9690 | 0.9261 | 0.9504 | 0.7995 | 0.9708 | 0.5211 | 0.9744 | 0.7809 | -0.9897 | -0.9260 | 0.9582 | 0.9807 | 0.9969 | 0.9811 | 0.2100 | 0.7742 | 0.8908 | 0.9747 | 0.8344 | 0.9870 | -0.4577 | 0.9592 | 0.8114 | -0.8299 | 0.9348 | -0.2075 | 0.9860 | 0.9714 0.8627 0.9440 -0.9269 -0.8413 0.9821 0.5927 0.7174 0.8645 -0.9337 -0.8149 0.9813 0.9784 -0.9685 0.9844 0.9841 0.9160 0.9314 0.8641 0.9689 0.9143 0.9882 0.9454 0.9650 0.9553 0.9638 -0.9357 0.6565 -0.7442 0.8378 0.6752 0.9677 1.0000 0.9911 0.9801 0.9773 0.9660 0.9703 0.9740 0.9669 0.9908 |
| Leaf NADP-MDH | 0.8387 | 0.9833 | -0.9866 | 0.6777 | 0.7354 | -0.8926 | 0.8992 | -0.9510 | 0.9834 | 0.8606 | 0.9725 | -0.2508 | 0.7859 | 0.9808 | -0.5248 | 0.9683 | 0.9813 | -0.9236 | -0.5454 | 0.9730 | 0.9541 | 0.9557 | 0.8605 | 0.8862 | 0.8821 | 0.8729 | 0.9066 | 0.8244 | -0.8261 | 0.6985 | 0.9588 | 0.7266 | 0.8372 | 0.7674 | 0.9313 | 0.8720 | 0.9142 | 0.7741 | 0.9356 | 0.5627 | 0.9647 | 0.8128 | -0.9696 | -0.9154 | 0.9177 | 0.9531 | 0.9777 | 0.9659 | 0.1807 | 0.8128 | 0.8637 | 0.9602 | 0.8575 | 0.9726 | -0.4460 | 0.9662 | 0.7510 | -0.7758 | 0.9365 | -0.2579 | 0.9693 | 0.9678 0.8635 0.9476 -0.9674 -0.8730 0.9783 0.5218 0.6751 0.8933 -0.9401 -0.7660 0.9507 0.9425 -0.9284 0.9556 0.9884 0.9159 0.8833 0.7907 0.9636 0.9241 0.9870 0.9665 0.9809 0.9416 0.9537 -0.9336 0.6968 -0.7990 0.7586 0.7098 0.9305 0.9911 1.0000 0.9863 0.9594 0.9801 0.9769 0.9747 0.9776 0.9874 |
| Leaf NAD-MDH | 0.7762 | 0.9645 | -0.9686 | 0.6466 | 0.7110 | -0.8730 | 0.8177 | -0.9168 | 0.9761 | 0.8704 | 0.9451 | -0.2624 | 0.7583 | 0.9456 | -0.5240 | 0.9405 | 0.9763 | -0.9016 | -0.5142 | 0.9567 | 0.9463 | 0.9474 | 0.8661 | 0.8776 | 0.8901 | 0.8498 | 0.8865 | 0.8282 | -0.7408 | 0.6921 | 0.9495 | 0.7719 | 0.8566 | 0.6943 | 0.9143 | 0.8462 | 0.9408 | 0.7830 | 0.9083 | 0.4235 | 0.9355 | 0.7539 | -0.9493 | -0.9179 | 0.9148 | 0.9646 | 0.9713 | 0.9601 | 0.0480 | 0.8082 | 0.8580 | 0.9244 | 0.8217 | 0.9659 | -0.5821 | 0.9339 | 0.7357 | -0.7795 | 0.9189 | -0.3175 | 0.9590 | 0.9380 0.8231 0.9249 -0.9590 -0.8508 0.9813 0.5995 0.5844 0.8557 -0.9182 -0.7193 0.9435 0.9381 -0.9135 0.9513 0.9998 0.9111 0.8519 0.7852 0.9552 0.9058 0.9763 0.9904 0.9932 0.9224 0.9384 -0.9092 0.6626 -0.7257 0.7533 0.6775 0.9426 0.9801 0.9863 1.0000 0.9564 0.9919 0.9684 0.9486 0.9898 0.9956 |
| Leaf PEPC | 0.8543 | 0.9654 | -0.9890 | 0.7508 | 0.8039 | -0.9277 | 0.8717 | -0.9712 | 0.9511 | 0.8725 | 0.9874 | -0.4336 | 0.6884 | 0.9667 | -0.6412 | 0.9841 | 0.9930 | -0.9591 | -0.6561 | 0.9251 | 0.9673 | 0.9883 | 0.8597 | 0.8521 | 0.8084 | 0.8292 | 0.7990 | 0.7827 | -0.6824 | 0.7714 | 0.9970 | 0.7371 | 0.7628 | 0.8460 | 0.9782 | 0.9115 | 0.9274 | 0.6809 | 0.9500 | 0.4310 | 0.9865 | 0.8274 | -0.9886 | -0.8366 | 0.9231 | 0.9594 | 0.9810 | 0.9996 | 0.0929 | 0.8393 | 0.7961 | 0.9794 | 0.8894 | 0.9382 | -0.4359 | 0.9699 | 0.7356 | -0.7516 | 0.9749 | -0.3463 | 0.9370 | 0.9829 0.9238 0.9750 -0.8935 -0.8889 0.9288 0.5417 0.6314 0.8991 -0.9686 -0.8875 0.9509 0.9764 -0.9503 0.9561 0.9612 0.9700 0.9008 0.8593 0.9940 0.9577 0.9900 0.9213 0.9543 0.9905 0.9935 -0.9751 0.7423 -0.6146 0.8395 0.7644 0.9788 0.9773 0.9594 0.9564 1.0000 0.9601 0.9829 0.9827 0.9679 0.9616 |
| Leaf PEPP | 0.7394 | 0.9433 | -0.9705 | 0.7322 | 0.7889 | -0.8349 | 0.8209 | -0.9045 | 0.9490 | 0.8119 | 0.9483 | -0.3678 | 0.6777 | 0.9369 | -0.6226 | 0.9535 | 0.9690 | -0.9382 | -0.6100 | 0.9224 | 0.9754 | 0.9328 | 0.8044 | 0.8162 | 0.8258 | 0.7864 | 0.8313 | 0.7529 | -0.7101 | 0.6270 | 0.9455 | 0.6941 | 0.7843 | 0.7349 | 0.8986 | 0.8065 | 0.9007 | 0.6986 | 0.8790 | 0.4264 | 0.9434 | 0.8154 | -0.9408 | -0.8621 | 0.8696 | 0.9341 | 0.9530 | 0.9639 | -0.0172 | 0.8761 | 0.7888 | 0.9258 | 0.8781 | 0.9322 | -0.5407 | 0.9535 | 0.6545 | -0.6985 | 0.9489 | -0.4234 | 0.9241 | 0.9501 0.8678 0.9519 -0.9735 -0.9070 0.9511 0.5091 0.5224 0.9049 -0.9494 -0.7359 0.9087 0.9152 -0.8801 0.9185 0.9913 0.9455 0.8083 0.7338 0.9703 0.9434 0.9814 0.9911 0.9997 0.9390 0.9538 -0.9371 0.7480 -0.7057 0.7013 0.7605 0.9235 0.9660 0.9801 0.9919 0.9601 1.0000 0.9849 0.9615 0.9993 0.9786 |
| Leaf PK | 0.7981 | 0.9517 | -0.9904 | 0.8054 | 0.8534 | -0.8635 | 0.8848 | -0.9440 | 0.9403 | 0.8014 | 0.9820 | -0.4511 | 0.6510 | 0.9635 | -0.6887 | 0.9894 | 0.9798 | -0.9808 | -0.7005 | 0.9140 | 0.9948 | 0.9541 | 0.7917 | 0.8022 | 0.7801 | 0.7811 | 0.7978 | 0.7226 | -0.7232 | 0.6550 | 0.9716 | 0.6480 | 0.7268 | 0.8412 | 0.9333 | 0.8421 | 0.8787 | 0.6405 | 0.9073 | 0.4972 | 0.9823 | 0.8856 | -0.9680 | -0.8205 | 0.8698 | 0.9243 | 0.9597 | 0.9863 | 0.0547 | 0.9038 | 0.7570 | 0.9683 | 0.9343 | 0.9210 | -0.4099 | 0.9892 | 0.6512 | -0.6746 | 0.9876 | -0.4243 | 0.9169 | 0.9874 0.9382 0.9902 -0.9547 -0.9443 0.9255 0.4356 0.5770 0.9507 -0.9880 -0.8330 0.9123 0.9323 -0.8997 0.9200 0.9710 0.9802 0.8392 0.7615 0.9954 0.9820 0.9956 0.9534 0.9810 0.9815 0.9890 -0.9829 0.8126 -0.6926 0.7339 0.8268 0.9323 0.9703 0.9769 0.9684 0.9829 0.9849 1.0000 0.9926 0.9884 0.9604 |
| Leaf CS | 0.8555 | 0.9635 | -0.9954 | 0.7997 | 0.8446 | -0.8927 | 0.9317 | -0.9702 | 0.9436 | 0.8137 | 0.9956 | -0.4277 | 0.6821 | 0.9829 | -0.6637 | 0.9994 | 0.9806 | -0.9804 | -0.6980 | 0.9243 | 0.9830 | 0.9642 | 0.8063 | 0.8213 | 0.7814 | 0.8101 | 0.8100 | 0.7375 | -0.7679 | 0.6937 | 0.9789 | 0.6430 | 0.7233 | 0.8854 | 0.9529 | 0.8774 | 0.8707 | 0.6471 | 0.9346 | 0.5718 | 0.9965 | 0.8993 | -0.9820 | -0.8212 | 0.8844 | 0.9220 | 0.9648 | 0.9870 | 0.1634 | 0.8769 | 0.7717 | 0.9897 | 0.9357 | 0.9263 | -0.3215 | 0.9978 | 0.6877 | -0.6953 | 0.9871 | -0.3491 | 0.9257 | 0.9991 0.9527 0.9928 -0.9345 -0.9329 0.9196 0.4152 0.6569 0.9518 -0.9885 -0.8751 0.9245 0.9433 -0.9209 0.9292 0.9536 0.9712 0.8765 0.7924 0.9927 0.9782 0.9939 0.9201 0.9569 0.9896 0.9930 -0.9888 0.8043 -0.7173 0.7673 0.8196 0.9321 0.9740 0.9747 0.9486 0.9827 0.9615 0.9926 1.0000 0.9659 0.9491 |
| Leaf ACO | 0.7454 | 0.9438 | -0.9738 | 0.7450 | 0.8010 | -0.8421 | 0.8224 | -0.9105 | 0.9472 | 0.8148 | 0.9536 | -0.3927 | 0.6673 | 0.9386 | -0.6398 | 0.9589 | 0.9730 | -0.9461 | -0.6276 | 0.9188 | 0.9799 | 0.9396 | 0.8058 | 0.8137 | 0.8175 | 0.7832 | 0.8197 | 0.7488 | -0.6961 | 0.6381 | 0.9531 | 0.6951 | 0.7754 | 0.7499 | 0.9075 | 0.8144 | 0.9034 | 0.6874 | 0.8839 | 0.4154 | 0.9497 | 0.8218 | -0.9463 | -0.8535 | 0.8723 | 0.9366 | 0.9559 | 0.9709 | -0.0240 | 0.8821 | 0.7817 | 0.9319 | 0.8860 | 0.9300 | -0.5353 | 0.9575 | 0.6543 | -0.6965 | 0.9569 | -0.4341 | 0.9223 | 0.9555 0.8795 0.9587 -0.9665 -0.9123 0.9466 0.5096 0.5211 0.9094 -0.9563 -0.7554 0.9110 0.9219 -0.8855 0.9208 0.9896 0.9552 0.8136 0.7445 0.9770 0.9509 0.9846 0.9866 0.9981 0.9484 0.9619 -0.9458 0.7574 -0.6859 0.7136 0.7710 0.9315 0.9669 0.9776 0.9898 0.9679 0.9993 0.9884 0.9659 1.0000 0.9772 |
| Leaf NADP-IDH | 0.8245 | 0.9818 | -0.9731 | 0.6095 | 0.6762 | -0.9118 | 0.8364 | -0.9397 | 0.9913 | 0.9100 | 0.9540 | -0.2175 | 0.8058 | 0.9601 | -0.4759 | 0.9428 | 0.9847 | -0.8890 | -0.4767 | 0.9771 | 0.9305 | 0.9657 | 0.9070 | 0.9170 | 0.9191 | 0.8934 | 0.9121 | 0.8719 | -0.7636 | 0.7525 | 0.9618 | 0.8135 | 0.8874 | 0.7031 | 0.9388 | 0.8890 | 0.9624 | 0.8223 | 0.9398 | 0.4422 | 0.9428 | 0.7321 | -0.9648 | -0.9418 | 0.9480 | 0.9830 | 0.9865 | 0.9650 | 0.1203 | 0.7667 | 0.8959 | 0.9380 | 0.7992 | 0.9852 | -0.5656 | 0.9309 | 0.7943 | -0.8304 | 0.9102 | -0.2401 | 0.9806 | 0.9415 0.8152 0.9178 -0.9381 -0.8213 0.9923 0.6412 0.6488 0.8340 -0.9082 -0.7441 0.9698 0.9612 -0.9452 0.9753 0.9969 0.8979 0.8952 0.8349 0.9524 0.8910 0.9763 0.9739 0.9797 0.9254 0.9393 -0.9046 0.6217 -0.7300 0.8055 0.6393 0.9605 0.9908 0.9874 0.9956 0.9616 0.9786 0.9604 0.9491 0.9772 1.0000 |
